# Trends in Structure and Ethylene Polymerization Reactivity of Transition-Metal Permethylindenyl-phenoxy (PHENI*) Complexes

**DOI:** 10.1021/acs.organomet.3c00503

**Published:** 2024-02-14

**Authors:** Clement G. Collins Rice, Justin A. Hayden, Adam D. Hawkins, Louis J. Morris, Zoë R. Turner, Jean-Charles Buffet, Dermot O’Hare

**Affiliations:** Chemistry Research Laboratory, Department of Chemistry, University of Oxford, 12 Mansfield Road, Oxford OX1 3TA, U.K.

## Abstract

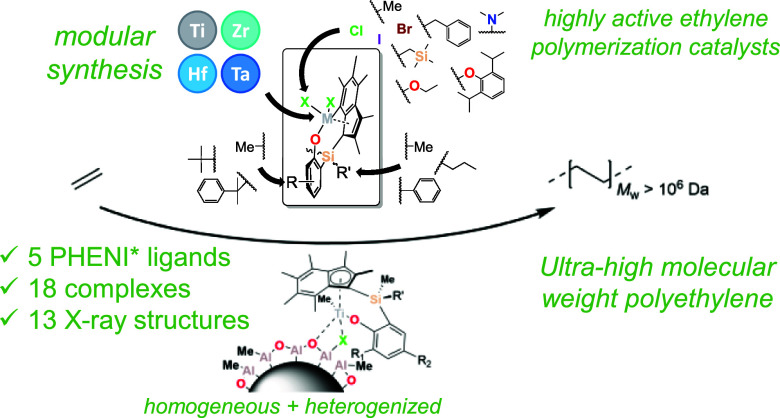

A family of *ansa*-permethylindenyl-phenoxy (PHENI*) transition-metal chloride complexes has been synthesized and characterized (**1–7**; {(η^5^-C_9_Me_6_)Me(R″)Si(2-R-4-R′-C_6_H_2_O)}MCl_2_; R,R′ = Me, ^*t*^Bu, Cumyl (CMe_2_Ph); R″ = Me, ^*n*^Pr, Ph; M = Ti, Zr, Hf). The ancillary chloride ligands could readily be exchanged with halides, alkyls, alkoxides, aryloxides, or amides to form PHENI* complexes [L]TiX_2_ (**8–17**; X = Br, I, Me, CH_2_SiMe_3_, CH_2_Ph, NMe_2_, OEt, ODipp). The solid-state crystal structures of these PHENI* complexes indicate that one of two conformations may be preferred, parametrized by a characteristic torsion angle (TA′), in which the η^5^ system is either disposed away from the metal center or toward it. Compared to indenyl PHENICS complexes, the permethylindenyl (I*) ligand appears to favor a conformation in which the metal center is more accessible. When heterogenized on solid polymethylaluminoxane (sMAO), titanium PHENI* complexes exhibit exceptional catalytic activity toward the polymerization of ethylene. Substantially greater activities are reported than for comparable PHENICS catalysts, along with the formation of ultrahigh-molecular-weight polyethylenes (UHMWPE). Catalyst–cocatalyst ion pairing effects are observed in cationization experiments and found to be significant in homogeneous catalytic regimes; these effects are also related to the influence of the ancillary ligand leaving groups in slurry-phase polymerizations. Catalytic efficiency and polyethylene molecular weight are found to increase with pressure, and PHENI* catalysts can be categorized as being among the most active for the controlled synthesis of UHMWPE.

## Introduction

Traditional heterogeneous polymerization catalysts are now commonly replaced by soluble Kaminsky metallocenes for applications where fine control over the polymer molecular weight and distribution, comonomer content, and tacticity is required.^1,2^ The metallocene catalysts can be tuned sterically and electronically to enhance catalytic activity and give control over both macroscopic and microscopic properties of the polymer.^3^ The improved performance of group four constrained geometry complexes (CGCs), bearing an *ansa*-cyclopentadienyl-amido ligand, compared to more classical MCp_2_ (Cp = η^5^-C_5_H_5_) Kaminsky metallocenes, is believed to be a result of the less crowded coordination sphere, the smaller Cp–M–N bite angle, and a suppression of chain transfer processes.^4^ This leads to increased activities, generally higher molecular weight polymers, and also the ability to incorporate comonomers with high efficiency.^5^ The development of new generations of CGCs and related postmetallocene catalysts—many of which incorporate phenoxide ligands—is an area of ongoing intensive research.^6−12^

The phenoxy-induced complexes of Sumitomo (PHENICS) are a class of *ansa*-bridged postmetallocenes bearing an apical Cp derivative and a basal aryloxide motif: {(η^5^-^R^Cp)R_2_E(ArO)}MX_2_. Complexes have been reported with titanium, zirconium, hafnium, and tantalum metal centers; Me_2_Si, Et_2_Si, MePhSi, MeEtSi, MeCySi, silolyl (C_4_H_8_Si), and Me_2_C *ansa* bridges; Cp, ^^*t*^Bu^Cp, Cp*, indenyl (Ind), fluorenyl (Flu), and ^^*t*^Bu_2_^Flu apical ligands; and phenoxy substituents including Me, ^*i*^Pr, ^*t*^Bu, and 1-adamantyl.^13−19^ PHENICS complexes have been shown to be useful catalysts for the copolymerization of ethylene and 1-hexene at elevated temperatures, producing high-molecular-weight copolymers with high comonomer incorporation.^18^

Following the synthesis of permethylindenyl-based metallocenes,^20−24^ Williams *et al.* reported the synthesis of permethylindenyl CGC analogues of the type ^Me_2_^SB(^R^N,I*)TiCl_2_ ({(η^5^-C_9_Me_6_)Me_2_Si(^R^N)}TiCl_2_, where I* = η^5^-C_9_Me_6_).^25,26^ Ethylene polymerization activities up to three times that of the Cp* (η^5^-C_5_Me_5_) derivatives were reported, with more electron-donating amido fragments shown to result in increased catalytic activities.^25^ More recently, we have reported the permethylindenyl-phenoxy (PHENI*) ligand, offering exceptional performance in olefinic polymerizations producing ultrahigh-molecular-weight homopolymers and highly tunable copolymers.^27−30^

The control and tuneability afforded by single-site catalysts is brought into the realms of industrial-scale applicability through heterogenization.^31^ The immobilization of single-site catalysts on suitable carriers facilitates their use in slurry-phase or fluidized bed reactors previously used for Ziegler–Natta reactions with minimal modifications as well as ensuring the formation of polymer particles with dispersed morphologies and desirable bulk densities.^32,33^ Many inert inorganic supports such as silica,^34,35^ alumina,^36^ zirconia,^37^ metal−organic frameworks,^38^ and layered double hydroxides^39,40^ have been developed, but these need pretreating to enable metallocene immobilization.^41^ Solid polymethylaluminoxane (sMAO) has been introduced as a dual-function activator and catalyst support. The structure of sMAO and of supported metallocenes is poorly understood, but many catalysts of this type yield polymers with narrow molecular-weight distributions and apparent single-site behavior, suggesting that a similar polymerization mechanism is occurring using both solution-phase metallocenes and slurry-phase supported metallocenes.^41,42^

In this work, we expand the scope of PHENI* complexes, reporting the synthesis and characterization of 15 group four complexes bearing five PHENI* ligands. Complexes of zirconium and hafnium are reported as well as titanium complexes bearing chiral-at-silicon ligands and a range of ancillary donors. Structural trends are examined in solution and in the solid state, and are related to the ethylene polymerization performance of heterogenized catalysts.

## Results and Discussion

### Synthesis of PHENI* Ligands

The synthesis of PHENICS ligands has been reasonably well-developed: substituted phenols are first *ortho*-brominated using either Br_2_ or N-bromosuccinimide, and the phenolic hydroxyl protected as a methoxy group,^17^ though it was subsequently shown that an allyloxy protecting group led to improved yields.^14^ The resulting aryl bromide is exchanged for lithium and the *ansa* bridge incorporated by reaction with either 6,6-dimethylfulvene or the desired R_*a*_R_*b*_SiCl_2_. Reactions with the lithium or sodium salt of the desired Cp derivative leads to the proligands in yields of 75–86%. Group four complexes were made by in situ deprotection with ^*n*^BuLi followed by the addition of TiCl_4_, TiCl_2_(NMe_2_)_2_, ZrCl_4_, ZrCl_2_(NMe_2_)_2_, or HfCl_2_(NMe_2_)_2_ in yields of 2–50%.^14,17,18^ The amido metal sources were found to react more selectively toward the deprotected dilithium salt, and subsequent chlorination could be accomplished with Me_3_SiCl.^18^

**Scheme 1:**
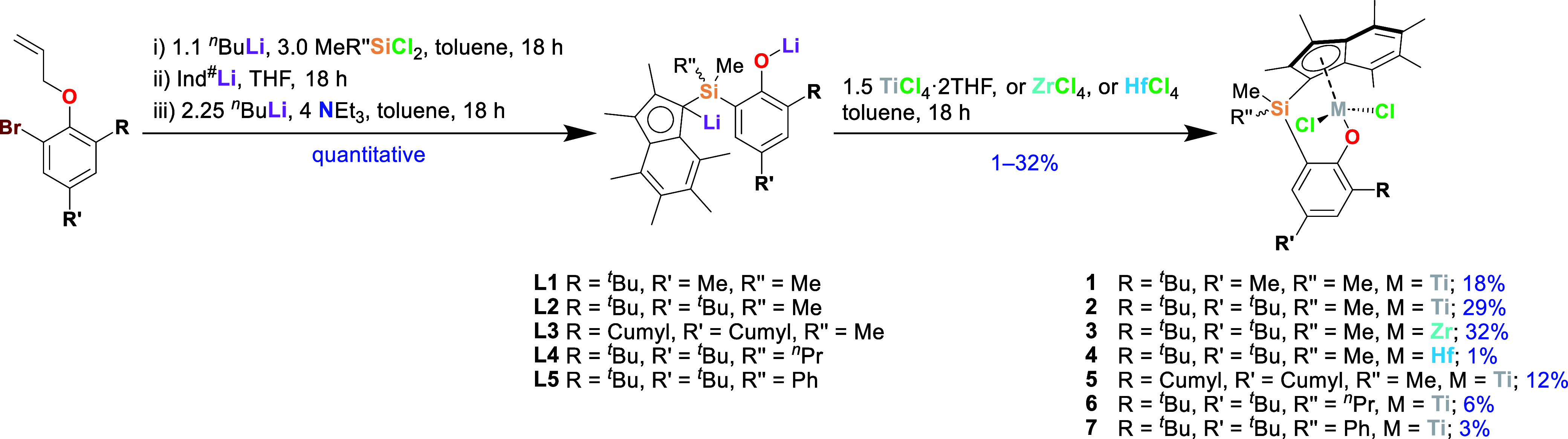
Synthesis of Group Four PHENI* Dichloride Complexes**1–7** via Ligand Salts**L1–L5**; All Chiral Compounds Formed as Racemic Mixtures; Cumyl = CMe_2_Ph; Yields Given Are for Isolated Recrystallized Complexes

The related stepwise synthesis of permethylindenyl analogues of PHENICS, PHENI* proligands ^Me_2_^SB(^R,R′^ArOAllyl,I*)H (I* = C_9_Me_6_; R,R′ = (Me,^*t*^Bu; **P1**), (^*t*^Bu,^*t*^Bu; **P2**), (CMe_2_Ph,CMe_2_Ph; **P3**); Cumyl = CMe_2_Ph) has previously been reported.^27^ Subsequent optimizations have been performed to the synthesis: isolation and workup of each intermediate has not been proved necessary and the transformation of the allyl-protected bromophenols to lithium ligand salts can be achieved in a one-pot process (Scheme 1). Allyl-protected bromophenols were *ortho* silylated via lithium–halogen exchange and then combined with hexamethylindenyl lithium (Ind^#^Li) to form allyloxy-protected proligands. The proligands were treated with ^*n*^BuLi/NEt_3_ in toluene, and ligand salts ^Me_2_^SB(^R,R′^ArO,I*)Li_2_ (**L1–L3**) were obtained as highly air-sensitive orange powders in quantitative yields following removal of the volatile components under vacuum. The addition of Et_3_N to the deprotonation and deprotection step had been previously found to significantly increase yields of isolated PHENICS complexes.^17^ Here, the presence of 1-heptene and NEt_3_ was identified in the distillate by ^1^H NMR spectroscopy (Figure S22), confirming the proposed deprotection mechanism—^*n*^Bu^–^ attack at the allyl group—and the catalytic role of the tertiary amine. Chiral ligands with “unsymmetrical” silane bridges, *rac*-^Me,R″^SB(^^*t*^Bu_2_^ArO,I*)Li_2_ (R″ = ^*n*^Pr (**L4**), Ph (**L5**)), were synthesized analogously using Me(^*n*^Pr)SiCl_2_ or Me(Ph)SiCl_2_, respectively, as the silicon source. **L1–L5** are observed to decompose over the course of a few weeks when stored in the solid state at −30 °C.

Single crystals suitable for X-ray diffraction of the proligands **P2** and **P3** were obtained from saturated hexane solutions at −30 °C. **P2** crystallized in the monoclinic space group *I*2/*a*, whereas **P3** adopted the triclinic space group *P*1̅ (Figures S76 and S77). The bridging silicon atoms adopt ideal tetrahedral geometries (mean C–Si–C = 109.4 ± 3.5°). In both cases, the I*, ArO, and Allyl groups are all essentially planar and approaching mutual perpendicularity in **P2** (interplanar angles 76.4–79.3°) but distorted away from this arrangement in **P3** (47.6–83.1°).

### Group Four PHENI* Halide Complexes

Direct combination of the crude dilithium ligand salts with TiCl_4_·2THF in toluene afforded PHENI* titanium dichloride complexes in isolated recrystallized yields of 20–30%, comparable to the yields reported for PHENICS complexes. In addition to the previously reported complexes **1**, **2**, and **5**, titanium complexes ^Me,^*n*^Pr^SB(^^*t*^Bu_2_^ArO,I*)TiCl_2_ (**6**) and ^Me,Ph^SB(^^*t*^Bu_2_^ArO,I*)TiCl_2_ (**7**) of the mixed-bridge ligands **L4** (Me,^*n*^Pr) and **L5** (Me,Ph) were prepared and isolated. The comparatively low isolated yields in these cases (6 and 3%, respectively) are attributed to poorer differential solubility with respect to impurities, with extraction from the bulk crude proving challenging. Heavier group four congeners of **2** were synthesized from ligand **L2** by reaction with either ZrCl_4_ or HfCl_4_. The zirconium complex, ^Me_2_^SB(^^*t*^Bu_2_^ArO,I*)ZrCl_2_ (**3**), was synthesized as a dark yellow solid in a 32% isolated recrystallized yield. The hafnium analogue, ^Me_2_^SB(^^*t*^Bu_2_^ArO,I*)HfCl_2_ (**4**), was isolated by recrystallization from pentane at −80 °C as a yellow solid in a low isolated yield of 1%. As well as challenges associated with obtaining sufficiently pure material from the crude bulk material, the low yield is attributed to the poor solubility of HfCl_4_ in toluene and poor reaction selectivity. An additional factor is the apparently poor stability of **L1–L5** when stored in the solid state.

The same salt metathesis protocol can be successfully applied beyond group four metals, and a tantalum(V) complex, ^Me_2_^SB(^^*t*^Bu_2_^ArO,I*)TaCl_3_ (**18**), was synthesized from **L2** and TaCl_5_. Complex **18** was isolated and fully characterized including by X-ray crystallography (see the Supporting Information for further details).

All complexes were analyzed by multinuclear NMR spectroscopy. The ^1^H, ^13^C{^1^H} and ^29^Si NMR spectra all show similar features and are consistent with the proposed *C*_1_-symmetry structures (Figures S23–S43). Notably, the six diagnostic I*Me singlets were observed in the ^1^H NMR spectra between δ 2.66 and 1.90 ppm and the *meta*-aryl protons as a pair of doublets between δ 7.58 and 7.19 ppm with a ^4^*J*_H–H_ constant of 2 Hz. For complex **6**, the SiC*H*_3_ resonance is a singlet at δ 0.73 ppm, and the SiCH_2_CH_2_C*H*_3_ resonance is a triplet at δ 0.94 ppm (^3^*J*_H–H_ = 7.1 Hz). The SiC*H*_2_C*H*_2_CH_3_ resonances are obscured by the *tert*-butyl signals but can be clearly identified in the ^1^H–^13^C HSQC NMR spectrum at δ^2^ (1.37, 20) and (1.43, 18) ppm, with the negative phase DEPT editing confirming the assignment of methylene groups. In the ^1^H NMR spectrum of **7**, the SiC*H*_3_ singlet is at δ 1.04 ppm, and the SiC_6_*H*_5_ resonances are multiplets at δ 7.23–7.32 and 7.78–7.82 ppm.

**Figure 1:**
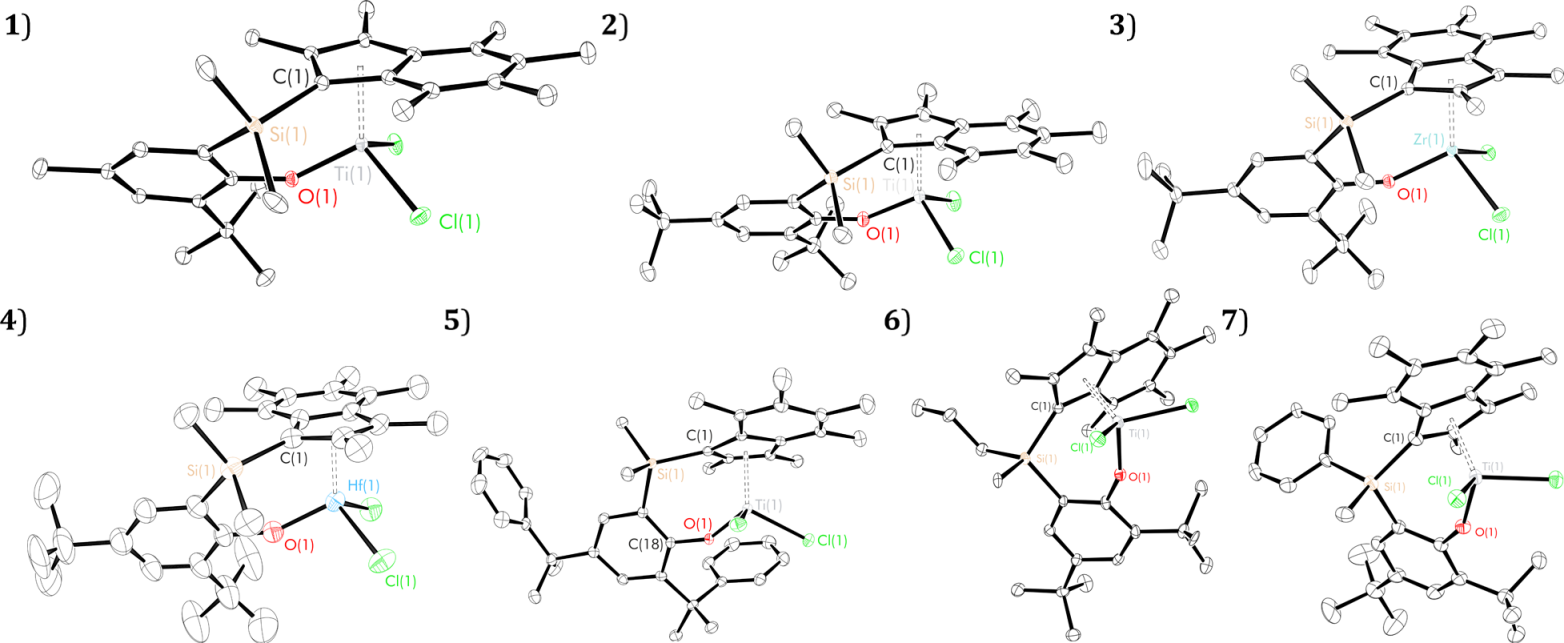
Solid-state ellipsoid plots of group four PHENI* dichloride complexes ^Me_2_^SB(^^*t*^Bu,Me^ArO,I*)TiCl_2_ (**1**), ^Me_2_^SB(^^*t*^Bu_2_^ArO,I*)TiCl_2_ (**2**),^27^^Me_2_^SB(^^*t*^Bu_2_^ArO,I*)ZrCl_2_ (**3**), ^Me_2_^SB(^^*t*^Bu_2_^ArO,I*)HfCl_2_ (**4**), ^Me_2_^SB(^Cumyl_2_^ArO,I*)TiCl_2_ (**5**), (*R*)-*Z*-^Me^,*n*^Pr^SB(^^*t*^Bu_2_^ArO,I*)TiCl_2_ (**6**), and (*R*)-*E*-^Me,Ph^SB(^^*t*^Bu_2_^ArO,I*)TiCl_2_ (**7**). Hydrogen atoms omitted for clarity; one stereoisomer shown; thermal ellipsoids drawn at 30% [or 15% (**6**)] probability. The solid-state structure of **6** contains crystallographically independent molecules (*Z*′ = 2), one residue depicted.

In addition to the previously reported solid-state structure for complex **2**, X-ray crystal structures of PHENI* dichloride complexes **1** and **3–7** are reported in the current work, enabling a detailed investigation of structural trends (Figure 1 and Table 1). The solid-state structures of PHENI* complexes are tetrahedral at the metal center, with τ_4_ geometry indices of 0.89–0.92.^43^ Measured Ti–I_cent_* distances are similar to permethylindenyl post metallocene complexes,^25^ to the analogous Ti–Cp_cent_ for PHENICS complexes^14,17,18^ and titanium constrained geometry complexes.^44,45^ The Ti–O bond lengths and I_cent_*–Ti–O angles are in the same range as PHENICS complexes.^14,17,18^ The Ti–O bonds are slightly shorter than in other phenoxy-titanium systems such as phenoxy-tetrazole,^46^ phenoxy-imine,^47^ phenoxy-amine,^48,49^ bis-phenoxy,^50^ and phenoxy-azo ligands.^51^

**Table 1:** Selected Geometric Parameters (Lengths in Å, Angles in °) for PHENI* Complexes**1–7** and the Ind-PHENICS Complex**A**^a^

complex	**A**(14)	**1**	**2**(27)	**3**	**4**	**5**	**6**	**7**
space group	*P*2_1_/*c*	*Pbca*	*P*1̅	*P*1̅	*P*1̅	*P*1̅	*Pn*2_1_*a*	*P*2_1_/*c*
M(1)–O(1)	1.7722(18)	1.7949(12)	1.7838(1)	1.9236(16)	1.918(3)	1.791(2)	1.783(4)	1.798(6)
M(1)–I_cent_*	2.031	2.0242(9)	2.0156(1)	2.1703(11)	2.140(2)	2.0243(17)	2.010(3)	2.0280(2)
O(1)–M(1)–I_cent_*	115.86	115.49(5)	114.16(1)	115.30(6)	115.32(12)	114.53(10)	115.13(14)	116.42(1)
C(1)–Si(1)–C(23)–C(18)	11.8(3)	45.3(2)	35.89(1)	44.4(2)	41.2(5)	27.1(4)	33.2(5)	44.75(1)
TA′	71.6	31.8(1)	33.0(2)	80.2(2)	79.2(5)	42.9(3)	86.4(5)	35.8(7)
I*–ArO	60.85	54.38	45.08	66.52	64.00	46.31	49.25	51.86
Δ_M__–__C_	0.180	0.098	0.096	0.122	0.118	0.136	0.078	0.084
τ_4_	0.90	0.90	0.90	0.92	0.91	0.92	0.90	0.89
conformation type	A	B	B	A	A	B	A	B

a Estimated standard deviations (ESDs) given in parentheses.

**Figure 2:**
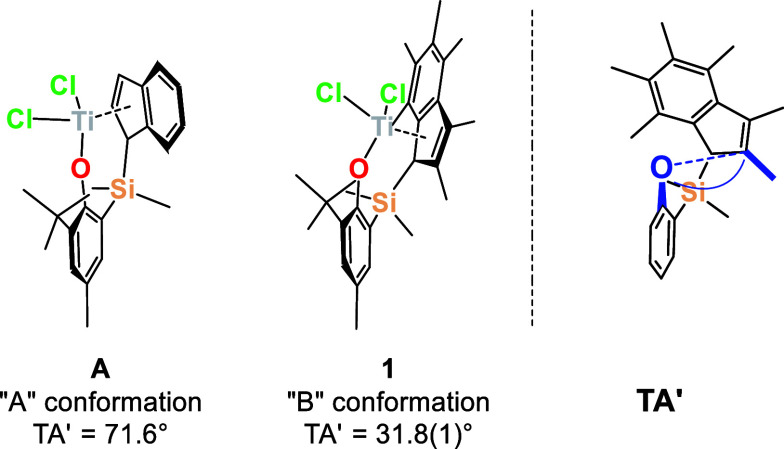
Schematic solid-state crystallographic conformations, left, of Ind-PHENICS (**A**) and the PHENI* analogue (**1**); right, the characteristic torsion angle TA′.

Compared to the indenyl–PHENICS complex reported by Hanaoka *et al.*, ^Me_2_^SB(^^*t*^Bu,Me^ArO,Ind)TiCl_2_ (**A**),^14^ the PHENI* complexes are significantly more twisted, as defined by the larger torsion angle C(1)–Si(1)–C(23)–C(18) = 45.3(2) (**1**), 35.89(1) (**2**), and 27.1(4)° (**5**) (*cf.* 11.75° (**A**)) and a smaller I*–ArO interplanar dihedral angle (54.38 (**1**), 45.08 (**2**), and 46.31° (**5**) *cf.* 60.85° (**A**)). This can be summarized by the TA′ parameter (Figure 2) calculated as the torsion angle C(10)–C(2)–O(1)–C(18). It is noted that the synthesized complexes adopt one of two conformations: the “A” geometry is defined as having TA′ between 70 and 80°, while the “B” geometry has TA′ between 30 and 40°. Under this parametrization, it is interesting to note that, while **A** crystallizes with the A-type geometry, the three analogous PHENI* complexes (**1**, **2**, and **5**) crystallize with the B conformation. This may have implications in catalysis, where the geometry around the metal center influences the energy barrier for olefin coordination. In the B conformation, the I* system is oriented away from the active center, counterintuitively leaving the titanium atom more accessible than in the less bulky indenyl complex.

In the solid-state structure of **5**, the *ortho*-cumyl phenyl ring is held at a distance of Ti–Ph_cent_ = 4.657 Å from the metal center in a conformation which may enable a degree of long-range π–d stabilization despite the steric incentive to dispose the ring away from the metal center.

The zirconium (**3**) and hafnium (**4**) complexes are isostructural as expected from the similar ionic radii; *r*_Zr_^4+^ = 0.59 Å and *r*_Hf_^4+^ = 0.58 Å.^52,53^ Both display A-type conformation in the solid state, with comparable M–O and M–I_cent_* distances, and O–M–I_cent_* and TA′ angles. By contrast, the smaller radius of Ti^4+^ (0.42 Å) and higher charge density likely account for shorter M–L bonds, a smaller bridge torsion angle, a smaller interplanar dihedral angle, and B-type conformation in **2**. The heavy group four congeners have a dihedral angle and TA′ torsion angle comparable to **A**, perhaps indicating that the larger metals compensate for the steric bulk of the permethylated ligand.

Complexes **6** and **7**, where the ligand is chiral at silicon, have TA′ parameters of 56.4(5) and 35.8(7)°, respectively. The solid-state geometry of **7** is consistent with B-type conformation, with a very similar TA′ angle to that of **2** (33.0(2)°), which shows that additional steric bulk at the bridge does not significantly impact the ligand conformation preference. Conversely, **6** has a solid-state structure toward the A-type conformation, demonstrating the delicate balance of steric, electronic, and packing factors in determining crystallographic conformation. In both cases, racemic mixtures are obtained with a 1:1 ratio of the (*R*) and (*S*) configurations at the silicon. Notably, though, the A–B conformation results in the possibility of diastereomers: in the solid-state structure of **6**, the I* C_6_ ring is orientated *cis* to the Si^*n*^Pr (*Z*), whereas **7** displays the (*E*) configuration with the I* C_6_ disposed *trans* to the SiPh.

### Ancillary Ligand Substitution

**Scheme 2:**
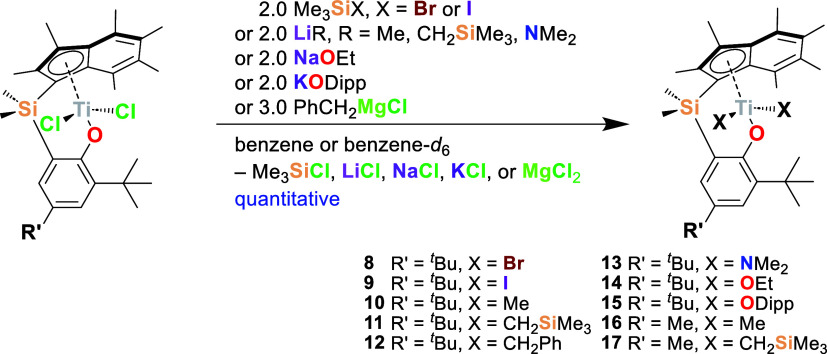
Synthesis of Titanium PHENI* Complexes **8–17** by Ancillary Ligand Substitution; Dipp = 2,6-Diisopropylphenyl; Reactions Are Deemed Quantitative by ^1^H NMR Spectroscopy

The chloride ancillary ligands of PHENI* complexes could readily be replaced by halide, alkyl, alkoxide, aryloxide, and amide groups through stoichiometric reactions with suitable reagents (Scheme 2).

**Figure 3:**
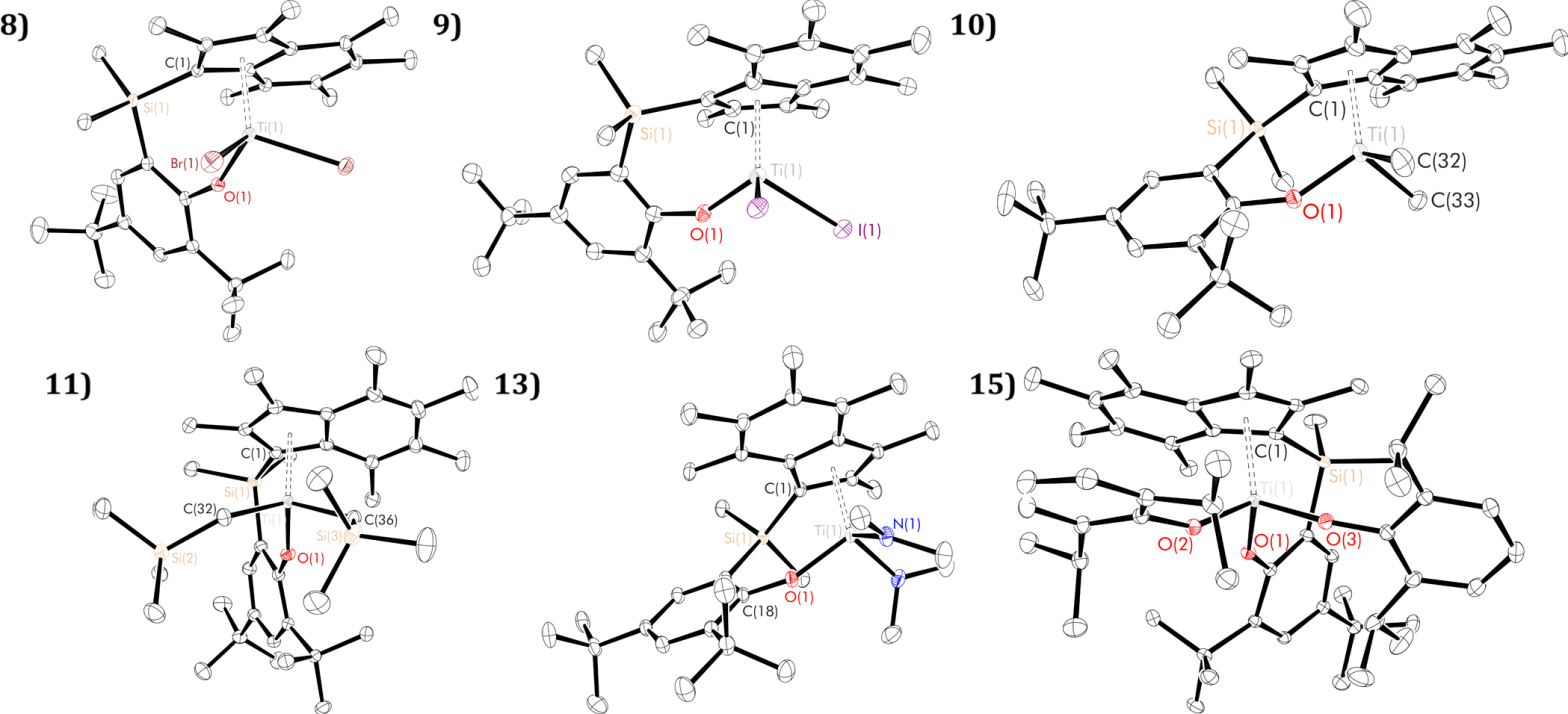
Solid-state ellipsoid plots of group four PHENI* complexes ^Me_2_^SB(^^*t*^Bu_2_^ArO,I*)TiBr_2_ (**8**), ^Me_2_^SB(^^*t*^Bu_2_^ArO,I*)TiI_2_ (**9**; residue 1, **9** B, shown), ^Me_2_^SB(^^*t*^Bu_2_^ArO,I*)TiMe_2_ (**10**), ^Me_2_^SB(^^*t*^Bu_2_^ArO,I*)Ti(CH_2_SiMe_3_)_2_ (**11**), ^Me_2_^SB(^^*t*^Bu_2_^ArO,I*)Ti(NMe_2_)_2_ (**13**), and ^Me_2_^SB(^^*t*^Bu_2_^ArO,I*)Ti(ODipp)_2_ (**15**). Hydrogen atoms omitted for clarity; thermal ellipsoids drawn at 30% probability. Asymmetric unit of **9** plotted in Figure S78.

The syntheses of bromide- and iodide-substituted complexes ^Me_2_^SB(^^*t*^Bu_2_^ArO,I*)TiX_2_ (X = Br, **8**; X = I, **9**) can be achieved quantitatively using Me_3_SiBr and Me_3_SiI, respectively, and have been previously described.^27^ Both complexes crystallize in the triclinic space group *P*1̅ with the solid-state structure of **8** exhibiting the A geometry (TA′ = 78.8(2)°; Figure 3 and Table 2). Uniquely, the solid-state structure of **9** contains crystallographically distinct molecules in the asymmetric unit (*Z*′ = 3), which are in a 2:1 ratio of and A and B conformations, having TA′ angles of 36.7(5) and 72.4(7)°, respectively (Figure S78). This is excellent evidence for the existence of the proposed A–B conformational regime, with the observed symmetric ^1^H NMR spectrum of complex **9** suggestive of a time-averaged structure in solution. Rapid conformational interconversion is observed on the NMR spectroscopic timescale even at −70 °C (Figure S50), indicative of a low energy barrier for interconversion, presumably via hemilabile I* coordination and rotation about the Si–I* bond. Single-point energies calculated by density functional theory (DFT) indicate only minor differences in thermodynamic preference (on the order of *k*_B_*T*) between the A and B conformations (Table S2). The observed crystallographic conformations may result from changes in the position of the A ⇌ B equilibria, which is clearly a finely balanced system with small changes in steric and electronic effects able to promote one solid-state conformation over the other.

**Table 2:** Selected Geometric Parameters (Lengths in Å, Angles in °) for PHENI* Complexes**8–11**, **13**, **15**^a^

complex	**8**	**9-A**	**9-B**	**10**	**11**	**13**	**15**
space group	*P*1̅	*P*1̅	*P*1̅	*P*1̅	*P*1̅	*P*1̅	*P*1̅
Ti(1)–O(1)	1.7960(14)	1.794(5)	1.793(5)	1.8021(9)	1.8174(1)	1.8603(13)	1.8595(1)
Ti(1)–I_cent_*	2.0342(10)	2.033(4)	2.031(3)	2.0357(8)	2.0842(1)	2.1003(9)	2.0971(1)
O(1)–Ti(1)–I_cent_*	118.29(6)	117.80(19)	114.92(18)	118.02(4)	119.10(1)	113.18(5)	112.17(1)
C(1)–Si(1)–C(23)–C(18)	38.16(18)	17.1(8)	36.3(7)	40.25(15)	1.69(1)	34.46(17)	7.51(1)
TA′	78.8(2)	72.4(7)	36.7(5)	33.8(1)	65.4(2)	78.5(1)	62.5(1)
I*–ArO	61.46	58.50	48.53	54.24	70.86	51.92	56.00
Δ_M__–__C_	0.174	0.210	0.165	0.117	0.287	0.054	0.245
τ_4_	0.88	0.89	0.89	0.89	0.88	0.86	0.90
conformation type	A	A	B	B	intermediate	A	intermediate

a Estimated standard deviations (ESDs) given in parentheses.

Alkyl complexes with methyl and neosilyl (CH_2_SiMe_3_) ancillary ligands were formed from both **1** and **2** as the parent complex, using MeLi (as an Et_2_O solution) or LiCH_2_SiMe_3_, in both cases with the elimination of LiCl. Both reactions were essentially instantaneous at room temperature. In both methyl complexes—^Me_2_^SB(^^*t*^Bu_2_^ArO,I*)TiMe_2_ (**10**) and ^Me_2_^SB(^^*t*^Bu,Me^ArO,I*)TiMe_2_ (**16**)—two TiC*H*_3_ resonances are found at δ 0.16 and 0.87 ppm in the ^1^H NMR spectra. The neosilyl complexes—^Me_2_^SB(^^*t*^Bu_2_^ArO,I*)Ti(CH_2_SiMe_3_)_2_ (**11**) and ^Me_2_^SB(^^*t*^Bu,Me^ArO,I*)Ti(CH_2_SiMe_3_)_2_ (**17**)—each display two CH_2_Si*Me*_3_ resonances between δ 0.12 and 0.17 ppm. In addition, the C*H*_2_SiMe_3_ resonances appear as two pairs of diastereotopic doublets at δ 1.91, 0.97, 0.94, and −0.89 ppm with ^2^*J*_H–H_ geminal coupling constants of 11 Hz. In the solid-state crystal structures, while **10** adopts the B conformation (TA′ = 33.8(1)°), **11** displays an intermediate torsion angle toward A-type geometry (TA′ = 65.4(2)°). This demonstrates that methylation has little structural effect compared to the parent dichloride complex, **2**, which is significant as methylation is involved in the initiation step of catalytic olefin polymerization. Attempts to synthesize benzyl (CH_2_Ph, Bn) complexes derived from both **1** and **2** were unsuccessful using KBn. However, a clean product was obtained between **2** and the Grignard reagent BnMgCl in a mixed benzene/THF solvent system. The bright orange product, ^Me_2_^SB(^^*t*^Bu_2_^ArO,I*)TiBn_2_ (**12**), was then extracted in toluene, using 1,4-dioxane to aid precipitation of the byproduct MgCl_2_. In the ^1^H NMR spectrum of **12**, the benzyl aromatic protons are seen as three multiplets at δ 6.86, 6.93, and 7.04 ppm, and the diastereotopic C*H*_2_Ph protons are two pairs of doublets at δ 1.31, 2.12, 2.66, and 2.74 ppm, with a ^2^*J*_H–H_ geminal coupling constant of 11 Hz.

Amide, alkoxide, and aryloxide complexes were formed from the reaction between **2** and two equivalents of either LiNMe_2_, NaOEt, or KODipp (Dipp = 2,6-di-*iso*-propylphenyl), eliminating the corresponding alkali metal chloride salt. These reactions occurred more slowly than the halide and alkyl exchanges: complete substitution was observed with the amide after 42 h at 80 °C, the alkoxide after 137 h at 60–80 °C, and the aryloxide after 24 h at 60 °C. In all cases, the formation of the monosubstituted intermediates ^Me_2_^SB(^^*t*^Bu_2_^ArO,I*)Ti(Cl)X (X = NMe_2_, OEt, ODipp) could be observed during the course of reaction by ^1^H NMR spectroscopy (Figure S66).

For the fully substituted amide complex, ^Me_2_^SB(^^*t*^Bu_2_^ArO,I*)Ti(NMe_2_)_2_ (**13**), the N*Me*_2_ protons have resonances at δ 2.60 and 3.00 ppm in the ^1^H NMR spectrum. For the alkoxide complex, ^Me_2_^SB(^^*t*^Bu_2_^ArO,I*)Ti(OEt)_2_ (**14**), the OCH_2_C*H*_3_ protons resonate as triplets at δ 1.01 and 1.18 ppm (^3^*J*_H–H_ = 6.9 Hz), and the OC*H*_2_CH_3_ protons are seen as a multiplet at δ 3.88 ppm and a quartet at δ 4.42 ppm, all shifted slightly to higher frequency compared to the monosubstituted intermediate. In the ^1^H NMR spectrum of the aryloxide complex, ^Me_2_^SB(^^*t*^Bu_2_^ArO,I*)Ti(ODipp)_2_ (**15**), the ArC*H*Me_2_ resonances are binomial septets at δ 3.14 and 3.52 ppm (^3^*J*_H–H_ ≈ 6.8 ppm), the ArCHMe_2_ protons exist as doublets at δ 0.89, 0.98, 1.16, and 1.20 ppm (^3^*J*_H–H_ ≈ 6.7 ppm). This suggests that, while the ODipp leaving groups are magnetically inequivalent to each other, rotation about the O–Dipp bond is fast on the NMR spectroscopic timescale, leading to a time-averaged signal from the two *iso*-propyl groups on each Dipp. The Dipp aromatic protons are seen as multiplets between δ 6.81 and 7.12 ppm. In all cases, therefore, the two ancillary ligands are inequivalent and do not exchange on the NMR timescale. Both **13** and **15** have large TA′ parameters in the solid-state structures (78.5(1) and 62.5(1)° respectively), **13** classified as A-type geometry, and **15** somewhat intermediate between the A- and B-type conformations.

### Exploration of Reactive PHENI* Cationic Intermediates

The catalytically active species in group four metallocene olefin polymerization is believed to be a cationic monoalkyl complex of the form [[L]MMe]^+^, formed by the reaction of the precatalyst with a suitable activator. Activators such as [Ph_3_C][BAr^F^_4_] (Ar^F^ = C_6_F_5_) or [PhNHMe_2_][BAr^F^_4_] have been demonstrated to abstract an alkyl group from the metal center, while MAO is additionally able to alkylate the precatalyst prior to abstraction.^54,55^

To probe this reaction, and the identity and stability of the reactive intermediate in olefin polymerization, dichloride complex **2** and dimethyl complex **10** were reacted separately with 1–10 molar equivalents of MAO and monitored by ^1^H NMR spectroscopy in a range of solvents (benzene-*d*_6_, bromobenzene-*d*_5_, and dichloromethane-*d*_2_; Figures S81–S86). In all cases, the formation of a single PHENI* species is observed consistent with *C*_1_ symmetry, with a single resonance corresponding to a TiC*H*_3_ environment in a cationic complex. For **2**/MAO, this ^1^H NMR resonance is seen at δ 0.17 ppm, correlating to a ^13^C shift of δ 66 ppm in the HSQC NMR spectrum (dichloromethane-*d*_2_); for **10**/MAO, the resonance is at δ 0.46 ppm, correlated to a ^13^C shift of δ 55 ppm. Notably, the species formed from **2** and **10** gave resonances with different absolute chemical shifts, which suggests non-negligible interaction of the counterion (either [MAO–Cl]^−^ or [MAO–Me]^−^) with the cationic metal center. This may have implications for catalysis, where ion-pairing of this sort is commonly found to affect activity.^56^ Exposing the NMR tubes to an atmosphere of ethylene (1 bar) resulted in the instant formation of a polyethylene precipitate, demonstrating that both activated PHENI* complexes are effective catalysts for olefinic polymerization.

The PHENI* dimethyl complex **10** was also reacted with [Ph_3_C][BAr^F^_4_] (TB) and [PhNHMe_2_][BAr^F^_4_] and monitored by ^1^H NMR spectroscopy in a range of solvents (benzene-*d*_6_, bromobenzene-*d*_5_, dichloromethane-*d*_2_, pyridine-*d*_5_, and acetonitrile-*d*_3_; Figures S87–S91). Reactions between **10** and [Ph_3_C][BAr^F^_4_] resulted in the instant formation of a dark red solution. The resulting salt was virtually insoluble in any of the noncoordinating solvents studied. The formation of Ph_3_CMe could be clearly identified by ^1^H NMR spectroscopy, and the formation of a single BAr^F^ salt was confirmed by ^11^B and ^19^F NMR spectroscopy. The resonances corresponding to the PHENI* ligand were not assignable due to the poor solubility or possibly indicating a mixture of products or ligand decomposition. A resonance at δ 0.21 ppm (dichloromethane-*d*_2_) is consistent with the [TiC*H*_3_]^+^ resonance observed from the reactions with MAO. Subsequent exposure of the NMR tubes to ethylene (1 bar) led to the instant formation of a polyethylene precipitate. It was postulated that a coordinating solvent may stabilize and solubilize the ionic salt product; however, reactions between **10** and [Ph_3_C][BAr^F^_4_] in pyridine-*d*_5_ or acetonitrile-*d*_3_ led to a mixture of products. A broad resonance at δ 0.62 ppm (acetonitrile-*d*_3_) is tentatively assigned to the [TiC*H*_3_]^+^ resonance, but catalyst decomposition is seen to occur in these coordinating solvents.

The reaction between **10** and [PhNHMe_2_][BAr^F^_4_] was studied in benzene-*d*_6_, bromobenzene-*d*_5_, and dichloromethane-*d*_2_. A mixture of products was obtained, indicating poor solution-phase stability of the cationic titanium complex. However, the presence of CH_4_ in the ^1^H NMR spectrum (δ 0.16 ppm (benzene-*d*_6_), 0.15 ppm (bromobenzene-*d*_5_), 0.21 ppm (dichloromethane-*d*_2_)) is suggestive that the anilinium borate is able to abstract a methyl group from **10**. The poorly coordinating BAr^F^_4_ anion is expected to reduce the ion pairing effect and increase catalytic activity. However, it also results in a destabilized cation relative to the more tightly bound [[L]TiMe][Cl–MAO] pair.

### Homogeneous Polymerization Reactivity

**Figure 4:**
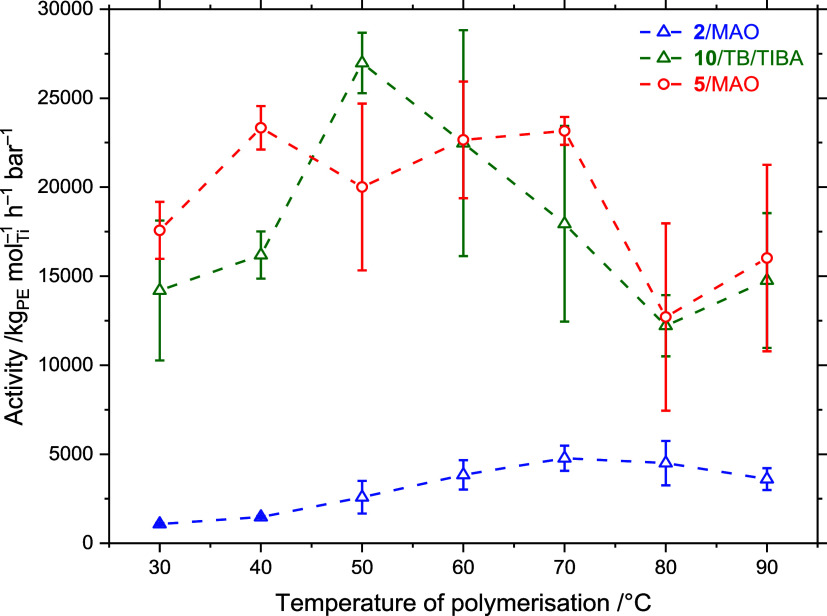
Solution-phase mean polymerization activity as a function of temperature of polymerization using the catalysts ^Me_2_^SB(^^*t*^Bu_2_^ArO,I*)TiCl_2_ (**2**), ^Me_2_^SB(^^*t*^Bu_2_^ArO,I*)TiMe_2_ (**10**), and ^Me_2_^SB(^^Cumyl^_2_^ArO,I*)TiCl_2_ (**5**). Polymerization conditions: 715 nmol complex, MAO ([Al_MAO_]_0_/[Ti]_0_ = 1000) or TB/TIBA ([TB]_0_/[TIBA]_0_/[Ti]_0_ = 1:200:1), 2 bar ethylene, 50 mL hexanes, and 5 min or until stirring ceased. Error bars shown at one standard deviation.

The PHENI* complexes ^Me_2_^SB(^^*t*^Bu_2_^ArO,I*)TiCl_2_ (**2**), and ^Me_2_^SB(^^Cumyl^_2_^ArO,I*)TiCl_2_ (**5**) were initially screened for polymerization activity in homogeneous solution-phase reactions, activated by MAO ([Al_MAO_]_0_/[Ti]_0_ = 1000), across a temperature of polymerization (*T*_p_) range of 30 ≤ *T*_p_ ≤ 90 °C (Figure 4; Table S4). These complexes were selected to probe the effect of ligand sterics on polymerization activity, with the bulky cumyl groups (**5**) compared to the *tert*-butyl (**2**). Polymerization runs were performed in hexanes for 5 min or until reactor fouling resulted in the cessation of stirring. Both complexes show high activities toward ethylene polymerization, with **2**/MAO up to 4220 kg_PE_ mol_Ti_^–1^ h^–1^ bar^–1^ (*T*_p_ = 70 °C) and **5**/MAO up to a remarkable 23 300 kg_PE_ mol_Ti_^–1^ h^–1^ bar^–1^ (*T*_p_ = 40 °C). Nabika *et al.* have claimed that bulkier ortho substituents on PHENICS complexes result in increased polymerization activities,^17^ and this also appears to be the case for PHENI* catalysts. As is commonly seen for solution-phase polymerizations, the polymer formed with a highly aggregated morphology, which caused reactor fouling.^27,57^ Reactor fouling occurred within 2 min for **5**/MAO due to rapid polymer formation, and runs were stopped when the mechanical stirring became hindered. This contributes to the extremely high reported activities and also results in reduced experimental reproducibility. The activity of **5**/MAO is greater than that reported for unbridged indenyl zirconocenes ((^2,4,6-^Me^_3_^Ind)_2_ZrCl_2_: 3890 kg_PE_ mol_Zr_^–1^ h^–1^ bar^–1^ (*T*_p_ = 50 °C, 1 bar)),^58^ constrained geometry complexes (^Me_2_^SB(^^*t*^Bu^N,Cp*)TiMe_2_: 114 kg_PE_ mol_Ti_^–1^ h^–1^ bar^–1^ (*T*_p_ = 25 °C, 1 bar)),^59^ and PHENICS complexes (^Me_2_^SB(^^*t*^Bu,Me^ArO,Cp*)TiCl_2_: 10 267 kg_PE_ mol_Ti_^–1^ h^–1^ bar^–1^ (*T*_p_ = 40 °C, 6 bar)).^14^ Activity is comparable to that of *rac*-(SBI*)ZrCl_2_ (22 622 kg_PE_ mol_Zr_^–1^ h^–1^ bar^–1^ (*T*_p_ = 60 °C, 2 bar),^22^ and less than that of *rac*-(EBI*)ZrCl_2_ (61 800 kg_PE_ mol_Zr_^–1^ h^–1^ bar^–1^ (*T*_p_ = 70 °C, 2 bar)).^60^

Solution-phase polymerizations were also performed using complex **10** and the stoichiometric activator trityl borate (TB; [Ph_3_C][BAr^F^_4_]; Ar^F^ = C_6_F_5_). The reactive cationic intermediates arising from **2**/MAO and **10**/TB ought to be identical species ([^Me_2_^SB(^^*t*^Bu_2_^ArO,I*)TiMe]^+^), with this experiment probing the effects of activation and counteranion. Activators bearing weakly coordinating anions such as BAr^F^_4_^–^, CN(BAr^F^_3_)_2_^–^, C_6_F_4_-1,2-[BAr^F^_2_]_2_(μ-OAr^F^)^−^, or tetrakis[3,5-bis(pentafluorosulfanyl)phenyl]borate have been shown to result in extremely high polymerization activities.^61−65^ Furthermore, anion engineering has been identified as an important emerging method of modulating polymer properties.^66^ Al^*i*^Bu_3_ (TIBA) was included to act as a scavenger for protic impurities. Runs with **10**/TB/TIBA ([Ti]_0_/[TB]_0_/[Al]_0_ = 1:1:200) were performed across a temperature range of 30 ≤ *T*_p_ ≤ 90 °C. Polymerization activities were much higher than with **2**/MAO, up to 27 000 kg_PE_ mol_Ti_^–1^ h^–1^ bar^–1^ (*T*_p_ = 50 °C). As with **5**/MAO, reactor fouling interfered with the mechanical stirring, and runs were terminated after 26–190 s. The temperature profile of **10**/TB/TIBA is broadly similar to that of **5**/MAO, suggestive of a common rate-limiting process, likely resulting from mass-transport and morphological limitations.

The polymers resulting from solution-phase polymerizations were analyzed by differential scanning calorimetry (DSC) and gel-permeation chromatography (GPC). The polymers displayed melting transition temperatures at 141–143 °C and crystallinities of 56–66%, consistent with UHMWPE.^67^

Both **2**/MAO and **5**/MAO produced high- or ultrahigh-molecular-weight polyethylene, with **5**/MAO consistently greater than **2**/MAO (Figure S118). At 30 °C, **5**/MAO produced UHMWPE with a *M*_w_ of 1.7 MDa, while **2**/MAO produced PE with *M*_w_ of 690 kDa. This is greater than the *M*_w_ of polyethylene produced by reported PHENICS complexes (^Me_2_^SB(^^*t*^Bu,Me^ArO,Cp*)TiCl_2_: 635 kDa (*T*_p_ = 40 °C, 6 bar)), though in that case, the presence of 1-hexene is expected to lower the molecular weight compared to homopolymerization.^14^ Both complexes produced polymers with decreasing *M*_w_ at increasing temperatures, resulting from an increased rate of chain termination processes relative to propagation.^68^ By contrast, **10**/TB/TIBA produced polymers with ultrahigh molecular weights, up to 2.6 MDa, and largely independent of polymerization temperature, though with a large variance resulting from irregular reaction times. Increased molecular weights with a borate activator are expected from a reduction in chain transfer to aluminum.

### Heterogeneous Polymerization Reactivity; Effect of the PHENI* Ligand

Group four PHENI* complexes **1–15** were immobilized on third-generation sMAO in a [Al_sMAO_]_0_/[M]_0_ (M = Ti, Zr, Hf) ratio of 200:1 by standard procedures to afford **1**_**sMAO**_**–15**_**sMAO**_ as pale orange to brown solids^22^ and used for the polymerization of ethylene (Tables 3, S4 and S5). The ethylene polymerization performance of **1**_**sMAO**_, **2**_**sMAO**_, **5**_**sMAO**_, **8**_**sMAO**_, and **9**_**sMAO**_ have been previously described.^27^ It has been noted that the PHENI* catalyst **1**_**sMAO**_ is up to 22 times more active than the Ind-PHENICS analogue **A**_**sMAO**_. Moreover, while **2**_**sMAO**_ is more active than **1**_**sMAO**_, further increasing the ligand bulk results in decreased slurry-phase activity for **5**_**sMAO**_, contrary to the observed solution-phase trend.

**Table 3:** Selected Ethylene Polymerization Results for sMAO-Supported PHENI* Catalysts^a^

catalyst	activity/kg_PE_ mol_M_^–^^1^ h^–^^1^ bar^–^^1^	polymer yield/g	*M*_w_/kDa	*D̵*	*T*_m_/°C	*a* (%)
**1**_**sMAO**_/TIBA	3230 ± 150	2.277	2088	3.0	133.7	67.9
**2**_**sMAO**_/TIBA	3720 ± 110	2.629	2088	5.2	133.4	77.7
**3**_**sMAO**_/TIBA	340 ± 10	0.229	120.0/1303	3.8/3.4	135.5	95.0
**4**_**sMAO**_/TIBA	<30	trace	n.d.	n.d.	121.0	1.7^b^
**5**_**sMAO**_/TIBA	1980 ± 40	1.353	1777	3.5	133.9	71.3
**6**_**sMAO**_/TIBA	3900 ± 20	2.688	2337	3.1	136.4	64.4
**7**_**sMAO**_/TIBA	3710 ± 540	2.518	1366	3.1	136.1	89.0
**8**_**sMAO**_/TIBA	3130 ± 30	2.204	2438	3.0	135.3	83.0
**9**_**sMAO**_/TIBA	2490 ± 260	1.671	2696	2.6	134.5	75.7
**10**_**sMAO**_/TIBA	2820 ± 290	1.880	2174	3.0	134.6	70.1
**11**_**sMAO**_/TIBA	2590 ± 50	1.716	2114	1.7	135.9	64.3
**12**_**sMAO**_/TIBA	1870 ± 110	1.239	2274	1.9	136.3	72.8
**13**_**sMAO**_/TIBA	1870 ± 160	1.251	2523	2.3	134.3	67.1
**14**_**sMAO**_/TIBA	1370 ± 50	0.906	2609	2.4	134.9	67.2
**15**_**sMAO**_/TIBA	760 ± 40	0.505	2525	2.2	135.3	57.5
**A**_**sMAO**_/TIBA	140 ± 1	0.096	1092	20.9	132.4	78.0

a Data for all polymerizations may be found in Tables S4 and S5. Activity is reported as mean ± standard deviation. Weight-average molecular weight determined by GPC; thermal characterization determined by DSC (20 K min^–1^, second heat). Polymerization conditions: 10 mg solid catalysts, 150 mg TIBA, 2 bar ethylene, 50 mL hexanes, 30 min, and *T*_p_ = 60 °C.

b The low crystallinity of PE produced by **4**_**sMAO**_/TIBA is attributed to the large amount of catalyst residue present.

In addition to the typical kinetic and thermodynamic advantages of permethylation, increased stability, and hindered TIBA coordination, it is proposed that the large increase in activity of PHENI* complexes compared to Ind-PHENICS may in part be related to the conformational geometry of the catalyst. As defined by the torsion angle TA′, **A** has a value of 71.6°, and **1**, **2**, and **5** are in the range 32–43° and approximately 22 times more active in polymerization. In the B-conformation, the I* system is oriented away from the coordination sites at the metal, thus increasing accessibility and reducing the energy barrier for coordination–insertion. While methylation did not significantly change the TA′ parameter in **10** compared to the dichloride precatalyst, **2**, it remains unclear how the dynamic *in operando* ligand geometry is affected by the formation of the cationic active species or by immobilization at the sMAO surface. The observed rapid interconversion in solution is consistent with a hemilabile PHENI* ligand, which may also reduce the energy barrier to monomer insertion. DFT calculations suggest that the B geometry is thermodynamically preferred in both the cationic active species and the ethylene π complex (Table S3).

### Effect of the Group Four Metal

The zirconium complex **3**_**sMAO**_/TIBA showed moderate polymerization activity, up to 353 kg_PE_ mol_Zr_^–1^ h^–1^ bar^–1^ at *T*_p_ = 50 °C. Polymerization with the hafnium complex, **4**_**sMAO**_/TIBA, resulted in only trace quantities of polymer being isolated (<20 mg) with activities of <30 kg_PE_ mol_Hf_^–1^ h^–1^ bar^–1^. The presence of minor impurities in the NMR spectrum of bulk samples of **4** may be expected to have a disproportionate effect on the catalytic performance, but single crystals of **4** were then used in the immobilization to mitigate this as far as possible given the small sample mass.

A similar trend was reported for group four PHENICS complexes by Hanaoka *et al.* with ^Me_2_^SB(^^*t*^Bu,Me^ArO,Cp*)TiCl_2_ and ^Me_2_^SB(^^*t*^Bu,Me^ArO,Cp*)ZrCl_2_ having activities of 10 300 and 530 kg mol_M_^–1^ h^–1^ bar^–1^, respectively.^14^ Likewise, Senda *et al.* report a series of fluorenyl PHENICS complexes with activities of 2800, 1030, and 200 kg mol_M_^–1^ h^–1^ bar^–1^, respectively, for ^Me_2_^SB(^^*t*^Bu,Me^ArO,^^*t*^Bu_2_^Flu)TiCl_2_, ^Me_2_^SB(^^*t*^Bu,Me^ArO,^^*t*^Bu_2_^Flu)ZrCl_2_, and ^Me_2_^SB(^^*t*^Bu,Me^ArO,^^*t*^Bu_2_^Flu)HfCl_2_.^18^ However, in both cases, polymerization data for PHENICS complexes were reported only for ethylene/1-hexene copolymerization. DFT studies *in silico* have found that the activation barrier for ethylene insertion is lower for Ti(IV)-constrained geometry complexes, modeled as [^H_2_^SB(^H^N,Cp)MMe]^+^ (M = Ti, Zr, Hf), than either Zr(IV) or Hf(IV).^69^ Similar results have also been found for metallocenic systems, Cp_2_MMe_2_/B(C_6_F_5_)_3_, with titanocenes having lower coordination and insertion barriers than zirconocenes or hafnocenes.^70^ These trends are somewhat controversial, however, with claims published that both titanium-^71,72^ and zirconium-based^73,74^ complexes are more active for ethylene polymerization, and counterclaims that there is little difference.^49,75^ The divergence of experimental results from computed insertion energy barriers highlights the role of nontrivial reaction mechanisms and kinetics, with polymerization activity determined by the subtle interplay of many steric, electronic, kinetic, and thermodynamic factors. In the current work, it is noted that the less active catalysts, **3** and **4**, both have A-type solid-state conformations, while the more active **2** is B-type.

### Effect of the *ansa* Bridging SiMeR″ Group

PHENI* complexes bearing varied substituents at the silane *ansa* bridge displayed largely similar slurry-phase polymerization behavior. All three complexes, the SiMePh mixed-bridge complex, **7**_**sMAO**_/TIBA (3710 kg mol_Ti_^–1^ h^–1^ bar^–1^ at *T*_p_ = 60 °C), the SiMe_2_ bridge complex **2**_**sMAO**_/TIBA (3720 kg mol_Ti_^–1^ h^–1^ bar^–1^ at *T*_p_ = 60 °C), and the SiMe^*n*^Pr complex, **6**_**sMAO**_/TIBA (3900 kg mol_Ti_^–1^ h^–1^ bar^–1^ at *T*_p_ = 60 °C), all displayed similar activities. The slightly higher activity of **6**_**sMAO**_/TIBA is despite **6** having an A-type solid-state conformation, highlighting the complex nature of structure–activity relationships in olefin polymerization chemistry.

### Effect of the Ancillary X Ligand

**Figure 5:**
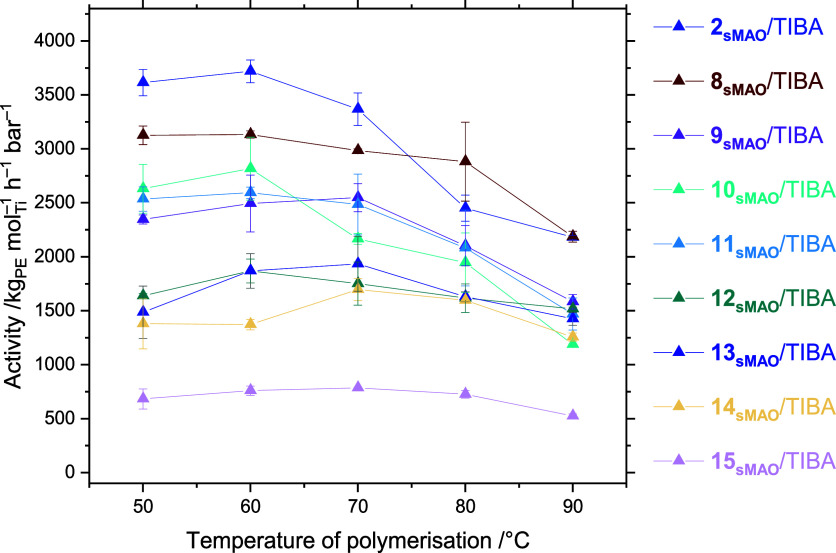
Slurry-phase mean ethylene polymerization activity as a function of temperature for sMAO-supported ^Me_2_^SB(^^*t*^Bu_2_^ArO,I*)TiCl_2_ (**2**), ^Me_2_^SB(^^*t*^Bu_2_^ArO,I*)TiBr_2_ (**8**), ^Me_2_^SB(^^*t*^Bu_2_^ArO,I*)TiI_2_ (**9**), ^Me_2_^SB(^^*t*^Bu_2_^ArO,I*)TiMe_2_ (**10**), ^Me_2_^SB(^^*t*^Bu_2_^ArO,I*)Ti(CH_2_SiMe_3_)_2_ (**11**), ^Me_2_^SB(^^*t*^Bu_2_^ArO,I*)TiBn_2_ (**12**), ^Me_2_^SB(^^*t*^Bu_2_^ArO,I*)Ti(NMe_2_)_2_ (**13**), ^Me_2_^SB(^^*t*^Bu_2_^ArO,I*)Ti(OEt)_2_ (**14**), ^Me_2_^SB(^^*t*^Bu_2_^ArO,I*)Ti(ODipp)_2_ (**15**). Polymerization conditions: 10 mg solid catalyst, 150 mg TIBA, 2 bar ethylene, 50 mL hexanes, and 30 min. Error bars shown at one standard deviation.

All the complexes **8**_**sMAO**_**–15**_**sMAO**_ are theorized to form the same [L]TiMe^+^ active species and are observed to tend toward mutually similar polymerization activities at high temperatures (Figure 5). A particular exception to this is the ODipp complex **15**, though a confounding factor in this case was incomplete immobilization resulting from the large ODipp ligands shown by a significantly colored supernatant even after heating at 80 °C for 3 h with stirring. Otherwise, the chloride and bromide complexes show activities of 2180–2190 kg_PE_ mol_Ti_^–1^ h^–1^ bar^–1^, and the complexes **9**_**sMAO**_**–14**_**sMAO**_ show activities between 1190 and 1580 kg_PE_ mol_Ti_^–1^ h^–1^ bar^–1^ at a polymerization temperature of 90 °C. At lower polymerization temperatures, much more substantial deviations in polymerization activities are observed, with the catalysts **2**_**sMAO**_, **8**_**sMAO**_**–14**_**sMAO**_ having activities of 1380–3610 kg_PE_ mol_Ti_^–1^ h^–1^ bar^–1^.

It was anticipated that alkylated complexes **10**_**sMAO**_**–12**_**sMAO**_ would have increased activities relative to halide precatalysts, since halide abstraction is the first step of the initiation pathway; however, the general trend halide ≳ alkyl ≳ amide ≳ alkoxide ≳ aryloxide emerges. This indicates that activation is relatively facile, and a mixture of steric, electronic, and stability effects are likely at play: since charge separation is disfavored in nonpolar solvents, ion pairing between [[L]TiMe]^+^ and [sMAO–X]^−^ in a secondary coordination sphere is the most probable justification for the influence of the leaving group. Metallocenium ion pairing has been shown to be a significant factor in ethylene polymerization, with the Lewis acidity and free energy of ion pair separation correlating with catalytic activity.^76^ Punzalan *et al.* have recently shown *in silico* that the complex interactions of ion pairing, catalyst conformation, and polymer chain conformation in titanium CGC systems influence ethylene insertion and β-elimination kinetics.^77^ Alkylated permethylindenyl metallocenes, supported on sMAO, have been shown previously to have higher ethylene polymerization activities than the parent halide complexes.^24^ However, in the current work, the methyl (**10**) and neosilyl (**11**) complexes demonstrated activities comparable to iodide (**9**) and less than both bromide (**8**) and chloride (**2**), with benzyl (**12**) having still lower activities. These results suggest that precatalyst activation is comparatively facile, with steric, electronic, conformational, and secondary coordination effects dominating the propagation kinetics. Ionic reassociation has been shown to lead to the formation of long-lived resting states.^78^ Superimposed on the ion pair effect is the influence of the ancillary ligands on PHENI* conformation. In the slurry-phase it has been shown that the position of the A–B conformational equilibrium may be a relevant parameter when comparing Ind and I* titanium dichloride complexes. The ancillary ligands alter the conformation of the solid-state structures, and this may be expected to have an observable effect on catalysis if the initial conformational preference is preserved upon immobilization.

### Polyolefin Characterization

All titanium catalysts produced polyethylenes with melting transitions between 133 and 136 °C, and crystallinities of α = 58–83%, consistent with the formation of linear HDPE (Figure S119 and Table S6).

The molecular weight of PE produced by the PHENI* complex **1**_**sMAO**_/TIBA was substantially greater than that by **A**_**sMAO**_/TIBA as well having a much narrower dispersity (Figure S117). The bimodal distribution observed for **A**_**sMAO**_/TIBA is indicative of a departure from single-site catalytic behavior. The molecular weight of PE produced by **1**_**sMAO**_/TIBA and **2**_**sMAO**_/TIBA is similar and unimodal, suggesting that substitution at the para position of the phenoxy group has little bearing on polymerization kinetics. Cumyl substitution at the ortho position resulted in reduced molecular weights, with the additional steric bulk seemingly unable to reduce chain transfer processes. Substitution at the silicon bridge was found to have a more significant effect on the polymer molecular weight, with the Me^*n*^PrSi bridge (**6**_**sMAO**_/TIBA; 2.3 MDa, *T*_p_ = 60 °C) producing slightly higher *M*_w_ polymer than the Me_2_Si bridge (**2**_**sMAO**_/TIBA; 2.1 MDa, *T*_p_ = 60 °C), and the MePhSi bridge (**7**_**sMAO**_/TIBA; 1.4 MDa, *T*_p_ = 60 °C) significantly lower. This is evidence that the substituents at the silicon *ansa* bridge influence the kinetics of the polymerization mechanism, altering the relative rates of termination and propagation.

The zirconium catalyst **3**_**sMAO**_/TIBA produces polyethylene with a bimodal molecular weight distribution (MWD), with large aggregate dispersities in the range 41.0–92.3 (Figure S111). Peak deconvolution reveals that approximately 80% of the polymer mass is composed of low-molecular-weight PE (*M*_w_ ≤ 25 kDa) with a moderate *D̵* of 3.4–7.9. The remainder is composed of UHMWPE (*M*_w_ = 1.2–2.1 MDa) with narrow MWDs, having dispersities in the range 1.9–3.4. This is a clear departure from single-site catalysis, with at least two reaction modes active, and may in part be due the increased chain transfer to aluminum possible with the larger group four metals. No evidence was found for a bimodal copolymer MWD for zirconium PHENICS complexes.^14,18^ The melting temperature of 136 °C (α = 95%) is consistent with the formation of a single thermal phase of HDPE. By modeling group four metallocenes *in silico*, Martínez-Araya *et al.* calculated, on the basis of energetic barriers, that titanocenes were expected to produce higher-molecular-weight polymers than either zirconocenes or hafnocenes.^79^ Very few complexes of zirconium or hafnium have been reported able to produce UHMWPE owing to their propensity for β-hydrogen transfer.^80,81^ For the reported PHENI* complexes, it is clear that titanium gives not only dramatically higher polymerization activity than its heavier congeners, but also better controlled single-site reactivity. The quantity of PE isolated from polymerization using the hafnium catalyst **16**_**sMAO**_/TIBA was insufficient for GPC characterization, though the identity of the sample as HDPE was confirmed by DSC analysis, having a weak melting transition at 121 °C, slightly lower than expected probably due to relatively high amounts of catalyst residue in the polymer resin.

**Figure 6:**
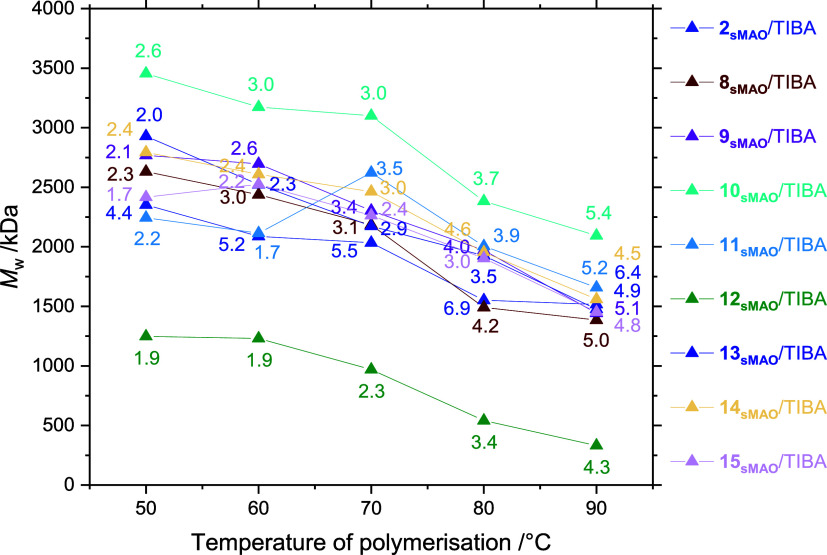
Polyethylene molecular weight (*M*_w_; *D̵* annotated) as a function of temperature synthesized by sMAO-supported ^Me_2_^SB(^^*t*^Bu_2_^ArO,I*)TiCl_2_ (**2**), ^Me_2_^SB(^^*t*^Bu_2_^ArO,I*)TiBr_2_ (**8**), ^Me_2_^SB(^^*t*^Bu_2_^ArO,I*)TiI_2_ (**9**), ^Me_2_^SB(^^*t*^Bu_2_^ArO,I*)TiMe_2_ (**10**), ^Me_2_^SB(^^*t*^Bu_2_^ArO,I*)Ti(CH_2_SiMe_3_)_2_ (**11**), ^Me_2_^SB(^^*t*^Bu_2_^ArO,I*)TiBn_2_ (**12**), ^Me_2_^SB(^^*t*^Bu_2_^ArO,I*)Ti(NMe_2_)_2_ (**13**), ^Me_2_^SB(^^*t*^Bu_2_^ArO,I*)Ti(OEt)_2_ (**14**), ^Me_2_^SB(^^*t*^Bu_2_^ArO,I*)Ti(ODipp)_2_ (**15**). Polymerization conditions: 10 mg solid catalyst, 150 mg TIBA, 2 bar ethylene, 50 mL hexanes, and 30 min.

For the titanium catalysts with varied ancillary ligands (**2**_**sMAO**_, **8**_**sMAO**_**–15**_**sMAO**_), with the exception of the dimethyl complex (**10**) and the dibenzyl complex (**12**), PE was produced within a narrow molecular-weight envelope, spanning 2.2–2.9 MDa at *T*_p_ = 50 °C, and 1.4–1.7 MDa at *T*_p_ = 90 °C (Figure 6). This is to be expected from the formation of a common propagating cationic active species. Surprisingly, **10**_**sMAO**_/TIBA produced PE with notably higher *M*_w_ (3.5 MDa at *T*_p_ = 50 °C) and **12**_**sMAO**_/TIBA with notably lower *M*_w_ (1.2 MDa at *T*_p_ = 50 °C). In all cases, dispersities are relatively narrow in the range 1.9–4.4 at *T*_p_ = 50 °C, indicative of consistent single-site catalysis. A trend toward broader MWDs at higher polymerization temperatures is observed, with a dispersity range at *T*_p_ = 90 °C of 4.3–6.4, possibly due to partial thermal degradation of the catalyst, or increased ligand flexibility.

### High-Pressure High-Throughput Screening

**Figure 7:**
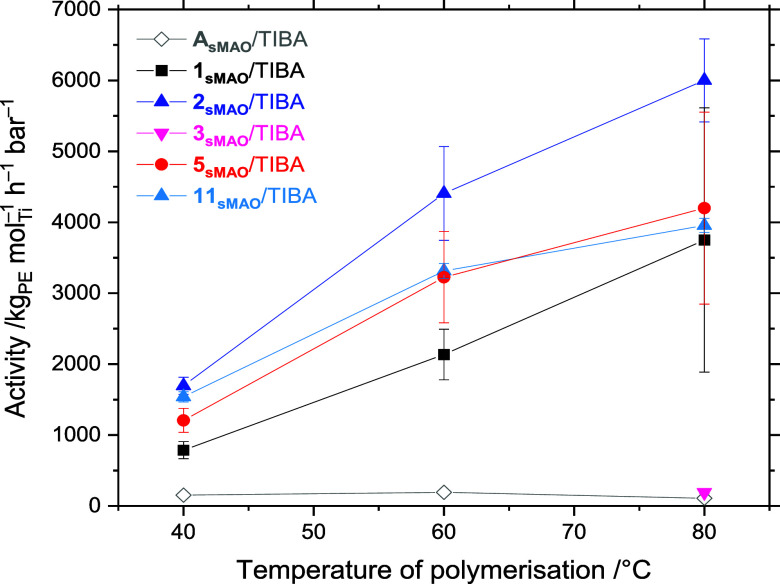
Slurry-phase mean polymerization activity as a function of polymerization temperature for sMAO-supported ^Me_2_^SB(^^*t*^Bu,Me^ArO,Ind)TiCl_2_ (**A**), ^Me_2_^SB(^^*t*^Bu,Me^ArO,I*)TiCl_2_ (**1**), ^Me_2_^SB(^^*t*^Bu_2_^ArO,I*)TiCl_2_ (**2**), ^Me_2_^SB(^^*t*^Bu_2_^ArO,I*)ZrCl_2_ (**3**), ^Me_2_^SB(^^Cumyl^_2_^ArO,I*)TiCl_2_ (**5**), and ^Me_2_^SB(^^*t*^Bu_2_^ArO,I*)Ti(CH_2_SiMe_3_)_2_ (**11**). Polymerization conditions: 0.05–0.80 mg solid catalyst, 10 μmol TIBA, 8.3 bar ethylene, 5 mL heptane, and either 60 min or until 120 psi ethylene uptake. Error bars shown at one standard deviation.

Parallelized high-throughput studies were performed at Xplore s.r.l. under 120 psi (8.3 bar) ethylene in 23 mL cells containing 5 mL of heptane, 10 μmol TIBA, and 0.05–0.80 mg of the precatalysts **1**_**sMAO**_**–3**_**sMAO**_, **5**_**sMAO**_, **11**_**sMAO**_, and **A**_**sMAO**_ across a temperature range of 40 ≤ *T*_p_ ≤ 80 °C.^82^ Contrary to the observed behavior at 2 bar, polymerization activity was greater at 80 °C than 60 °C (Figure 7). This suggests that the dominating factor in the previously observed high temperature activity decrease (*vide supra*) is the reduced solubility of ethylene, rather than catalyst thermal degradation. The ligand effects are fairly consistent across the two polymerization regimes, with **2**_**sMAO**_/TIBA displaying the highest activities at 8.3 bar, up to 6001 kg_PE_ mol_Ti_^–1^ h^–1^ bar^–1^ at 80 °C, more than double the efficiency reported at 2 bar pressure. The activity of **1**_**sMAO**_/TIBA at 80 °C was 3750 kg_PE_ mol_Ti_^–1^ h^–1^ bar^–1^, 34 times greater than for the analogous supported Ind–PHENICS complex, **A**_**sMAO**_/TIBA under the same conditions.

Kinetic profiles are stable up to a reaction time of 1 h with minimal catalyst deactivation observed (Figures S124–S127). In all cases, the polyethylene produced had significantly greater molecular weights, as measured by GPC, than when synthesized at 2 bar (Figure S129). At *T*_p_ = 60 °C, **A**_**sMAO**_ produced PE with a *M*_w_ of 2.3 MDa, double that recorded at 2 bar, and with a narrower, though still poorly controlled, dispersity of 9.2. The PHENI* catalysts all produce UHMWPE in a narrow range between 4.1 and 4.9 MDa, approximately double that recorded at 2 bar. Remarkable temperature invariance was displayed, while also being excellently controlled single-site catalysts, with dispersities in the range 1.8–2.2, close to the ideal Flory–Schulz value of 2.0 for controlled single-site catalysts.^83,84^ Furthermore, very little ligand dependence is observed with all four PHENI* catalysts producing similar PEs, which indicates a common limiting factor. It is anticipated that increased ethylene pressure leads to increased rates of chain propagation and therefore high polymer molecular weights for cases where the controlling process is not chain transfer to monomer.^85−87^

## Conclusions

Seventeen PHENI* complexes have been synthesized and characterized with a variety of phenoxide substituents, ancillary ligands, and group four metals. For the titanium dichloride complexes, it appears that the permethylindenyl complexes adopt a distorted conformation compared to the Ind-PHENICS in the solid state, which results in a more open active site. Conformation seems to depend on permethylation, the group four metal, and ancillary X ligands. The efficacy of catalysts based on group four PHENI* complexes toward the polymerization of ethylene has been demonstrated. In addition to exceptional homogeneous catalytic activities, up to 27 000 kg_PE_ mol_Ti_^–1^ h^–1^ bar^–1^, the complexes have been immobilized on sMAO, which offers excellent morphological control. Ultrahigh-molecular-weight polyethylene is produced, with *M*_w_ up to 3.5 MDa. The influence of catalyst structure on polymerization has been studied and steric, conformational, electronic, and coordinative influences have been discussed. Ion pairing and ligand geometry have been explored as parameters for determining polymerization activity. The conformation may exert an influence on catalytic performance, but against a background of solution-phase interconversion equilibria, the effects of immobilization and activation, and other parameters, the significance of conformation in this system remains unclear. At 8.3 bar, slurry-phase activities over 6000 kg_PE_ mol_Ti_^–1^ h^–1^ bar^–1^ are reported, with polymer molecular weight up to 4.9 MDa and narrow MWDs, consistent with ideal controlled single-site catalysis. Overall, this establishes the PHENI* catalysts as being among the most active complexes for the controlled synthesis of UHMWPE.

## Experimental Section

### General Procedures

Air- and moisture-sensitive compounds were manipulated under an inert atmosphere of nitrogen, using standard Schlenk line techniques^88^ on a dual manifold vacuum/nitrogen line or in an MBraun Labmaster 100 glovebox. Pentane, hexane, toluene, and benzene were dried using an MBraun SPS 800 solvent purification system, stored over a potassium mirror, and degassed under partial vacuum before use. Anhydrous dichloromethane was dried using an MBraun SPS 800 system, stored over preactivated 3 Å molecular sieves and degassed under partial vacuum before use. Tetrahydrofuran was distilled from sodium/benzophenone, stored over preactivated 3 Å molecular sieves and degassed under partial vacuum before use. Diethyl ether was distilled from NaK, stored over a potassium mirror, and degassed under partial vacuum before use. Acetone was used as received. 1,4-Dioxane was distilled from sodium, stored over preactivated 3 Å molecular sieves, and degassed under partial vacuum before use. Deuterated solvents were dried over potassium metal (benzene-*d*_6_, and toluene-*d*_8_) or CaH_2_ (chloroform-*d*, dichloromethane-*d*_2_, acetonitrile-*d*_3_, bromobenzene-*d*_5_, pyridine-*d*_5_, and tetrahydrofuran-*d*_8_) and refluxed under reduced pressure, distilled under static vacuum, freeze–pump–thaw degassed three times, and stored over preactivated 3 or 4 Å molecular sieves. NMR spectra were recorded on either a Bruker Avance III HD NanoBay NMR (9.4 T, 400.2 MHz), a Bruker Avance III NMR (11.75 T, 499.9 MHz), a Bruker Avance NMR (11.75 T, 500.3 MHz) with a ^13^C-detect cryoprobe, or a Bruker NEO 600 (14.1 T, 600.4 MHz) with a broadband helium cryoprobe. Solid-state NMR spectra were recorded by Dr Nicholas Rees (University of Oxford) on a Bruker Avance III HD NanoBay solid-state NMR spectrometer (9.4 T, 399.9 MHz) or by Dr Dinu Iuga (University of Warwick, UK High-Field Solid-State NMR Facility) on a Bruker WB US^2^ solid-state NMR spectrometer (20.0 T, 850.2 MHz). Samples were spun at the magic angle at spin rates of 10, 12, 20, or 60 kHz. ^13^C NMR spectra were referenced to adamantane, ^27^Al to aluminum nitrate, and ^29^Si to kaolinite. CHN elemental analyses were carried out in duplicate by Orla McCullough (London Metropolitan University). GPC was performed on a high-temperature gel permeation chromatograph with an IR5 infrared detector (GPC-IR5). Samples were prepared by dissolution in 1,2,4-trichlorobenzene (TCB) containing 300 ppm of 3,5-di-*tert*-butyl-4-hydroxytoluene (BHT) at 160 °C for 90 min and then filtered with a 10 μm SS filter before being passed through the GPC column. The samples were run under a flow rate of 0.5 mL min^–1^ using TCB containing 300 ppm of BHT as the mobile phase with 1 mg mL^–1^ BHT added as a flow rate marker. The GPC column and detector temperature were set at 145 and 160 °C respectively. Differential scanning calorimetry was performed on a PerkinElmer DSC 4000 System within a temperature range of 30–180 °C at a rate of 20 K min^–1^. Polymer samples were sealed in 100 μL of aluminum crucibles. An empty crucible was used as a reference, and the DSC was calibrated using indium and zinc.

(2,3,4,5,6,7-Hexamethyl-1*H*-inden-1-yl)lithium,^20,89^ TiCl_4_·2THF,^90^ and KODipp,^91^ were synthesized according to literature procedures or modified literature procedures. The syntheses of **1**, **2**, **5**, **8**, and **9** have been described elsewhere.^27^^*n*^BuLi (1.6 or 2.5 M in hexanes), MeLi (1.6 M in diethyl ether), PhCH_2_MgCl (2.0 M in THF), 4-methyl-2-*tert*-butylphenol, bromine, titanium tetrachloride, tri-*iso*-butylaluminum, 2,6-di-*iso*-propylphenol, potassium hydroxide, sodium hydroxide, sodium carbonate, 4-methyl-2-*tert*-butylphenol, sulfuric acid, triphenylmethylium tetrakis(pentafluorophenyl)borate, allyl bromide, and sodium ethoxide were purchased from Sigma-Aldrich and used as received. 2,3,4,5,6,7-Hexamethylindene (SCG Chemicals PLC), 6-bromo-2,4-di-*tert*-butylphenol (Alfa Aesar), and magnesium sulfate (Fisher Scientific) were all used as received. Me_3_SiI and Me_3_SiBr (Sigma-Aldrich) were stored over preactivated 3 Å molecular sieves and metallic copper. Et_3_N was dried over KOH, distilled under static vacuum, and freeze–pump–thaw degassed before use. 2,4-bis(α,α-dimethylbenzyl)phenol (Sigma-Aldrich) was recrystallized from hot ethanol before use. Me_2_SiCl_2_, Me(^*n*^Pr)SiCl_2_, and Me(Ph)SiCl_2_ (Sigma-Aldrich) were dried over preactivated 3 Å molecular sieves before use. Ethylene was supplied by BOC Ltd. and was passed through preactivated molecular sieves before use. MAO (Chemtura Corporation) and solid polymethylaluminoxane (sMAO, third generation; SCG Chemicals PLC) were supplied as slurries in toluene and were dried under vacuum before use.

### Preparation of ^Me,R″^SB(^R,R′^ArO,I*)Li_2_ (**L1–5**)

Allyl-protected 6-bromo-2,4-alkylphenols were dissolved in toluene in a Schlenk flask. To this was added dropwise at −70 °C 1.3 equiv of ^*n*^BuLi solution (1.6 M in hexanes), and the reaction was allowed to warm to −20 °C. This mixture was then dropwise added to a toluene solution of 3 equiv MeR′′SiCl_2_ at −20 °C. The reaction was allowed to warm to room temperature and stirred for 18 h. The solid byproducts were removed by filtration, and the volatiles removed under vacuum, leaving the chlorosilyl product, ^^Me^,^R''^,S^iCl,^R,R'^ArOAllyl, as a pale yellow to colorless oil. This was then combined with 1.1 equiv of (2,3,4,5,6,7-hexamethyl-1*H*-inden-1-yl)lithium (Ind^#^Li) and dissolved in THF at 0 °C. The reaction was allowed to warm to room temperature and stirred for 18 h, after which it was dried under vacuum; the product was extracted in pentane and the solvent removed under vacuum to yield the proligands (**P1–5**) ^Me,R″^SB(^R,R′^ArOAllyl,I*)H. The proligand was then redissolved in toluene. 3.5 equiv of Et_3_N was added, and the mixture cooled to −78 °C. 2.25 equiv of ^*n*^BuLi solution (1.6 M in hexanes) was added dropwise. The reaction was allowed to warm to room temperature and stirred for 3–18 h, during which the solution gradually darkens. Removal of the volatiles under vacuum yielded the ligands as dilithium salts, ^Me,R″^SB(^R,R′^ArO,I*)Li_2_ (**L1–5**), as highly air-sensitive bright orange powders in approximately quantitative yields. The crude products were used to synthesize PHENI* transition-metal complexes without further purification.

The proligands ^Me_2_^SB(^^*t*^Bu,Me^ArOAllyl,I*)H (**P1**), ^Me_2_^SB(^^*t*^Bu_2_^ArOAllyl,I*)H (**P2**), and ^Me_2_^SB(^^Cumyl^_2_^ArOAllyl,I*)H (**P3**) were also isolated as white solids prior to lithiation by washing the crude material with cold (−78 °C) hexanes. Further crops were isolated by recrystallization from hexanes (4 °C) and combined to give a yield of 40–75%.

#### **P1**

^1^H NMR (C_6_D_6_): δ 7.24 (d, 1H, Ar**H**), 7.03 (d, 1H, Ar**H**), 5.87 (ddt, 1H, OCH_2_C**H**CH_2_), 5.57 (app. dq, 1H, OCH_2_CHC**H**_2_), 5.16 (app. dq, 1H, OCH_2_CHC**H**_2_), 4.47 (ddt, 1H, OC**H**_2_CHCH_2_), 4.26 (ddt, 1H, OC**H**_2_CHCH_2_), 4.00 (s, 1H, I*H), 2.53 (s, 3H, I*Me), 2.21 (s, 6H, I*Me), 2.19 (s, 3H, I*Me), 2.17 (s, 6H, I*Me and Ar**Me**), 1.85 (s, 3H, I*Me), 1.49 (s, 9H, C**Me**_3_), 0.29 (s, 3H, Si**Me**), 0.07 (s, 3H, Si**Me**). ^13^C NMR (C_6_D_6_): δ 161.43 (1-**C**_6_H_2_), 142.06 (**Ar**), 141.96 (**Ar**), 141.65 (**Ar**), 139.38 (Ι*), 135.12 (3,5-**C**_6_H_2_), 133.80 (OCH_2_**C**HCH_2_), 132.54 (I*),132.48 (Ι*),132.14 (I*),131.74 (**Ar**), 130.01 (3,5-**C**_6_H_2_), 129.31 (**Ar**), 127.98 (**Ar**), 127.09 (**Ar**), 125.72 (I*),115.57 (OCH_2_CH**C**H_2_), 75.82 (O**C**H_2_CHCH_2_), 47.71 (I*Si), 35.03 (**C**Me_3_), 31.05 (C**Me**_3_), 20.81 (C_6_H_2_**Me**), 18.74 (I*Me), 16.10 (I*Me), 16.04 (I*Me), 16.03 (I*Me), 14.87 (I*Me), 14.57 (I*Me), 0.32 (Si**Me**), −3.12 (Si**Me**).

#### **P2**

^1^H NMR (C_6_D_6_): δ 7.59 (d, 1H, Ar**H**), 7.37 (d, 1H, Ar**H**), 5.87 (ddt, 1H, OCH_2_C**H**CH_2_), 5.56 (app. dq, 1H, OCH_2_CHC**H**_2_), 5.16 (app. dq, 1H, OCH_2_CHC**H**_2_), 4.47 (ddt, 1H, OC**H**_2_CHCH_2_), 4.26 (ddt, 1H, OC**H**_2_CHCH_2_), 3.98 (s, 1H, I*H), 2.53 (s, 3H, I*Me), 2.24 (s, 3H, I*Me), 2.21 (s, 3H, I*Me), 2.18 (s, 3H, I*Me), 2.14 (s, 3H, I*Me), 1.86 (s, 3H, I*Me), 1.53 (s, 9H, C**Me**_3_), 1.30 (s, 9H, C**Me**_3_), 0.28 (s, 3H, Si**Me**), 0.17 (s, 3H, Si**Me**). ^13^C NMR (C_6_D_6_): δ 161.70 (1-**C**_6_H_2_), 145.45 (2,4-**C**_6_H_2_), 142.51 (I*), 142.29 (I*), 141.58 (2,4-**C**_6_H_2_), 139.74 (I*), 134.21 (OCH_2_**C**HCH_2_), 132.96 (I*), 132.31 (6-**C**_6_H_2_), 132.17 (I*), 132.03 (3,5-**C**_6_H_2_), 129.67 (I*), 127.45 (I*), 126.51 (3,5-**C**_6_H_2_), 126.12 (I*), 115.91 (OCH_2_CH**C**H_2_), 76.00 (O**C**H_2_CHCH_2_), 48.45 (I*Si), 35.76 (**C**Me_3_), 34.65 (**C**Me_3_), 31.69 (C**Me**_3_), 31.46 (C**Me**_3_), 19.10 (I*Me), 16.51 (I*Me), 16.43 (I*Me), 16.39 (I*Me), 15.27 (I*Me), 15.05 (I*Me), 0.20 (Si**Me**), −2.32 (Si**Me**). ^29^Si NMR (C_6_D_6_): δ −0.72. Anal. Calcd for C_34_H_50_OSi: C, 81.21; H, 10.02. Found: C, 81.20; H, 9.64.

#### **P3**

^1^H NMR (C_6_D_6_): δ 7.63 (d, 1H, 3,5-C_6_**H**_2_), 7.35–7.28 (m, 5H, CMe_2_Ph**H** and 3,5-C_6_**H**_2_), 7.23–6.97 (m, 6H, CMe_2_Ph**H**), 5.49 (ddt, 1H, OCH_2_C**H**CH_2_), 5.15 (dd, 1H, OCH_2_CHC**H**_2_), 4.96 (dd, 1H, OCH_2_CHC**H**_2_), 3.83 (I*H), 3.49 (ddt, 1H, OC**H**_2_CHCH_2_), 3.26 (ddt, 1H, OC**H**_2_CHCH_2_), {2.50 (3H) 2.21 (3H) 2.17 (6H) 2.04 (3H) 1.78 (3H) 1.75 (3H) 1.71 (3H) 1.70 (3H) 1.67 (3H) (s, I*Me and C**Me**_2_Ph)}, 0.14 (s, 3H, Si**Me**), 0.02 (s, 3H, Si**Me**) ppm. ^13^C NMR (C_6_D_6_): δ 161.12 (O**Ar**), 151.80 (CMe_2_**Ph**), 151.05 (CMe_2_**Ph**), 145.28 (Ar), 142.45 (I*), 142.17 (I*), 142.04 (Ar), 139.57 (I*), 134.97 (OCH_2_**C**HCH_2_), 134.34 (O**Ar**), 132.93 (I*), 132.78 (I*), 132.10 (I*), 129.61 (I*), 128.38 (CMe_2_**Ph**), 128.35 (CMe_2_**Ph**), 128.16 (Ar), 128.12 (Ar), 127.97 (O**Ar**), 127.35 (Ar), 127.17 (Ar), 126.62 (Ar), 126.07 (I*), 126.04 (CMe_2_**Ph**), 125.62 (CMe_2_**Ph**), 114.95 (OCH_2_CH**C**H_2_), 74.34 (O**C**H_2_CHCH_2_), 48.41 (I*Si), 43.11 (**C**Me_2_Ph), 42.85 (**C**Me_2_Ph), 31.15 (C**Me**_2_Ph), 31.08 (C**Me**_2_Ph), 30.88 (C**Me**_2_Ph), 30.59 (C**Me**_2_Ph), 19.11 (I*Me), 16.54 (I*Me), 16.39 (I*Me), 16.36 (I*Me), 15.25 (I*Me), 15.08 (I*Me), −0.39 (Si**Me**), −2.29 (Si**Me**). Two additional aromatic ^13^C NMR resonances were expected but not detected. ^29^Si NMR (C_6_D_6_): δ −2.34. Anal. Calcd for C_44_H_54_OSi: C, 84.29; H, 8.68. Found: C, 83.77; H, 8.69.

#### Preparation of ^Me_2_^SB(^^*t*^Bu_2_^ArO,I*)ZrCl_2_ (**3**)

The ligand **L2** (7.1 g, 15 mmol) was prepared *in situ* and added dropwise to a toluene slurry of ZrCl_4_ (5.2 g, 22 mmol) and the reaction mixture was then stirred at 23 °C for 18 h. The reaction mixture was filtered, the volatiles removed from the filtrate under vacuum, and the resulting solid was washed with pentane at 23 °C to yield **3** as a dark brown powder (2.0 g). A further crop was isolated as a precipitate from the pentane washings stored at −30 °C, to give a total recovery of 2.98 g (32% yield). ^1^H NMR (C_6_D_6_): δ 7.58 (d, 1H, Ar**H**), 7.56 (d, 1H, Ar**H**), 2.56 (s, 3H, I*Me), 2.49 (s, 3H, I*Me), 2.13 (s, 3H, I*Me), 2.12 (s, 3H, I*Me), 2.02 (s, 3H, I*Me), 1.90 (s, 3H, I*Me), 1.47 (s, 9H, C**Me**_3_), 1.39 (s, 9H, C**Me**_3_), 0.75 (s, 3H, Si**Me**), 0.70 (s, 3H, Si**Me**). ^13^C NMR (C_6_D_6_): δ 162.8 (1-**Ar**O), 145.8 (2,4-**Ar**O), 142.9 (I*), 136.8 (2,4-**Ar**O), 135.7 (I*), 135.10 (I*), 135.08 (I*), 131.1 (Si**Ar**O), 130.4 (I*), 130.2 (I*), 129.7 (I*), 127.4 (3,5-**Ar**O), 125.6 (3,5-**Ar**O), 123.7 (I*), 99.1 (SiI*), 35.3 (**C**Me_3_), 34.8 (**C**Me_3_), 31.9 (C**Me**_3_), 30.2 (C**Me**_3_), 21.6 (I*Me), 17.4 (I*Me), 17.2 (I*Me), 16.5 (I*Me), 15.9 (I*Me), 15.1 (I*Me), 3.1 (Si**Me**_2_), 3.0 (Si**Me**_2_). Anal. Calcd for C_31_H_45_Cl_2_OSiZr: C, 59.68; H, 7.27. Found: C, 59.58; H, 7.20.

#### Preparation of ^Me_2_^SB(^^*t*^Bu_2_^ArO,I*)HfCl_2_ (**4**)

To a mixture of **L2** (1.0 g, 2.1 mmol) and HfCl_4_ (0.74 g, 2.3 mmol) was added toluene, and the reaction mixture stirred at 60 °C for 96 h. The product was extracted in toluene, dried, and recrystallized from pentane at −80 °C, to afford **4** (0.015 g, 1% yield). ^1^H NMR (C_6_D_6_): δ 7.59 (s, 2H, Ar**H**), 2.56 (s, 3H, I*Me), 2.52 (s, 3H, I*Me), 2.23 (s, 3H, I*Me), 2.13 (s, 3H, I*Me), 2.04 (s, 3H, I*Me), 1.92 (s, 3H, I*Me), 1.47 (s, 9H, C**Me**_3_), 1.39 (s, 9H, C**Me**_3_), 0.76 (s, 3H, Si**Me**_2_), 0.70 (s, 3H, Si**Me**_2_). ^13^C NMR (C_6_D_6_): δ 162.24 (1-**Ar**O), 145.49 (2,4-**Ar**O), 141.82 (I*), 137.40 (2,4-**Ar**O), 135.34 (I*), 134.68 (I*), 133.88 (I*), 131.29 (Si**Ar**O), 130.03 (I*), 129.52 (I*), 129.31 (I*), 127.35 (3,5-**Ar**O), 125.63 (3,5-**Ar**O), 120.72 (I*), 96.18 (SiI*), 35.21 (**C**Me_3_), 34.78 (**C**Me_3_), 31.93 (C**Me**_3_), 30.14 (C**Me**_3_), 21.66 (I*Me), 17.38 (I*Me), 17.07 (I*Me), 16.45 (I*Me), 15.78 (I*Me), 14.78 (I*Me), 3.41 (Si**Me**), 3.19 (Si**Me**). ^29^Si NMR (C_6_D_6_): δ −13.8 ppm.

#### Preparation of *rac*-^Me,^*n*^Pr^SB(^^*t*^Bu_2_^ArO,I*)TiCl_2_ (**6**)

To a mixture of **L4** (4.0 g, 8.0 mmol) and TiCl_4_·2THF was added toluene, and the resulting slurry stirred at 23 °C for 18 h. The product was extracted in benzene, dried, and recrystallized from pentane at −30 °C, to furnish **6** as a dark red powder (0.288 g, 6% yield). ^1^H NMR (C_6_D_6_): δ 7.57 (d, 1H, Ar**H**), 7.54 (d, 1H, Ar**H**), 2.63 (s, 3H, I*Me), 2.58 (s, 3H, I*Me), 2.23 (s, 3H, I*Me), 2.09 (s, 3H, I*Me), 2.01 (s, 3H, I*Me), 1.88 (s, 3H, I*Me), 1.47 (s, 9H, C**Me**_3_), 1.38 (s, 9H, C**Me**_3_), 0.74 (t, 3H, SiCH_2_CH_2_C**H**_3_), 0.73 (s, 3H, Si**Me**). Additional ^1^H NMR resonances were observed in ^1^H–^13^C HSQC at δ 1.43 and 1.37 ppm corresponding to SiC**H**_2_CH_2_CH_3_ and SiCH_2_C**H**_2_CH_3_. ^13^C NMR (C_6_D_6_): δ 168.2 (1-**Ar**O), 147.1 (2,4-**Ar**O), 145.7 (I*), 138.0 (I*), 136.8 (I*), 136.4 (2,4-**Ar**O), 136.3 (I*), 133.2 (Si**Ar**O), 132.9 (I*), 132.1 (I*), 131.4 (I*), 130.6 (I*), 128.4 (3,5-**Ar**O), 125.1 (3,5-**Ar**O), 109.4 (SiI*), 35.4 (**C**Me_3_), 34.9 (**C**Me_3_), 31.8 (C**Me**_3_), 30.1 (C**Me**_3_), 21.8 (I*Me), 20.7 (I*Me), 18.7 (I*Me), 18.5 (I*Me), 17.7 (I*Me), 17.3 (I*Me), 16.9 (Si**C**H_2_CH_2_Me), 16.62 (SiCH_2_**C**H_2_Me), 16.56 (SiCH_2_CH_2_**Me**), and 1.7 (Si**Me**) ppm. ^29^Si NMR (C_6_D_6_): δ −11.2 ppm. Anal. Calcd for C_33_H_48_Cl_2_OSiTi: C, 65.23; H, 7.96. Found: C, 65.26; H, 7.89.

#### Preparation of *rac*-^Me,Ph^SB(^^*t*^Bu_2_^ArO,I*)TiCl_2_ (**7**)

To a mixture of **L5** (3.0 g, 5.6 mmol) and TiCl_4_·2THF was added toluene, and the resulting dark red slurry was stirred at 23 °C for 51 h. The product was extracted in toluene, washed with pentane, and recrystallized from boiling hexanes to yield **7** as a dark red solid (0.081 g, 3% yield). ^1^H NMR (C_6_D_6_): δ 7.82–7.78 (m, 2H, SiC_6_**H**_5_), 7.59 (d, 1H, Ar**H**), 7.48 (d, 1H, Ar**H**), 7.32–7.23 (m, 3H, SiC_6_**H**_5_), 2.68 (s, 3H, I*Me), 2.57 (s, 3H, I*Me), 2.17 (s, 3H, I*Me), 2.03 (s, 3H, I*Me), 1.98 (s, 3H, I*Me), 1.94 (s, 3H, I*Me), 1.60 (s, 9H, C**Me**_3_), 1.24 (s, 9H, C**Me**_3_), 1.04 (s, 3H, Si**Me**). ^13^C NMR (C_6_D_6_): δ 168.1 (1-**Ar**O), 147.3 (2,4-**Ar**O), 144.4 (I*), 138.9 (I*), 138.0 (I*), 137.0 (I*), 136.6 (2,4-**Ar**O), 136.0 (Si**Ar**O), 135.9 (Si**Ar**), 132.6 (I*), 132.1 (I*), 131.9 (I*), 131.6 (I*), 131.4 (I*), 130.4 (Si**Ar**), 128.8 (3,5-**Ar**O), 128.7 (Si**Ar**), 128.4 (Si**Ar**), 125.6 (3,5-**Ar**O), 110.1, 35.6 (**C**Me_3_), 35.0 (**C**Me_3_), 31.7 (C**Me**_3_), 30.3 (C**Me**_3_), 21.7 (I*Me), 17.2 (I*Me), 17.1 (I*Me), 16.69 (I*Me), 16.66 (I*Me), 15.0 (I*Me), and 4.1 (Si**Me**) ppm. Two additional aromatic resonances were expected but not observed. ^29^Si NMR (C_6_D_6_): δ −16.0. Anal. Calcd for C_36_H_46_Cl_2_OSiTi: C, 67.39; H, 7.23. Found: C, 67.50; H, 7.24.

#### Preparation of ^Me_2_^SB(^^*t*^Bu_2_^ArO,I*)TiMe_2_ (**10**)

To a benzene solution of **2** (0.200 g, 0.345 mmol) was added dropwise MeLi (1.6 M in Et_2_O; 1.04 mmol). Solid byproducts were removed by filtration, and volatiles were removed under vacuum to afford **13** as yellow solids (0.171 g, 92% yield). ^1^H NMR (C_6_D_6_): δ 7.61 (d, 1H, *m*Ar**H**), 7.55 (d, 1H, *m*Ar**H**), 2.61 (s, 3H, I*Me), 2.48 (s, 3H, I*Me), 2.12 (s, 3H, I*Me), 2.08 (s, 3H, I*Me), 1.95 (s, 3H, I*Me), 1.78 (s, 3H, I*Me), 1.71 (s, 9H, C**Me**_3_), 1.40 (s, 9H, C**Me**_3_), 0.87 (s, 3H, Ti**Me**), 0.67 (s, 3H, Si**Me**), 0.61 (s, 3H, Si**Me**), 0.16 (s, 3H, Ti**Me**). ^13^C NMR (C_6_D_6_): δ 165.01 (1-**Ar**O), 144.58 (2,4-**Ar**O), 138.24 (I*), 135.46 (2,4-**Ar**O), 133.88 (I*), 133.79 (I*), 133.15 (I*), 133.02 (Si**Ar**O), 130.26 (I*), 130.05 (I*), 129.51 (I*), 127.44 (3,5-**Ar**O), 125.05 (3,5-**Ar**O), 120.75 (I*), 98.96 (SiI*), 56.92 (Ti**Me**), 55.21 (Ti**Me**), 35.51 (**C**Me_3_), 34.79 (**C**Me_3_), 31.98 (C**Me**_3_), 30.20 (C**Me**_3_), 21.70 (I*Me), 17.43 (I*Me), 16.97 (I*Me), 16.44 (I*Me), 15.43 (I*Me), 14.64 (I*Me), 4.04 (Si**Me**), 2.16 (Si**Me**). ^29^Si NMR (C_6_D_6_): δ −13.8. IR (KBr): 746, 766, 778, 812, 829, 879, 923, 983, 1120, 1183, 1198, 1234, 1306, 1414, and 2954 cm^–1^. Anal. Calcd for C_33_H_50_OSiTi: C, 73.58; H, 9.36. Found: C, 66.71; H, 8.42.

#### Preparation of ^Me_2_^SB(^^*t*^Bu_2_^ArO,I*)Ti(CH_2_SiMe_3_)_2_ (**11**)

To a J. Young’s tap NMR tube were added **2** (0.050 g, 0.086 mmol) and LiCH_2_SiMe_3_ (0.017 g, 0.18 mmol) dissolved in benzene-*d*_6_. The reaction was judged quantitative by ^1^H NMR spectroscopy. Solid byproducts were removed by filtration, and volatiles were removed under vacuum. ^1^H NMR (C_6_D_6_): δ 7.60 (d, 1H, *m*Ar**H**), 7.54 (d, 1H, *m*Ar**H**), 2.67 (s, 3H, I*Me), 2.65 (s, 3H, I*Me), 2.15 (s, 3H, I*Me), 2.08 (s, 3H, I*Me), 2.04 (s, 3H, I*Me), 1.90 (s, 1H, C**H**_2_SiMe_3_), 1.87 (s, 3H, I*Me), 1.75 (s, 9H, C**Me**_3_), 1.36 (s, 9H, C**Me**_3_), 0.96 (dd, 2H, C**H**_2_SiMe_3_), 0.76 (s, 3H, Si**Me**), 0.69 (s, 3H, Si**Me**), 0.15 (s, 9H, CH_2_Si**Me**_3_), 0.12 (s, 9H, CH_2_Si**Me**_3_), −0.88 (d, 1H, C**H**_2_SiMe_3_). ^13^C NMR (C_6_D_6_): δ 166.04 (1-ArO), 144.22 (2,4-ArO), 136.84 (I*), 135.32 (2,4-ArO), 134.26 (I*), 133.36 (SiArO), 132.99 (I*), 132.12 (I*), 131.95 (I*), 130.47 (I*), 128.83 (I*), 128.16 (3,5-ArO), 125.38 (3,5-ArO), 122.39 (I*), 98.12 (SiI*), 84.11 (TiCH_2_SiMe_3_), 70.66 (TiCH_2_SiMe_3_), 35.83 (CMe_3_), 34.68 (CMe_3_), 31.86 (CMe_3_), 31.30 (CMe_3_), 21.39 (I*Me), 17.76 (I*Me), 16.92 (I*Me), 16.58 (I*Me), 16.50 (I*Me), 16.34 (I*Me), 4.87 (SiMe), 4.71 (SiMe), 3.72 (CH_2_SiMe_3_), 2.71 (CH_2_SiMe_3_). ^29^Si NMR (C_6_D_6_): δ 0.3 (CH_2_SiMe_3_), 0.0 (CH_2_SiMe_3_), −0.5 (CH_2_SiMe_3_), −14.8 (SiMe_2_). Anal. Calcd for C_39_H_66_OSi_3_Ti: C, 68.58; H, 9.76. Found: C, 67.27; H, 9.53.

#### Preparation of ^Me_2_^SB(^^*t*^Bu_2_^ArO,I*)Ti(CH_2_Ph)_2_ (**12**)

To a solution of **2** (0.20 g, 0.34 mmol) in benzene was added PhCH_2_MgCl (2 M in THF, 1.0 mmol), resulting in a dark red solution. The mixture was stirred at 23 °C for 20 h during which the solution changed in color to a dark orange. The volatiles were removed under vacuum, and the product was extracted in toluene (with ∼5 mL dioxane to aid precipitation of MgCl_2_)^92^ to afford **15** as a bright orange solid (0.180 g, 76% yield). ^1^H NMR (C_6_D_6_): δ 7.62 (d, 1H, *m*Ar**H**), 7.55 (d, 1H, *m*Ar**H**), 7.07–7.01 (m, 4H, CH_2_C_6_**H**_5_), 6.93 (ddd, 4H, CH_2_C_6_**H**_5_), 6.86 (qt, 2H, CH_2_C_6_**H**_5_), 2.74 (d, 1H, C**H**_2_C_6_H_5_), 2.66 (d, 1H, C**H**_2_C_6_H_5_), 2.39 (s, 3H, I*Me), 2.14 (s, 3H, I*Me), 2.12 (d, 1H, C**H**_2_Ph), 2.10 (s, 3H, I*Me), 2.04 (s, 3H, I*Me), 1.96 (s, 3H, I*Me), 1.74 (s, 3H, I*Me), 1.55 (s, 9H, C**Me**_3_), 1.37 (s, 9H, C**Me**_3_), 1.31 (d, 1H, C**H**_2_Ph), 0.65 (s, 3H, Si**Me**), 0.64 (s, 3H, Si**Me**). ^13^C NMR (C_6_D_6_): δ 165.50 (1-**Ar**O), 149.58, 149.01, 144.93 (2,4-**Ar**O), 138.56, 135.85, 134.47, 133.57, 133.44, 133.21, 131.56, 130.15, 129.67, 128.81, 128.61, 128.38, 128.35, 127.82 (3,5-**Ar**O), 127.56 (2,4-**Ar**O), 126.22, 125.30 (3,5-**Ar**O), 123.01, 122.82 (CH_2_**Ph**), 122.38 (CH_2_**Ph**), 101.12 (SiI*), 86.23 (Ti**C**H_2_Ph), 86.03 (Ti**C**H_2_Ph), 35.51 (**C**Me_3_), 34.76 (**C**Me_3_), 31.89 (C**Me**_3_), 30.76 (C**Me**_3_), 21.70 (I*Me), 17.35 (I*Me), 17.10 (I*Me), 16.62 (I*Me), 15.02 (I*Me), 14.76 (I*Me), 4.68 (Si**Me**), 3.17 (Si**Me**). Two additional aromatic resonances were expected but not observed. ^29^Si NMR (C_6_D_6_): δ −13.8. Anal. Calcd for C_45_H_58_OSiTi: C, 78.23; H, 8.46. Found: C, 69.46; H, 8.04.

#### Preparation of ^Me_2_^SB(^^*t*^Bu_2_^ArO,I*)Ti(NMe_2_)_2_ (**13**)

To a J. Young’s tap NMR tube were added **2** (0.050 g, 0.086 mmol) and LiNMe_2_ (0.0088 g, 0.17 mmol) and dissolved in benzene-*d*_6_. The reaction was heated at 80 °C for 42 h, and conversion was judged quantitative by ^1^H NMR spectroscopy. Solid byproducts were removed by filtration and volatiles were removed under vacuum. ^1^H NMR (C_6_D_6_): δ 7.67 (d, 1H, *m*ArH), 7.61 (d, 1H, *m*ArH), 3.00 (s, 6H, NMe_2_), 2.60 (s, 6H, NMe_2_), 2.58 (s, 3H, I*Me), 2.47 (s, 3H, I*Me), 2.15 (s, 3H, I*Me), 2.08 (s, 3H, I*Me), 2.06 (s, 3H, I*Me), 1.91 (s, 3H, I*Me), 1.51 (s, 9H, CMe_3_), 1.44 (s, 9H, CMe_3_), 0.88 (s, 3H, SiMe), 0.72 (s, 3H, SiMe). ^13^C NMR (C_6_D_6_): δ 166.75 (1-**Ar**O), 142.86 (2,4-**Ar**O), 139.30 (I*), 136.43 (2,4-**Ar**O), 133.11 (Si**Ar**O), 132.22 (I*), 131.39 (I*), 130.27 (I*), 129.92 (I*), 129.56 (I*), 128.35 (I*), 127.61 (3,5-**Ar**O), 124.68 (3,5-**Ar**O), 116.42 (I*), 94.88 (SiI*), 47.98 (N**Me**_2_), 47.67 (N**Me**_2_), 35.41 (**C**Me_3_), 34.71 (**C**Me_3_), 32.12 (C**Me**_3_), 30.48 (C**Me**_3_), 21.35 (I*Me), 17.69 (I*Me), 16.96 (I*Me), 16.56 (I*Me), 15.85 (I*Me), 15.23 (I*Me), 5.64 (Si**Me**), 2.99 (Si**Me**). ^29^Si NMR (C_6_D_6_): δ −14.6. Anal. Calcd for C_35_H_56_N_2_OSiTi: C, 70.32; H, 9.61; N, 4.69. Found: C, 63.49; H, 9.49; N, 3.42.

#### Preparation of ^Me_2_^SB(^^*t*^Bu_2_^ArO,I*)Ti(OEt)_2_ (**14**)

To a J. Young’s tap NMR tube were added **2** (0.050 g, 0.086 mmol) and NaOEt (0.012 g, 0.17 mmol) and dissolved in benzene-*d*_6_. The reaction was heated at 80 °C for 137 h, and conversion was judged quantitative by ^1^H NMR spectroscopy. Solid byproducts were removed by filtration, and volatiles were removed under vacuum. ^1^H NMR (C_6_D_6_): δ 7.62 (d, 1H, *m*Ar**H**), 7.54 (d, 1H, *m*Ar**H**), 4.42 (qd, 2H, OC**H**_2_CH_3_), 3.98–3.80 (m, 2H, OC**H**_2_CH_3_), 2.58 (s, 3H, I*Me), 2.51 (s, 3H, I*Me), 2.31 (s, 3H, I*Me), 2.05 (s, 3H, I*Me), 1.98 (s, 3H, I*Me), 1.89 (s, 3H, I*Me), 1.45 (s, 9H, C**Me**_3_), 1.42 (s, 9H, C**Me**_3_), 1.18 (t, 3H, OCH_2_C**H**_3_), 1.01 (t, 3H, OCH_2_C**H**_3_), 0.88 (s, 3H, Si**Me**), 0.83 (s, 3H, Si**Me**). ^13^C NMR (C_6_D_6_): δ 167.16 (1-**Ar**O), 143.05 (2,4-**Ar**O), 142.14 (I*), 135.75 (2,4-**Ar**O), 133.55 (Si**Ar**O), 132.57 (I*), 132.37 (I*), 129.75 (I*), 129.15 (I*), 128.66 (I*), 127.56 (I*), 127.14 (3,5-**Ar**O), 124.49 (3,5-**Ar**O), 119.98 (I*), 100.20 (SiI*), 71.50 (O**C**H_2_Me), 68.70 (O**C**H_2_Me), 35.25 (**C**Me_3_), 34.72 (**C**Me_3_), 32.13 (C**Me**_3_), 29.83 (C**Me**_3_), 21.89 (OCH_2_**Me**), 19.75 (OCH_2_**Me**), 19.73 (I*Me), 17.66 (I*Me), 17.01 (I*Me), 16.31 (I*Me), 16.20 (I*Me), 15.05 (I*Me), 4.06 (Si**Me**), 1.75 (Si**Me**). Anal. Calcd for C_35_H_55_O_3_SiTi: C, 70.09; H, 9.24. Found: C, 73.82; H, 10.25.

#### Preparation of ^Me_2_^SB(^^*t*^Bu_2_^ArO,I*)Ti(ODipp)_2_ (**15**)

To a J. Young’s tap NMR tube were added **2** (0.050 g, 0.086 mmol) and KODipp (0.037 g, 0.17 mmol) and dissolved in benzene-*d*_6_. The reaction was heated at 60 °C for 24 h, and conversion was judged quantitative by ^1^H NMR spectroscopy. Solid byproducts were removed by filtration, and volatiles were removed under vacuum. ^1^H NMR (C_6_D_6_): δ 7.56 (d, 1H, *m*Ar**H**), 7.53 (d, 1H, *m*Ar**H**), 7.09–6.87 (m, 6H, O(^*i*^Pr_2_)C_6_**H**_3_), 3.52 (hept, 2H, C**H** (CH_3_)_2_), 3.14 (hept, 2H, C**H** (CH_3_)_2_), 2.37 (s, 3H, I*Me), 2.31 (s, 3H, I*Me), 2.24 (s, 3H, I*Me), 2.06 (s, 3H, I*Me), 2.04 (s, 3H, I*Me), 1.92 (s, 3H, I*Me), 1.56 (s, 9H, C**Me**_3_), 1.38 (s, 9H, C**Me**_3_), 1.20 (d, 6H, CH(C**H**_3_)_2_), 1.16 (d, 6H, CH(C**H**_3_)_2_), 0.98 (d, 6H, CH(C**H**_3_)_2_), 0.89 (d, 6H, CH(C**H**_3_)_2_), 0.70 (s, 3H, Si**Me**), 0.70 (s, 3H, Si**Me**). ^13^C NMR (C_6_D_6_): δ 168.70 (1-**Ar**O), 162.23, 161.76, 144.82, 143.82 (2,4-**Ar**O), 138.99, 138.94, 138.45, 135.28 (2,4-**Ar**O), 134.49, 133.62, 133.23, 132.37, 129.73, 128.99, 128.62 (3,5-**Ar**O), 128.56, 128.35, 127.97, 127.53, 125.17, 125.01 (3,5-**Ar**O), 123.63 (**Ar**^Dipp^), 123.09 (**Ar**^Dipp^), 122.52 (**Ar**^Dipp^), 122.28 (**Ar**^Dipp^), 106.10 (SiI*), 35.76 (**C**Me_3_), 34.61 (**C**Me_3_), 31.85 (C**Me**_3_), 30.90 (C**Me**_3_), 26.78 (Ar**C**HMe_2_), 26.73(Ar**C**HMe_2_), 25.39 (ArCH**Me**_2_), 24.90 (ArCH**Me**_2_), 24.40 (ArCH**Me**_2_), 21.59 (I*Me), 17.70 (I*Me), 17.20 (I*Me), 17.10 (I*Me), 16.59 (I*Me), 15.20 (I*Me), 4.15 (Si**Me**), 3.54 (Si**Me**). ^29^Si NMR (C_6_D_6_): δ −13.1 ppm. Anal. Calcd for C_55_H_78_O_3_SiTi: C, 76.53; H, 9.11. Found: C, 72.99; H, 8.76.

#### Preparation of ^Me_2_^SB(^^*t*^Bu,Me^ArO,I*)TiMe_2_ (**16**)

To a J. Young’s tap NMR tube were added **1** (0.083 g, 0.15 mmol) and MeLi (1.6 M in Et_2_O; 0.46 mmol) and dissolved in benzene-*d*_6_. Volatiles were removed under vacuum, and the product was extracted in pentane to give **16** as a yellow solid (0.013 g, 17% yield). ^1^H NMR (C_6_D_6_): δ 7.26 (d, 1H, *m*Ar**H**), 7.23 (d, 1H, *m*Ar**H**), 2.61 (s, 3H I*Me), 2.48 (s, 3H I*Me), 2.31 (s, 3H I*Me), 2.13 (s, 3H I*Me), 2.09 (s, 3H I*Me), 1.97 (s, 3H I*Me), 1.76 (s, 3H 4-**Me**-ArO), 1.67 (s, 9H C**Me**_3_), 0.87 (s, 3H Ti**Me**), 0.61 (s, 3H Si**Me**), 0.57 (s, 3H Si**Me**), 0.15 (s, 3H Ti**Me**). ^13^C NMR (C_6_D_6_): δ 165.07 (**Ar**O), 138.26 (I*), 136.00 (**Ar**O), 134.41 (**Ar**O), 133.82 (**Ar**O), 133.16 (I*), 133.03 (I*), 131.41 (**Ar**O), 131.29 (I*), 130.30 (I*), 130.05 (I*), 129.52 (I*), 128.94 (**Ar**O), 120.56 (I*), 98.96 (SiI*), 56.85 (Ti**Me**), 55.14 (Ti**Me**), 35.15 (**C**Me_3_), 30.14 (C**Me**_3_), 21.77 (I*Me), 21.40 (I*Me), 17.41 (I*Me), 16.97 (I*Me), 16.44 (I*Me), 15.40 (I*Me), 14.52 (4-**Me**-ArO), 3.90 (Si**Me**), 1.81 (Si**Me**).

#### Preparation of ^Me_2_^SB(^^*t*^Bu,Me^ArO,I*)Ti(CH_2_SiMe_3_)_2_ (**17**)

To a J. Young’s tap NMR tube were added **1** (0.100 g, 0.186 mmol) and LiCH_2_SiMe_3_ (0.0350 g, 0.372 mmol) and dissolved in benzene-*d*_6_. The reaction was heated at 80 °C for 137 h, and conversion was judged quantitative by ^1^H NMR spectroscopy. Solid byproducts were removed by filtration, and volatiles were removed under vacuum. ^1^H NMR (C_6_D_6_): δ 7.25 (d, 1H, *m*Ar**H**), 7.22 (d, 1H, *m*Ar**H**), 2.67 (s, 3H, I*Me), 2.66 (s, 3H, I*Me), 2.24 (s, 3H, I*Me), 2.15 (s, 3H, I*Me), 2.08 (s, 3H, I*Me), 2.06 (s, 3H, I*Me), 1.92 (d, 1H, C**H**_2_SiMe_3_), 1.88 (s, 3H, 4-**Me**-ArO), 1.70 (s, 9H, C**Me**_3_), 0.97 (d, 1H, C**H**_2_SiMe_3_), 0.94 (d, 1H, C**H**_2_SiMe_3_), 0.69 (s, 3H, Si**Me**), 0.64 (s, 3H, Si**Me**), 0.17 (s, 9H, CH_2_Si**Me**_3_), 0.13 (s, 9H, CH_2_Si**Me**_3_), −0.90 (d, 1H, C**H**_2_SiMe_3_).

### Crystallographic Data Collection and Structure Determination

Crystals were mounted on MiTeGen MicroMounts using perfluoropolyether oil and rapidly transferred to a goniometer head on a diffractometer fitted with an Oxford Cryostreams Cryostream open-flow nitrogen cooling device.^93^ Data collections were carried out at 100 or 150 K on an Oxford Diffraction Supernova or a Rigaku XtaLAB Synergy DW diffractometer using mirror-monochromated Cu Kα radiation (λ = 1.54178 Å) and data were processed using CrysAlisPro.^94^ Structures were solved using direct methods (SIR92) or a charge flipping algorithm (SUPERFLIP) and refined on *F*^2^ by full-matrix lest-squares regression.^95−98^ Geometric calculations were performed using PLATON, and illustrations were created using ORTEP.^99,100^

### Preparation of Cationic Intermediates

A J. Young’s tap NMR tube was charged with approximately 0.010 g of either **2** or **10** and dissolved in approximately 0.6 mL of perduterated solvent. Equivalents of one of TB, AB, or MAO were added, and the reactions were monitored by ^1^H NMR spectroscopy.

### Preparation of Supported Catalysts

sMAO (third generation, 250 mg) and the precatalyst ([Al_sMAO_]_0_/[Ti]_0_ = 200) were weighed into a Schlenk flask. Toluene (40 mL) was added, and the slurry was heated at 60 °C with regular swirling for 1 h or until the supernatant had discolored. The colored solid was allowed to settle, then the supernatant was decanted and the product dried under vacuum at room temperature (1 × 10^–2^ mbar).

### Polymerization of Ethylene

In a typical polymerization reaction, a 150 mL Rotaflo ampule with a magnetic stir bar is charged with TIBA (150 mg), solid catalyst (10 mg), and hexanes (50 mL). The ampule was sealed, cycled onto a Schlenk line, and degassed under reduced pressure. It was cycled a further two times using ethylene as a purge gas, while the vessel was brought to temperature in a thermostatic oil bath with the stirring set at 1000 rpm. The stopcock was opened to ethylene at a pressure of 2 bar, and the timer was started. After 30 min, the vessel was degassed under partial vacuum and then filtered on a sintered glass frit (porosity 3) and washed with 2 × 25 mL pentane. The polyethylene was dried under vacuum until constant weight. All runs were carried out at least in duplicate to ensure reproducibility.

### High-Throughput Polymerization Screening

Experiments were performed at Xplore s.r.l. (University of Naples Federico II) by V. Busico, R. Cipullo, L. Rongo, and A. Mingione. Polymerization experiments were conducted in a FreeSlate Parallel Pressure Reactor (PPR) platform consisting of 48 reaction cells. The procedure has been described extensively elsewhere.^101^

## Supporting Information Available

General experimental considerations, additional synthetic details, representative NMR spectra, crystallographic data, DFT studies, polymerization data, and polymer characterization (PDF)

## References

[ref1] Kaminsky W. Highly active metallocene catalysts for olefin polymerization. J. Chem. Soc., Dalton Trans. 1998, 1413–1418. 10.1039/a800056e. 19826710

[ref2] Coperet C.; Comas-Vives A.; Conley M. P.; Estes D. P.; Fedorov A.; Mougel V.; Nagae H.; Nunez-Zarur F.; Zhizhko P. A. Surface Organometallic and Coordination Chemistry toward Single-Site Heterogeneous Catalysts: Strategies, Methods, Structures, and Activities. Chem. Rev. 2016, 116, 323–421. 10.1021/acs.chemrev.5b00373. 26741024

[ref3] Kaminsky W.; Funck A.; Hahnsen H. New application for metallocene catalysts in olefin polymerization. Dalton Trans. 2009, 8803–8810. 10.1039/b910542p. 19826710

[ref4] Braunschweig H.; Breitling F. M. Constrained geometry complexes - Synthesis and applications. Coord. Chem. Rev. 2006, 250, 2691–2720. 10.1016/j.ccr.2005.10.022.

[ref5] Suhm J.; Schneider M. J.; Mülhaupt R. Influence of metallocene structures on ethene copolymerization with 1-butene and 1-octene. J. Mol. Catal. A: Chem. 1998, 128, 215–227. 10.1016/S1381-1169(97)00175-1.

[ref6] Li Y.; Liu Q.; Bao J.; Yiu S.-M.; Chan M. C. W. Coplanar binuclear group 4 post-metallocene complexes supported by chelating μ-(σ_2_-aryl) ligands: characterisation and olefin polymerisation catalysis. Dalton Trans. 2024, 53, 346–353. 10.1039/D3DT03641C. 38050668

[ref7] Uborsky D. V.; Sharikov M. I.; Goryunov G. P.; Li K. M.; Dall’Anese A.; Zuccaccia C.; Vittoria A.; Iovine T.; Galasso G.; Ehm C.; Macchioni A.; Busico V.; Voskoboynikov A. Z.; Cipullo R. Manipulating pre-equilibria in olefin polymerization catalysis: backbone-stiffening converts a living into a highly active salan-type catalyst. Inorg. Chem. Front. 2023, 10, 6401–6406. 10.1039/D3QI01537H.

[ref8] Okuda J. Molecular Olefin Polymerization Catalysts: From Metallocenes to Half-Sandwich Complexes with Functionalized Cyclopentadienyl Ligands. J. Organomet. Chem. 2023, 1000, 122833 10.1016/j.jorganchem.2023.122833.

[ref9] Tuskaev V. A.; Gagieva S. C.; Churakov A. V.; Kurmaev D. A.; Magomedov K. F.; Evseeva M. D.; Golubev E. K.; Buzin M. I.; Nikiforova G. G.; Saracheno D.; Shatokhin S. S.; Bulychev B. M. Novel titanium (IV) diolate complexes with thiophene-containing OSO-type ligand as pre-catalyst for ethylene polymerization and ethylene - propylene copolymerization. J. Organomet. Chem. 2022, 977, 122457 10.1016/j.jorganchem.2022.122457.

[ref10] Jeong S. M.; Park J. Y.; Hyun Y. B.; Baek J. W.; Kim H.; Yoon Y.; Chung S.; Lee J.; Lee B. Y. Syntheses of Silylene-Bridged Thiophene-Fused Cyclopentadienyl ansa-Metallocene Complexes for Preparing High-Performance Supported Catalyst. Catalysts 2022, 12, 283 10.3390/catal12030283.

[ref11] Dong Z.; Huang W.; Liu X.; Yu F.; Long C.; Feng S.; Luo L.; Chen Z. R. Molecular Bottlebrush Supported Mono(phenoxy-imine) Metal Complexes: Synthesis and Ethylene Polymerization. Macromolecules 2021, 54, 9385–9392. 10.1021/acs.macromol.1c01000.

[ref12] Furuyama R.; Saito J.; Ishii S.; Makio H.; Mitani M.; Tanaka H.; Fujita T. Fluorinated bis(phenoxy-imine) Ti complexes with MAO: Remarkable catalysts for living ethylene and syndioselective living propylene polymerization. J. Organomet. Chem. 2005, 690, 4398–4413. 10.1016/j.jorganchem.2005.03.060.

[ref13] Katayama H.; Nabika M.; Imai A.; Miyashita A.; Watanabe T.; Johohji H.; Oda Y.; Hanaoka H. Transition metal complexes soluble in saturated hydrocarbon solvents, manufacture thereof, olefin polymerization catalysts containing the same, and producing olefin polymers using the same. WO 9703992 A1, 1997.

[ref14] Hanaoka H.; Hino T.; Souda H.; Yanagi K.; Oda Y.; Imai A. Synthesis and characterization of titanium and zirconium complexes with silicone-bridged phenoxycyclopentadienyl ligands. J. Organomet. Chem. 2007, 692, 4059–4066. 10.1016/j.jorganchem.2007.06.006.

[ref15] Hanaoka H.; Hino T.; Nabika M.; Kohno T.; Yanagi K.; Oda Y.; Imai A.; Mashima K. Synthesis and characterization of titanium alkyl, oxo, and diene complexes bearing a SiMe2-bridged phenoxy-cyclopentadienyl ligand and their catalytic performance for copolymerization of ethylene and 1-hexene. J. Organomet. Chem. 2007, 692, 4717–4724. 10.1016/j.jorganchem.2007.06.012.

[ref16] Senda T.; Hanaoka H.; Hino T.; Oda Y.; Tsurugi H.; Mashima K. Substituent Effects on Silicon of Bridged Tetramethylcyclopentadienyl–Phenoxy Titanium Complexes for Controlling the Regiochemistry and Molecular Weight in 1-Olefin Polymerization. Macromolecules 2009, 42, 8006–8009. 10.1021/ma901678u.

[ref17] Nabika M.; Katayama H.; Watanabe T.; Kawamura-Kuribayashi H.; Yanagi K.; Imai A. ansa-Cyclopentadienyl-Phenoxy Titanium(IV) Complexes (PHENICS): Synthesis, Characterization, and Catalytic Behavior in Olefin Polymerization. Organometallics 2009, 28, 3785–3792. 10.1021/om900019q.

[ref18] Senda T.; Hanaoka H.; Nakahara S.; Oda Y.; Tsurugi H.; Mashima K. Rational Design of Silicon-Bridged Fluorenyl-Phenoxy Group 4 Metal Complexes as Catalysts for Producing High Molecular Weight Copolymers of Ethylene and 1-Hexene at Elevated Temperature. Macromolecules 2010, 43, 2299–2306. 10.1021/ma902700v.

[ref19] Senda T.; Hanaoka H.; Hino T.; Oda Y.; Tsurugi H.; Mashima K. Substituent Effects on Silicon of Bridged Tetramethylcyclopentadienyl–Phenoxy Titanium Complexes for Controlling the Regiochemistry and Molecular Weight in 1-Olefin Polymerization. Macromolecules 2010, 43, 5162 10.1021/ma100651j.

[ref20] Tudor J.; Barlow S.; Payne B. R.; O’Hare D.; Nguyen P.; Evans C. E. B.; Manners I. Synthesis and Structure of [Fe(η^5^-C_9_Me_6_)(η^5^-C_5_H_4_)SiMe_2_]: A Mixed-Ring [1]Ferrocenophane. Organometallics 1999, 18, 2281–2284. 10.1021/om980988u.

[ref21] Ransom P.; Ashley A.; Thompson A.; O’Hare D. Synthesis, structure and characterisation of rac and meso-ansa-bridged permethylindenyl cobalt complexes. J. Organomet. Chem. 2009, 694, 1059–1068. 10.1016/j.jorganchem.2008.09.002.

[ref22] Buffet J. C.; Arnold T. A. Q.; Turner Z. R.; Angpanitcharoen P.; O’Hare D. Synthesis and characterisation of permethylindenyl zirconium complexes and their use in ethylene polymerisation. RSC Adv. 2015, 5, 87456–87464. 10.1039/C5RA20465H.

[ref23] Buffet J. C.; Turner Z. R.; O’Hare D. Popcorn-shaped polyethylene synthesised using highly active supported permethylindenyl metallocene catalyst systems. Chem. Commun. 2018, 54, 10970–10973. 10.1039/C8CC05350B. 30206626

[ref24] Lamb J. V.; Buffet J. C.; Turner Z. R.; O’Hare D. Group 4 permethylindenyl complexes for slurry-phase polymerisation of ethylene. Polym. Chem. 2019, 10, 1386–1398. 10.1039/C8PY01796D.

[ref25] Williams T. J.; Buffet J. C.; Turner Z. R.; O’Hare D. Group 4 permethylindenyl constrained geometry complexes for ethylene polymerisation catalysis. Catal. Sci. Technol. 2018, 8, 5454–5461. 10.1039/C8CY01374H.

[ref26] Williams T. J.; Lamb J. V.; Buffet J.-C.; Khamnaen T.; O’Hare D. Synthesis of ultra-high molecular weight poly(ethylene)-co-(1-hexene) copolymers through high-throughput catalyst screening. RSC Adv. 2021, 11, 5644–5650. 10.1039/D1RA00446H. 35423070 PMC8694734

[ref27] Collins Rice C. G.; Buffet J.-C.; Turner Z. R.; O’Hare D. Supported permethylindenyl titanium catalysts for the synthesis of disentangled ultra-high molecular weight polyethylene (disUHMWPE). Chem. Commun. 2021, 57, 8600–8603. 10.1039/D1CC03418A. 34365496

[ref28] Collins Rice C. G.; Buffet J.-C.; Turner Z. R.; O’Hare D. Efficient synthesis of thermoplastic elastomeric amorphous ultra-high molecular weight atactic polypropylene (UHMWaPP). Polym. Chem. 2022, 13, 5597–5603. 10.1039/D2PY00708H.

[ref29] Collins Rice C. G.; Morris L. J.; Buffet J.-C.; Turner Z. R.; O’Hare D. Fully tuneable ethylene-propylene elastomers using a supported permethylindenyl-phenoxy (PHENI*) catalyst. Chem. Commun. 2023, 59, 12128–12131. 10.1039/D3CC03791F. 37740304

[ref30] Collins Rice C. G.; Morris L. J.; Buffet J.-C.; Turner Z. R.; O’Hare D. Towards designer polyolefins: highly tuneable olefin copolymerisation using a single permethylindenyl post-metallocene catalyst. Chem. Sci. 2024, 15, 250–258. 10.1039/D3SC04861F. PMC10731910 38131091

[ref31] Zou C.; Si G.; Chen C. A general strategy for heterogenizing olefin polymerization catalysts and the synthesis of polyolefins and composites. Nat. Commun. 2022, 13, 1954 10.1038/s41467-022-29533-9. 35414067 PMC9005542

[ref32] Choi Y.; Soares J. B. Supported single-site catalysts for slurry and gas-phase olefin polymerisation. Can. J. Chem. Eng. 2012, 90, 646–671. 10.1002/cjce.20583.

[ref33] Severn J. R.; Chadwick J. C.; Duchateau R.; Friederichs N. “Bound but Not Gagged” Immobilizing Single-Site α-Olefin Polymerization Catalysts. Chem. Rev. 2005, 105, 4073–4147. 10.1021/cr040670d. 16277372

[ref34] Wongwaiwattanakul P.; Jongsomjit B. Copolymerization of ethylene/1-octene via different pore sized silica-based-supported zirconocene/dMMAO catalysts. Catal. Commun. 2008, 10, 118–122. 10.1016/j.catcom.2008.08.007.

[ref35] Yang H.; Lolage S.; van der Eem J.; Rastogi S.; Romano D. Silica-supported catalyst for the synthesis of low entangled UHMWPE suitable for solid-state processing. Mol. Catal. 2024, 552, 113668 10.1016/j.mcat.2023.113668.

[ref36] Soga K.; Kaminaka M. Polymerization of propene with zirconocene-containing supported catalysts activated by common trialkylaluminiums. Makromol. Chem. 1993, 194, 1745–1755. 10.1002/macp.1993.021940621.

[ref37] Pothirat T.; Jongsomjit B.; Praserthdam P. A comparative study of SiO2- and ZrO2-supported zirconocene/MAO catalysts on ethylene/1-olefin copolymerization. Catal. Commun. 2008, 9, 1426–1431. 10.1016/j.catcom.2007.12.005.

[ref38] Manianglung C.; Lee J. S.; Ko Y. S. Olefin polymerization behavior of metallocene immobilized inside pore of metal-organic frameworks. Catal. Today 2023, 411–412, 113893 10.1016/j.cattod.2022.08.035.

[ref39] Buffet J.-C.; Wanna N.; Arnold T. A. Q.; Gibson E. K.; Wells P. P.; Wang Q.; Tantirungrotechai J.; O’Hare D. Highly Tunable Catalyst Supports for Single-Site Ethylene Polymerization. Chem. Mater. 2015, 27, 1495–1501. 10.1021/cm503433q.

[ref40] Mazhar H.; Shehzad F.; Hong S.-G.; Al-Harthi M. A. Enhancing Metallocene Catalyst Activity: Utilizing Layered Double Hydroxide for Ethylene-Propylene Copolymerization. Macromol. Mater. Eng. 2023, 309, 2300245 10.1002/mame.202300245.

[ref41] Kilpatrick A. F. R.; Buffet J.-C.; Nørby P.; Rees N. H.; Funnell N. P.; Sripothongnak S.; O’Hare D. Synthesis and Characterization of Solid Polymethylaluminoxane: A Bifunctional Activator and Support for Slurry-Phase Ethylene Polymerization. Chem. Mater. 2016, 28, 7444–7450. 10.1021/acs.chemmater.6b03009.

[ref42] Arnold T. A. Q.; Turner Z. R.; Buffet J. C.; O’Hare D. Polymethylaluminoxane supported zirconocene catalysts for polymerisation of ethylene. J. Organomet. Chem. 2016, 822, 85–90. 10.1016/j.jorganchem.2016.08.015.

[ref43] Yang L.; Powell D. R.; Houser R. P. Structural variation in copper(i) complexes with pyridylmethylamide ligands: structural analysis with a new four-coordinate geometry index, τ4. Dalton Trans. 2007, 955–964. 10.1039/B617136B. 17308676

[ref44] Klosin J.; Kruper W. J.; Nickias P. N.; Roof G. R.; De Waele P.; Abboud K. A. Heteroatom-Substituted Constrained-Geometry Complexes. Dramatic Substituent Effect on Catalyst Efficiency and Polymer Molecular Weight. Organometallics 2001, 20, 2663–2665. 10.1021/om010016d.

[ref45] Feng S.; Klosin J.; Kruper W. J.; McAdon M. H.; Neithamer D. R.; Nickias P. N.; Patton J. T.; Wilson D. R.; Abboud K. A.; Stern C. L. Synthesis and Characterization of Cyclohexadienyl-Based Constrained Geometry Complexes. Organometallics 1999, 18, 1159–1167. 10.1021/om980847s.

[ref46] Park Y. J.; Ryu J. Y.; Hwang S.; Park K. H.; Lee J. M.; Cho S.; Lee S.; Saha M. L.; Stang P. J.; Lee J. Cationic Ti Complexes with Three [N,O]-Type Tetrazolyl Ligands: Ti↔Fe Transmetalation within Fe Metallascorpionate Complexes. Inorg. Chem. 2017, 56, 14060–14068. 10.1021/acs.inorgchem.7b02209. 29120170

[ref47] Mitani M.; Mohri J. I.; Yoshida Y.; Saito J.; Ishii S.; Tsuru K.; Matsui S.; Furuyama R.; Nakano T.; Tanaka H.; Kojoh S. I.; Matsugi T.; Kashiwa N.; Fujita T. Living Polymerization of Ethylene Catalyzed by Titanium Complexes Having Fluorine-Containing Phenoxy–Imine Chelate Ligands. J. Am. Chem. Soc. 2002, 124, 3327–3336. 10.1021/ja0117581. 11916417

[ref48] Oakes D. C. H.; Gibson V. C.; White A. J. P.; Williams D. J. Highly Active Titanium-Based Olefin Polymerization Catalysts Supported by Bidentate Phenoxyamide Ligands. Inorg. Chem. 2006, 45, 3476–3477. 10.1021/ic060146k. 16634572

[ref49] Bott R. K. J.; Hughes D. L.; Schormann M.; Bochmann M.; Lancaster S. J. Monocyclopentadienyl phenoxy-imine and phenoxy-amine complexes of titanium and zirconium and their application as catalysts for 1-alkene polymerisation. J. Organomet. Chem. 2003, 665, 135–149. 10.1016/S0022-328X(02)02106-X.

[ref50] Okuda J.; Fokken S.; Kang H. C.; Massa W. Synthesis and Characterization of Mononuclear Titanium Complexes Containing a Bis(phenoxy) Ligand Derived from 2,2′-Methylene-bis(6-tert-butyl-4-methylphenol). Chem. Ber. 1995, 128, 221–227. 10.1002/cber.19951280304.

[ref51] Tanaka R.; Viehmann P.; Hecht S. Bis(phenoxy-azo)titanium(IV) Complexes: Synthesis, Structure, and Catalytic Activity in Styrene Polymerization. Organometallics 2012, 31, 4216–4220. 10.1021/om3001636.

[ref52] Shannon R. Revised effective ionic radii and systematic studies of interatomic distances in halides and chalcogenides. Acta Crystallogr., Sect. A: Found. Crystallogr. 1976, 32, 751–767. 10.1107/S0567739476001551.

[ref53] Shannon R. D.; Prewitt C. T. Effective ionic radii in oxides and fluorides. Acta Crystallogr., Sect. B: Struct. Sci., Cryst. Eng. Mater. 1969, 25, 925–946. 10.1107/S0567740869003220.

[ref54] Kaminsky W. New polymers by metallocene catalysis. Macromol. Chem. Phys. 1996, 197, 3907–3945. 10.1002/macp.1996.021971201.

[ref55] Yano A.; Hasegawa S.; Yamada S.; Akimoto A. Influence of activators on ethylene polymerization with diphenylmethylidene-(cyclopentadienyl)(fluorenyl)zirconium dichloride catalysts at high temperature. J. Mol. Catal. A: Chem. 1999, 148, 77–86. 10.1016/S1381-1169(99)00107-7.

[ref56] Chen M. C.; Roberts J. A. S.; Marks T. J. Marked Counteranion Effects on Single-Site Olefin Polymerization Processes. Correlations of Ion Pair Structure and Dynamics with Polymerization Activity, Chain Transfer, and Syndioselectivity. J. Am. Chem. Soc. 2004, 126, 4605–4625. 10.1021/ja036288k. 15070378

[ref57] Ciardelli F.; Altomare A.; Michelotti M. From homogeneous to supported metallocene catalysts. Catal. Today 1998, 41, 149–157. 10.1016/S0920-5861(98)00045-5.

[ref58] Wang W.; Fan Z. q.; Feng L. x. Ethylene polymerization and ethylene/1-hexene copolymerization using homogeneous and heterogeneous unbridged bisindenyl zirconocene catalysts. Eur. Polym. J. 2005, 41, 2380–2387. 10.1016/j.eurpolymj.2005.04.026.

[ref59] Chen Y. X.; Marks T. J. Constrained Geometry” Dialkyl Catalysts. Efficient Syntheses, C–H Bond Activation Chemistry, Monomer–Dimer Equilibration, and α-Olefin Polymerization Catalysis. Organometallics 1997, 16, 3649–3657. 10.1021/om970288+.

[ref60] Ransom P.; Ashley A. E.; Brown N. D.; Thompson A. L.; O’Hare D. Synthesis, Characterization, and Polymerization Studies of Ethylenebis(hexamethylindenyl) Complexes of Zirconium and Hafnium. Organometallics 2011, 30, 800–814. 10.1021/om100986j.

[ref61] Siedle A. R.; Lamanna W. M.; Newmark R. A.; Stevens J.; Richardson D. E.; Ryan M. The role of non-coordinating anions in homogeneous olefin polymerization. Makromol. Chem., Macromol. Symp. 1993, 66, 215–224. 10.1002/masy.19930660119.

[ref62] Musikabhumma K.; Spaniol T. P.; Okuda J. Tritylpyridinium tetrakis(pentafluorophenyl)borate as an efficient activator for “constrained-geometry” catalysts in ethylene polymerization. J. Mol. Catal. A: Chem. 2004, 208, 73–81. 10.1016/j.molcata.2003.07.007.

[ref63] Zhou J.; Lancaster S. J.; Walker D. A.; Beck S.; Thornton-Pett M.; Bochmann M. Synthesis, Structures, and Reactivity of Weakly Coordinating Anions with Delocalized Borate Structure: The Assessment of Anion Effects in Metallocene Polymerization Catalysts. J. Am. Chem. Soc. 2001, 123, 223–237. 10.1021/ja002820h. 11456508

[ref64] Williams V. C.; Irvine G. J.; Piers W. E.; Li Z.; Collins S.; Clegg W.; Elsegood M. R. J.; Marder T. B. Novel Trityl Activators with New Weakly Coordinating Anions Derived from C_6_F_4_–1,2-[B(C_6_F_5_)_2_]_2_: Synthesis, Structures, and Olefin Polymerization Behavior. Organometallics 2000, 19, 1619–1621. 10.1021/om0001974.

[ref65] Langford D.; Göttker-Schnetmann I.; Wimmer F. P.; Casper L. A.; Kenyon P.; Winter R. F.; Mecking S. Tetrakis[3,5-bis(pentafluorosulfanyl)phenyl]borate: A Weakly Coordinating Anion Probed in Polymerization Catalysis. Organometallics 2019, 38, 2710–2713. 10.1021/acs.organomet.9b00332.

[ref66] Chen E. Y.; Marks T. J. Cocatalysts for metal-catalyzed olefin polymerization: activators, activation processes, and structure-activity relationships. Chem. Rev. 2000, 100, 1391–1434. 10.1021/cr980462j. 11749269

[ref67] Rudnik E.; Dobkowski Z. Thermal degradation of UHMWPE. J. Therm. Anal. 1997, 49, 471–475. 10.1007/BF01987473.

[ref68] Eskelinen M.; Seppälä J. V. Effect of polymerization temperature on the polymerization of ethylene with dicyclopentadienylzirconiumdichloride/methylalumoxane catalyst. Eur. Polym. J. 1996, 32, 331–335. 10.1016/0014-3057(95)00148-4.

[ref69] Fan L.; Harrison D.; Woo T. K.; Ziegler T. A Density Functional Study of Ethylene Insertion into the M-CH3 Bond of the Constrained Geometry Catalysts [(SiH2-C5H4-NH)MCH3]+ (M = Ti, Zr, Hf) and (SiH2-C5H4-NH)TiCH3. Organometallics 1995, 14, 2018–2026. 10.1021/om00004a064.

[ref70] Silanes I.; Mercero J. M.; Ugalde J. M. Comparison of Ti, Zr, and Hf as Cations for Metallocene-Catalyzed Olefin Polymerization. Organometallics 2006, 25, 4483–4490. 10.1021/om050790r.

[ref71] Kaminsky W. Zirconocene catalysts for olefin polymerization. Catal. Today 1994, 20, 257–271. 10.1016/0920-5861(94)80005-7.

[ref72] Kim S. J.; Lee Y. J.; Kang E.; Kim S. H.; Ko J.; Lee B.; Cheong M.; Suh I. H.; Kang S. O. Syntheses, Structural Characterizations, and Catalytic Behavior of ansa-Metallocene Complexes Derived from 1,1-Dicyclopentadienyl-1-silacycloalkanes. Organometallics 2003, 22, 3958–3966. 10.1021/om030287j.

[ref73] Möhring P. C.; Coville N. J. Homogeneous group 4 metallocene ziegler-natta catalysts: The influence of cyclopentadienyl-ring substituents. J. Organomet. Chem. 1994, 479, 1–29. 10.1016/0022-328X(94)84087-3.

[ref74] Alonso-Moreno C.; Antiñolo A.; López-Solera I.; Otero A.; Prashar S.; Rodríguez A. M.; Villaseñor E. Niobium, titanium, zirconium and hafnium complexes incorporating germanium bridged ansa ligands. X-Ray crystal structures of [Zr{Me2Ge(η5-C5Me4)2}Cl2] and [M{Me2Ge(η5-C5Me4)(η5-C5H4)}Cl2] (M = Zr, Hf). J. Organomet. Chem. 2002, 656, 129–138. 10.1016/S0022-328X(02)01576-0.

[ref75] Gil M. P.; Casagrande O. L. Titanium and zirconium complexes containing sterically hindered hydrotris(pyrazolyl)borate ligands: synthesis, structural characterization, and ethylene polymerization studies. J. Organomet. Chem. 2004, 689, 286–292. 10.1016/j.jorganchem.2003.10.013.

[ref76] Luo L.; Marks T. J. Ziegler-Natta catalyst activation. Thermodynamic and kinetic aspects of metallocenium ion-pair formation, dissociation, and structural reorganization. Top. Catal. 1999, 7, 97–106. 10.1023/A:1019155515306.

[ref77] Punzalan E.; Froese R. D.; Zimmerman P. M. Revealing the Interactions between the Flexible Polymer and Counteranion during Propagation and Termination for Olefin Polymerization with the Ti(IV) Constrained Geometry Catalyst. Organometallics 2023, 42, 3236–3248. 10.1021/acs.organomet.3c00365.

[ref78] Bochmann M. Kinetic and mechanistic aspects of metallocene polymerisation catalysts. J. Organomet. Chem. 2004, 689, 3982–3998. 10.1016/j.jorganchem.2004.07.006.

[ref79] Martínez-Araya J. I.; Quijada R.; Toro-Labbé A. The Mechanism of Ethylene Polymerization Reaction Catalyzed by Group IVB Metallocenes. A Rational Analysis Through the Use of Reaction Force. J. Phys. Chem. C 2012, 116, 21318–21325. 10.1021/jp302702h.

[ref80] Patel K.; Chikkali S. H.; Sivaram S. Ultrahigh molecular weight polyethylene: Catalysis, structure, properties, processing and applications. Prog. Polym. Sci. 2020, 109, 101290 10.1016/j.progpolymsci.2020.101290.

[ref81] Antonov A. A.; Bryliakov K. P. Post-metallocene catalysts for the synthesis of ultrahigh molecular weight polyethylene: Recent advances. Eur. Polym. J. 2021, 142, 110162 10.1016/j.eurpolymj.2020.110162.

[ref82] Busico V.; Pellecchia R.; Cutillo F.; Cipullo R. High-Throughput Screening in Olefin-Polymerization Catalysis: From Serendipitous Discovery Towards Rational Understanding. Macromol. Rapid Commun. 2009, 30, 1697–1708. 10.1002/marc.200900246. 21638440

[ref83] Vickroy V. V.; Schneider H.; Abbott R. F. The separation of SEC curves of HDPE into flory distributions. J. Appl. Polym. Sci. 1993, 50, 551–554. 10.1002/app.1993.070500318.

[ref84] Soares J. B. P.; Hamielec A. E. Deconvolution of chain-length distributions of linear polymers made by multiple-site-type catalysts. Polymer 1995, 36, 2257–2263. 10.1016/0032-3861(95)95305-K.

[ref85] Mehdiabadi S.; Lhost O.; Vantomme A.; Soares J. B. P. Ethylene Polymerization Kinetics and Microstructure of Polyethylenes Made with Supported Metallocene Catalysts. Ind. Eng. Chem. Res. 2021, 60, 9739–9754. 10.1021/acs.iecr.1c01091.

[ref86] Gates D. P.; Svejda S. A.; Oñate E.; Killian C. M.; Johnson L. K.; White P. S.; Brookhart M. Synthesis of Branched Polyethylene Using (α-Diimine)nickel(II) Catalysts: Influence of Temperature, Ethylene Pressure, and Ligand Structure on Polymer Properties. Macromolecules 2000, 33, 2320–2334. 10.1021/ma991234+.

[ref87] Soares J. B. P.; Kim J. D.; Rempel G. L. Analysis and Control of the Molecular Weight and Chemical Composition Distributions of Polyolefins Made with Metallocene and Ziegler–Natta Catalysts. Ind. Eng. Chem. Res. 1997, 36, 1144–1150. 10.1021/ie960479x.

[ref88] Shriver D. F.; Drezdon M. A. The Manipulation of Air-Sensitive Compounds, 2nd ed.; Wiley, 1986.

[ref89] Alías F. M.; Barlow S.; Tudor J. S.; O’Hare D.; Perry R. T.; Nelson J. M.; Manners I. Synthesis, characterisation and structure of a strained ring-tilted bis(indenyl)iron complex. J. Organomet. Chem. 1997, 528, 47–58. 10.1016/S0022-328X(96)06434-0.

[ref90] Görl C.; Betthausen E.; Alt H. G. Di- and trinuclear iron/titanium and iron/zirconium complexes with heterocyclic ligands as catalysts for ethylene polymerization. Polyhedron 2016, 118, 37–51. 10.1016/j.poly.2016.07.033.

[ref91] Monreal M. J.; Thomson R. K.; Cantat T.; Travia N. E.; Scott B. L.; Kiplinger J. L. UI4(1,4-dioxane)2, [UCl4(1,4-dioxane)]2, and UI3(1,4-dioxane)1.5: Stable and Versatile Starting Materials for Low- and High-Valent Uranium Chemistry. Organometallics 2011, 30, 2031–2038. 10.1021/om200093q.

[ref92] Fischer R.; Görls H.; Meisinger P. R.; Suxdorf R.; Westerhausen M. Structure-Solubility Relationship of 1,4-Dioxane Complexes of Di(hydrocarbyl)magnesium. Chem.—Eur. J. 2019, 25, 12830–12841. 10.1002/chem.201903120. 31328293 PMC7027550

[ref93] Cosier J.; Glazer A. M. A Nitrogen-Gas-Stream Cryostat for General X-Ray-Diffraction Studies. J. Appl. Crystallogr. 1986, 19, 105–107. 10.1107/S0021889886089835.

[ref94] Oxford Diffraction/Agilent Technologies UK Ltd. CrysAlis^Pro^: Yarnton, England, 2013.

[ref95] Altomare A.; Cascarano G.; Giacovazzo C.; Guagliardi A. Completion and Refinement of Crystal-Structures with Sir92. J. Appl. Crystallogr. 1993, 26, 343–350. 10.1107/S0021889892010331.

[ref96] Palatinus L.; Chapuis G. SUPERFLIP - a computer program for the solution of crystal structures by charge flipping in arbitrary dimensions. J. Appl. Crystallogr. 2007, 40, 786–790. 10.1107/S0021889807029238.

[ref97] Betteridge P. W.; Carruthers J. R.; Cooper R. I.; Prout K.; Watkin D. J. CRYSTALS version 12: software for guided crystal structure analysis. J. Appl. Crystallogr. 2003, 36, 1487 10.1107/S0021889803021800.

[ref98] Spek A. L. PLATON SQUEEZE: a tool for the calculation of the disordered solvent contribution to the calculated structure factors. Acta Crystallogr., Sect. C: Struct. Chem. 2015, 71, 9–18. 10.1107/S2053229614024929. 25567569

[ref99] Spek A. L. Single-crystal structure validation with the program PLATON. J. Appl. Crystallogr. 2003, 36, 7–13. 10.1107/S0021889802022112.

[ref100] Farrugia L. J. WinGX and ORTEP for Windows: an update. J. Appl. Crystallogr. 2012, 45, 849–854. 10.1107/S0021889812029111.

[ref101] Busico V.; Cipullo R.; Mingione A.; Rongo L. Accelerating the Research Approach to Ziegler-Natta Catalysts. Ind. Eng. Chem. Res. 2016, 55, 2686–2695. 10.1021/acs.iecr.6b00092.

