# Supplementary material for: Trends in Structure and Ethylene Polymerization Reactivity of Transition-Metal Permethylindenyl-phenoxy (PHENI*) Complexes
Source: Organometallics. 2024 Feb 14;43(4):540–56. doi: 10.1021/acs.organomet.3c00503 (PMC10900520; doi:10.1021/acs.organomet.3c00503)
Supplement: Supplementary file 1 — om3c00503_si_001.pdf [file om3c00503_si_001.pdf]

Supplementary Material

Trends in structure and ethylene polymerization reactivity of transition metal permethylindenyl-phenoxy (PHENI*) complexes

Clement G. Collins Rice, Justin A. Hayden, Adam D. Hawkins, Louis J. Morris, Zoë R. Turner, Jean-Charles Buffet, and Dermot O'Hare

Chemistry Research Laboratory, Department of Chemistry, University of Oxford, 12 Mansfield Road, Oxford, OX1 3TA, United Kingdom

Table of Contents

1. NMR spectra of synthesized compounds S1
    1.1. Synthesis of $^{Me_2}SB(^{tBu_2}ArO,I^*)TaCl_3$ (**18**) S38
2. Crystallographic parameters S41
    2.1. Supplementary X-ray crystal structures S47
    2.2. Solid-G ligand parameters S50
3. NMR spectra of cationic complexes S56
4. ssNMR spectra of solid catalysts S61
5. Density Functional Theory calculations S69
6. Homogeneous polymerization data S71
7. Heterogeneous polymerization data S72
    7.1. Structure-activity relationships S76
8. Ethylene uptake profiles S77
9. Gel-permeation chromatography S77
10. Differential scanning calorimetry S82
11. High pressure high-throughput screening S85
    11.1. Ethylene uptake profiles S85
    11.2. Gel-permeation chromatography S87
12. References S88

1. NMR spectra of synthesized compounds

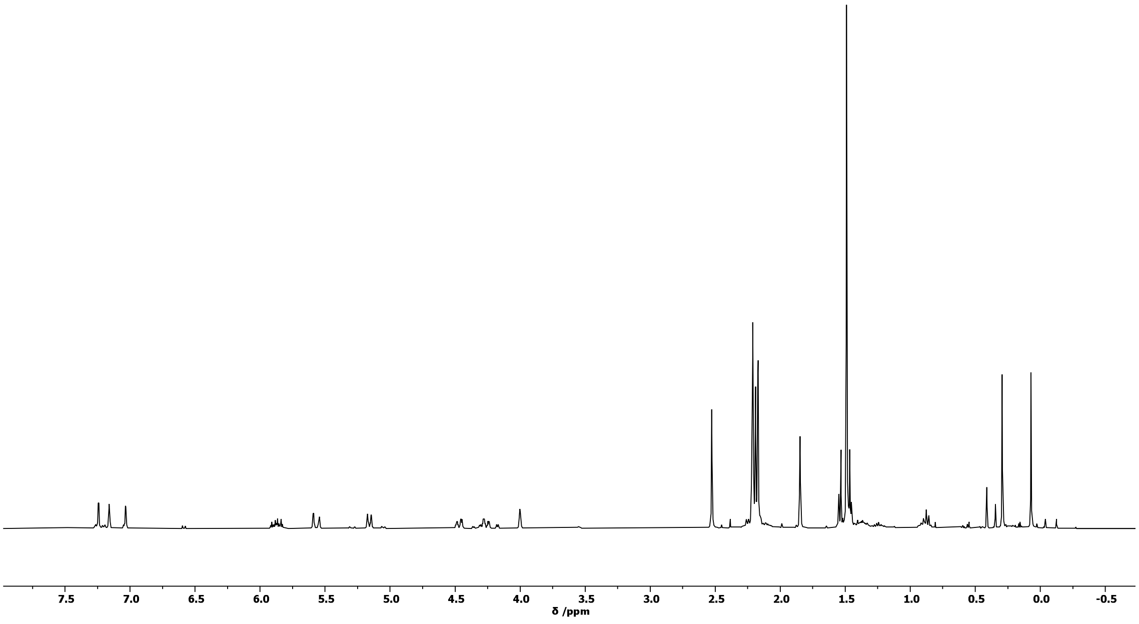

Figure S1 ^{1}H NMR spectrum of crude $^{Me_2}SB(^{tBu,Me}ArOAllyl,I^*)H$ (**P1**) (benzene-d_6, 400 MHz, 298 K).

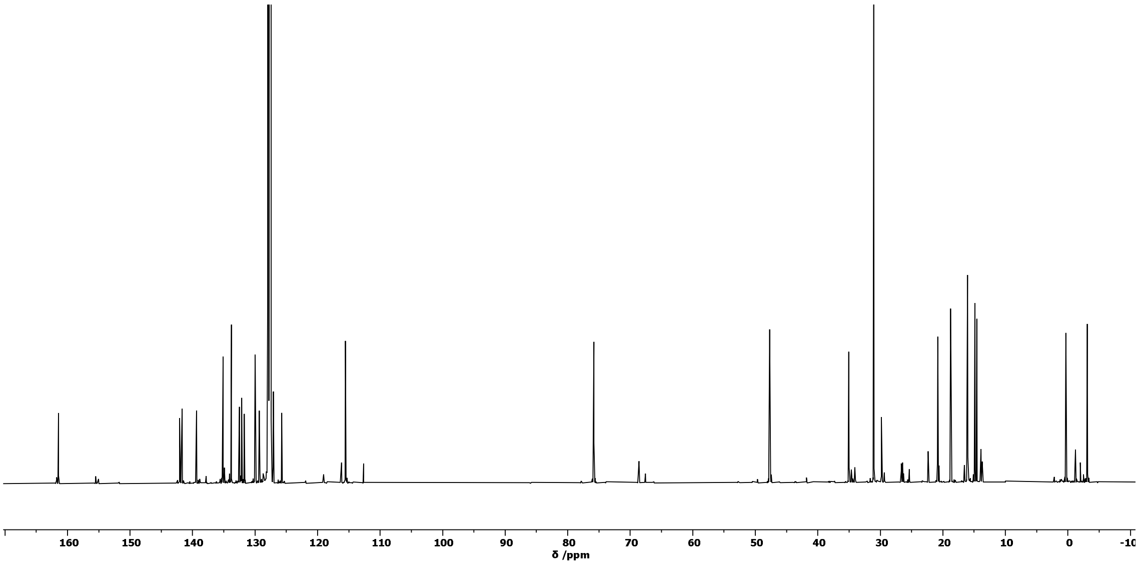

Figure S2 $^{13}C\{^{1}H\}$ NMR spectrum of crude $^{Me_2}SB(^{tBu,Me}ArOAllyl,I^*)H$ (**P1**) (benzene-d_6, 126 MHz, 298 K).

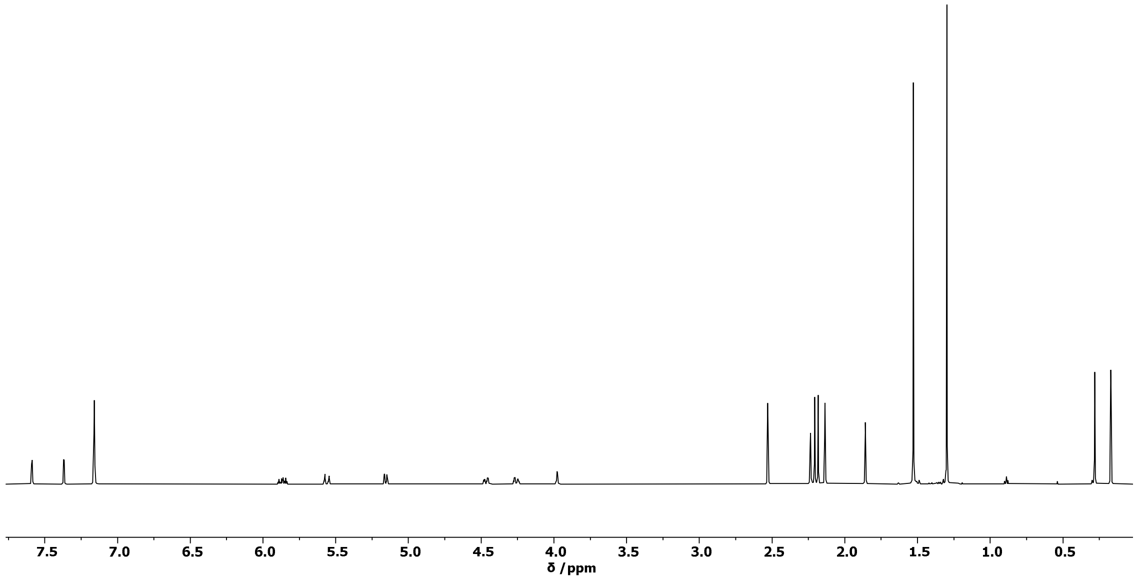

Figure S3 ^{1}H NMR spectrum of recrystallized ^{Me_2}SB($^{^tBu_2}$ArOAllyl,I*)H (**P2**) (benzene-d_6, 600 MHz, 298 K).

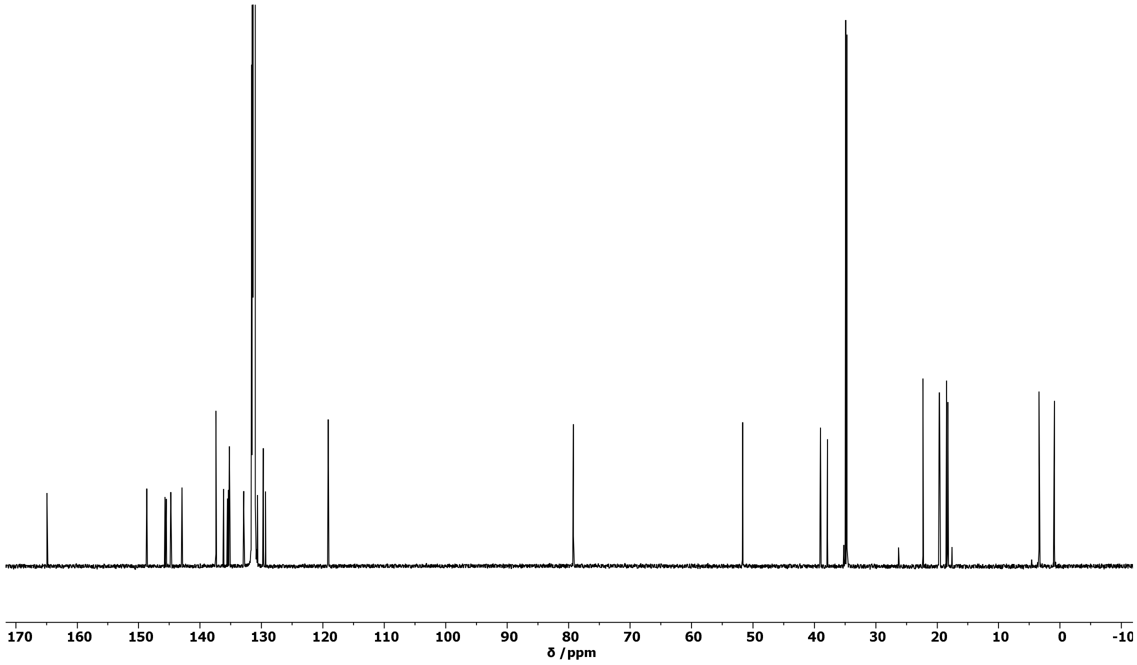

Figure S4 ^{13}C{^{1}H} NMR spectrum of recrystallized ^{Me_2}SB($^{^tBu_2}$ArOAllyl,I*)H (**P2**) (benzene-d_6, 151 MHz, 298 K).

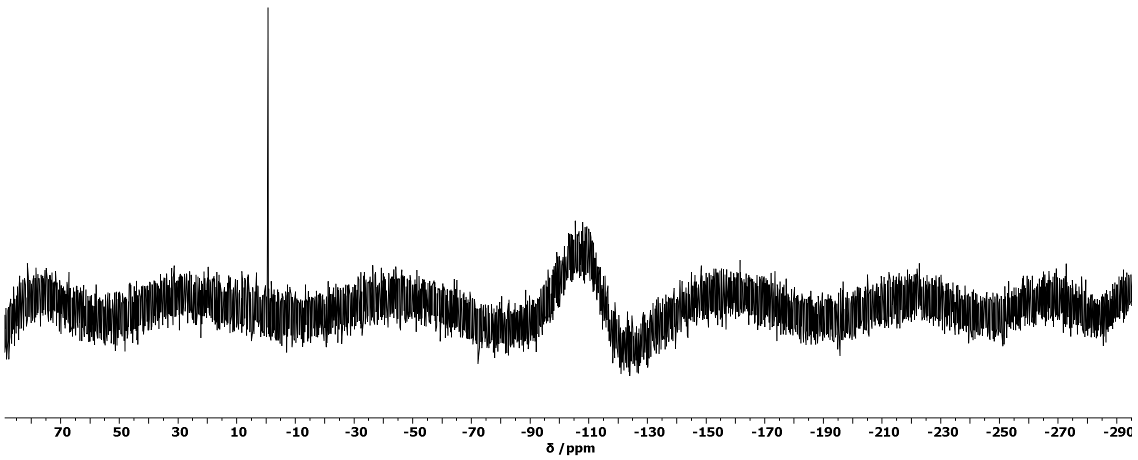

Figure S5 ^{29}Si NMR spectrum of recrystallized ^{Me2}SB(^{tBu2}ArOAllyl,I*)H (**P2**) (benzene-d_6, 119 MHz, 298 K).

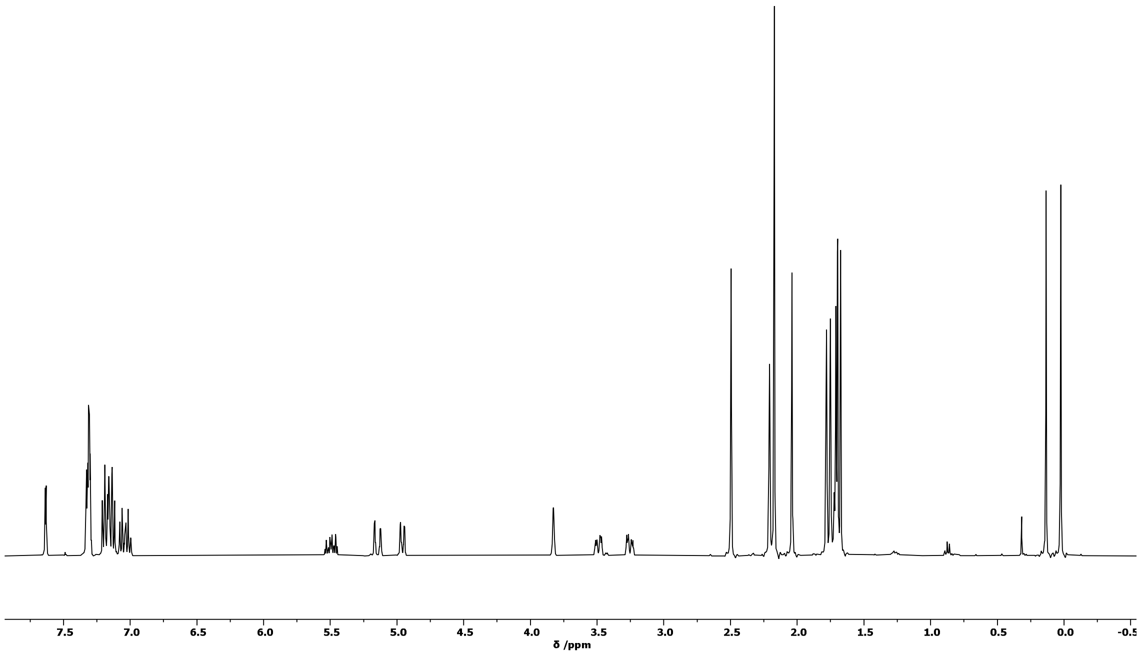

Figure S6 ^{1}H NMR spectrum of recrystallized ^{Me2}SB(^{Cumyl2}ArOAllyl,I*)H (**P3**) (benzene-d_6, 400 MHz, 298 K).

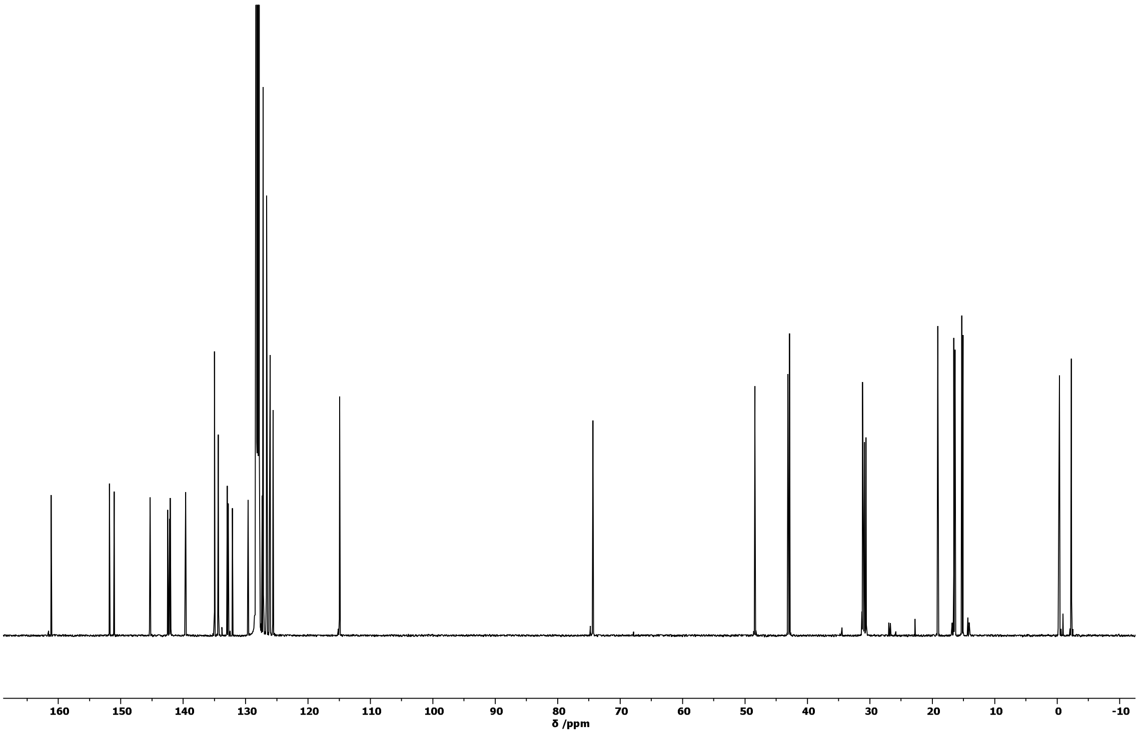

Figure S7 $^{13}C\{^{1}H\}$ NMR spectrum of recrystallized $^{Me_2}SB(^{Cumyl_2}ArOAllyl,I^*)H$ (**P3**) (benzene-d_6, 126 MHz, 298 K).

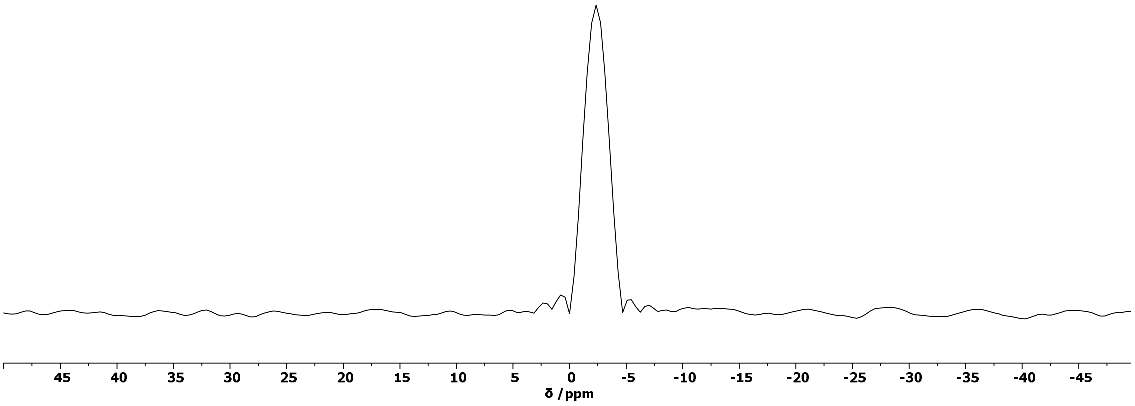

Figure S8 ^{29}Si NMR spectrum of recrystallized $^{Me_2}SB(^{Cumyl_2}ArOAllyl,I^*)H$ (**P3**) (^{1}H-observed HMBC, benzene-d_6, 99 MHz, 298 K).

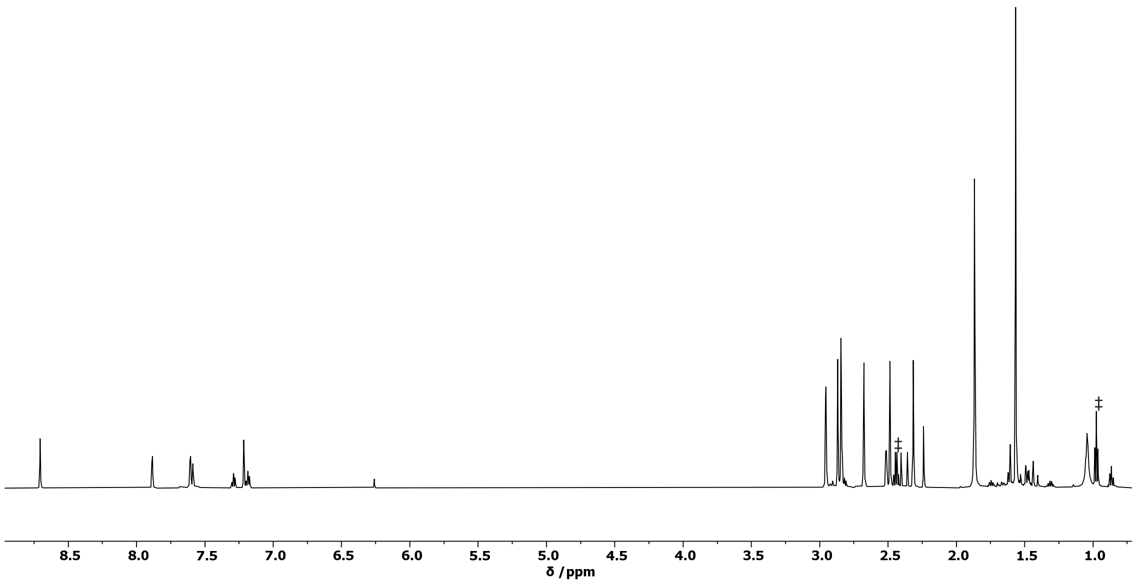

Figure S9 ^{1}H NMR spectrum of ^{Me_2}SB(^{tBu_2}ArO,I*)Li_2·$(NEt_3)_{0.43}$ (**L2**) (pyridine-d_5, 600 MHz, 298 K). ‡ denotes residual NEt_3.

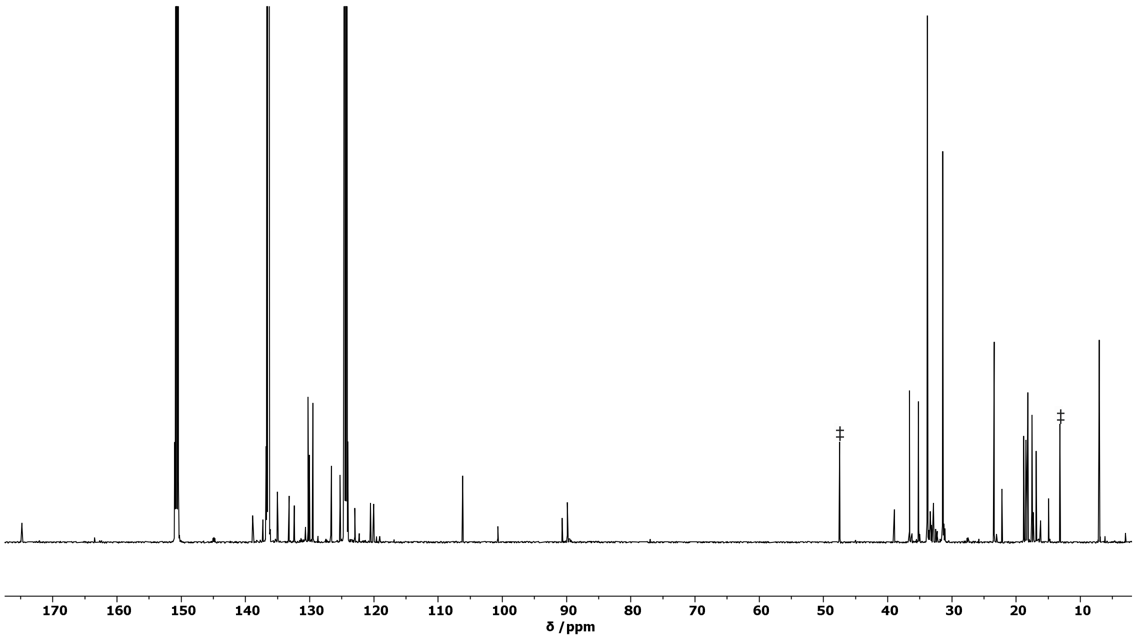

Figure S10 ^{13}C{^{1}H} NMR spectrum of ^{Me_2}SB(^{tBu_2}ArO,I*)Li_2·$(NEt_3)_{0.43}$ (**L2**) (pyridine-d_5, 151 MHz, 298 K). ‡ denotes residual NEt_3.

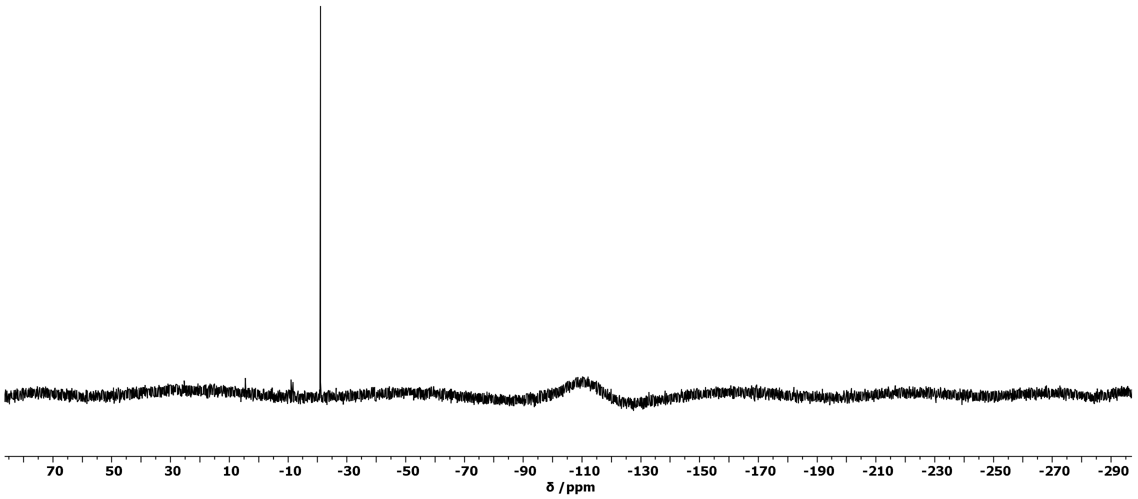

Figure S11 ^{29}Si NMR spectrum of ^{Me2}SB(^{tBu2}ArO,I*)Li$_2$ (**L2**) (pyridine-*d*$_5$, 119 MHz, 298 K).

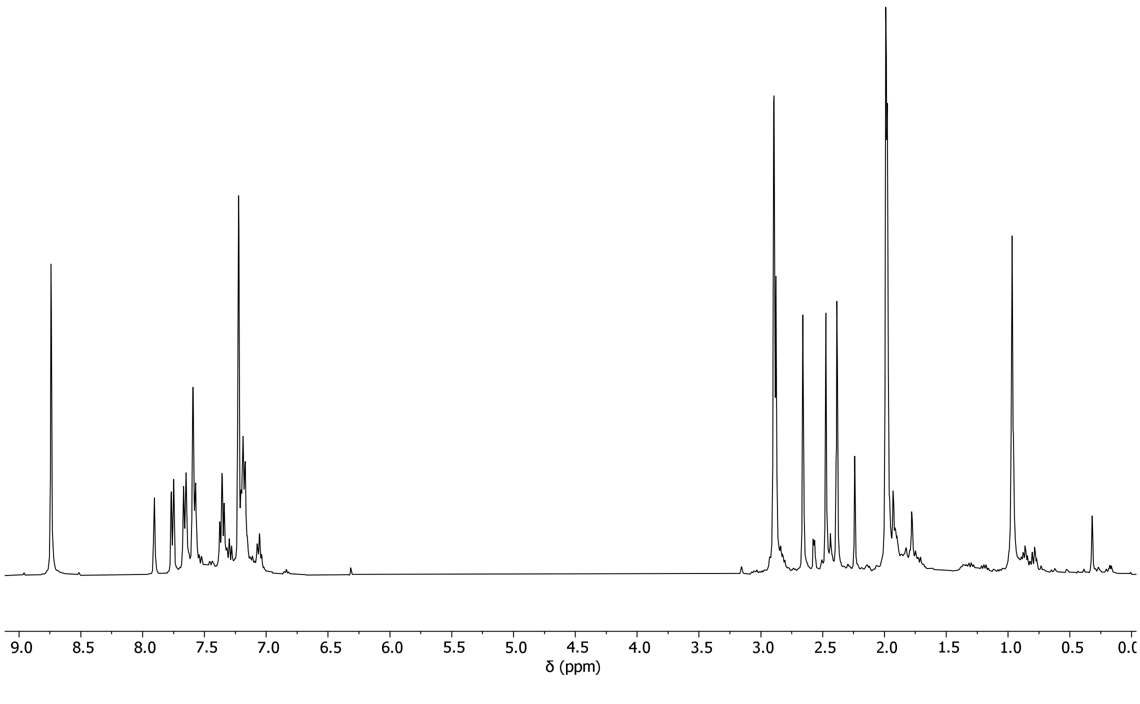

Figure S12 ^{1}H NMR spectrum of crude ^{Me2}SB(^{Cumyl2}ArO,I*)Li$_2$ (**L3**) (pyridine-*d*$_5$, 400 MHz, 298 K).

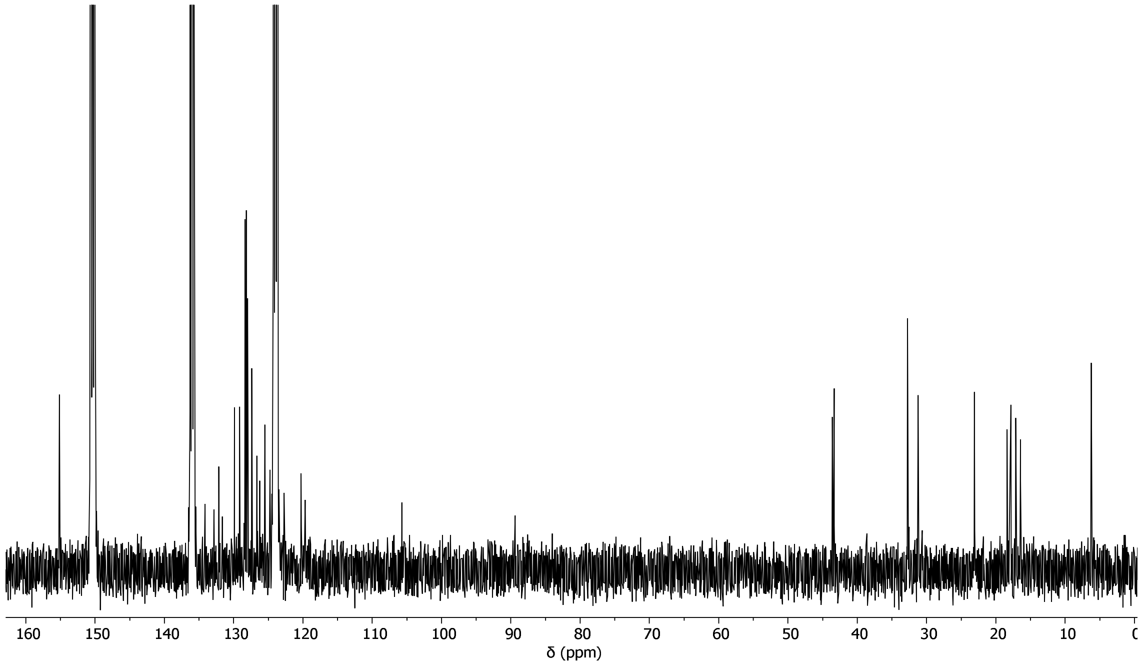

Figure S13 $^{13}C\{^{1}H\}$ NMR spectrum of crude ^{Me_2}SB(^{Cumyl_2}ArO,I*)Li$_2$ (**L3**) (pyridine-d_5, 101 MHz, 298 K).

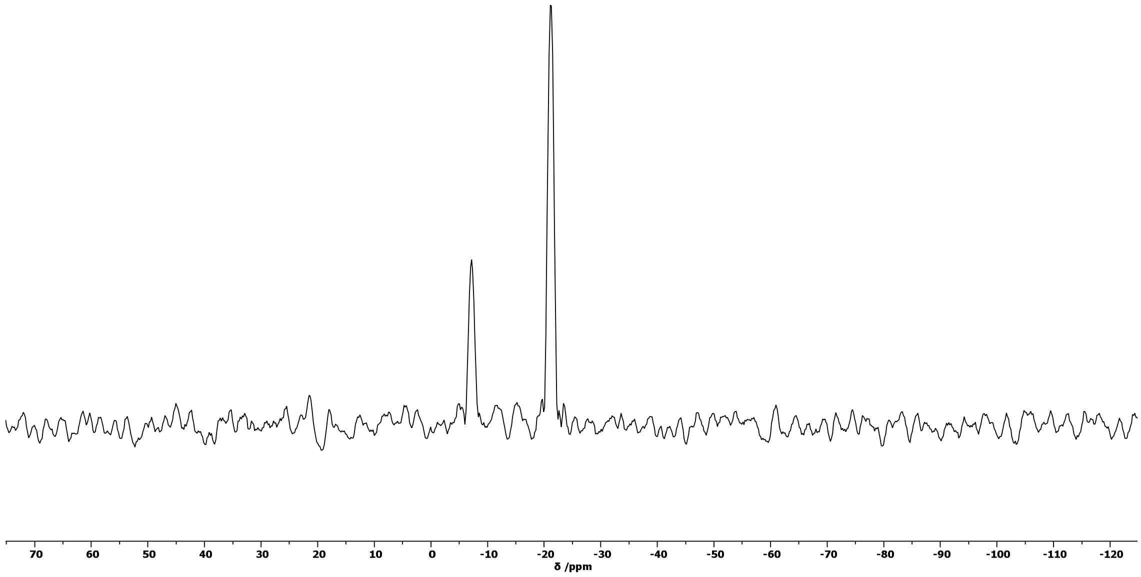

Figure S14 ^{29}Si NMR spectrum of crude ^{Me_2}SB(^{Cumyl_2}ArO,I*)Li$_2$ (**L3**) (^{1}H-observed HMBC, pyridine-d_5, 80 MHz, 298 K).

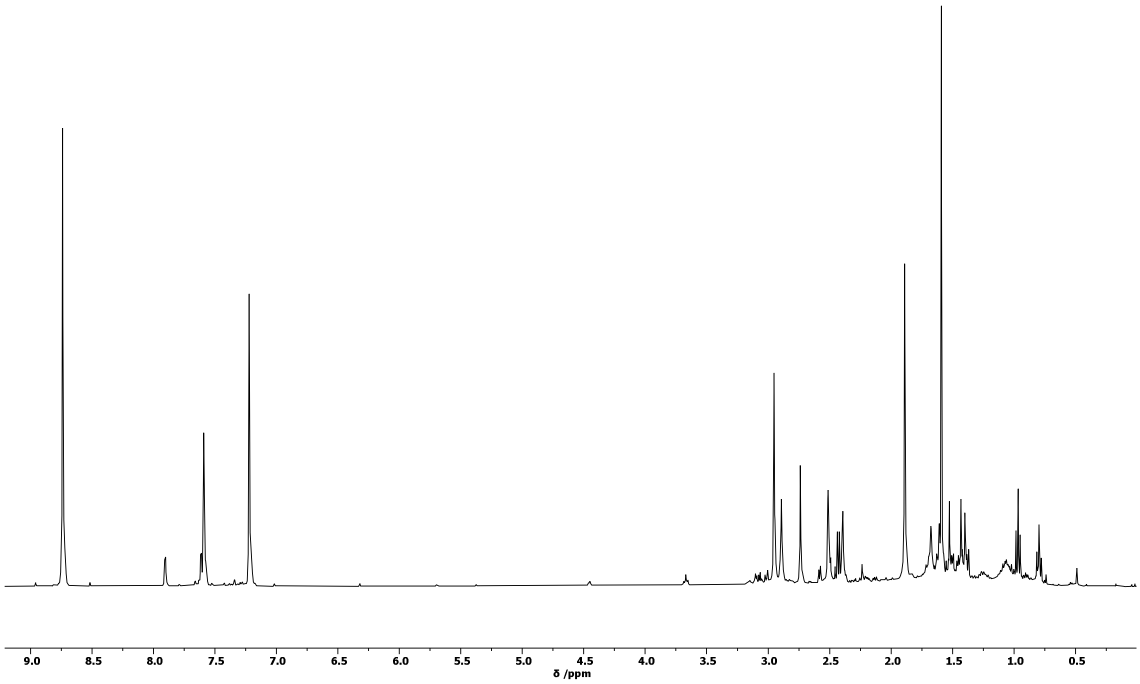

Figure S15 ^{1}H NMR spectrum of crude *rac*-$^{\mathrm{Me},{}^{n}\mathrm{Pr}}$SB($^{{}^{t}\mathrm{Bu}_2}$ArO,I*)$Li_2$ (**L4**) (pyridine-d_5, 400 MHz, 298 K).

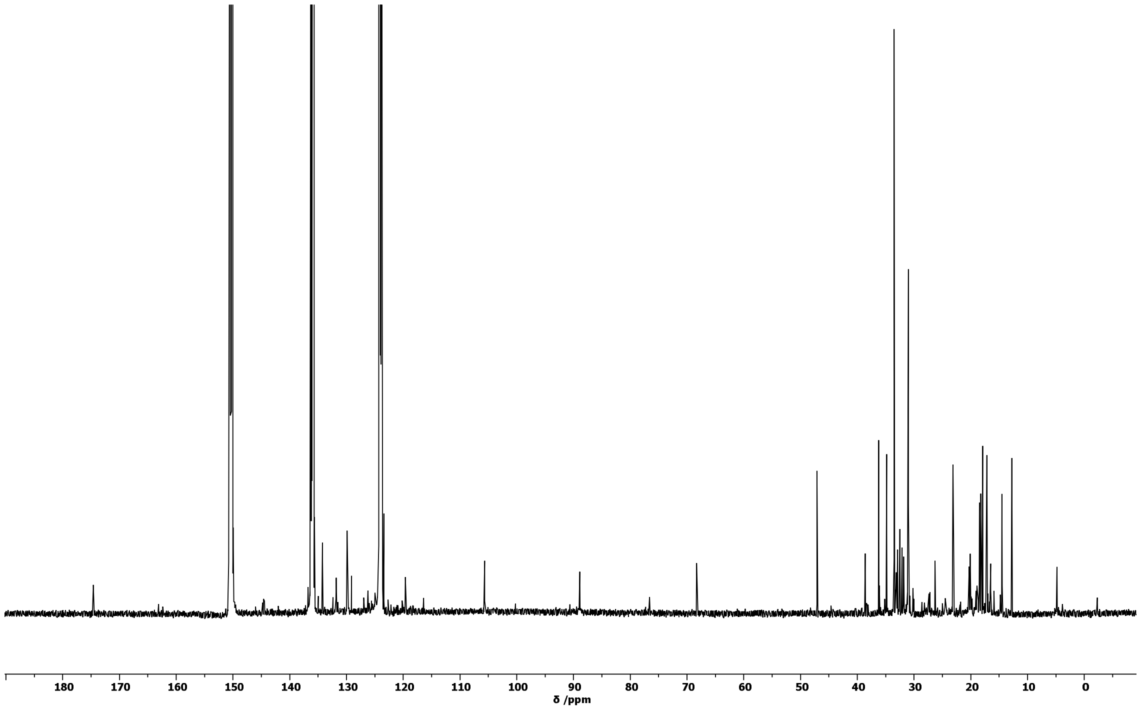

Figure S16 ^{13}C{^{1}H} NMR spectrum of crude *rac*-$^{\mathrm{Me},{}^{n}\mathrm{Pr}}$SB($^{{}^{t}\mathrm{Bu}_2}$ArO,I*)$Li_2$ (**L4**) (pyridine-d_5, 151 MHz, 298 K).

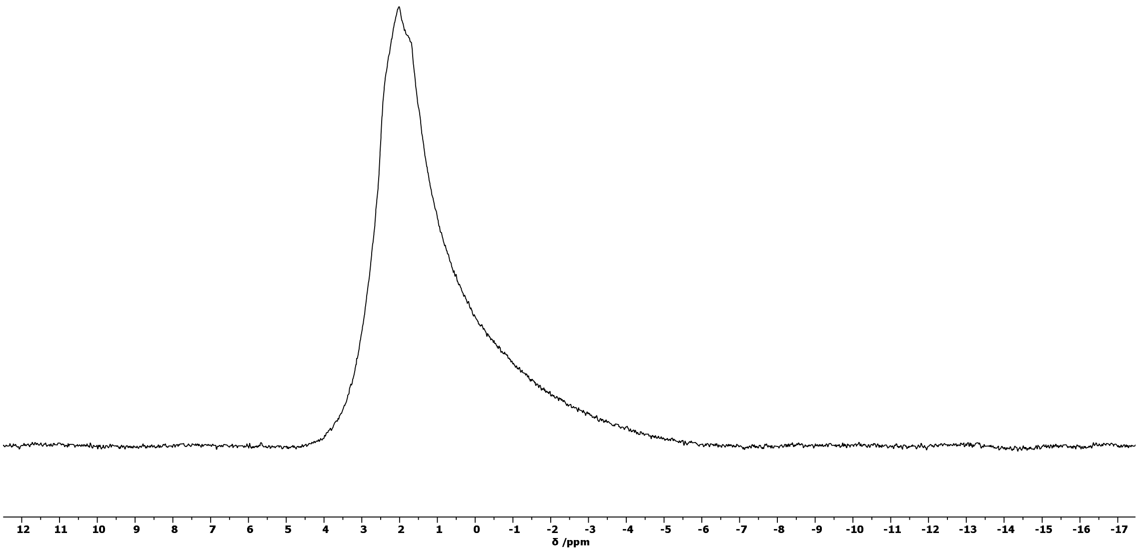

Figure S17 ^{7}Li NMR spectrum of crude *rac*-$^{\mathrm{Me},n\mathrm{Pr}}$SB($^{t\mathrm{Bu}_2}$ArO,I*)$\mathrm{Li}_2$ (**L4**) (pyridine-d_5, 156 MHz, 298 K).

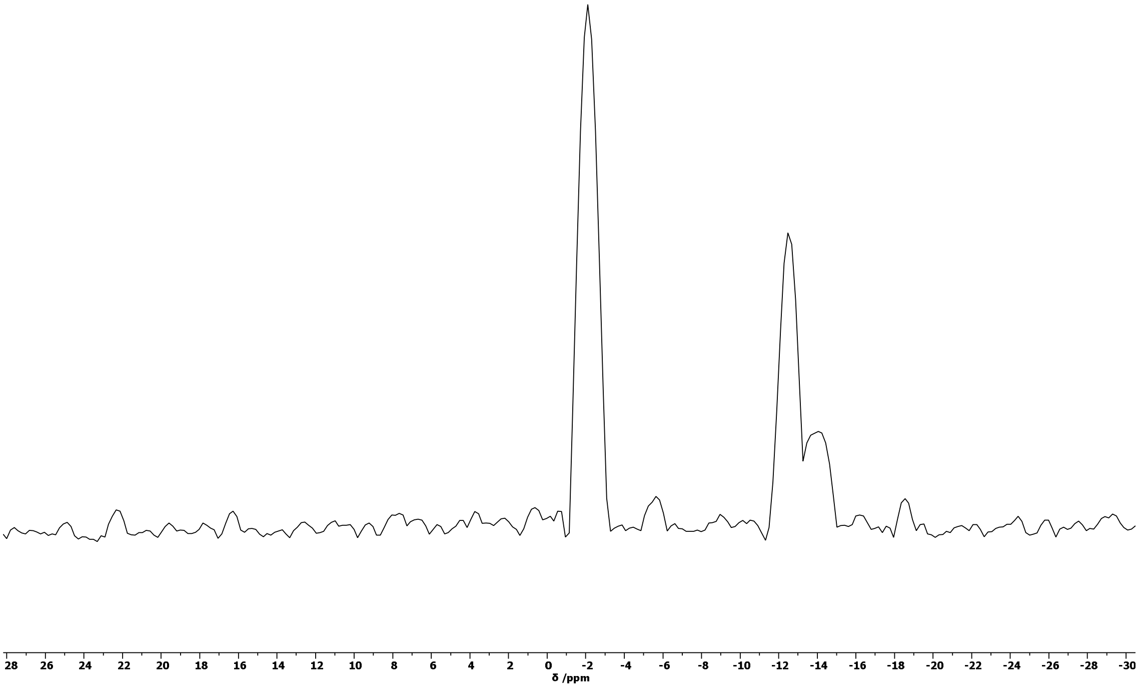

Figure S18 ^{29}Si NMR spectrum of crude *rac*-$^{\mathrm{Me},n\mathrm{Pr}}$SB($^{t\mathrm{Bu}_2}$ArO,I*)$\mathrm{Li}_2$ (**L4**) (^{1}H-observed HMBC, pyridine-d_5, 80 MHz, 298 K).

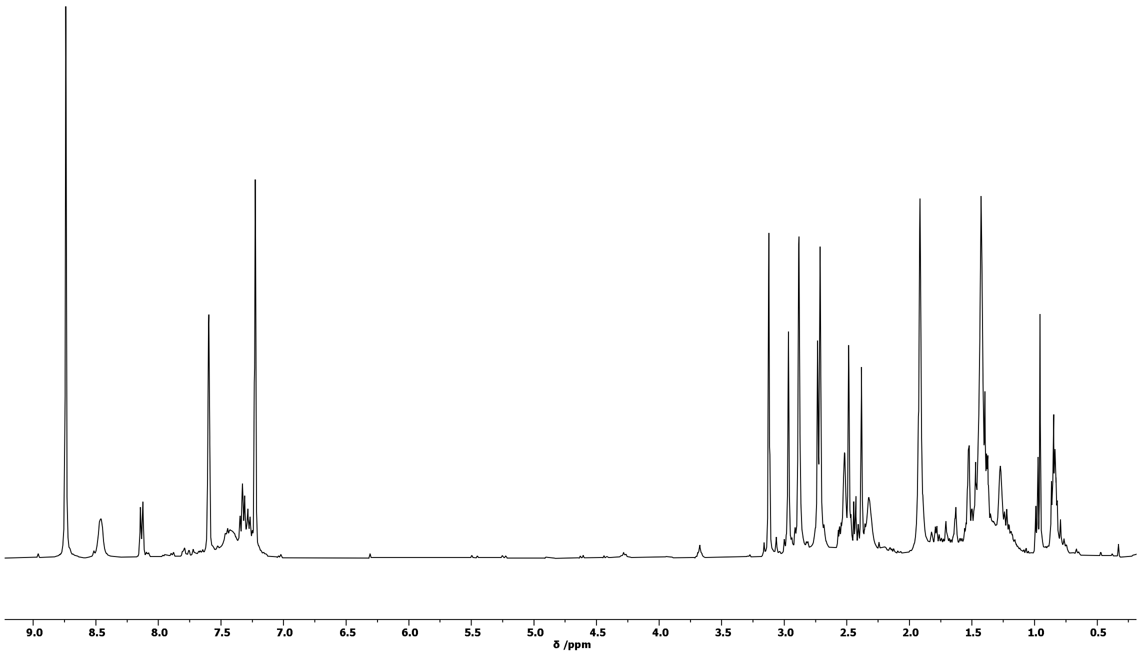

Figure S19 ^{1}H NMR spectrum of crude *rac*-^{Me,Ph}SB($^{{}^{t}Bu_2}$ArO,I*)Li_2 (**L5**) (pyridine-d_5, 400 MHz, 298 K).

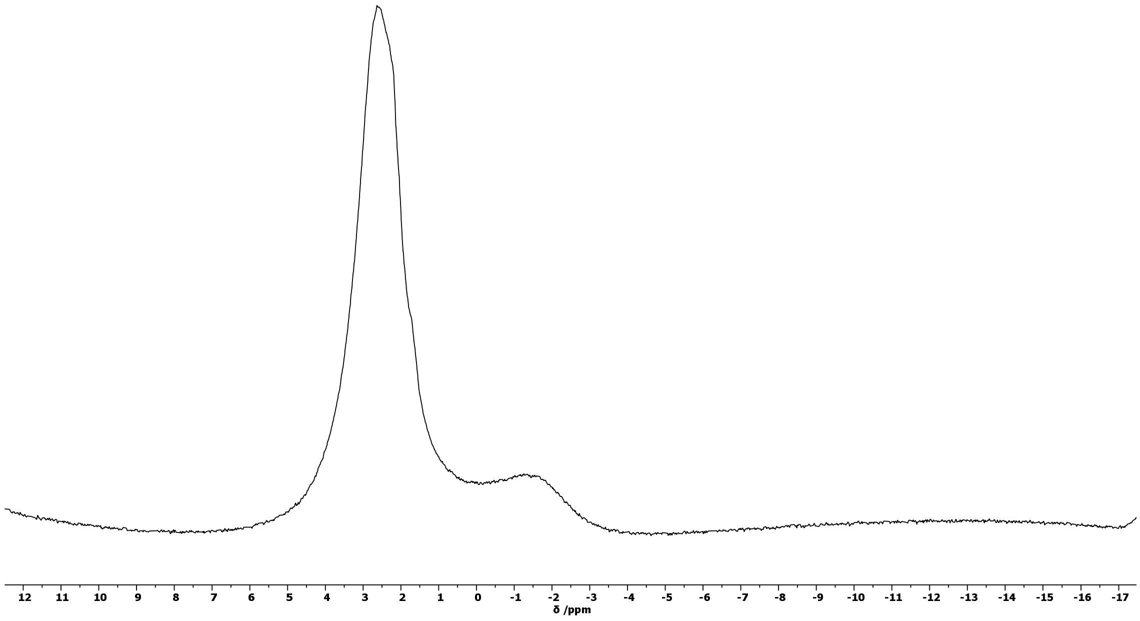

Figure S20 ^{7}Li NMR spectrum of crude *rac*-^{Me,Ph}SB($^{{}^{t}Bu_2}$ArO,I*)Li_2 (**L5**) (pyridine-d_5, 156 MHz, 298 K).

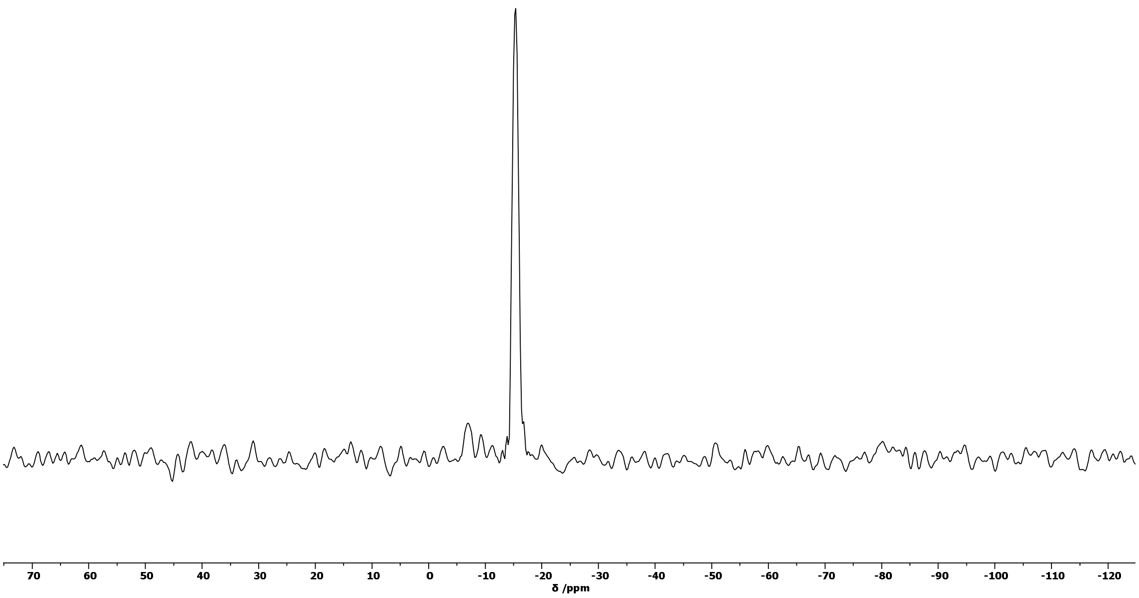

Figure S21 ^{29}Si NMR spectrum of crude *rac*-^{Me,Ph}SB(^{tBu_2}ArO,I*)Li_2 (**L5**) (^{1}H-observed HMBC, pyridine-d_5, 80 MHz, 298 K).

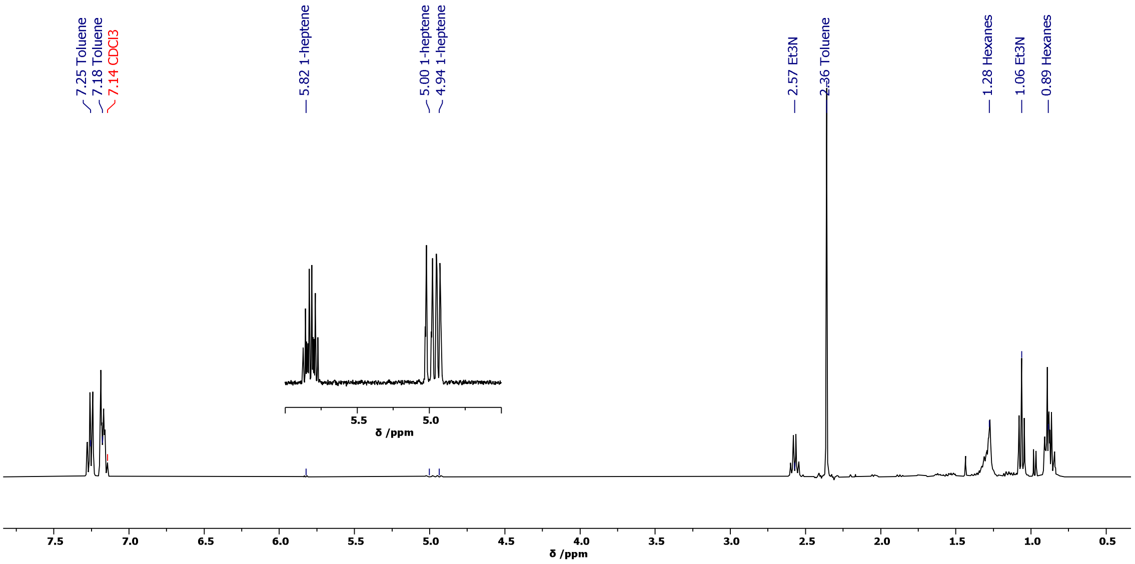

Figure S22 ^{1}H NMR spectrum (chloroform-*d*, 400 MHz, 298 K) of distillate following removal of volatiles from synthesis of **L2**. Resonances corresponding to 1-heptene and NEt_3 can be clearly identified.

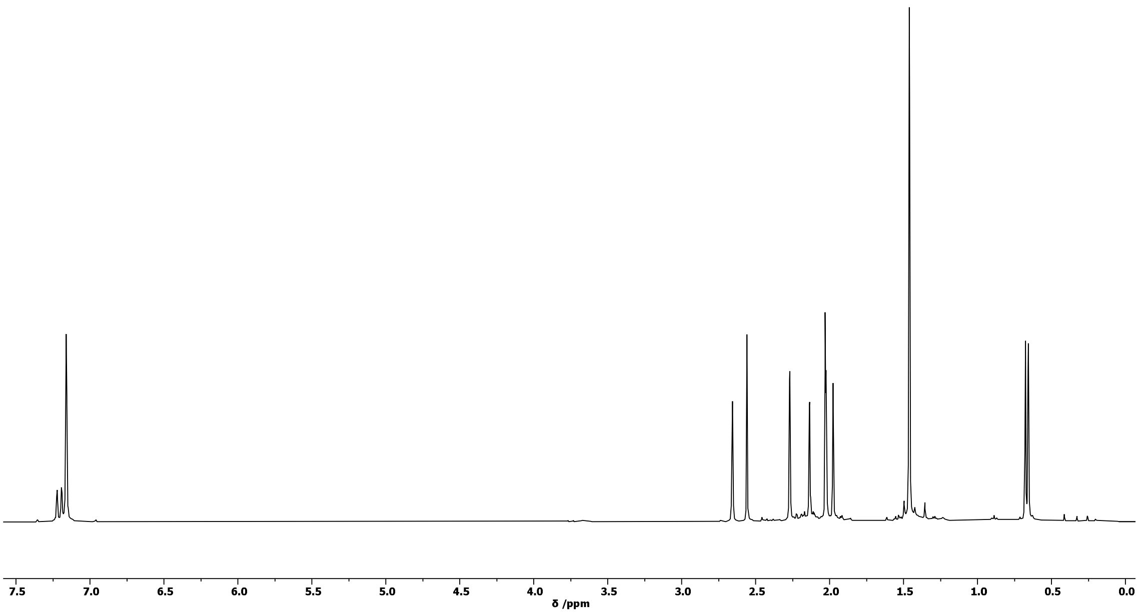

Figure S23 ^{1}H NMR spectrum of $^{Me_2}SB(^{tBu,Me}ArO,I^*)TiCl_2$ (**1**) (benzene-d_6, 400 MHz, 298 K).

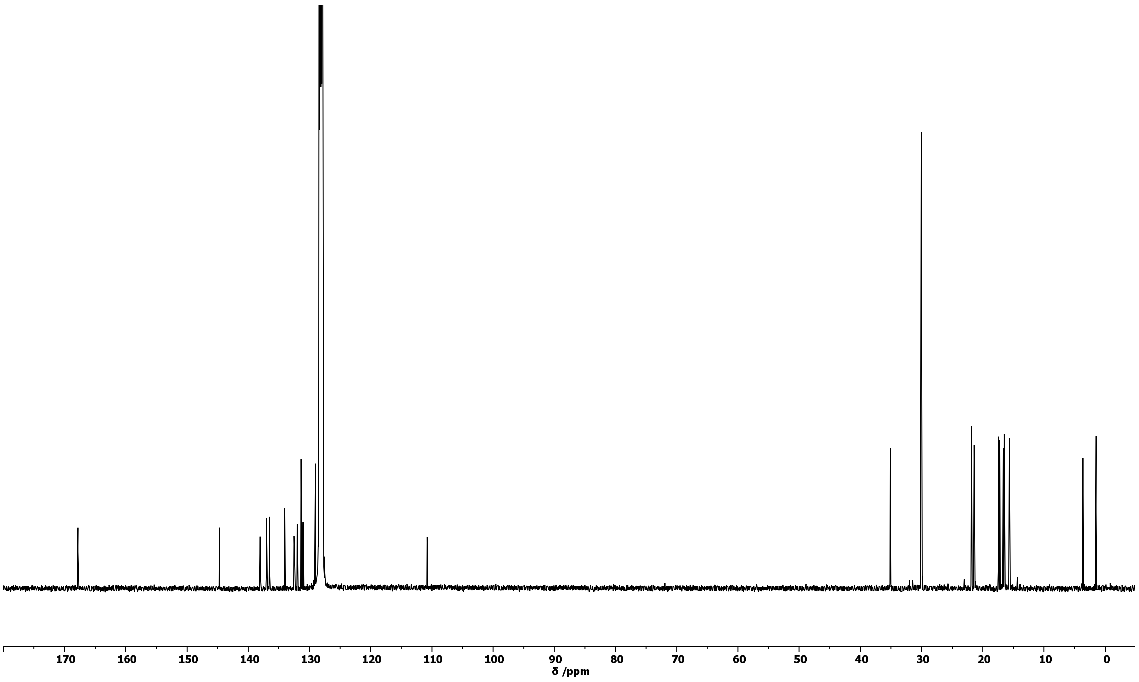

Figure S24 $^{13}C\{^{1}H\}$ NMR spectrum of $^{Me_2}SB(^{tBu,Me}ArO,I^*)TiCl_2$ (**1**) (benzene-d_6, 126 MHz, 298 K).

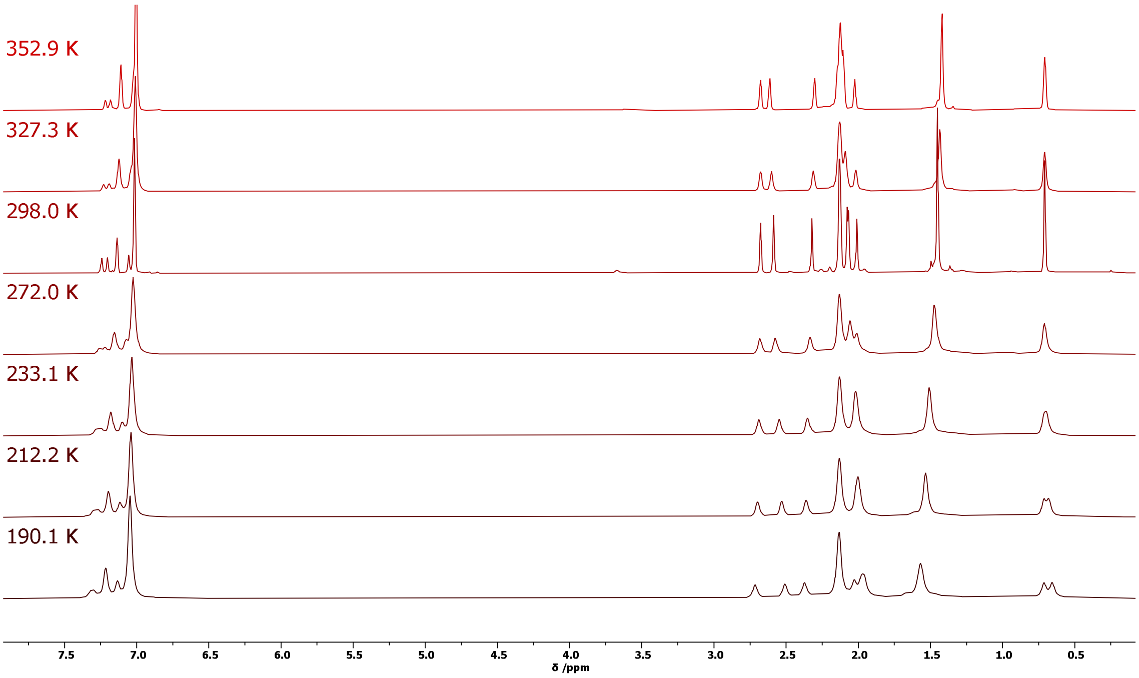

Figure S25 ^{1}H VT NMR spectra of ^{Me2}SB(^{tBu,Me}ArO,I*)$TiCl_2$ (**1**) as a function of temperature (toluene-d_8, 500 MHz, 193–353 K).

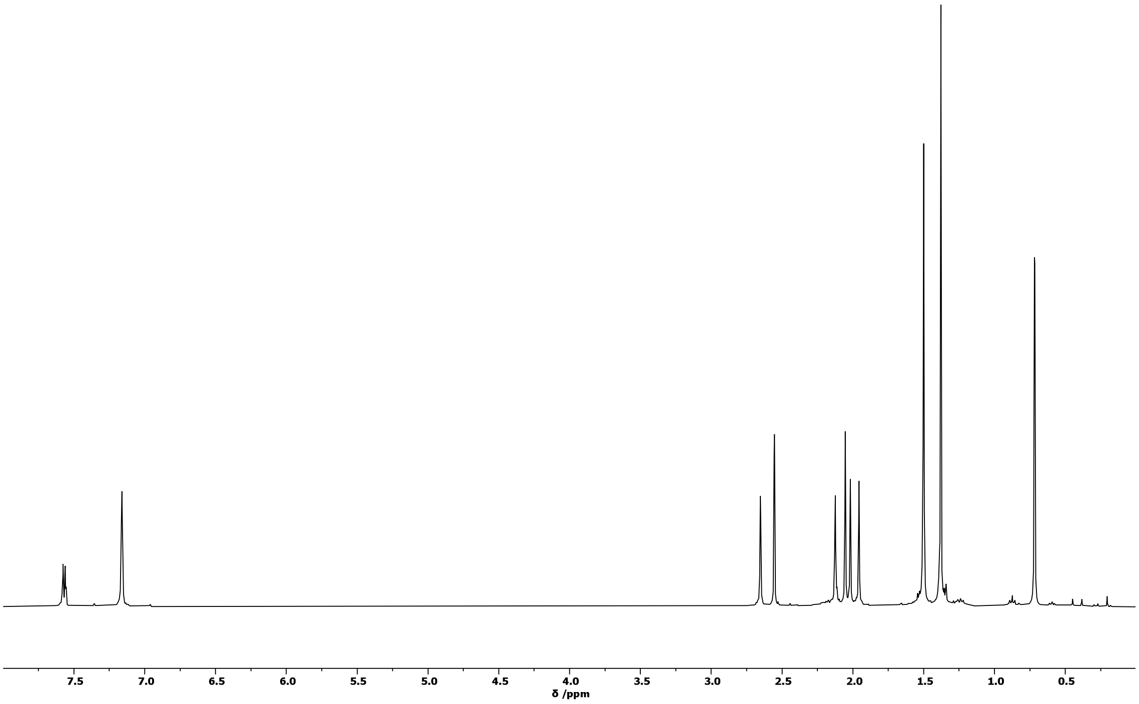

Figure S26 ^{1}H NMR spectrum of ^{Me2}SB(^{tBu2}ArO,I*)$TiCl_2$ (**2**) (benzene-d_6, 400 MHz, 298 K).

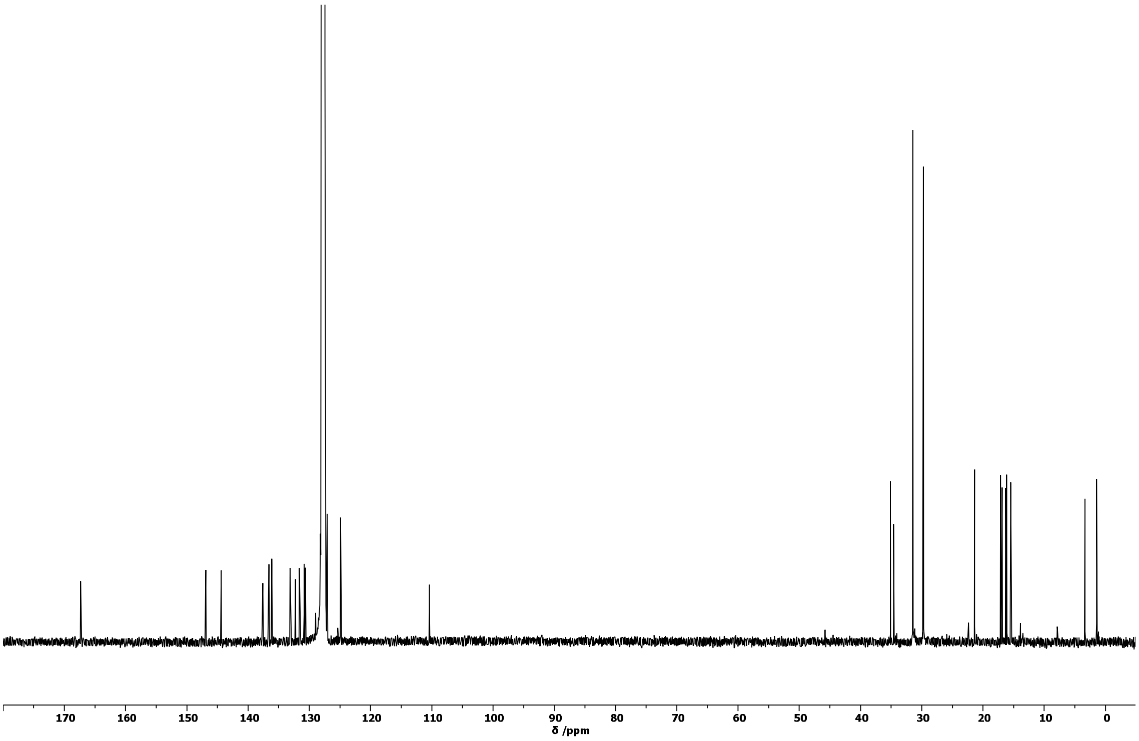

Figure S27 $^{13}C\{^{1}H\}$ NMR spectrum of ^{Me_2}SB($^{t\mathrm{Bu}_2}$ArO,I*)TiCl$_2$ (**2**) (benzene-d_6, 126 MHz, 298 K).

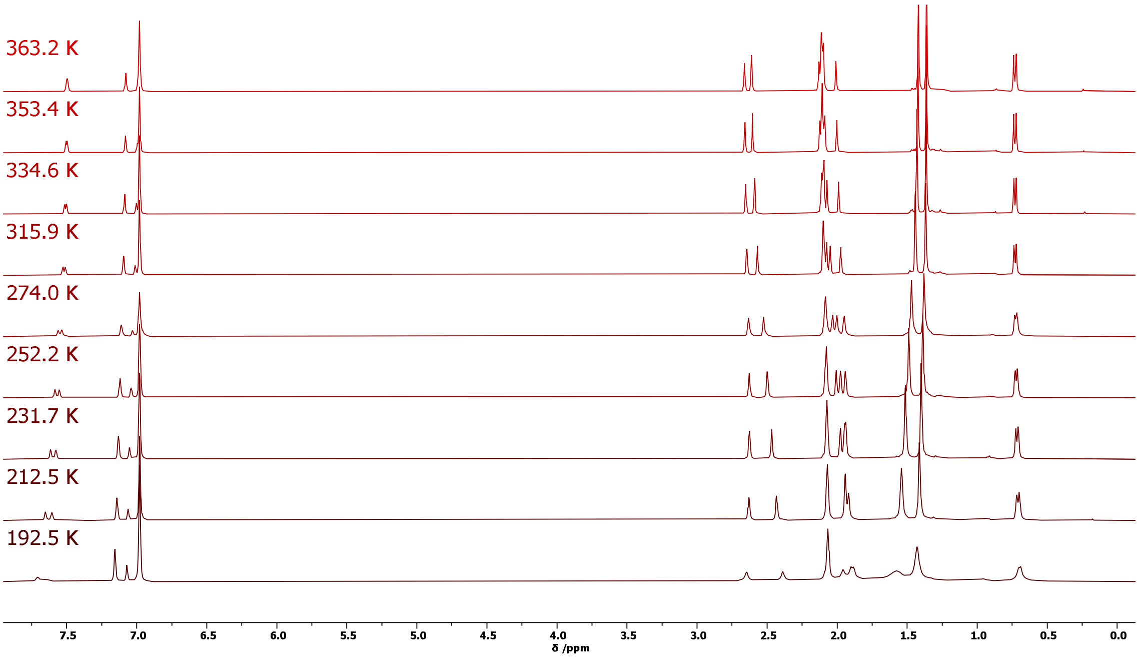

Figure S28 ^{1}H VT NMR spectra of ^{Me_2}SB($^{t\mathrm{Bu}_2}$ArO,I*)TiCl$_2$ (**2**) as a function of temperature (toluene-d_8, 500 MHz, 193–363 K).

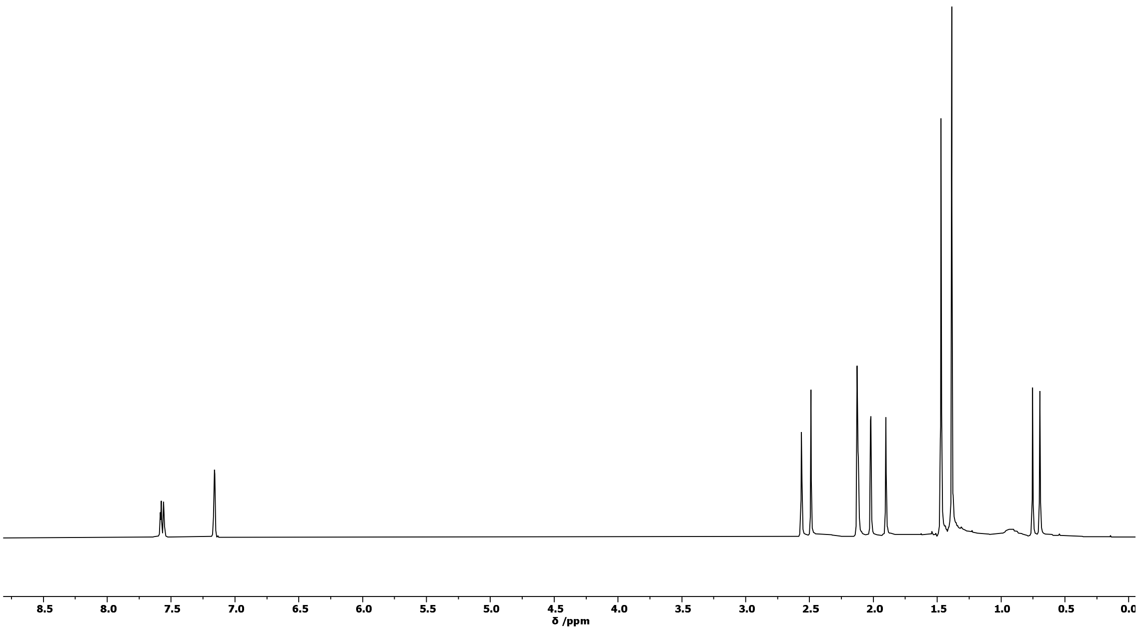

Figure S29 ^{1}H NMR spectrum of ^{Me_2}SB(^{tBu_2}ArO,I*)$ZrCl_2$ (**3**) (benzene-d_6, 400 MHz, 298 K).

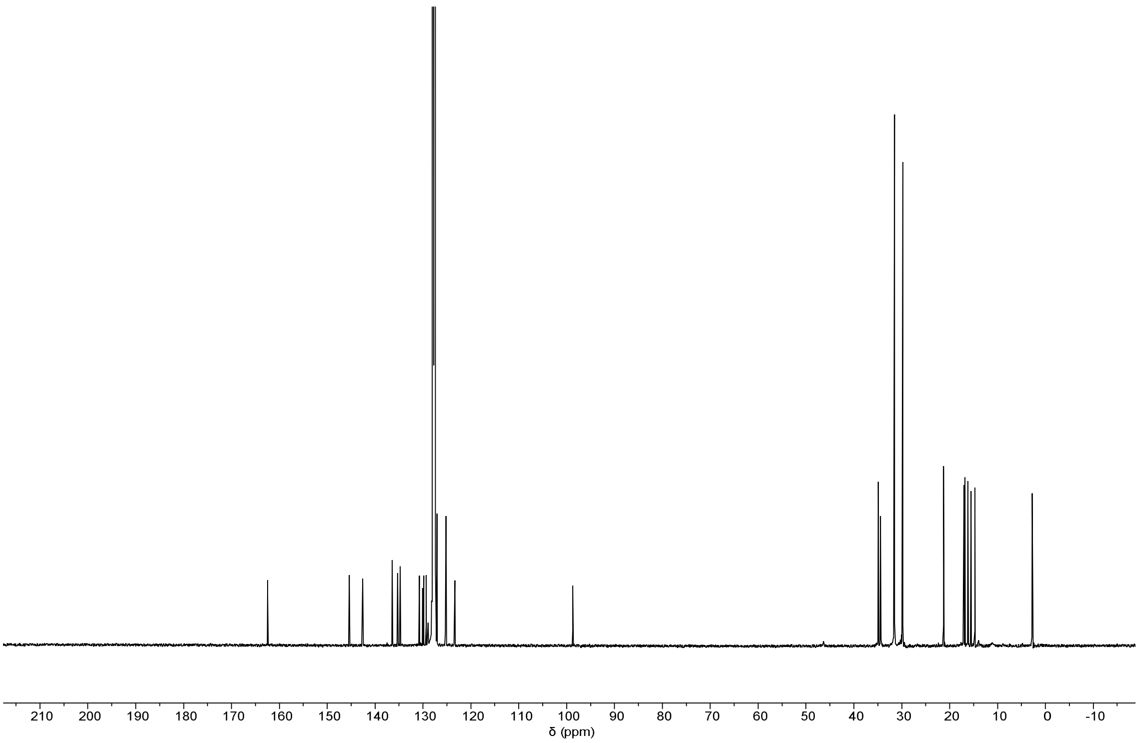

Figure S30 $^{13}C\{^{1}H\}$ NMR spectrum of ^{Me_2}SB(^{tBu_2}ArO,I*)$ZrCl_2$ (**3**) (benzene-d_6, 126 MHz, 298 K).

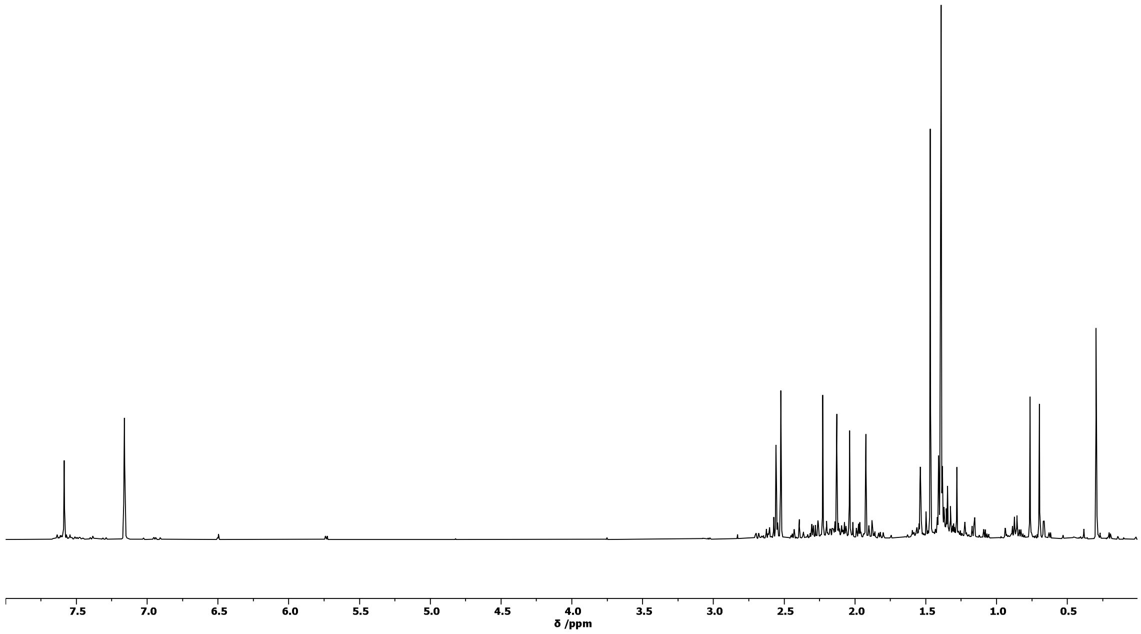

Figure S31 ^{1}H NMR spectrum of ^{Me2}SB(^{tBu2}ArO,I*)$HfCl_2$ (**4**) (benzene-d_6, 600 MHz, 298 K).

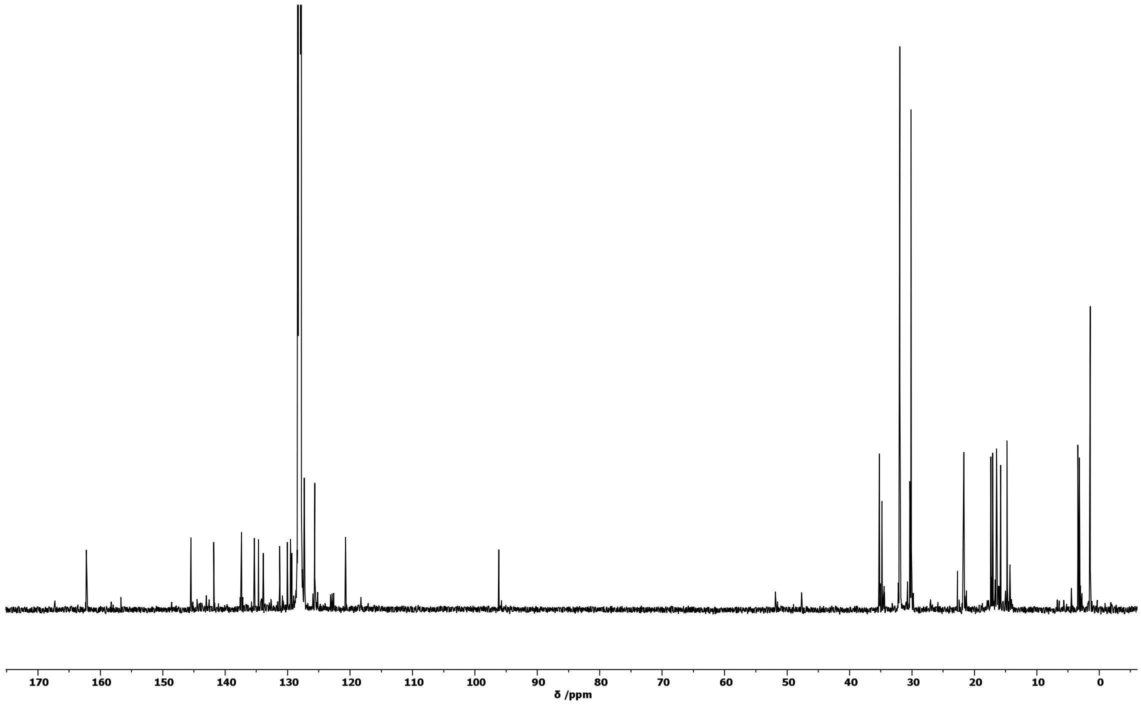

Figure S32 $^{13}C\{^{1}H\}$ NMR spectrum of ^{Me2}SB(^{tBu2}ArO,I*)$HfCl_2$ (**4**) (benzene-d_6, 151 MHz, 298 K).

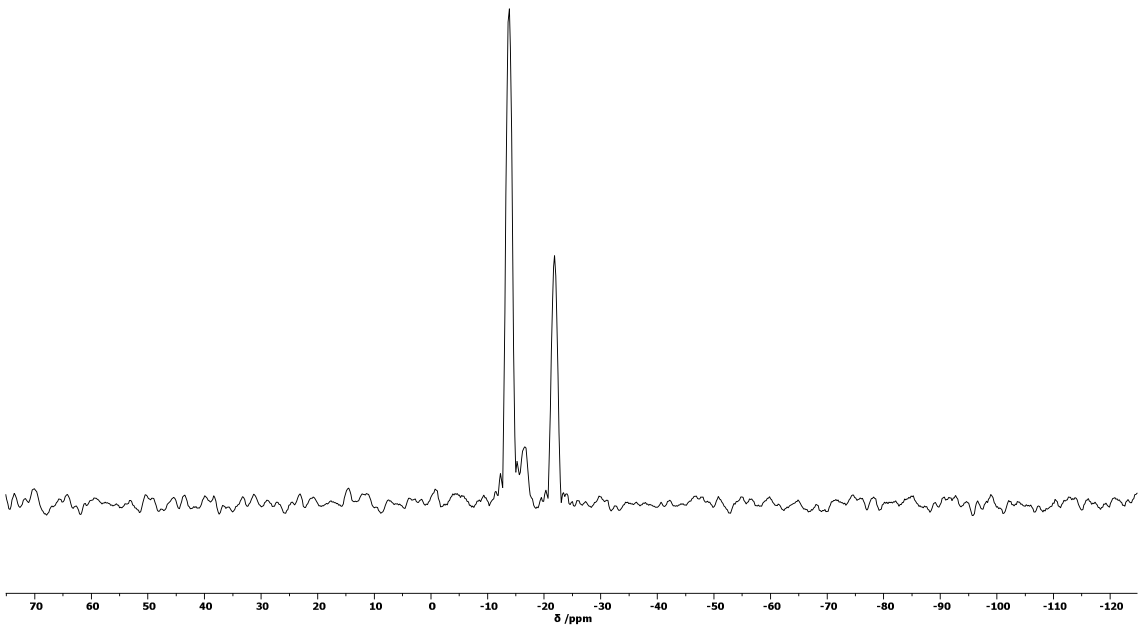

Figure S33 ^{29}Si NMR spectrum of ^{Me_2}SB($^{^tBu_2}$ArO,I*)HfCl$_2$ (**4**) (^{1}H-observed HMBC, benzene-d_6, 80 MHz, 298 K).

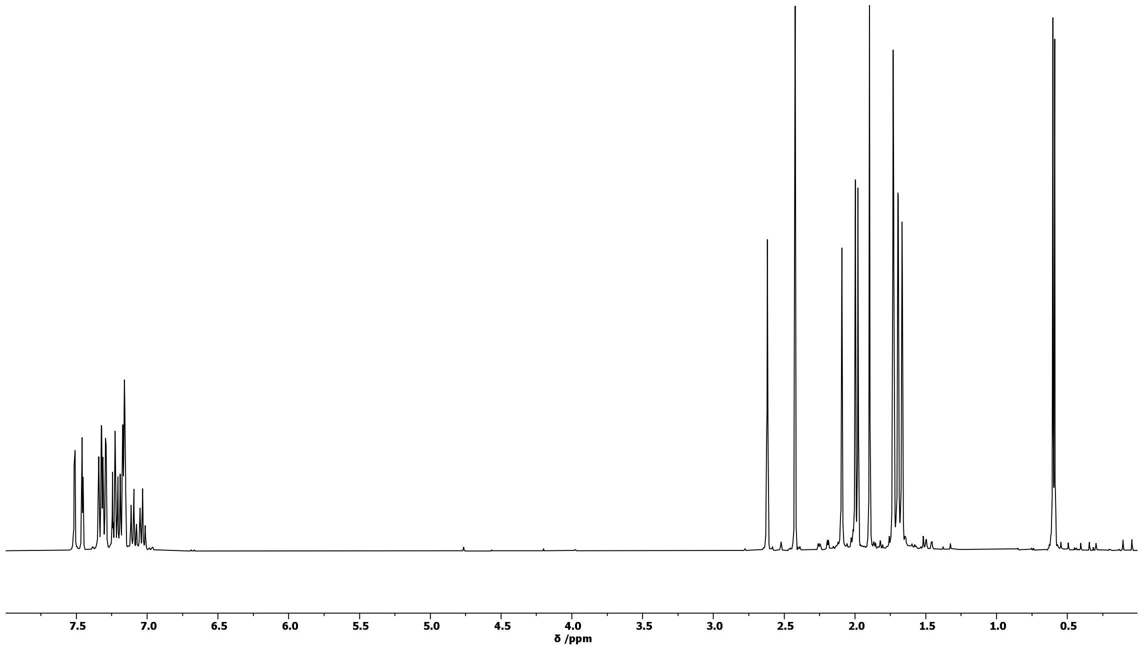

Figure S34 ^{1}H NMR spectrum of ^{Me_2}SB(^{Cumyl_2}ArO,I*)TiCl$_2$ (**5**) (benzene-d_6, 400 MHz, 298 K).

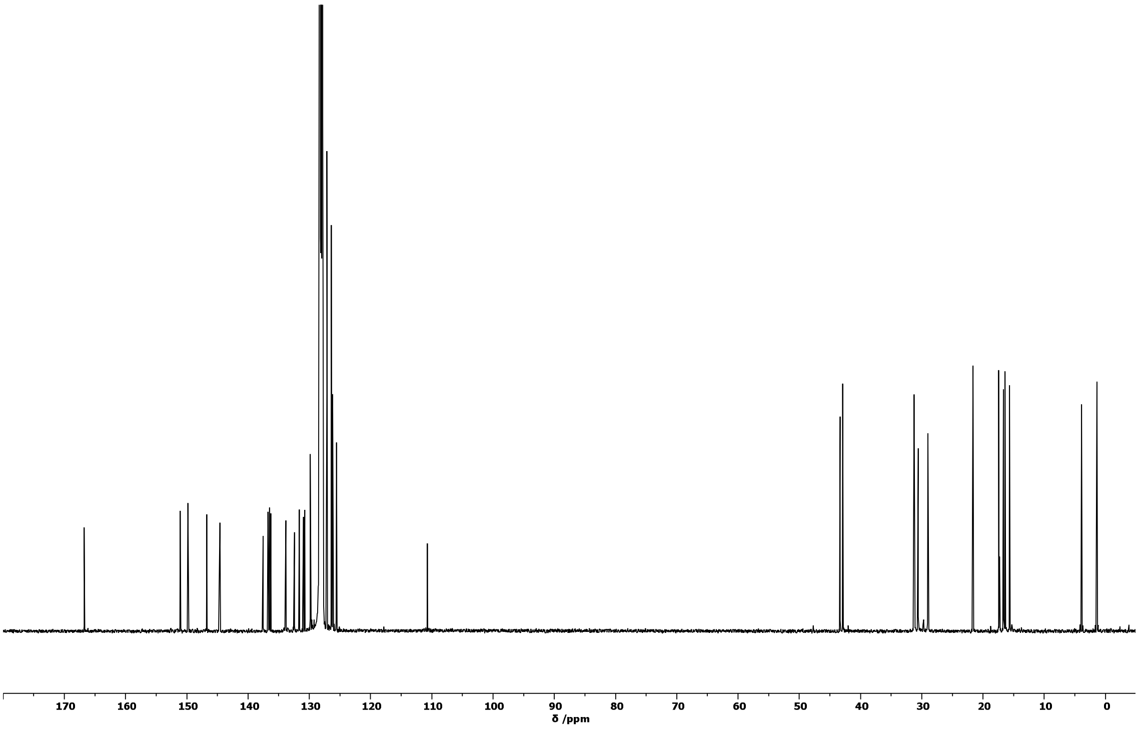

Figure S35 $^{13}C\{^{1}H\}$ NMR spectrum of $^{Me2}SB(^{Cumyl2}ArO,I^*)TiCl_2$ (**5**) (benzene-d_6, 126 MHz, 298 K).

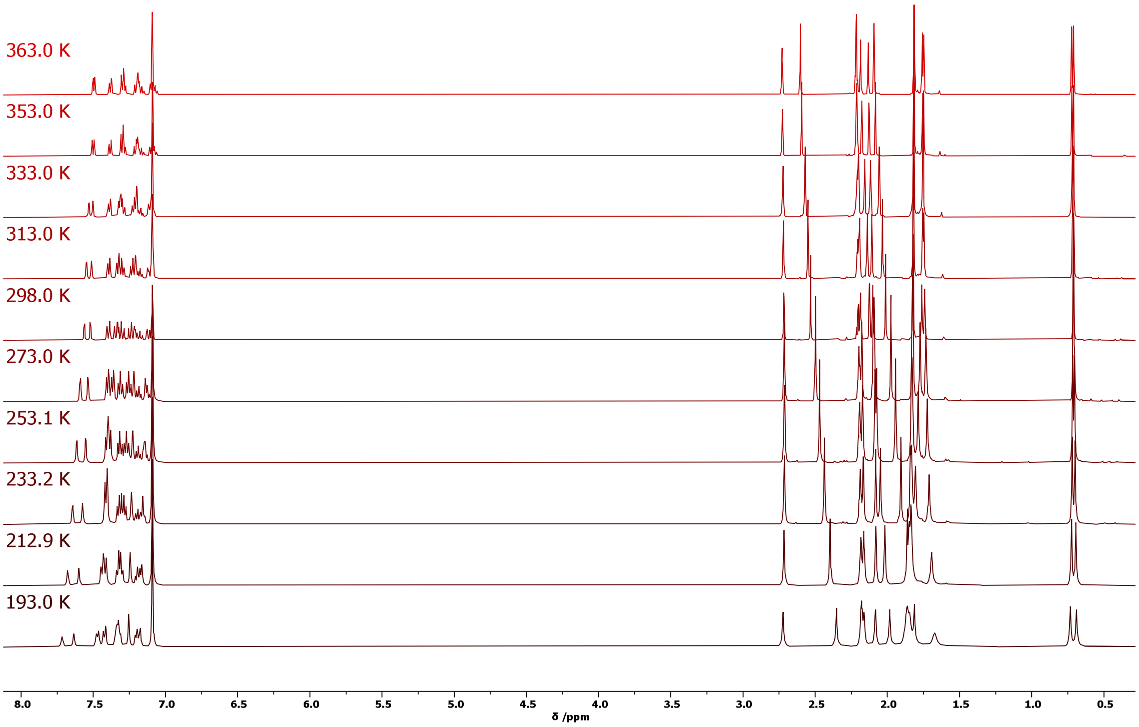

Figure S36 ^{1}H VT NMR spectra of $^{Me2}SB(^{Cumyl2}ArO,I^*)TiCl_2$ (**5**) as a function of temperature (toluene-d_8, 500 MHz, 193–363 K).

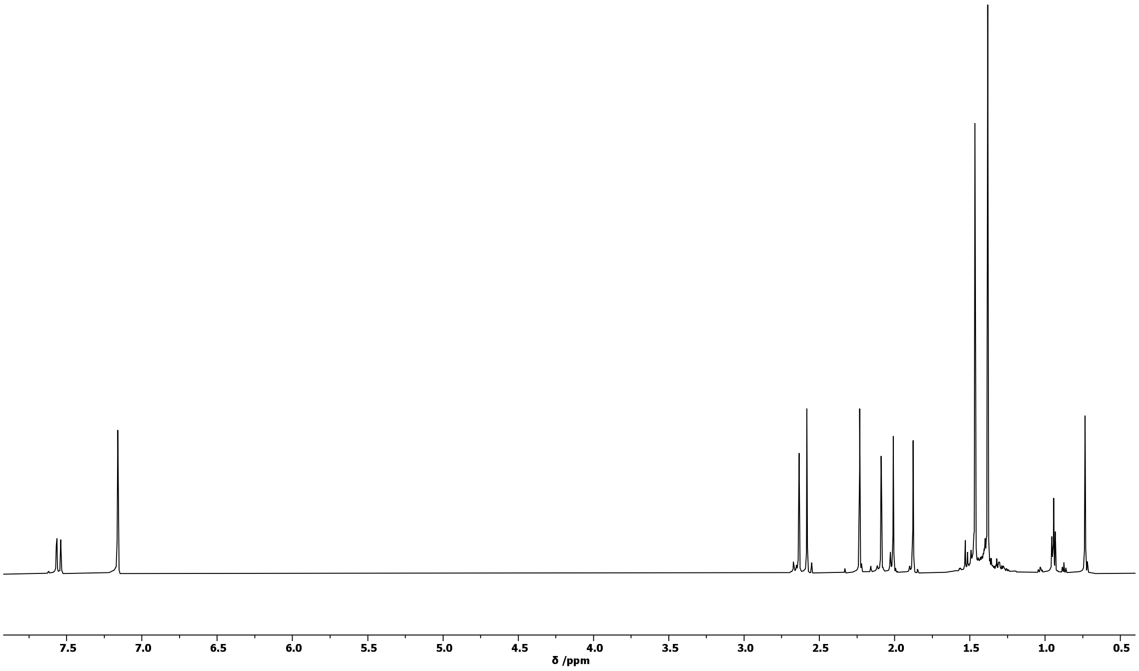

Figure S37 ^{1}H NMR spectrum of *rac*-^{Me,nPr}SB(^{tBu_2}ArO,I*)$TiCl_2$ (**6**) (benzene-d_6, 600 MHz, 298 K).

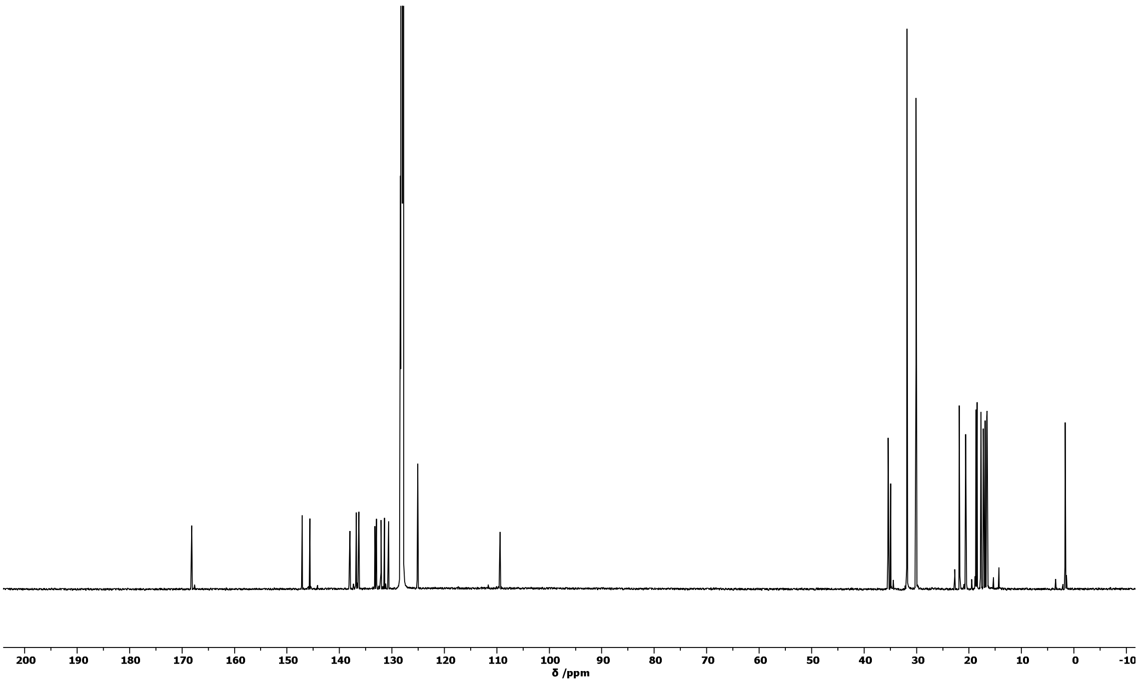

Figure S38 $^{13}C\{^{1}H\}$ NMR spectrum of *rac*-^{Me,nPr}SB(^{tBu_2}ArO,I*)$TiCl_2$ (**6**) (benzene-d_6, 151 MHz, 298 K).

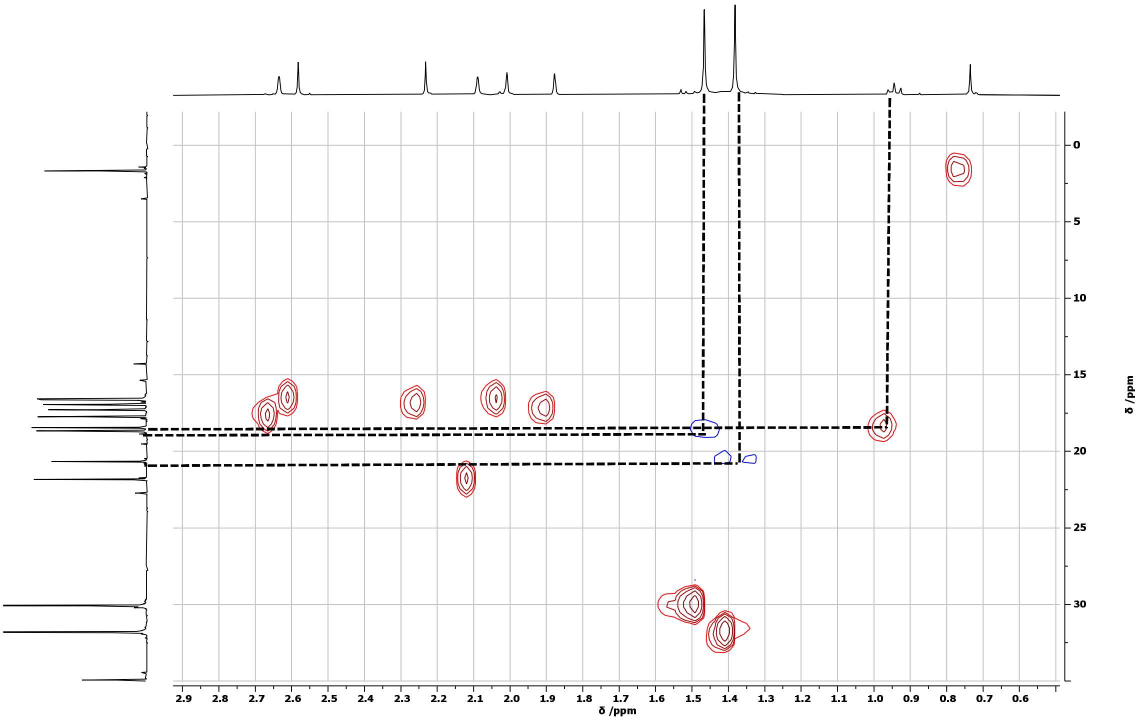

Figure S39 ^{1}H–^{13}C HSQC NMR spectrum of *rac*-$^{Me,{}^{n}Pr}$SB($^{{}^{t}Bu_{2}}$ArO,I*)$TiCl_2$ (**6**) (benzene-d_6, 400 MHz, 298 K), with resonances corresponding to $SiCH_2CH_2CH_3$ highlighted and distinct from the $C(CH_3)_3$ resonances.

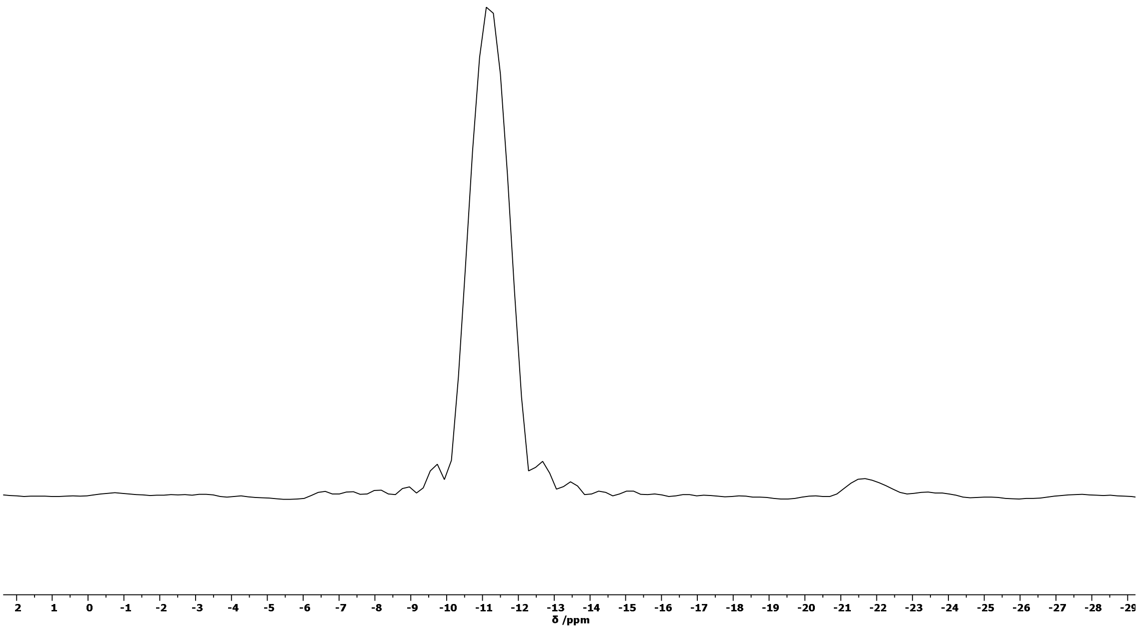

Figure S40 ^{29}Si NMR spectrum of *rac*-$^{Me,{}^{n}Pr}$SB($^{{}^{t}Bu_{2}}$ArO,I*)$TiCl_2$ (**6**) (^{1}H-observed HMBC, benzene-d_6, 80 MHz, 298 K).

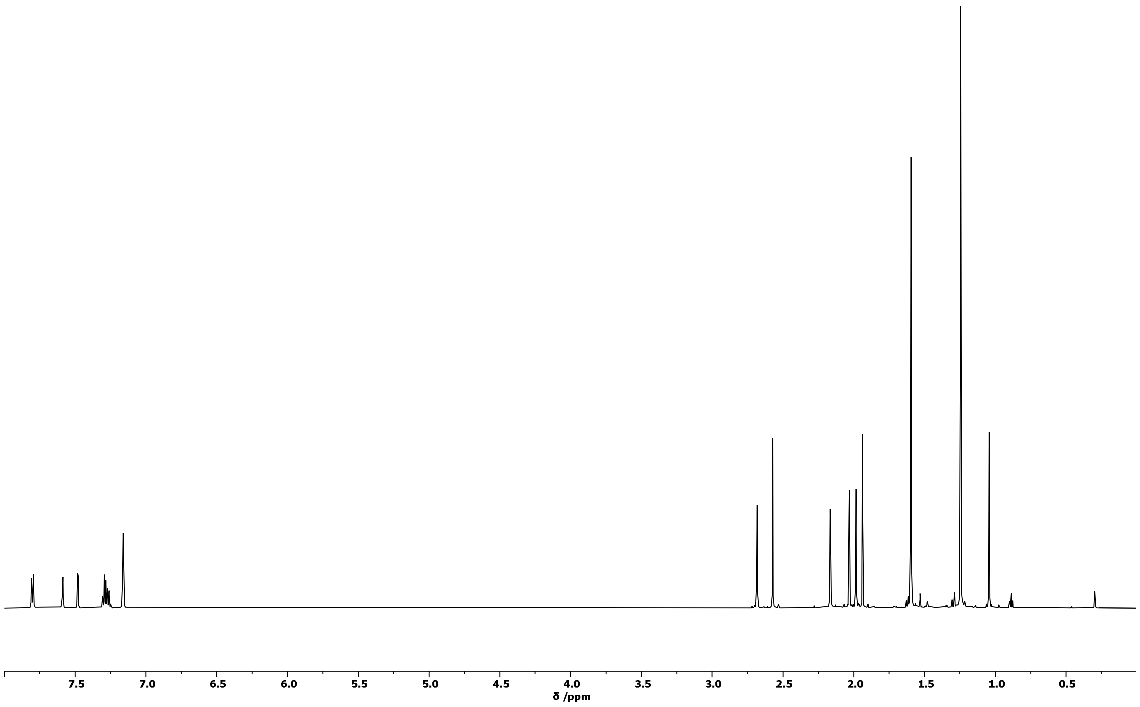

Figure S41 ^{1}H NMR spectrum of *rac*-^{Me,Ph}SB($^{^{t}Bu_2}$ArO,I*)TiCl$_2$ (**7**) (benzene-d_6, 600 MHz, 298 K).

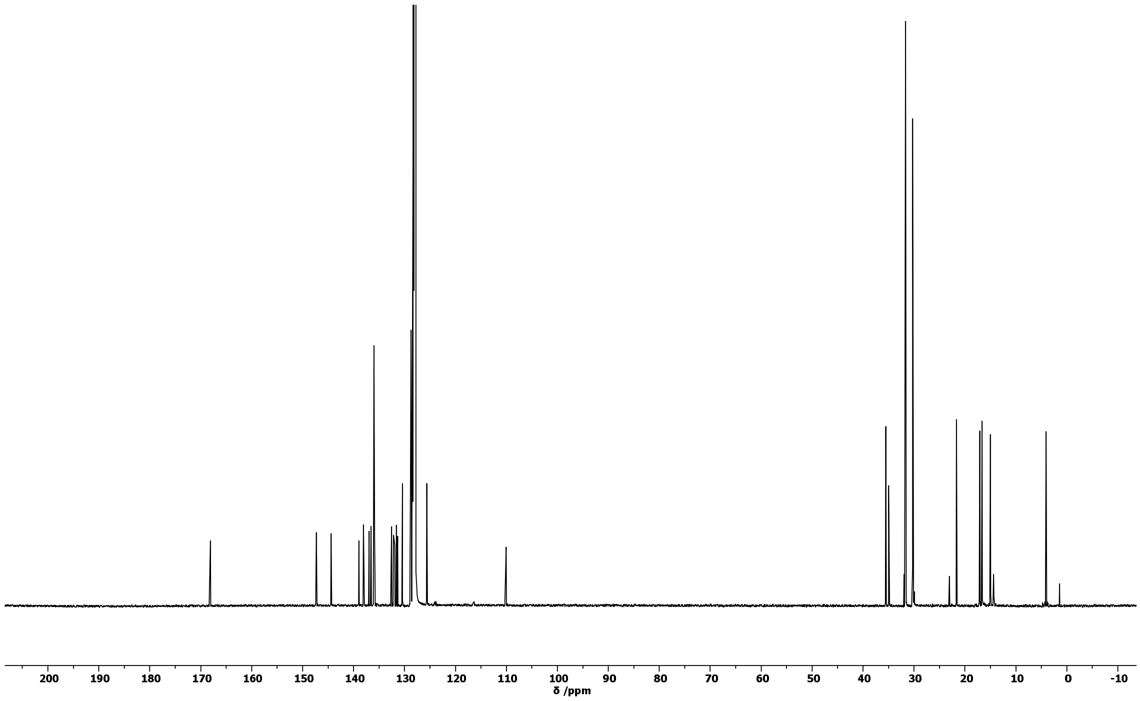

Figure S42 ^{13}C{^{1}H} NMR spectrum of *rac*-^{Me,Ph}SB($^{^{t}Bu_2}$ArO,I*)TiCl$_2$ (**7**) (benzene-d_6, 151 MHz, 298 K).

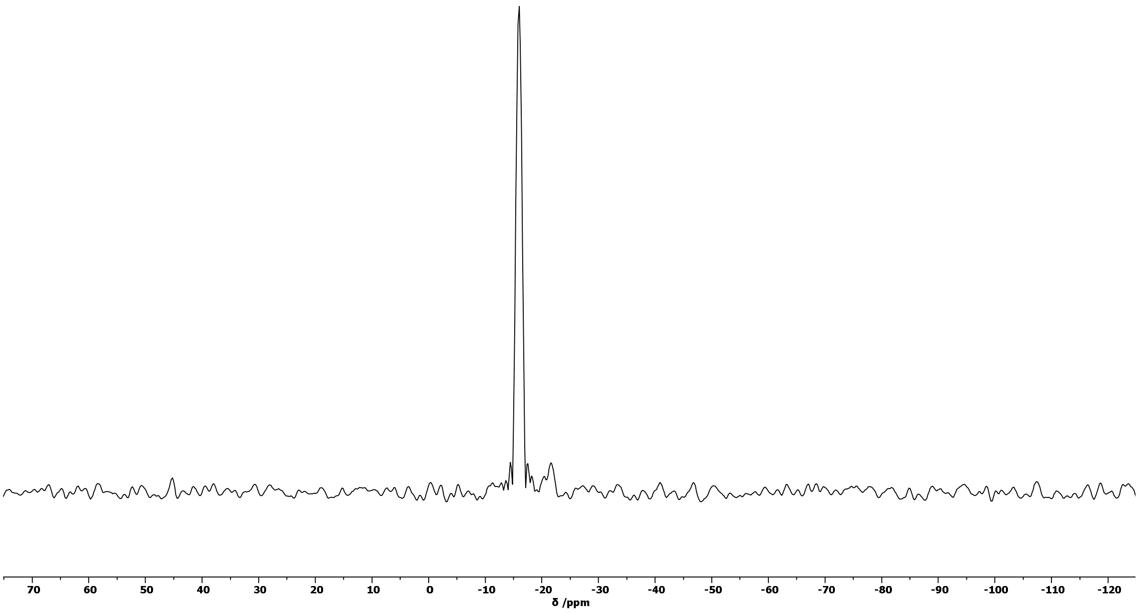

Figure S43 ^{29}Si NMR spectrum of *rac*-^{Me,Ph}SB($^{t\mathrm{Bu}_2}$ArO,I*)$TiCl_2$ (**7**) (^{1}H-observed HMBC, benzene-d_6, 80 MHz, 298 K).

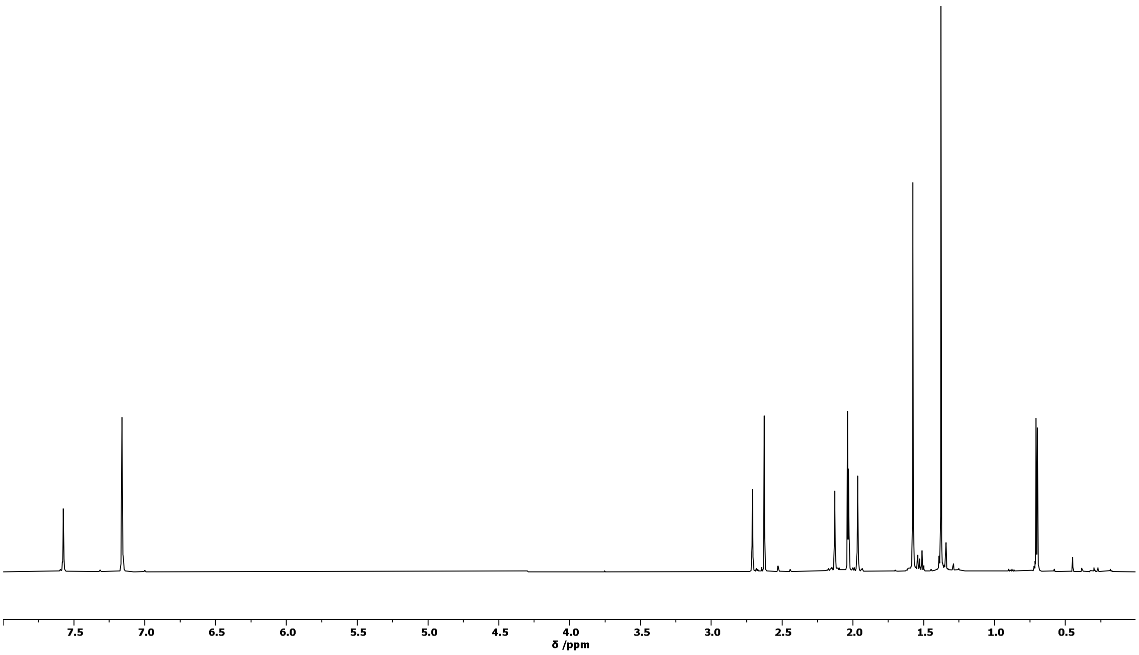

Figure S44 ^{1}H NMR spectrum of ^{Me_2}SB($^{t\mathrm{Bu}_2}$ArO,I*)$TiBr_2$ (**8**) (benzene-d_6, 500 MHz, 298 K).

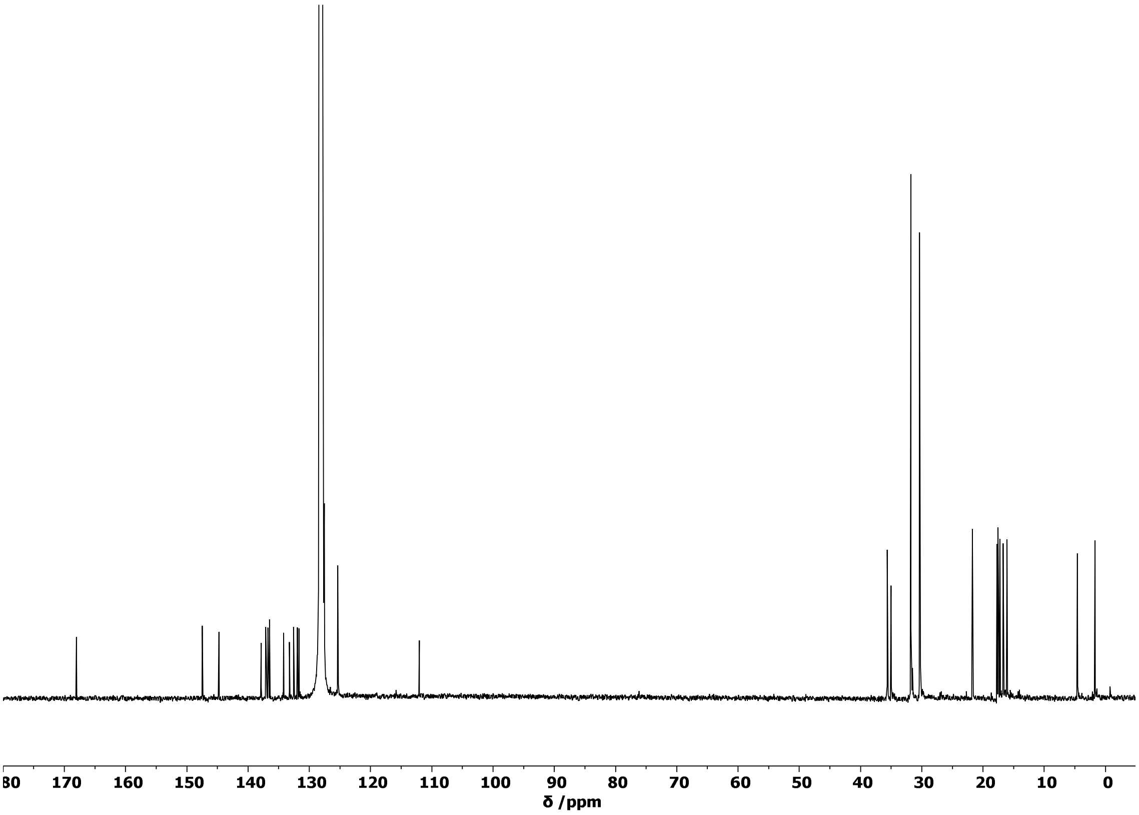

Figure S45 $^{13}C\{^{1}H\}$ NMR spectrum of ^{Me_2}SB($^{t\mathrm{Bu}_2}$ArO,I*)$TiBr_2$ (**8**) (benzene-d_6, 126 MHz, 298 K).

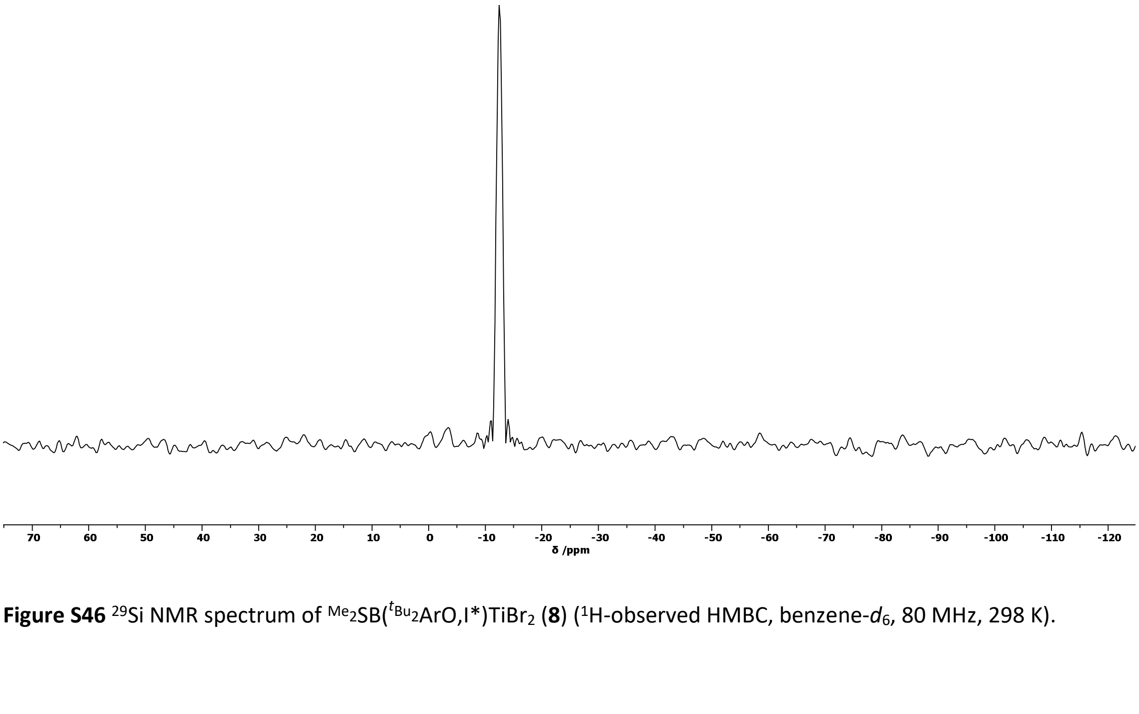

Figure S46 ^{29}Si NMR spectrum of ^{Me_2}SB($^{t\mathrm{Bu}_2}$ArO,I*)$TiBr_2$ (**8**) (^{1}H-observed HMBC, benzene-d_6, 80 MHz, 298 K).

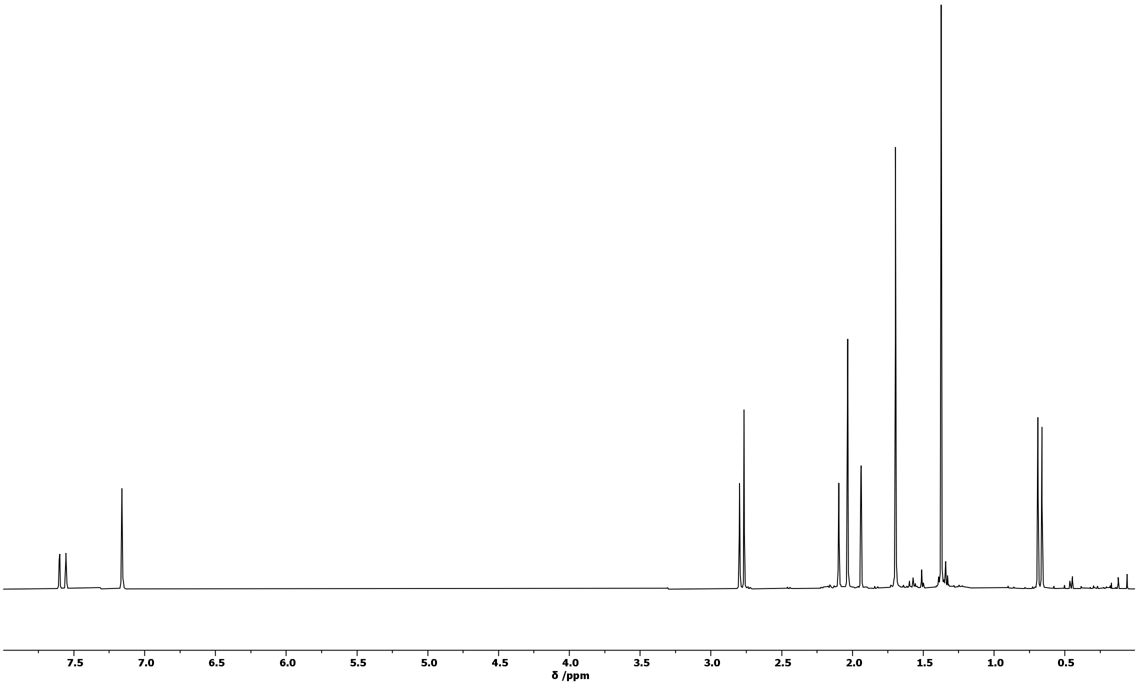

Figure S47 ^{1}H NMR spectrum of ^{Me2}SB($^{t\mathrm{Bu}2}$ArO,I*)TiI_2 (**9**) (benzene-d_6, 500 MHz, 298 K).

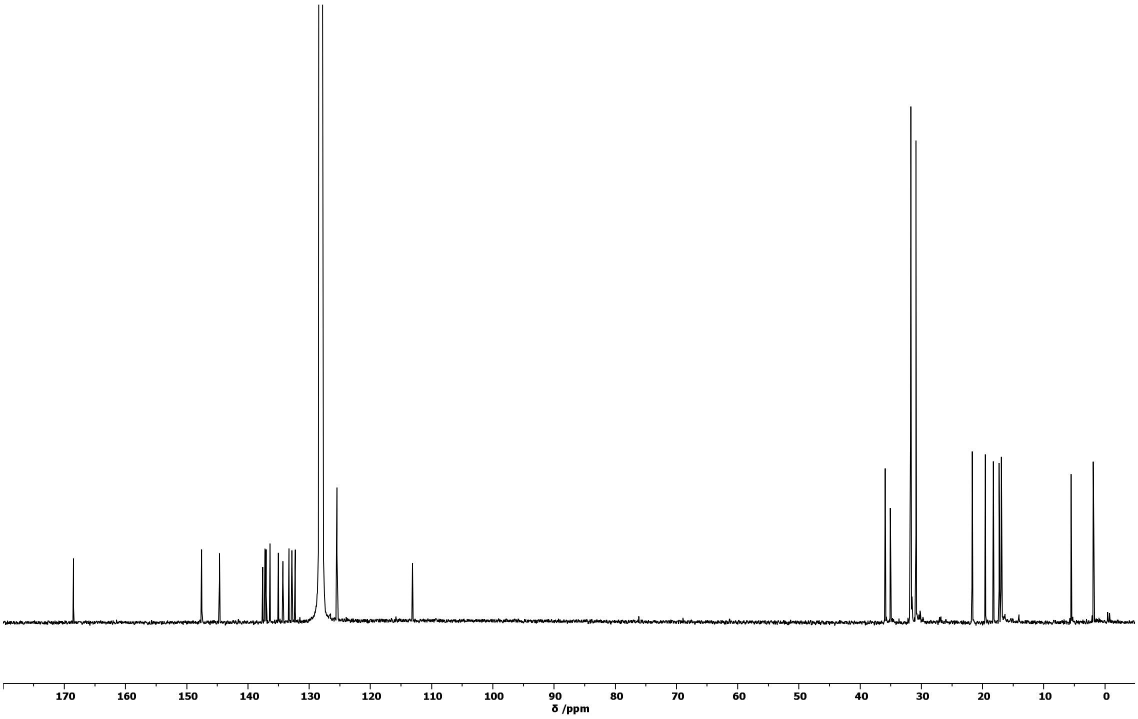

Figure S48 $^{13}C\{^{1}H\}$ NMR spectrum of ^{Me2}SB($^{t\mathrm{Bu}2}$ArO,I*)TiI_2 (**9**) (benzene-d_6, 126 MHz, 298 K).

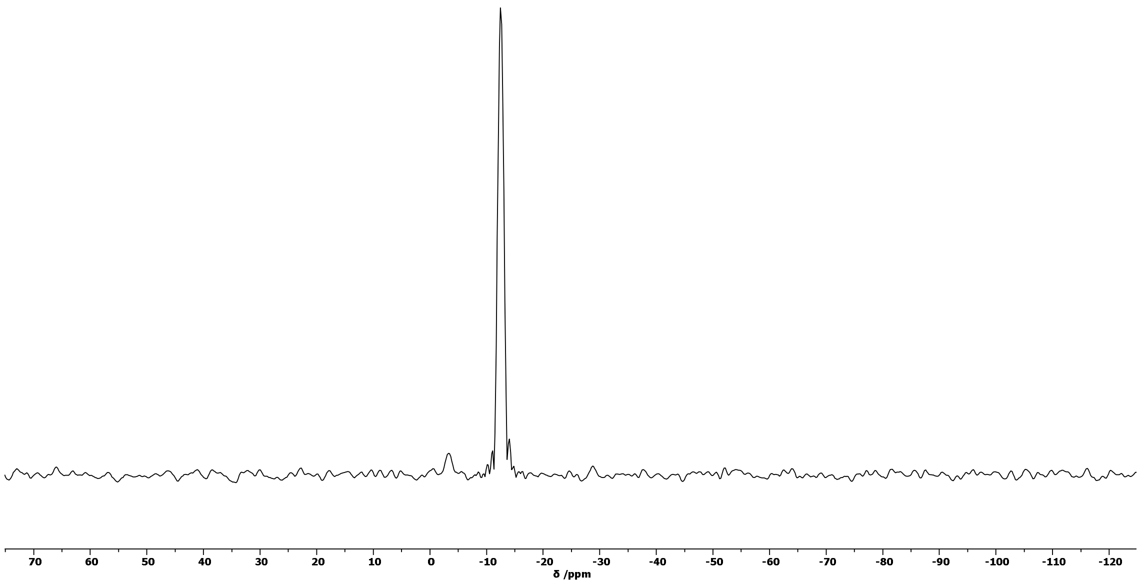

Figure S49 ^{29}Si NMR spectrum of ^{Me_2}SB(^{tBu_2}ArO,I*)TiI_2 (**9**) (^{1}H-observed HMBC, benzene-d_6, 80 MHz, 298 K).

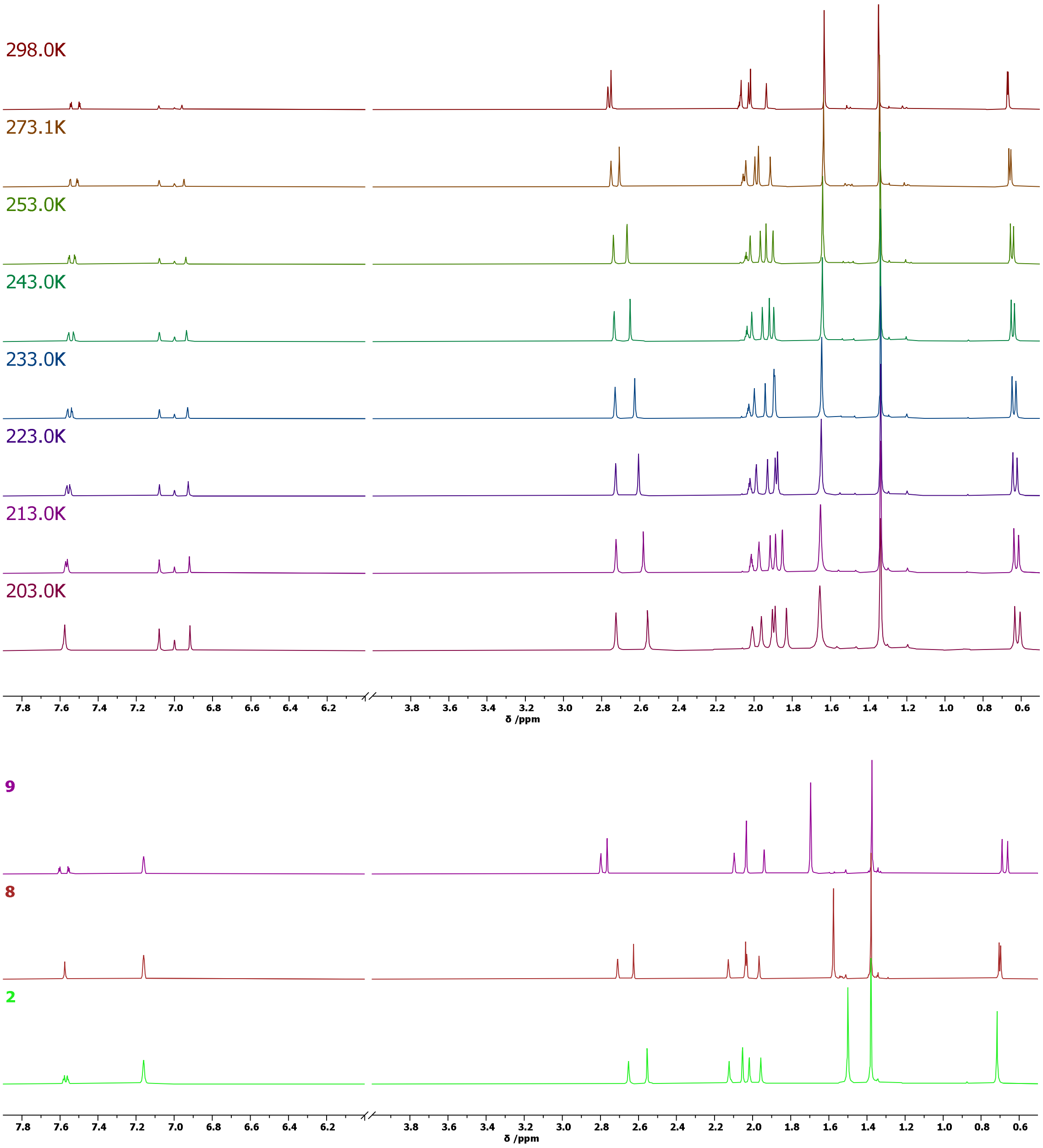

Figure S50 ^{1}H VT NMR spectra of ^{Me_2}SB($^{^tBu_2}$ArO,I*)TiI$_2$ (**9**) as a function of temperature (top; toluene-d_8, 500 MHz, 203–298 K), compared with ^{1}H NMR spectra of **9**, ^{Me_2}SB($^{^tBu_2}$ArO,I*)TiBr$_2$ (**8**), and ^{Me_2}SB($^{^tBu_2}$ArO,I*)TiCl$_2$ (**2**) (bottom; benzene-d_6, 400 MHz, 298 K).

It may be interesting to note that the ^{1}H NMR spectrum of **9** at 203 K more closely resembles that of **2** at 298 K – particularly with respect to the upfield shift of the I***Me** resonance from *ca.* δ 2.75 ppm (298 K) to δ 2.55 ppm (203 K). It is conceivable that this may be consistent with a shift in the position of the A ⇌ B conformational equilibrium – observed as time-averaged chemical shifts in the NMR experiment – towards the B-type of **2** observed in the solid state. While this remains speculative, it would imply that the B-conformation of **9** is thermodynamically preferred, and go some way to explaining the downfield resonances with *less* electronegative halide ligands.

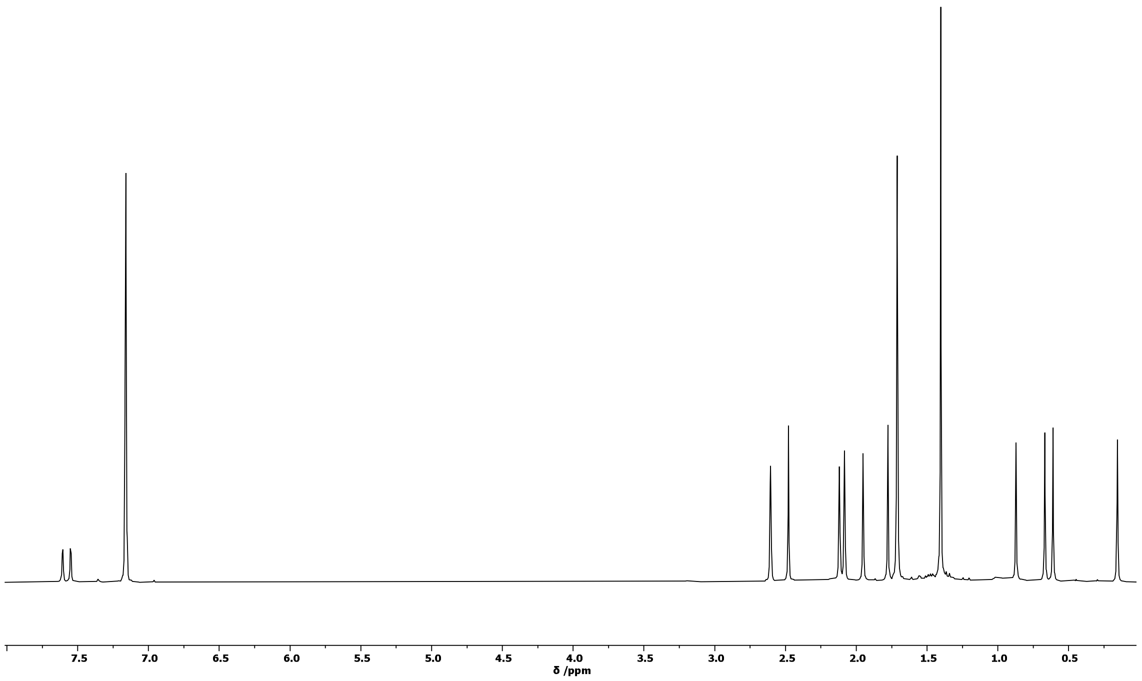

Figure S51 ^{1}H NMR spectrum of $^{Me_2}SB(^{tBu_2}ArO,I^*)TiMe_2$ (**10**) (benzene-d_6, 400 MHz, 298 K).

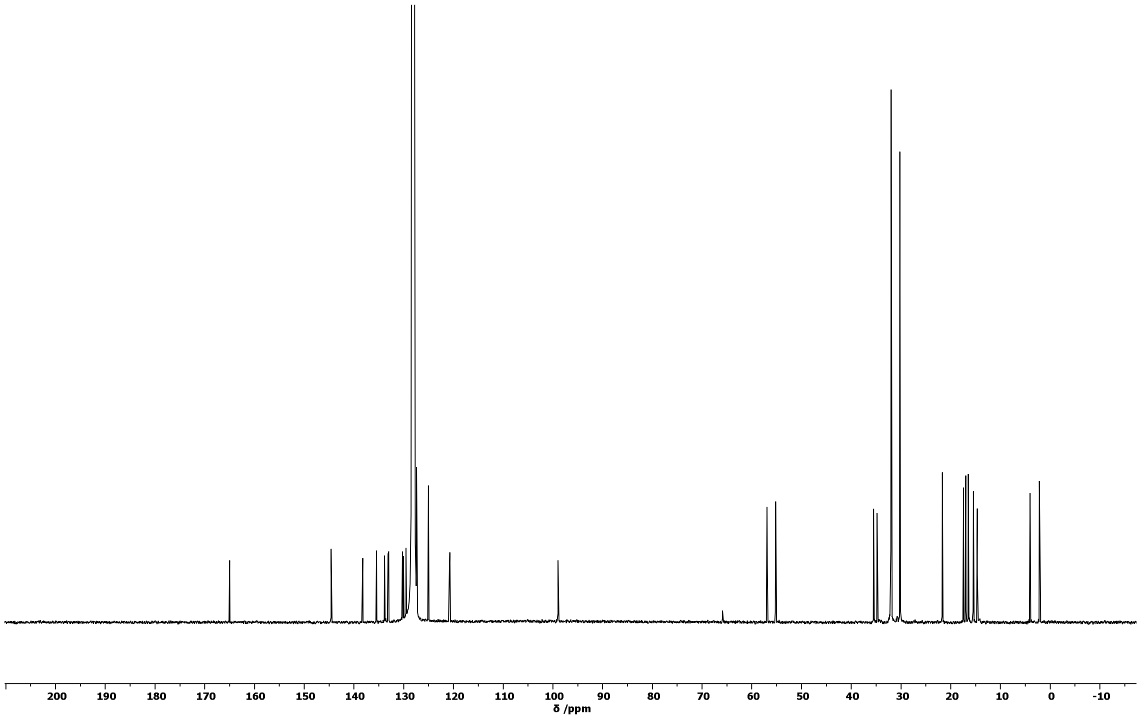

Figure S52 $^{13}C\{^{1}H\}$ NMR spectrum of $^{Me_2}SB(^{tBu_2}ArO,I^*)TiMe_2$ (**10**) (benzene-d_6, 126 MHz, 298 K).

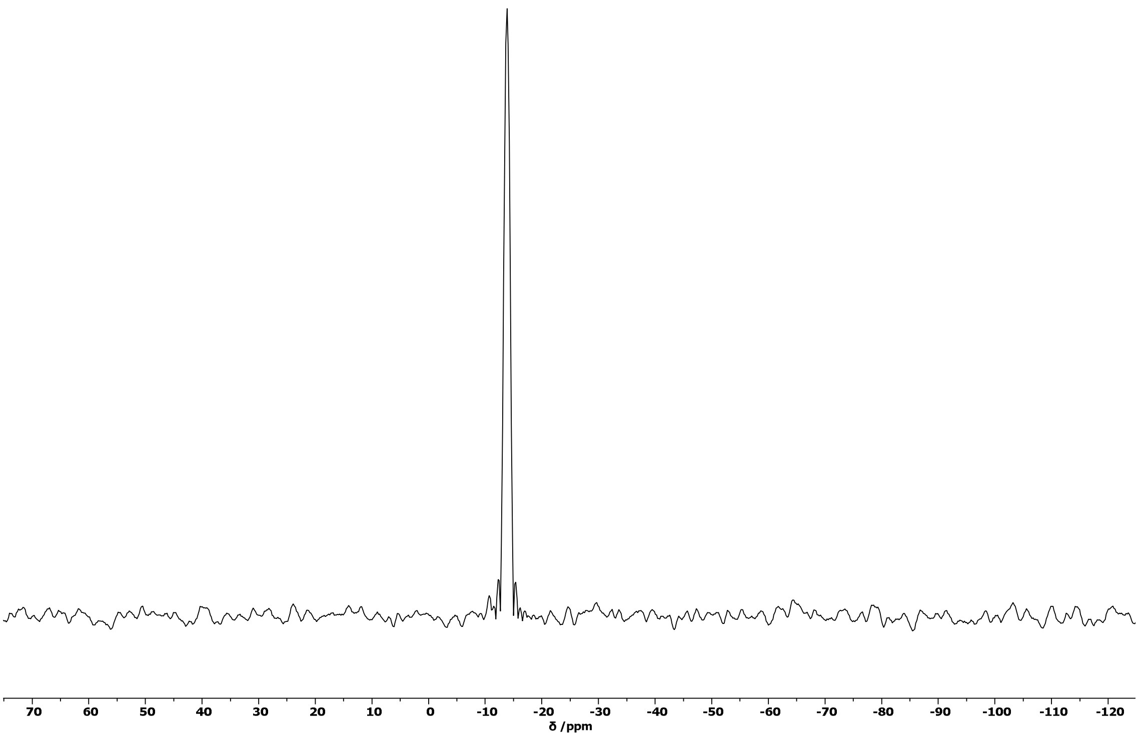

Figure S53 ^{29}Si NMR spectrum of ^{Me_2}SB($^{^tBu_2}$ArO,I*)TiMe$_2$ (**10**) (^{1}H-observed HMBC, benzene-d_6, 80 MHz, 298 K).

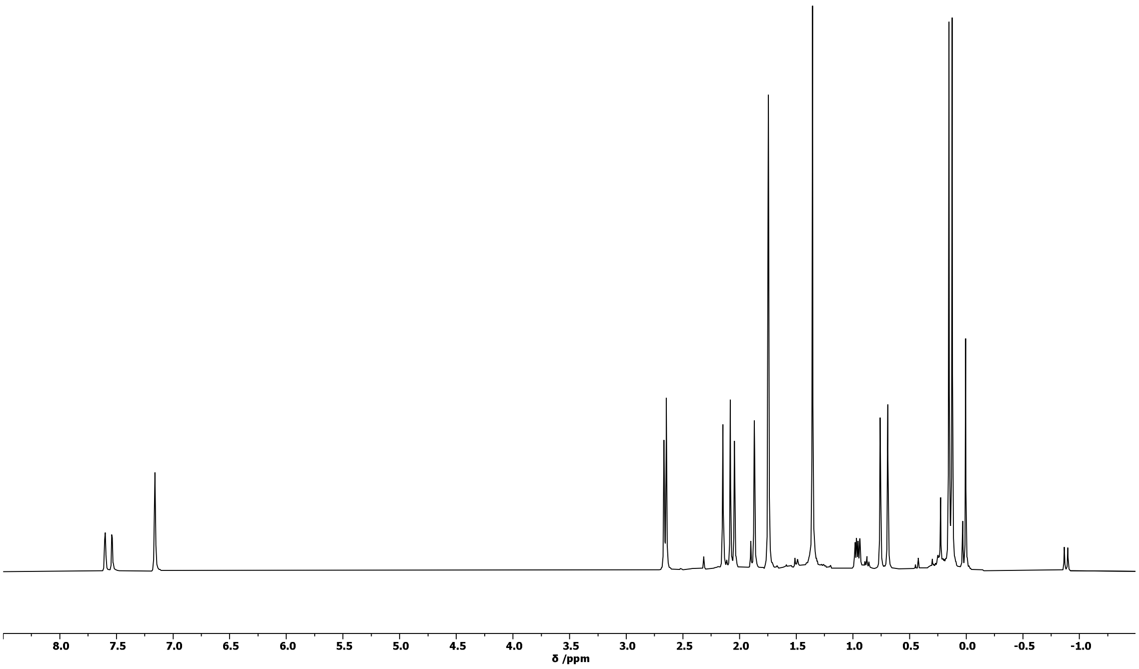

Figure S54 ^{1}H NMR spectrum of ^{Me_2}SB($^{^tBu_2}$ArO,I*)Ti(CH$_2$SiMe$_3$)$_2$ (**11**) (benzene-d_6, 400 MHz, 297 K).

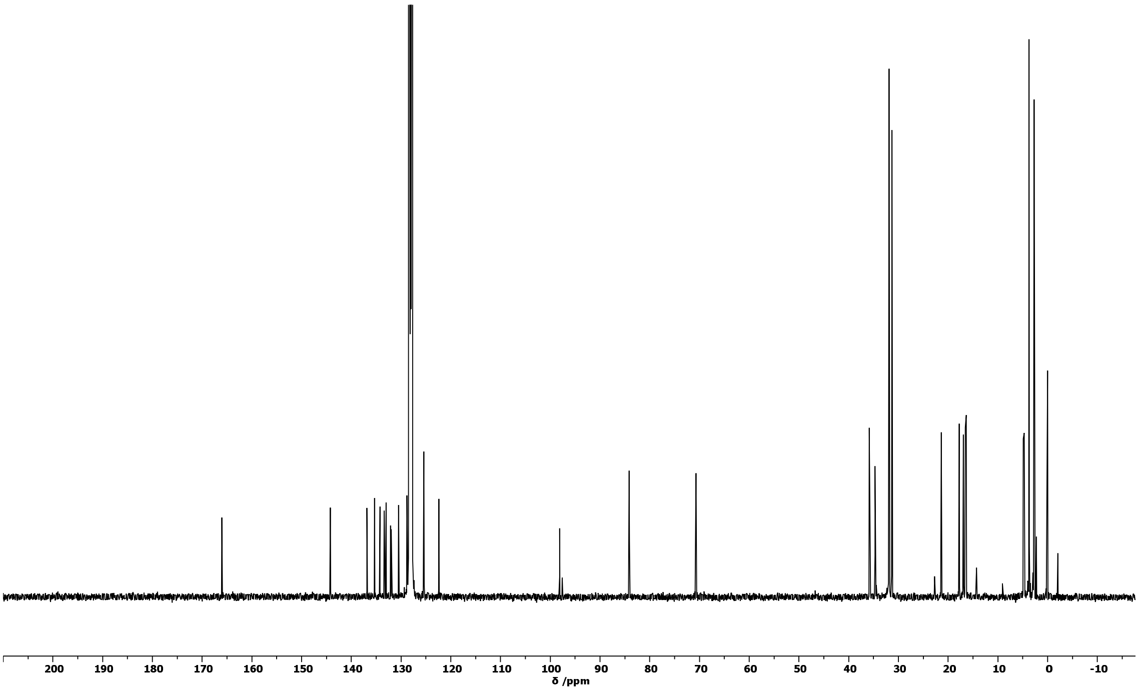

Figure S55 $^{13}C\{^{1}H\}$ NMR spectrum of ^{Me_2}SB($^{t\mathrm{Bu}_2}$ArO,I*)Ti(CH_2SiMe_3)$_2$ (**11**) (benzene-d_6, 101 MHz, 298 K).

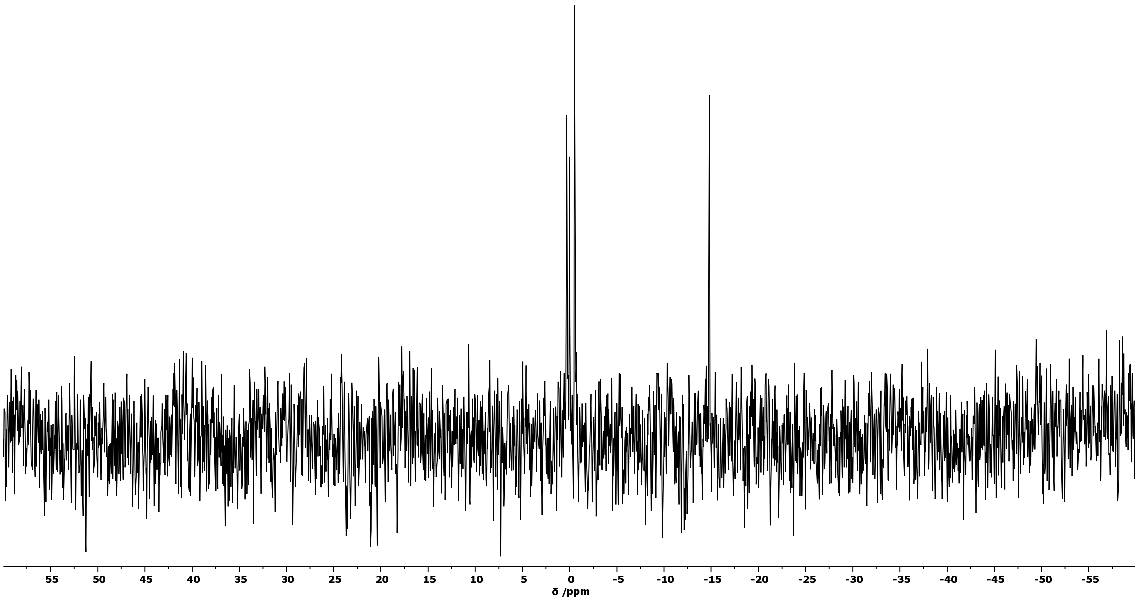

Figure S56 ^{29}Si NMR spectrum of ^{Me_2}SB($^{t\mathrm{Bu}_2}$ArO,I*)Ti(CH_2SiMe_3)$_2$ (**11**) (benzene-d_6, 80 MHz, 297 K).

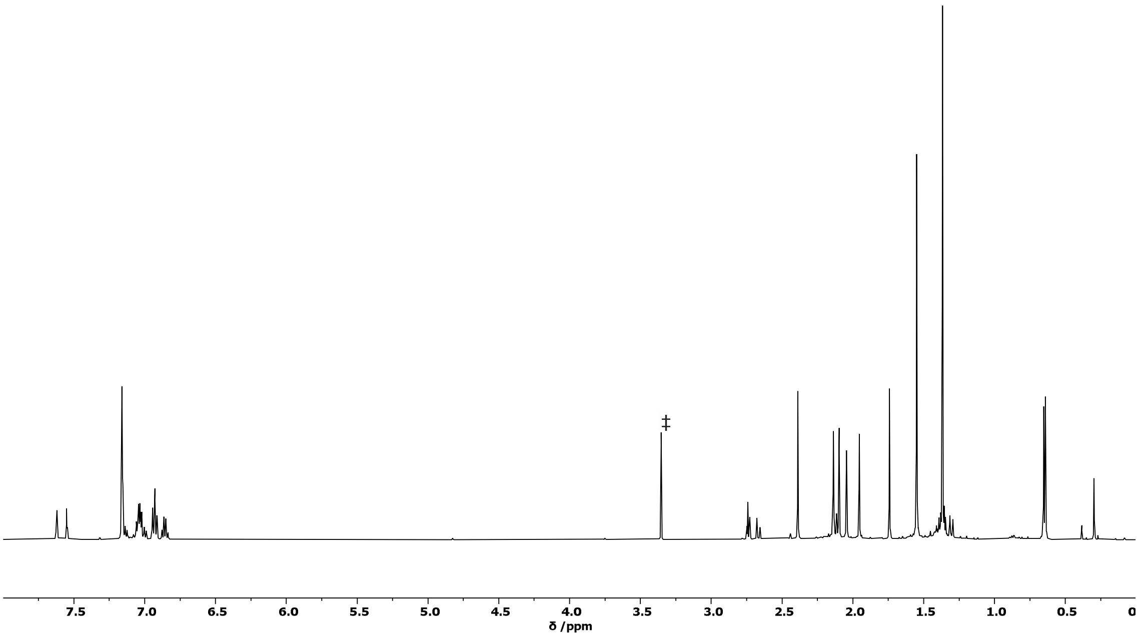

Figure S57 ^{1}H NMR spectrum of ^{Me2}SB(^{tBu2}ArO,I*)TiBn$_2$ (**12**) (benzene-d_6, 500 MHz, 298 K). ‡ denotes residual 1,4-dioxane (approx. 0.19 equivalents relative to **12**).

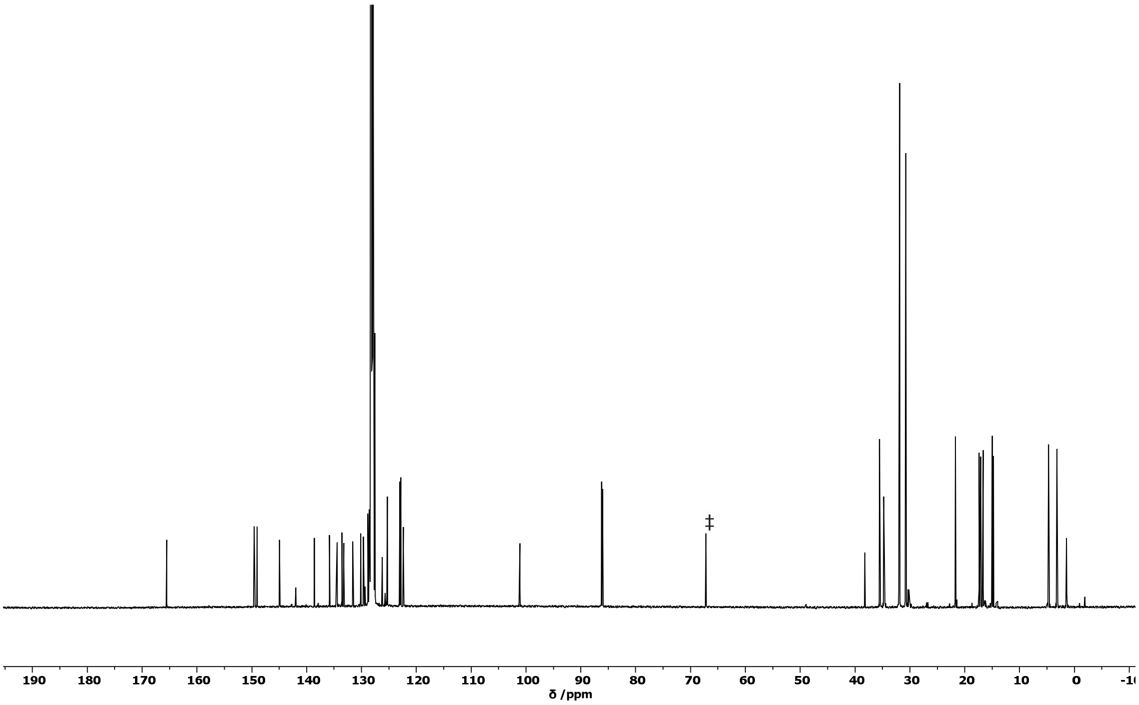

Figure S58 ^{3}C{^{1}H} NMR spectrum of ^{Me2}SB(^{tBu2}ArO,I*)TiBn$_2$ (**12**) (benzene-d_6, 151 MHz, 298 K). ‡ denotes residual 1,4-dioxane.

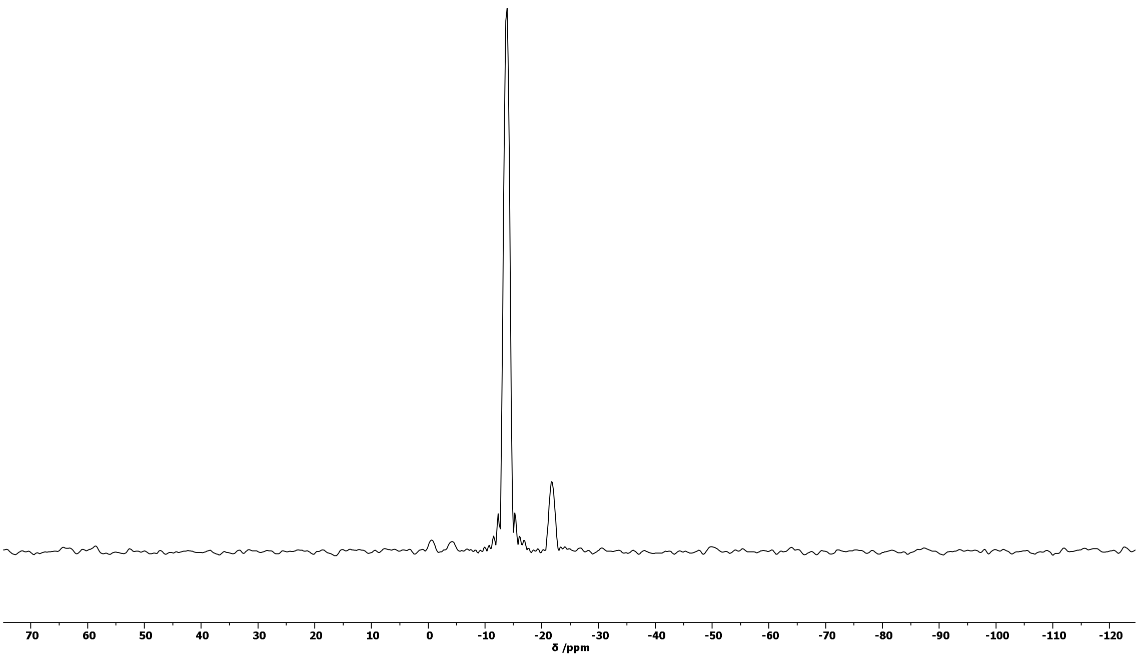

Figure S59 ^{29}Si NMR spectrum of ^{Me_2}SB($^{^tBu_2}$ArO,I*)TiBn$_2$ (**12**) (^{1}H-observed HMBC, benzene-d_6, 80 MHz, 298 K).

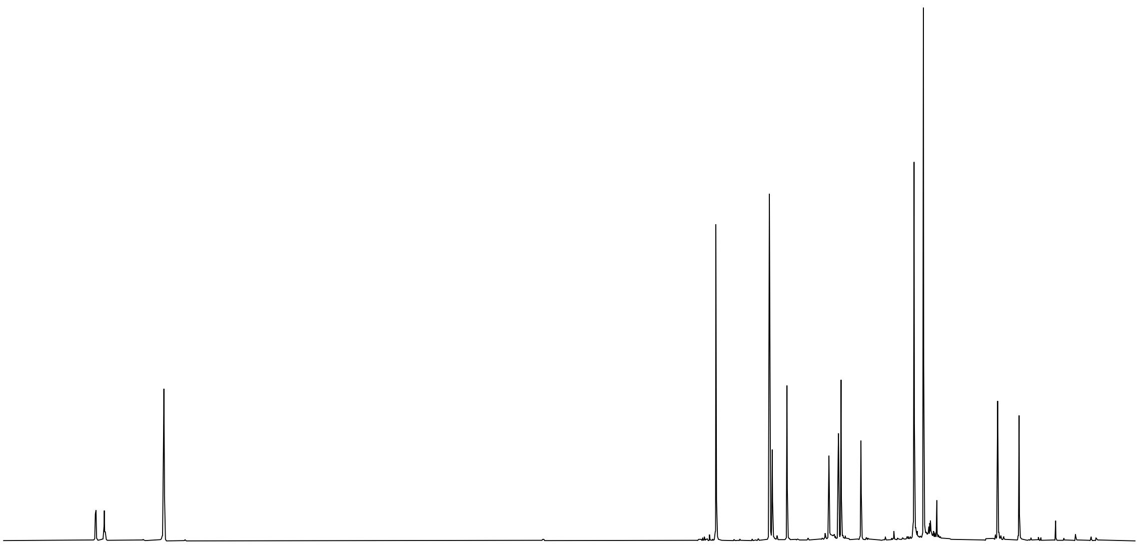

Figure S60 ^{1}H NMR spectrum of ^{Me_2}SB($^{^tBu_2}$ArO,I*)Ti(NMe$_2$)$_2$ (**13**) (benzene-d_6, 500 MHz, 298 K).

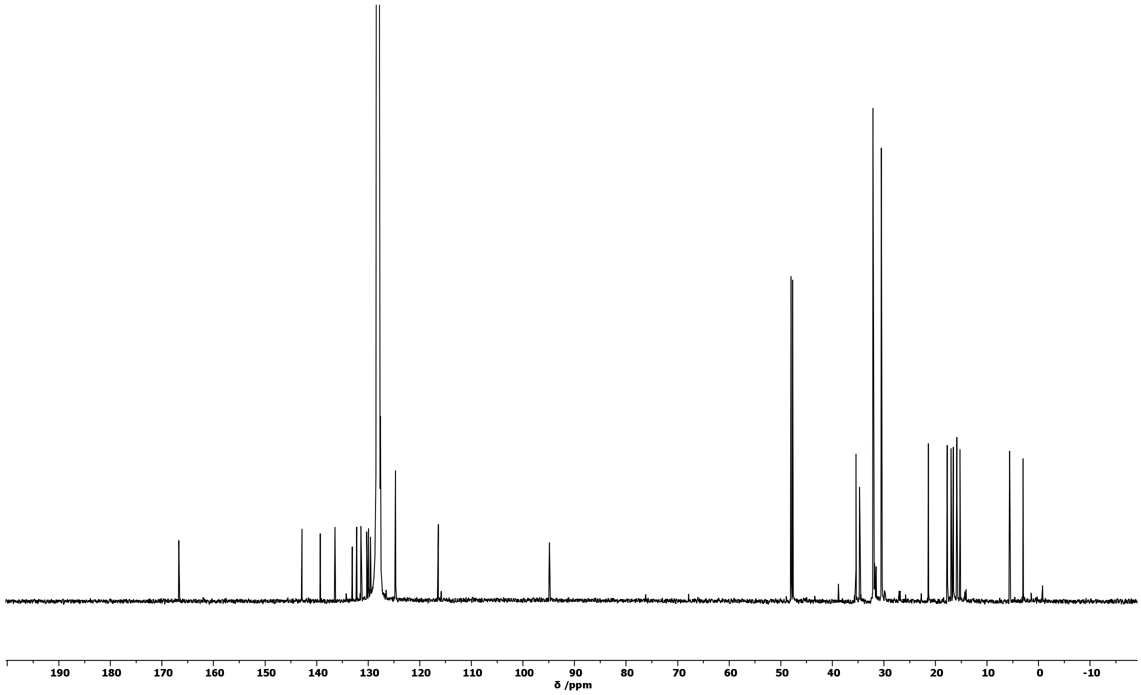

Figure S61 $^{3}C\{^{1}H\}$ NMR spectrum of $^{Me2}SB(^{tBu2}ArO,I^{*})Ti(NMe_2)_2$ (**13**) (benzene-*d*$_6$, 126 MHz, 298 K).

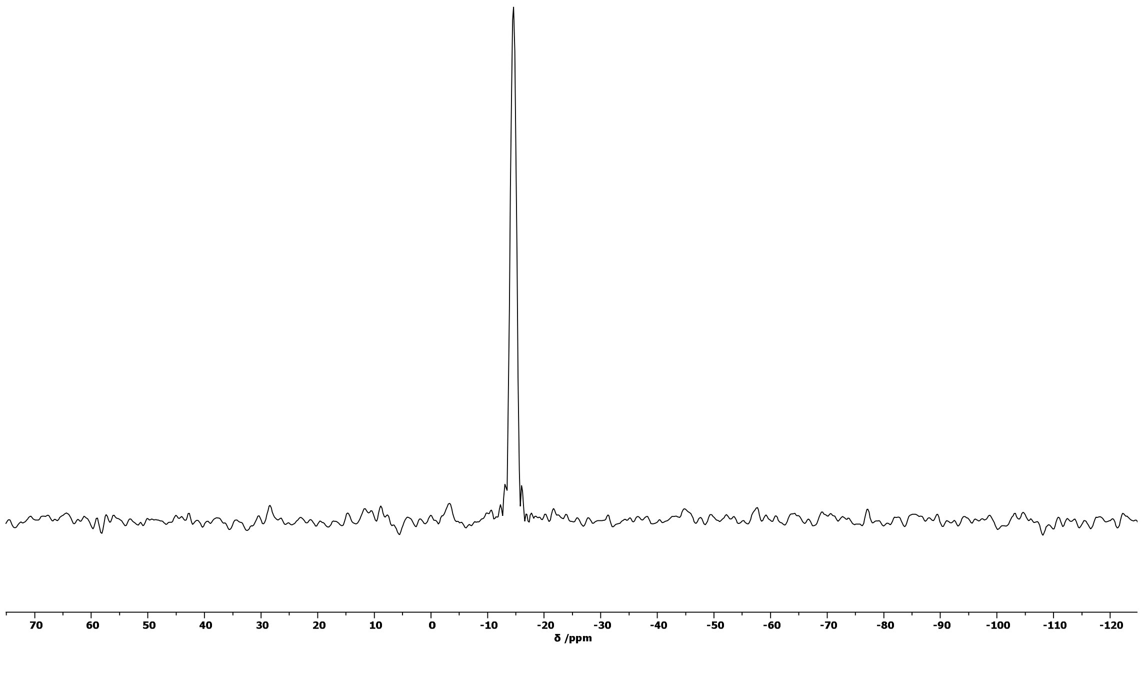

Figure S62 ^{29}Si NMR spectrum of $^{Me2}SB(^{tBu2}ArO,I^{*})Ti(NMe_2)_2$ (**13**) (^{1}H-observed HMBC, benzene-*d*$_6$, 80 MHz, 298 K).

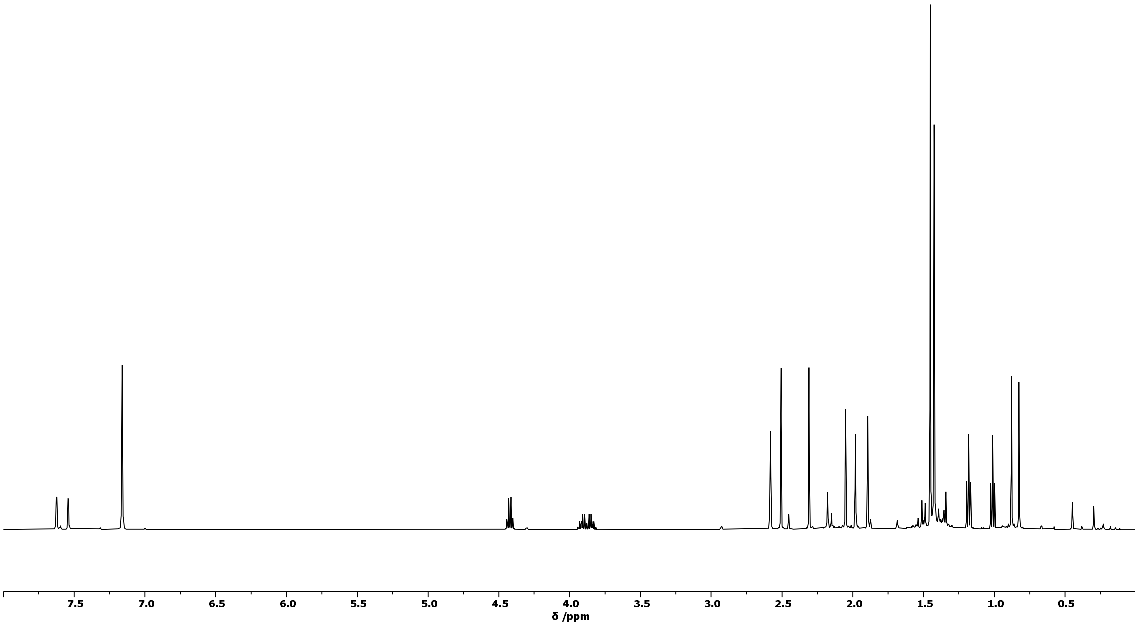

Figure S63 ^{1}H NMR spectrum of ^{Me_2}SB($^{t\mathrm{Bu}_2}$ArO,I*)Ti(OEt)$_2$ (**14**) (benzene-d_6, 500 MHz, 298 K).

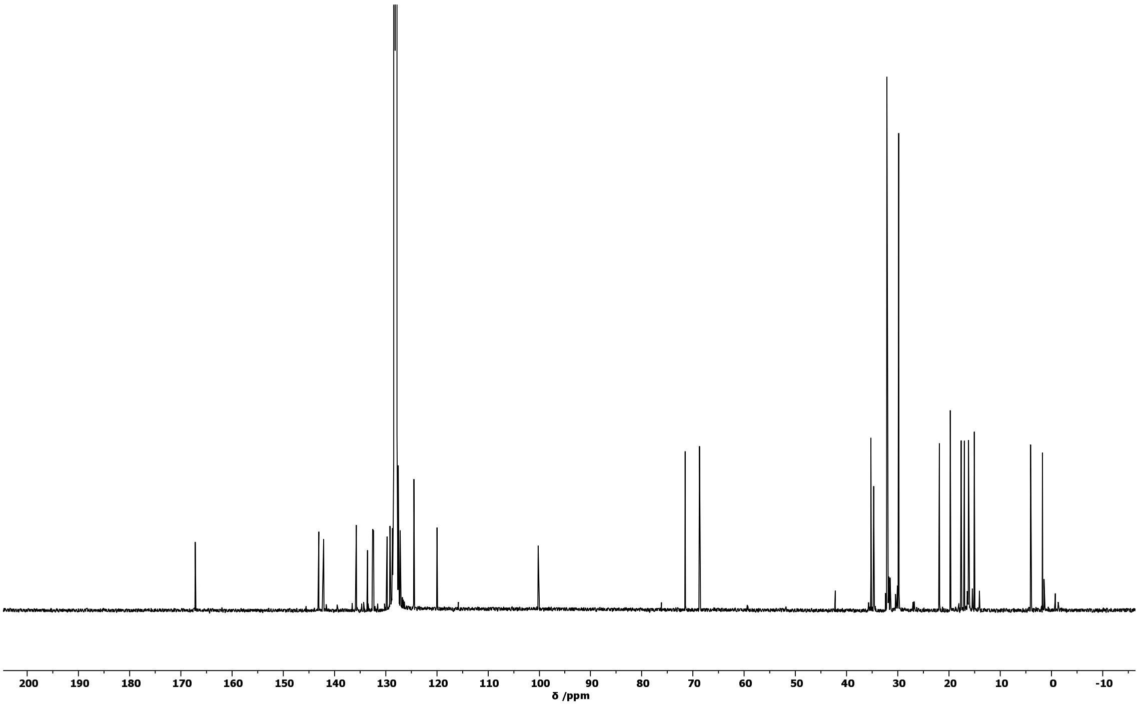

Figure S64 ^{3}C{^{1}H} NMR spectrum of ^{Me_2}SB($^{t\mathrm{Bu}_2}$ArO,I*)Ti(OEt)$_2$ (**14**) (benzene-d_6, 126 MHz, 298 K).

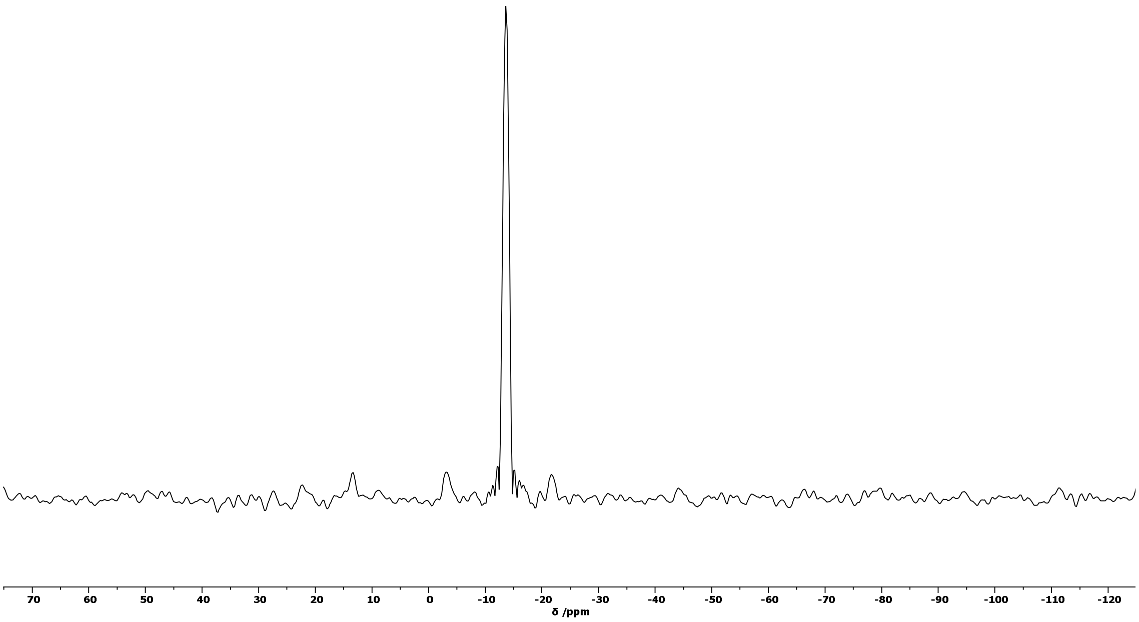

Figure S65 ^{29}Si NMR spectrum of ^{Me_2}SB(^{tBu_2}ArO,I*)Ti(OEt)$_2$ (**14**) (^{1}H-observed HMBC, benzene-d_6, 80 MHz, 298 K).

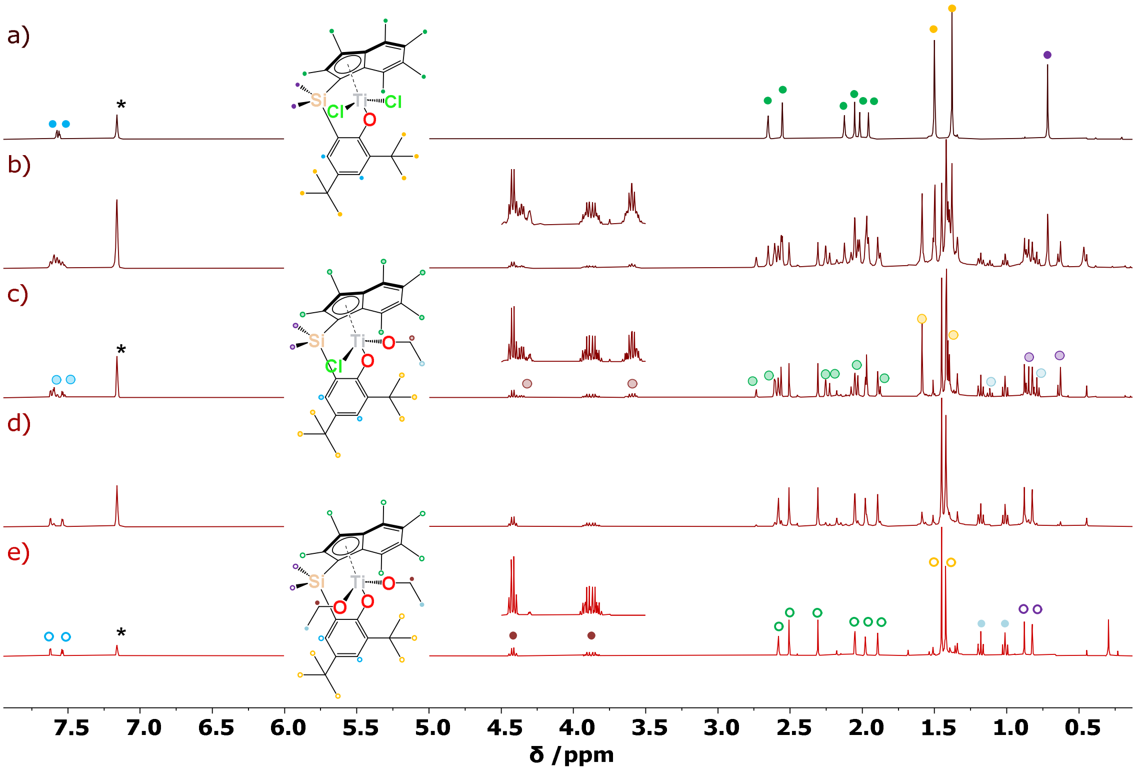

Figure S66 ^{1}H NMR spectra (400 MHz, benzene-d_6, 298 K) of the reaction between ^{Me_2}SB(^{tBu_2}ArO,I*)TiCl$_2$ (**2**) and NaOEt evolving as a function of time, forming first ^{Me_2}SB(^{tBu_2}ArO,I*)Ti(Cl)OEt, then ^{Me_2}SB(^{tBu_2}ArO,I*)Ti(OEt)$_2$ (**14**). Sample heated at b) 60 °C for 17 h, c) 80 °C for 1 h, d) 80 °C for 2.5 h, and e) 60 °C for 14.5 h between spectrum collections. Asterisk (*) denotes residual *protio*-benzene.

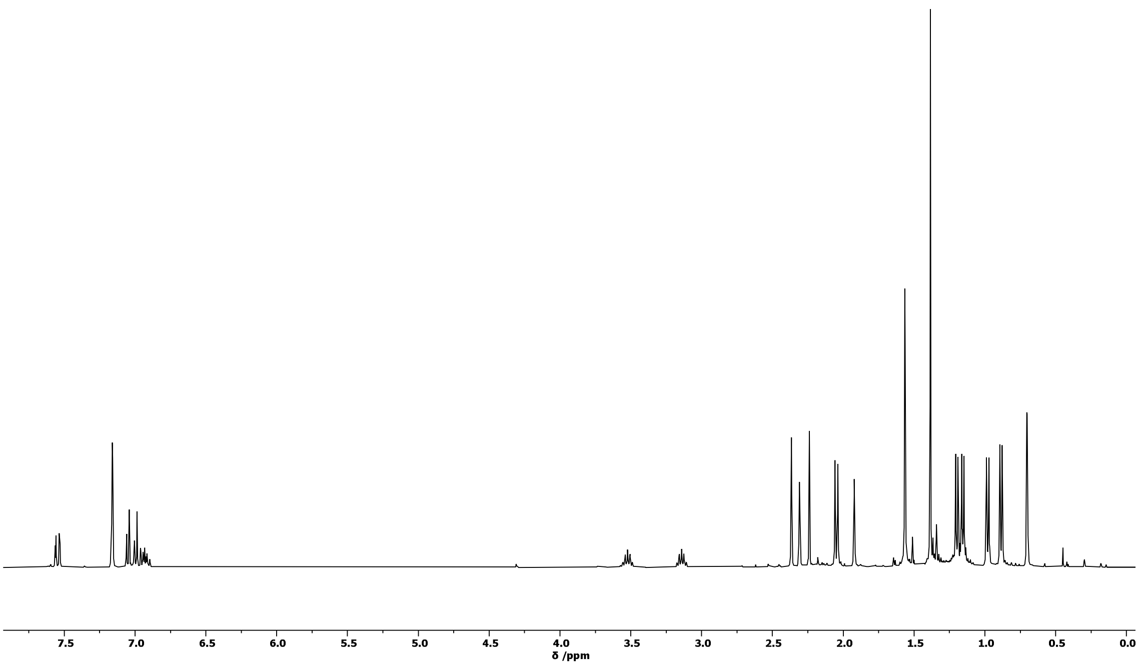

Figure S67 ^{1}H NMR spectrum of ^{Me_2}SB(^{tBu_2}ArO,I*)Ti(ODipp)$_2$ (**15**) (benzene-d_6, 400 MHz, 298 K).

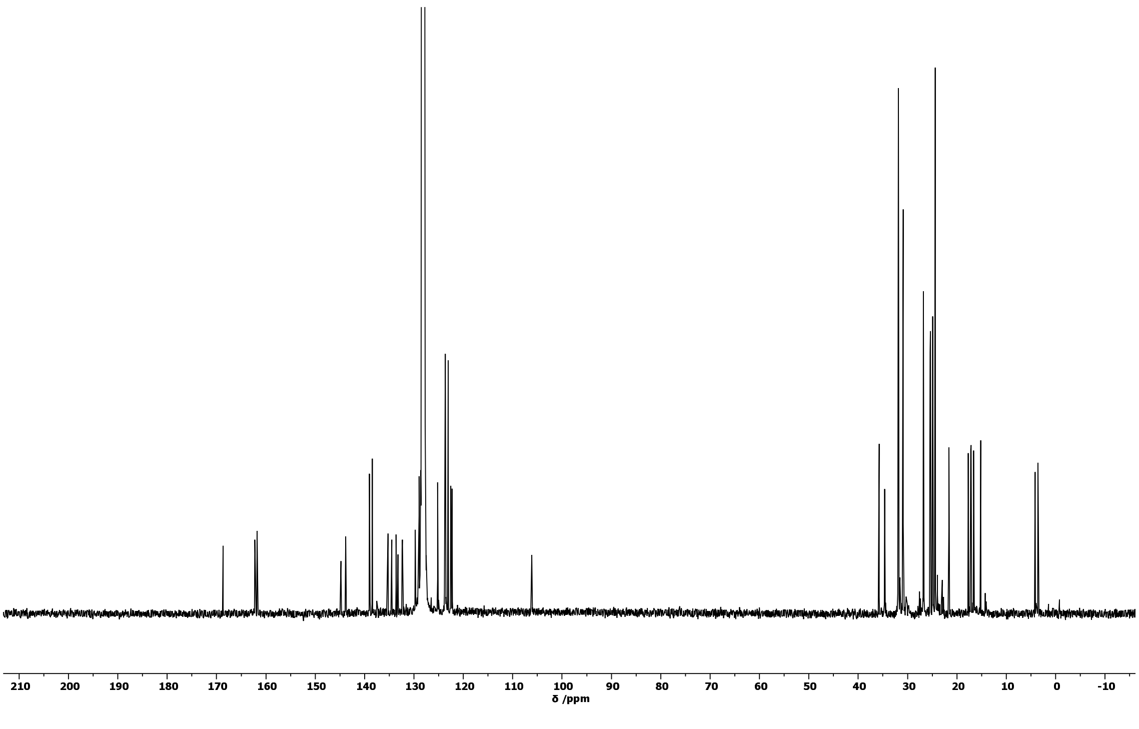

Figure S68 ^{13}C{^{1}H} NMR spectrum of ^{Me_2}SB(^{tBu_2}ArO,I*)Ti(ODipp)$_2$ (**15**) (benzene-d_6, 126 MHz, 298 K).

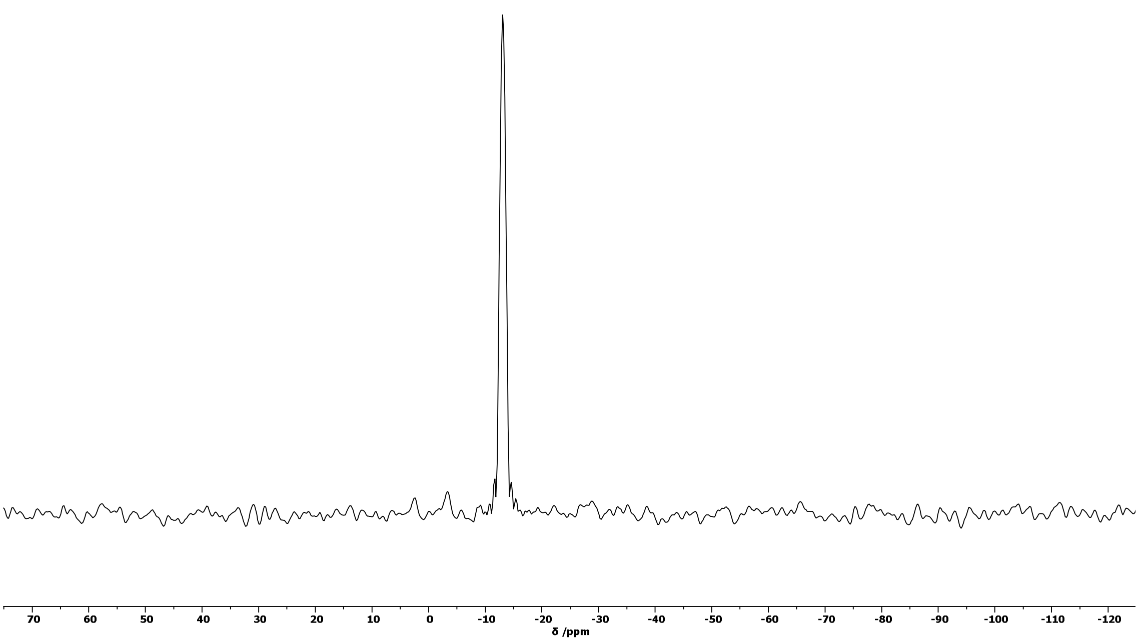

Figure S69 ^{29}Si NMR spectrum of ^{Me_2}SB($^{t\mathrm{Bu}_2}$ArO,I*)Ti(ODipp)$_2$ (**15**) (^{1}H-observed HMBC, benzene-*d*$_6$, 80 MHz, 298 K).

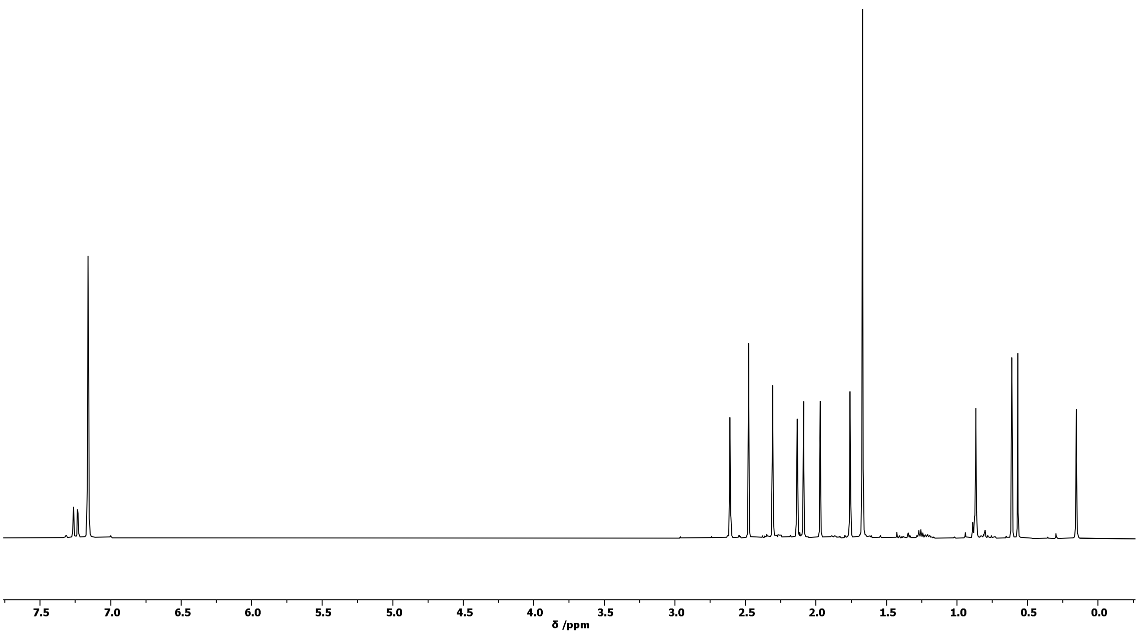

Figure S70 ^{1}H NMR spectrum of ^{Me_2}SB($^{t\mathrm{Bu,Me}}$ArO,I*)TiMe$_2$ (**16**) (benzene-*d*$_6$, 500 MHz, 298 K).

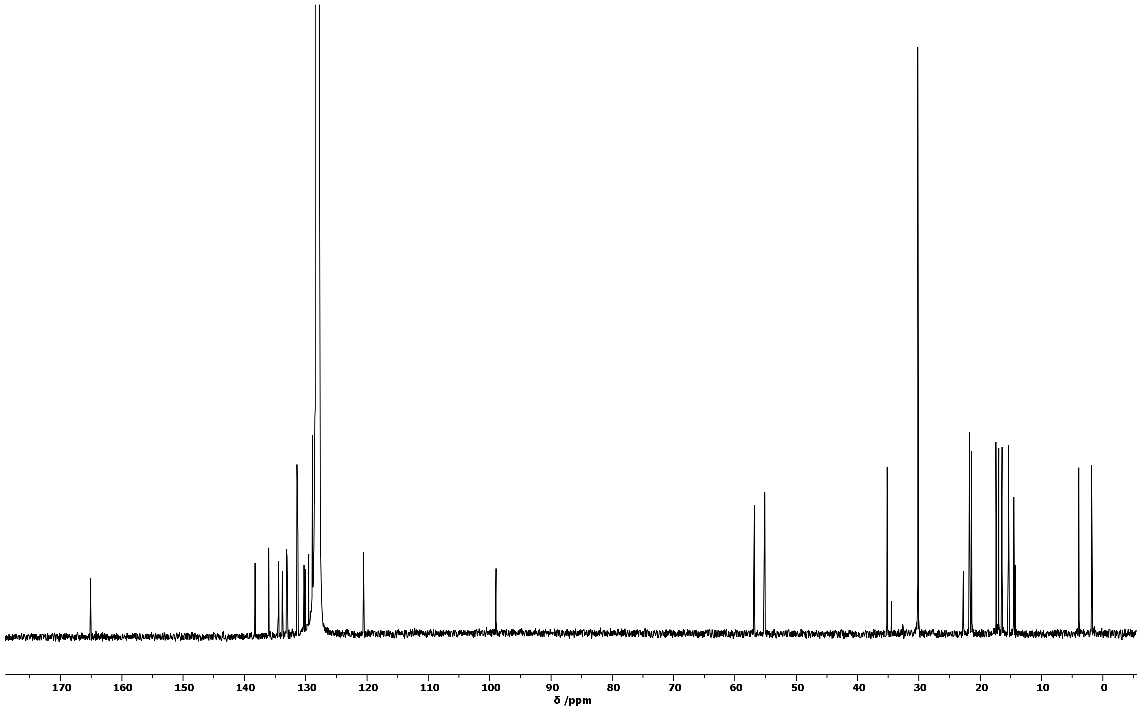

Figure S71 $^{13}C\{^{1}H\}$ NMR spectrum of $^{Me_2}SB(^{tBu,Me}ArO,I^*)TiMe_2$ (**16**) (benzene-d_6, 126 MHz, 298 K).

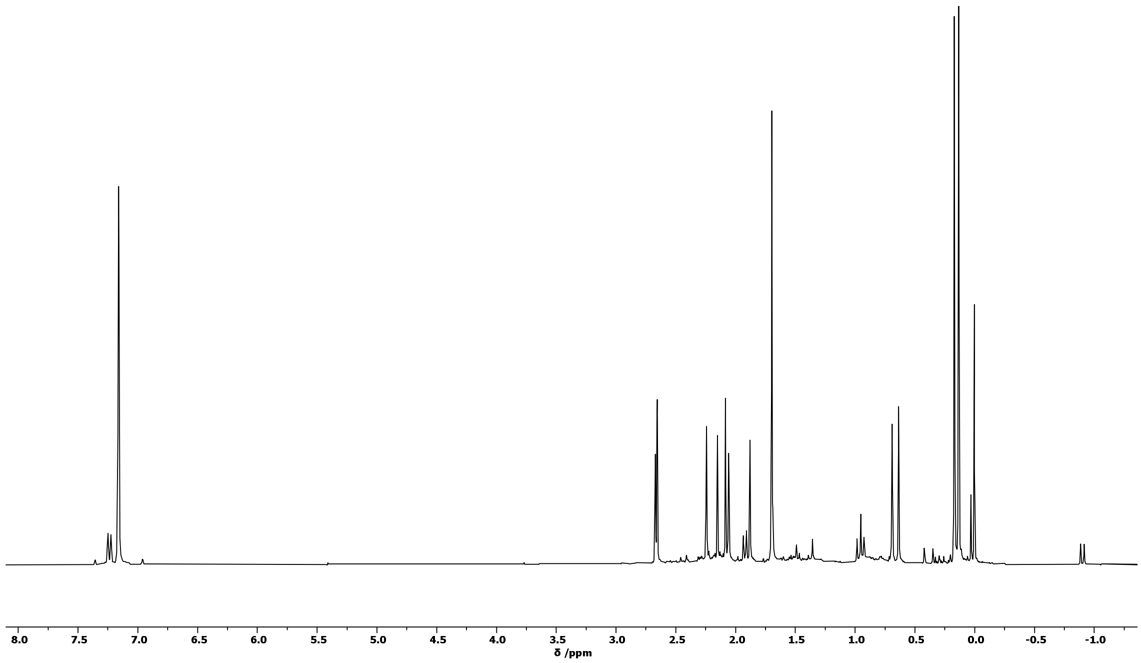

Figure S72 ^{1}H NMR spectrum of $^{Me_2}SB(^{tBu,Me}ArO,I^*)Ti(CH_2SiMe_3)_2$ (**17**) (benzene-d_6, 400 MHz, 298 K).

1.1. Synthesis of $^{Me2}SB(^{tBu2}ArO,I^*)TaCl_3$ (18)

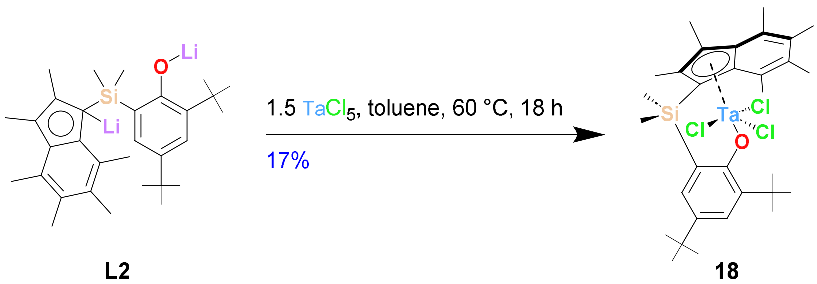

L2 **18**

Scheme S1 Synthesis of PHENI* complex of tantalum(V) by salt metathesis of ligand lithium salt precursor. Yield corresponds to the isolated recrystallized product.

2 g (4.22 mmol) of $^{Me2}SB(^{tBu2}ArO, I^*)Li_2$ (**L2**) and 1.5 eq. of $TaCl_5$ (2.26 g, 6.31 mmol) were added to a clean Schlenk flask containing a stirrer bar. To this was added 30 mL of toluene to give a dark orange slurry, which was stirred overnight and heated to 60 °C. The resulting dark orange solution was washed and filtered with 2 x 25 mL of toluene, leaving a black solid which was washed with 3 x 10 mL of pentane to give a dark orange solution, which was concentrated and recrystallized at –30 °C. The remaining mother liquor was dried under vacuum and the resulting solid was washed with 30 mL of benzene before being dried under vacuum again to give a brown powder. The solid products were combined to give 0.525 g of $^{Me2}SB(^{tBu2}ArO,I^*)TaCl_3$ (**18**) as a brown solid (17% yield). Crystals suitable for single crystal X-ray diffraction were grown by slow evaporation of a hexane solution at 23 °C (Figure S80).

^{1}H NMR (600.42 MHz, benzene-d_6, 298 K): δ 7.64 (d, 1H, $^4J_{HH}$ = 2.4 Hz, 3,5-C_6**H_2**), 7.56 (d, 1H, $^4J_{HH}$ = 2.4 Hz, 3,5-C_6**H_2**), 2.75 (s, 3H, I***Me**), 2.47 (s, 3H, I***Me**), 2.31 (s, 3H, I***Me**), 2.16 (s, 3H, I***Me**), 2.07 (s, 3H, I***Me**), 1.84 (s, 3H, I***Me**), 1.55 (s, 9H, C**Me_3**), 1.31 (s, 9H, C**Me_3**), 0.83 (s, 3H, Si**Me**), 0.77 (s, 3H, Si**Me**) ppm.

$^{13}C\{^1H\}$ NMR (150.99 MHz, benzene-d_6, 298 K): δ 166.24 (***i***Ph), 146.77 (***o,p***Ph), 142.05 (**I***), 140.05 (**I***), 139.64 (**I***), 139.41 (**I***), 138.09 (**I***), 137.04 (**I***), 134.34 (**I***), 132.59 (**I***), 131.43 (***o'***Ph), 128.35 (***m***Ph), 125.95 (***m***Ph), 94.37 (Si**I***), 35.52 (**C**Me_3), 34.77 (**C**Me_3), 31.74 (C**Me_3**), 31.05 (C**Me_3**), 21.85 (I***Me**), 17.55 (I***Me**), 17.08 (I***Me**), 16.99 (I***Me**), 16.71 (I***Me**), 15.86 (I***Me**), 5.81 (Si**Me**), 4.36 (Si**Me**) ppm.

^{29}Si NMR (^{1}H-observed HMBC, benzene-d_6, 119 MHz, 298 K): δ –11.7 ppm.

Anal. calcd for $C_{31}H_{44}Cl_3OSiTa$: C, 49.80; H, 5.89. Found: C, 49.46; H, 5.88.

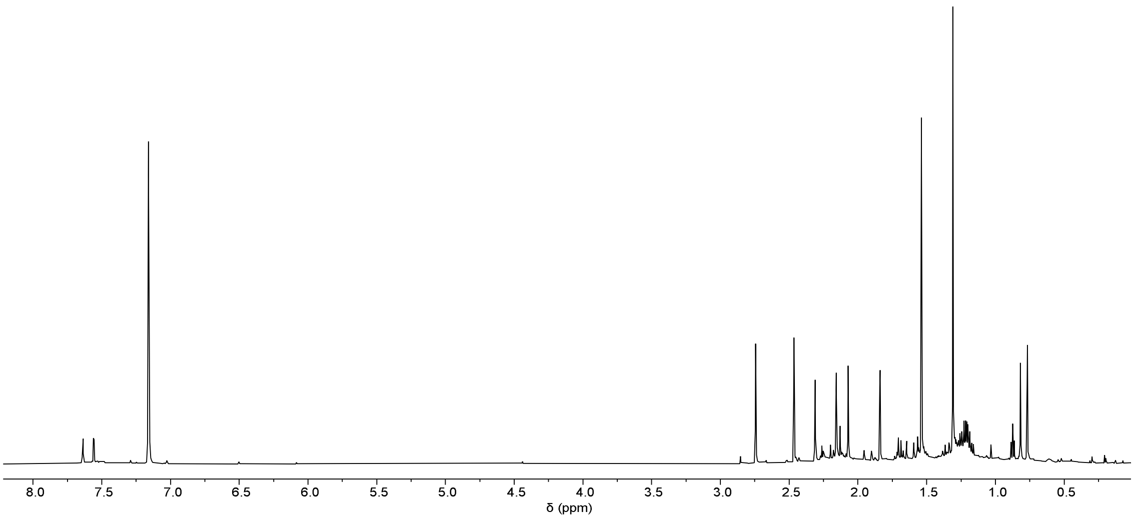

Figure S73 ^{1}H NMR spectrum of $^{Me_2}SB(^{tBu_2}ArO,I^*)TaCl_3$ (**18**) (benzene-d_6, 600 MHz, 298 K).

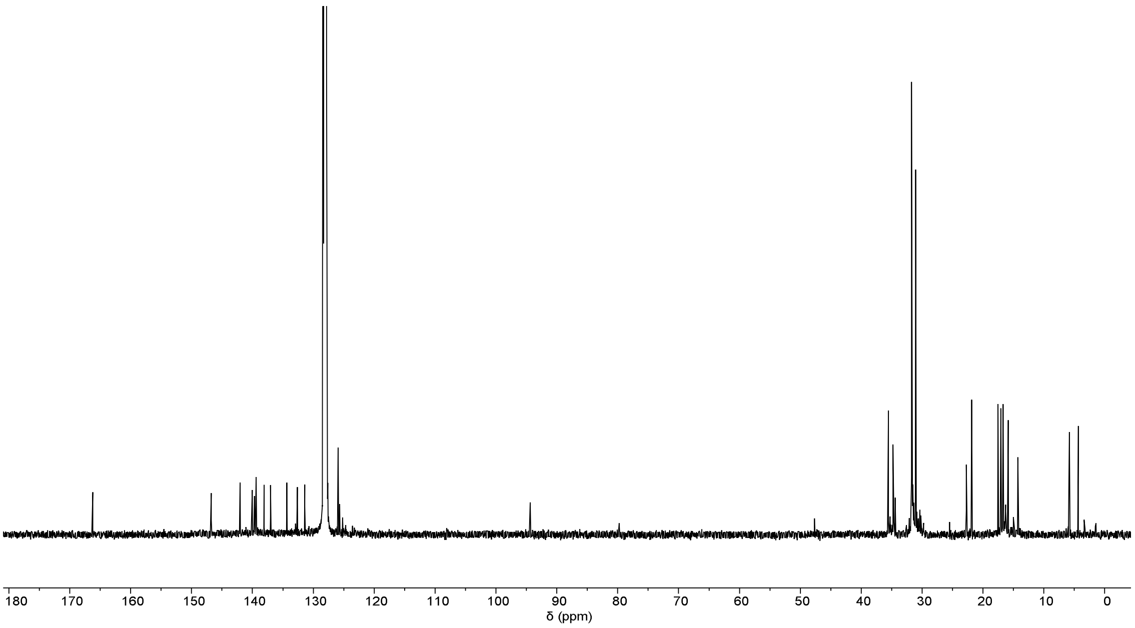

Figure S74 $^{13}C\{^{1}H\}$ NMR spectrum of $^{Me_2}SB(^{tBu_2}ArO,I^*)TaCl_3$ (**18**) (benzene-d_6, 151 MHz, 298 K).

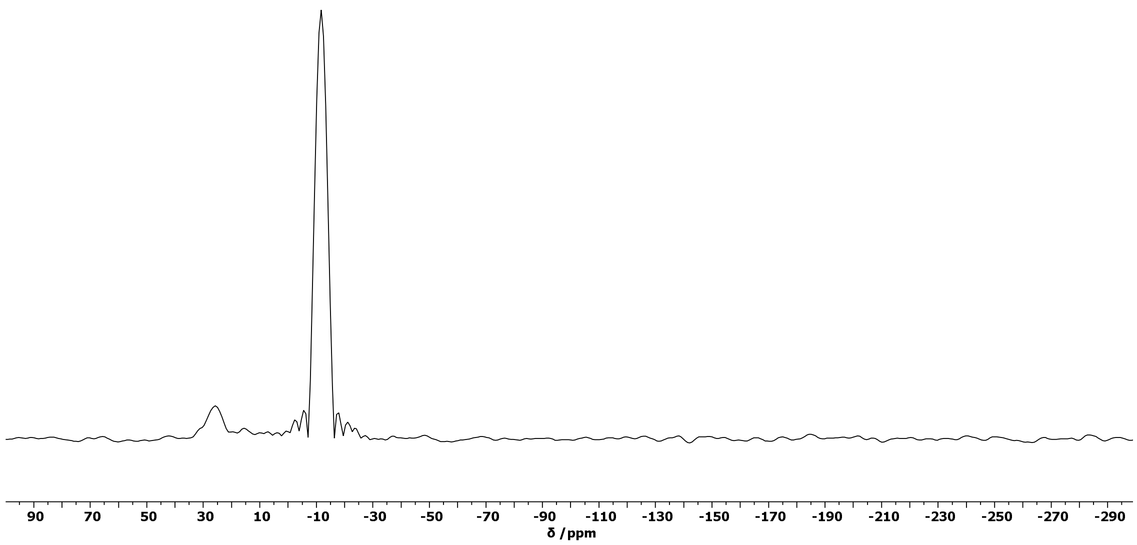

Figure S75 ^{29}Si NMR spectrum of ^{Me_2}SB(^{tBu_2}ArO,I*)TaCl$_3$ (**18**) (^{1}H-observed HMBC, benzene-d_6, 119 MHz, 298 K).

2. Crystallographic parameters

All experiments were carried out with Cu *K*a radiation. H-atom parameters were constrained.

	**P2**	**P3**	**1**	**3**	**4**
**Crystal data**					
Chemical formula	$C_{34}H_{50}OSi$	$C_{44}H_{54}OSi$	$C_{28}H_{38}Cl_2OSiTi$	$C_{31}H_{44}Cl_2OSiZr\cdot C_6H_6$	$C_{31}H_{44}Cl_2HfOSi$
M_r	502.85	627.00	537.50	701.01	710.17
Crystal system, space group	Monoclinic, *I*2/*a*	Triclinic, *P*¯1	Orthorhombic, *Pbca*	Triclinic, *P*¯1	Triclinic, *P*¯1
Temperature (K)	150	150	150	150	150
a, *b*, *c* (Å)	22.3439 (4), 15.8051 (3), 17.2084 (4)	9.2003 (5), 12.9126 (7), 16.6266 (9)	9.3542 (1), 15.5831 (2), 37.8666 (4)	8.6681 (1), 9.2302 (2), 23.6646 (3)	8.8737 (2), 9.2262 (1), 23.7302 (4)
α, β, γ (°)	90, 91.137 (2), 90	90.508 (4), 102.091 (5), 107.826 (5)	90, 90, 90	87.5766 (13), 83.2225 (12), 73.6327 (14)	87.0816 (14), 80.5280 (15), 72.9297 (16)
V (Å^3)	6075.9 (2)	1833.03 (18)	5519.72 (11)	1803.86 (5)	1831.89 (6)
Z	8	2	8	2	2
μ (mm^{-1})	0.84	0.79	4.96	4.37	7.05
Crystal size (mm)	0.18 × 0.13 × 0.13	0.11 × 0.10 × 0.02	0.17 × 0.04 × 0.04	0.22 × 0.16 × 0.03	0.12 × 0.12 × 0.04
**Data collection**					
Diffractometer	Oxford Diffraction SuperNova	Oxford Diffraction SuperNova	Oxford Diffraction SuperNova	Oxford Diffraction SuperNova	Oxford Diffraction SuperNova
Absorption correction	Multi-scan *CrysAlis PRO* (Rigaku Oxford Diffraction, 2017)	Multi-scan *CrysAlis PRO* (Rigaku Oxford Diffraction, 2017)	Multi-scan *CrysAlis PRO* (Rigaku Oxford Diffraction, 2017)	Multi-scan *CrysAlis PRO* (Rigaku Oxford Diffraction, 2017)	Multi-scan *CrysAlis PRO* (Rigaku Oxford Diffraction, 2017)
T_{min}, T_{max}	0.84, 0.90	0.95, 0.98	0.68, 0.82	0.49, 0.88	0.42, 0.75
No. of measured, independent and observed reflections	17207, 6271, 5394 [*I* > 2.0s(*I*)]	17566, 7576, 4916 [*I* > 2.0s(*I*)]	19453, 5709, 4965 [*I* > 2.0s(*I*)]	38067, 7483, 6960 [*I* > 2.0s(*I*)]	31554, 7550, 7215 [*I* > 2.0s(*I*)]

R_{int}	0.039	0.065	0.035	0.035	0.040
θ_{max} (°)	76.3	76.5	76.1	76.2	76.2
$(\sin \theta/\lambda)_{max}$ (Å^{-1})	0.630	0.631	0.630	0.630	0.630
**Refinement**					
$R[F^2 > 2\sigma(F^2)]$, $wR(F^2)$, S	0.045, 0.128, 0.97	0.058, 0.154, 1.02	0.033, 0.083, 0.98	0.032, 0.086, 1.02	0.041, 0.101, 1.03
No. of reflections	6271	7575	5702	7483	7550
No. of parameters	325	415	298	379	353
No. of restraints	0	0	0	0	0
	Method = Modified Sheldrick $w = 1/[s^2(F^2) + (0.07P)^2 + 3.81P]$, where $P = (\max(F_o^2,0) + 2F_c^2)/3$	Method = Modified Sheldrick $w = 1/[s^2(F^2) + (0.04P)^2 + 0.91P]$, where $P = (\max(F_o^2,0) + 2F_c^2)/3$	Method = Modified Sheldrick $w = 1/[s^2(F^2) + (0.03P)^2 + 3.46P]$, where $P = (\max(F_o^2,0) + 2F_c^2)/3$	Method = Modified Sheldrick $w = 1/[s^2(F^2) + (0.04P)^2 + 2.89P]$, where $P = (\max(F_o^2,0) + 2F_c^2)/3$	Method = Modified Sheldrick $w = 1/[s^2(F^2) + (0.03P)^2 + 10.74P]$, where $P = (\max(F_o^2,0) + 2F_c^2)/3$
$(\Delta/\sigma)_{max}$	0.001	0.0004	0.002	0.002	0.001
$\Delta\rangle_{max}$, $\Delta\rangle_{min}$ (e Å^{-3})	0.51, -0.31	0.90, -0.54	0.35, -0.34	0.83, -0.57	2.11, -1.21

	**5**	**6**	**7**	**8**	**9**
**Crystal data**					
Chemical formula	$C_{41}H_{48}Cl_2OSiTi$	$C_{33}H_{48.00}Cl_2OSiTi$	$C_{36}H_{46}Cl_2OSiTi$	$C_{31}H_{44}Br_2OSiTi·C_6H_6$	$3(C_{31}H_{44}I_2OSiTi)·C_4H_{10}O$
M_r	703.72	607.63	641.62	746.60	2361.39
Crystal system, space group	Triclinic, *P*¯1	Orthorhombic, *Pn*2$_1$*a*	Monoclinic, *P*2$_1$/*c*	Triclinic, *P*¯1	Triclinic, *P*¯1
Temperature (K)	150	100	150	150	100
a, *b*, *c* (Å)	10.6362 (8), 13.3872 (8), 16.0218 (12)	17.3507 (1), 23.1111 (1), 16.6347 (1)	24.6520 (11), 9.3785 (5), 16.9267 (15)	9.0454 (2), 9.2217 (1), 23.2505 (4)	10.2384 (1), 22.7315 (2), 23.8545 (1)
α, β, γ (°)	66.408 (6), 73.901 (6), 71.856 (6)	90, 90, 90	90, 98.503 (6), 90	92.3663 (12), 99.4751 (15), 107.5194 (14)	71.1207 (6), 87.0574 (6), 87.2403 (6)
V (Å^3)	1955.9 (3)	6670.41 (6)	3870.4 (4)	1815.70 (6)	5243.24 (7)
Z	2	8	4	2	2
μ (mm^{-1})	3.62	4.16	3.61	5.08	16.43
Crystal size (mm)	0.16 × 0.04 × 0.02	0.51 × 0.06 × 0.05	0.30 × 0.21 × 0.06	0.18 × 0.09 × 0.05	0.27 × 0.12 × 0.02
**Data collection**					
Diffractometer	Oxford Diffraction SuperNova	XtaLAB Synergy DW	SuperNova, Dual, Cu at home/near, Atlas	Oxford Diffraction SuperNova	XtaLAB Synergy DW
Absorption correction	Multi-scan *CrysAlis PRO* (Rigaku Oxford Diffraction, 2017)	Multi-scan *CrysAlis PRO* (Rigaku Oxford Diffraction, 2017)	Gaussian *CrysAlis PRO* 1.171.41.93a (Rigaku Oxford Diffraction, 2020) Numerical absorption correction based on gaussian integration over a multifaceted crystal model Empirical absorption correction	Multi-scan *CrysAlis PRO* (Rigaku Oxford Diffraction, 2017)	Multi-scan *CrysAlis PRO* (Rigaku Oxford Diffraction, 2017)

			using spherical harmonics, implemented in SCALE3 ABSPACK scaling algorithm.		
T_{min}, T_{max}	0.77, 0.93	0.51, 0.83	0.416, 1.000	0.56, 1.00	0.35, 0.72
No. of measured, independent and observed reflections	14761, 5911, 4306 [I > 2.0s(I)]	119695, 13499, 10609 [I > 2.0s(I)]	49770, 7914, 5835 [I > 2s(I)]	29485, 7525, 6961 [I > 2.0s(I)]	246637, 21488, 15686 [I > 2.0s(I)]
R_{int}	0.049	0.140	0.112	0.029	0.134
θ_{max} (°)	61.5	75.2	74.5	76.1	76.2
$(\sin \theta/\lambda)_{max}$ ($Å^{-1}$)	0.570	0.627	0.625	0.630	0.630
**Refinement**					
$R[F^2 > 2\sigma(F^2)]$, $wR(F^2)$, S	0.047, 0.124, 0.96	0.071, 0.205, 0.92	0.150, 0.376, 1.05	0.033, 0.091, 1.00	0.063, 0.188, 0.98
No. of reflections	5910	13499	7914	7525	21488
No. of parameters	415	686	383	379	1018
No. of restraints	0	1	0	0	0
	Method = Modified Sheldrick $w = 1/[s^2(F^2) + (0.06P)^2 + 1.1P]$, where $P = (\max(F_o^2,0) + 2F_c^2)/3$	Method = Modified Sheldrick $w = 1/[s^2(F^2) + (0.16P)^2 + 6.79P]$, where $P = (\max(F_o^2,0) + 2F_c^2)/3$	$w = 1/[s^2(F_o^2) + (0.0955P)^2 + 51.9365P]$ where $P = (F_o^2 + 2F_c^2)/3$	Method = Modified Sheldrick $w = 1/[s^2(F^2) + (0.05P)^2 + 1.88P]$, where $P = (\max(F_o^2,0) + 2F_c^2)/3$	Method = Modified Sheldrick $w = 1/[s^2(F^2) + (0.13P)^2 + 9.02P]$, where $P = (\max(F_o^2,0) + 2F_c^2)/3$
$(\Delta/\sigma)_{max}$	0.0004	0.003	0.001	0.001	0.001
$\Delta\rangle_{max}$, $\Delta\rangle_{min}$ (e $Å^{-3}$)	0.65, -0.58	2.71, -0.95	1.33, -0.97	0.68, -0.60	3.20, -2.26
Absolute structure parameter		0.671 (10)			

	**10**	**11**	**13**	**15**	**18**
**Crystal data**					
Chemical formula	$C_{33}H_{50}OSiTi$	$C_{39}H_{66}OSi_3Ti·C_5H_{12}$	$2(C_{35}H_{56}N_2OSiTi)·C_5H_{12}$	$C_{55}H_{78}O_3SiTi$	$C_{31}H_{44}Cl_3OSiTa·C_6H_{14}$
M_r	538.74	755.23	1265.70	863.16	834.26
Crystal system, space group	Triclinic, $P\bar{1}$	Triclinic, $P\bar{1}$	Triclinic, $P\bar{1}$	Triclinic, $P\bar{1}$	Triclinic, $P\bar{1}$
Temperature (K)	150	150	150	150	150
a, *b*, *c* (Å)	9.0440 (2), 13.9610 (5), 14.0102 (5)	11.3033 (4), 13.1608 (6), 17.5863 (5)	10.5569 (3), 11.8446 (3), 15.2700 (4)	10.0716 (3), 12.3398 (3), 21.4451 (8)	9.0349 (2), 9.8594 (2), 21.8940 (5)
α, β, γ (°)	114.214 (3), 92.161 (2), 103.906 (2)	106.682 (3), 90.040 (3), 112.380 (4)	100.985 (2), 90.922 (2), 99.828 (2)	88.842 (3), 76.694 (3), 71.553 (3)	81.5889 (19), 87.5783 (18), 75.3841 (18)
V (Å^3)	1547.39 (10)	2299.34 (16)	1844.58 (8)	2456.41 (14)	1866.85 (7)
Z	2	2	1	2	2
μ (mm^{-1})	2.87	2.53	2.49	2.02	7.91
Crystal size (mm)	0.25 × 0.16 × 0.14	0.32 × 0.20 × 0.04	0.10 × 0.07 × 0.05	0.26 × 0.13 × 0.05	0.07 × 0.06 × 0.03
**Data collection**					
Diffractometer	Oxford Diffraction SuperNova	SuperNova, Dual, Cu at home/near, Atlas	Oxford Diffraction SuperNova	SuperNova, Dual, Cu at home/near, Atlas	Oxford Diffraction SuperNova
Absorption correction	Multi-scan *CrysAlis PRO* (Rigaku Oxford Diffraction, 2017)	Gaussian *CrysAlis PRO* 1.171.41.93a (Rigaku Oxford Diffraction, 2020) Numerical absorption correction based on gaussian integration over a multifaceted crystal model Empirical absorption correction using spherical harmonics, implemented in	–	Gaussian *CrysAlis PRO* 1.171.41.93a (Rigaku Oxford Diffraction, 2020) Numerical absorption correction based on gaussian integration over a multifaceted crystal model Empirical absorption correction using spherical harmonics, implemented in	Multi-scan *CrysAlis PRO* (Rigaku Oxford Diffraction, 2017)

		SCALE3 ABSPACK scaling algorithm.		SCALE3 ABSPACK scaling algorithm.	
T_{min}, T_{max}	0.56, 0.67	0.528, 1.000	–	0.629, 1.000	0.72, 0.79
No. of measured, independent and observed reflections	22230, 6393, 5844 [I > 2.0s(I)]	23343, 9374, 7646 [I > 2s(I)]	29935, 7605, 6767 [I > 2.0s(I)]	26222, 10032, 8724 [I > 2s(I)]	19499, 7714, 6842 [I > 2.0s(I)]
R_{int}	0.033	0.042	0.029	0.031	0.041
θ_{max} (°)	76.1	74.5	76.0	74.5	76.1
$(\sin \theta/\lambda)_{max}$ (Å^{-1})	0.630	0.625	0.629	0.625	0.629
**Refinement**					
$R[F^2 > 2\sigma(F^2)]$, $wR(F^2)$, S	0.033, 0.097, 0.91	0.053, 0.149, 1.04	0.033, 0.093, 0.90	0.036, 0.095, 1.02	0.037, 0.097, 0.99
No. of reflections	6392	9374	7604	10032	7714
No. of parameters	325	464	397	563	388
No. of restraints	0	21	3	0	0
	Method = Modified Sheldrick $w = 1/[s^2(F^2) + (0.07P)^2 + 0.48P]$, where $P = (\max(F_o^2,0) + 2F_c^2)/3$	$w = 1/[s^2(F_o^2) + (0.0729P)^2 + 1.5926P]$ where $P = (F_o^2 + 2F_c^2)/3$	Method = Modified Sheldrick $w = 1/[s^2(F^2) + (0.06P)^2 + 0.71P]$, where $P = (\max(F_o^2,0) + 2F_c^2)/3$	$w = 1/[s^2(F_o^2) + (0.0505P)^2 + 0.4454P]$ where $P = (F_o^2 + 2F_c^2)/3$	Method = Modified Sheldrick $w = 1/[s^2(F^2) + (0.05P)^2 + 1.88P]$, where $P = (\max(F_o^2,0) + 2F_c^2)/3$
$(\Delta/\sigma)_{max}$	0.001	0.001	0.001	0.001	0.001
$\Delta\rangle_{max}$, $\Delta\rangle_{min}$ (e Å^{-3})	0.50, -0.35	1.41, -0.89	0.34, -0.27	0.31, -0.30	1.39, -0.90

Computer programs: SuperNova, (Oxford Diffraction, 2010), *CrysAlis PRO* 1.171.39.46 (Rigaku OD, 2018), *CrysAlis PRO* 1.171.41.93a (Rigaku OD, 2020), *CrysAlis PRO* (Rigaku Oxford Diffraction, 2017), *SUPERFLIP* (Palatinus & Chapuis, 2007), *SUPERFLIP* Palatinus, L.; Chapuis, G. J. Appl. Cryst. 2007, 40, 786-790., *CRYSTALS* (Betteridge *et al.*, 2003), *SHELXL2014* (Sheldrick, 2014), *SHELXL2018*/3 (Sheldrick, 2018), *CAMERON* (Watkin *et al.*, 1996), *ORTEP-3 for Windows* (Farrugia, 1997), *ORTEP-3 for Windows* Farrugia, L. J. J. Appl. Cryst. 1997, 30, 565., *WinGX* publication routines (Farrugia, 1999).

2.1. Supplementary X-ray crystal structures

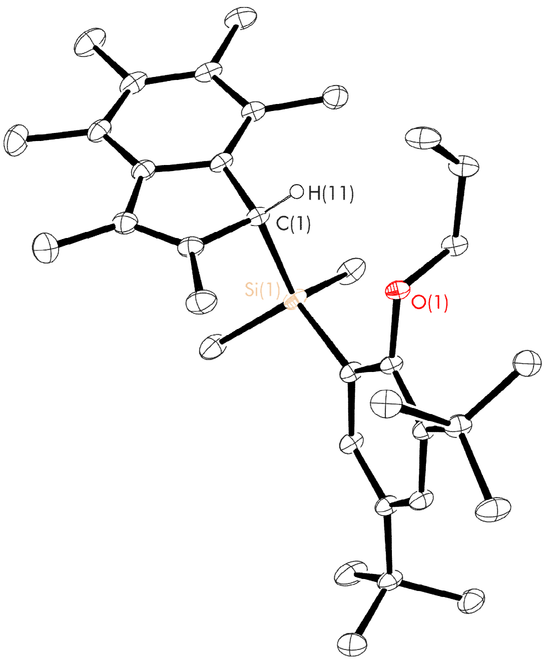

Figure S76 Solid-state ellipsoid plot of ^{Me_2}SB($^{^tBu_2}$ArOAllyl,I*)H (**P2**). All hydrogen atoms omitted for clarity except H(11) at C(1); thermal ellipsoids drawn at 30% probability.

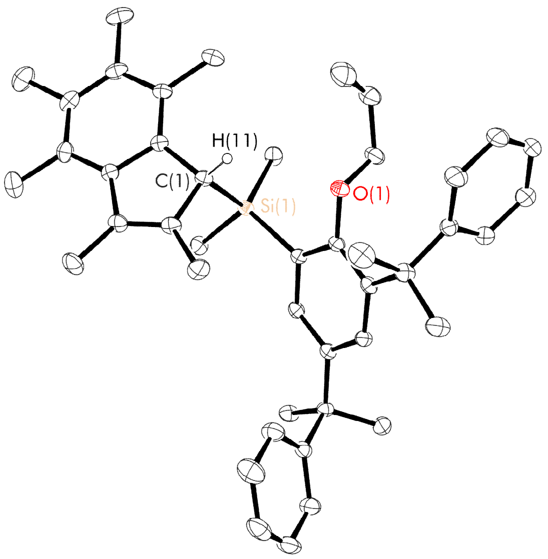

Figure S77 Solid-state ellipsoid plot of ^{Me_2}SB(^{Cumyl_2}ArOAllyl,I*)H (**P3**). All hydrogen atoms omitted for clarity except H(11) at C(1); thermal ellipsoids drawn at 30% probability.

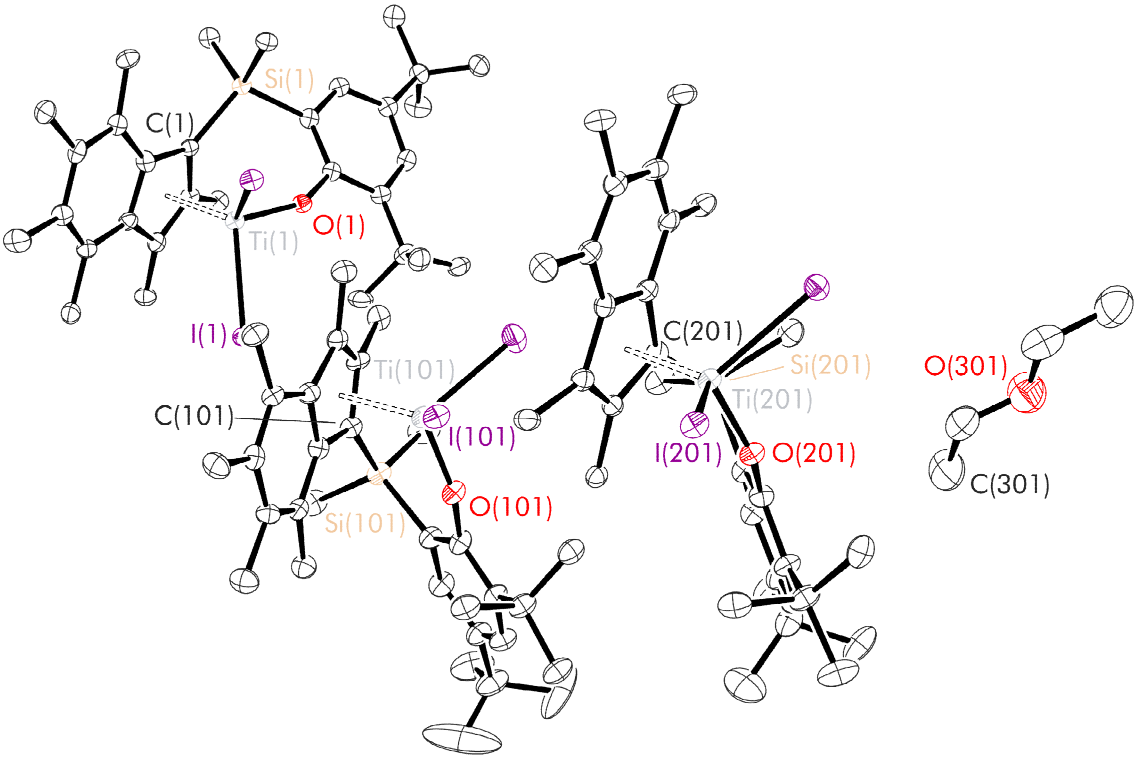

Figure S78 Solid-state ellipsoid plot of $^{Me2}SB(^{tBu2}ArO,I^*)TiI_2$ (**4**). Whole asymmetric unit (*Z*′ = 3) shown with (left to right) residue 1 containing Ti(1) (B-type conformation), 2 containing Ti(101) (A-type conformation), and 3 containing Ti(201) (B-type conformation). Residue 4 is an ether of crystallization. All hydrogen atoms omitted for clarity; thermal ellipsoids drawn at 30% probability.

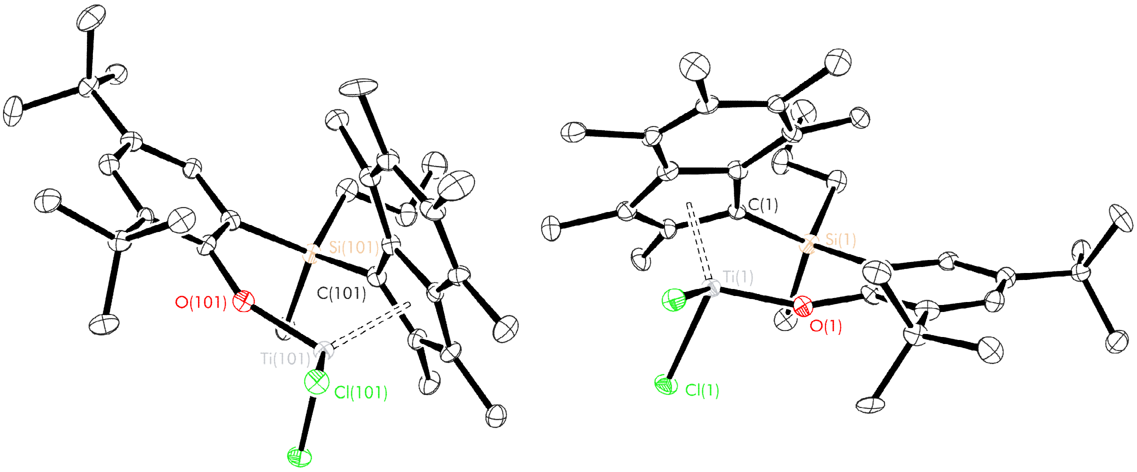

Figure S79 Solid-state ellipsoid plot of $^{Me,nPr}SB(^{tBu2}ArO,I^*)TiCl_2$ (**12**). Whole asymmetric unit (*Z*′ = 2) shown with both residues having similar structures. All hydrogen atoms omitted for clarity; thermal ellipsoids drawn at 30% probability.

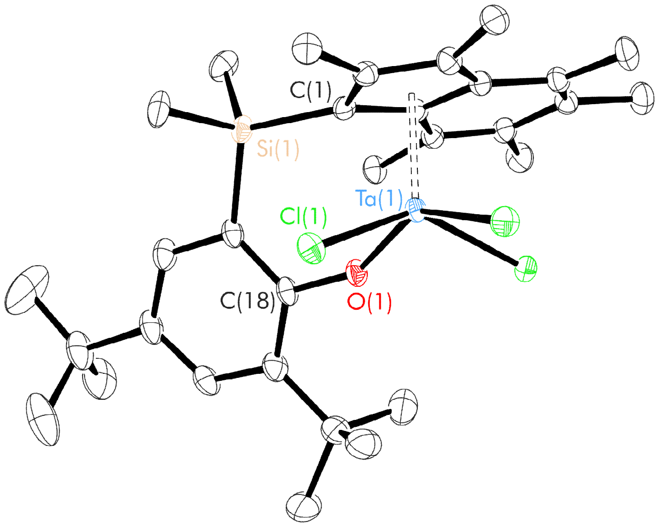

Ta(1)–O(1)	1.893(3)
Ta(1)–I_{cent}*	2.1134(19)
O(1)–Ta(1)–I_{cent}*	114.55(10)
C(1)–Si(1)–C(23)–C(18)	7.8(5)
TA'	56.5(4)
I*–ArO	66.65
Δ_{M-C}	0.330
τ_5	0.046

Figure S80 Solid-state ellipsoid plot and geometrical parameters (lengths in Å, angles in °) of ^{Me_2}SB(^{tBu_2}ArO,I*)$TaCl_3$ (**18**). All hydrogen atoms omitted for clarity; thermal ellipsoids drawn at 30% probability.

When compared to the analogous titanium complex **2**, **18** has longer metal bond lengths, as expected given the larger ionic radius of Ta^{5+} compared to that of four coordinate Ti^{4+} (0.78 *cf.* 0.56 Å).[1] Complex **18** also displays a much larger ring slip parameter (Δ_{M-C} = 0.330 Å) compared to **2** (Δ_{M-C} = 0.096 Å). The larger I*–ArO interplanar dihedral angle (66.66° *cf.* 46.31°), and smaller C(18)–C(23)–Si(1)–C(1) torsion angle (7.8° *cf.* 46.31°) of **18** compared to **2** suggests a less twisted ligand backbone, and shows that **18** is geometrically far more similar to the Ta PHENICS complex ^{Et_2}SB(^{tBu,Me}ArO,Cp*)$TaCl_3$ reported by Senda *et al.* (63.41° and 10.9° for the dihedral and torsion angles respectively).[2]

The Ta–O bond length (1.893(3) Å) is of a comparable length to that in the PHENICS analogue (1.875 Å), but shorter than those in both a bridged indenyl complex (OC_6H_2-2-{η^5-Ind}-4,6-^{t}Bu_2)Ta(NC_5H_4-4-Ph)Cl_3 (1.968(3) Å)[3] and in a non-bridged cyclopentadienyl-phenoxy complex Cp*Ta(OC_6H-2,6-Ph_2-3,5-^{t}Bu_2)Cl_3 (1.902(3) Å).[4] The three Ta–Cl bonds (mean 2.388 Å) are of comparable length to many Cp*L$TaCl_3$ complexes previously reported in the literature, where L is an ancillary ligand.[4-6] In **18**, Ta occupies the centre of a four legged "piano-stool" geometry. The geometry of a five-coordinate complex can be evaluated by the τ_5 parameter;[7] a value of 0 indicates a perfectly square pyramidal geometry whilst a τ_5 value of 1 indicates a perfectly trigonal bipyramidal geometry. For **18** τ_5 = 0.046, consistent with square planar coordination geometry.

2.2. Solid-G ligand parameters

Table S1 Calculations performed using Solid-G.[8] G(L) refers to the percentage of the sphere that is shielded by the PHENI* ligand.

Complex	Conformation type	Space-filling model	Solid-G plots		G(L) (%)	Equivalent Cone Angle (ECA) /°
**1**	B	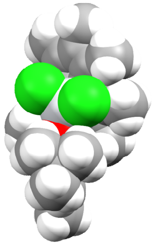	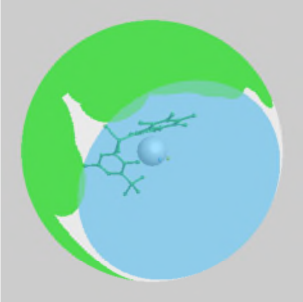	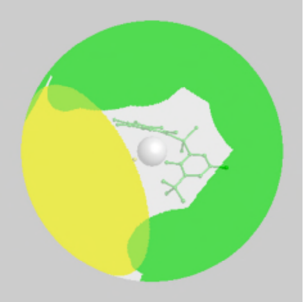	61.33	206.19
**2**	B	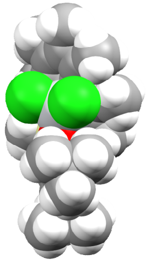	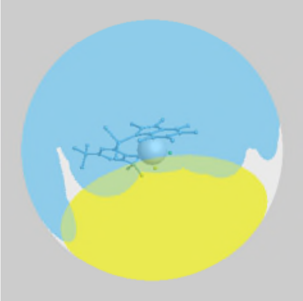	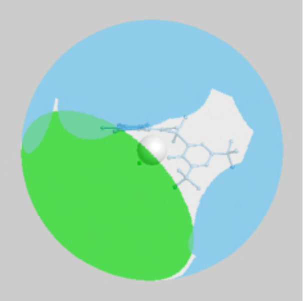	62.21	208.26

**3**	A	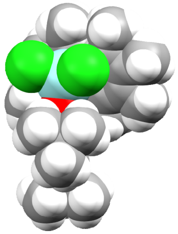	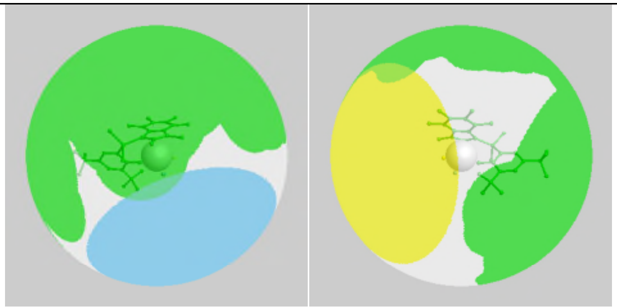	58.08	198.60
**4**	A	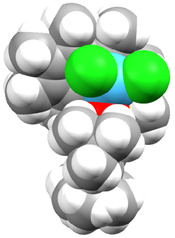	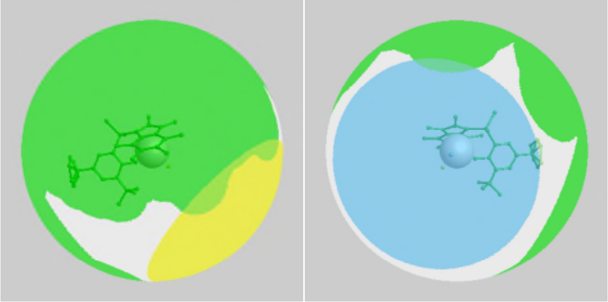	58.55	199.68
**5**	B	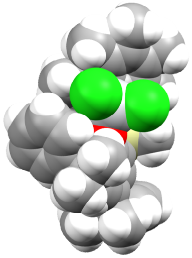	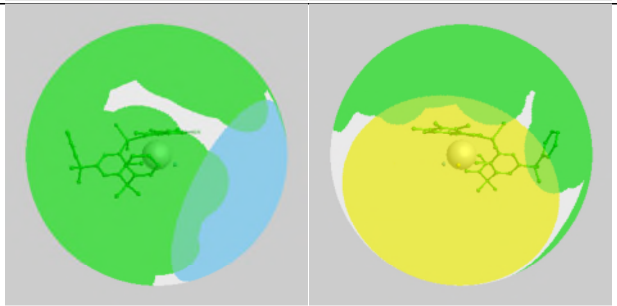	64.54	213.81

**6**	A	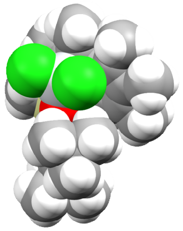	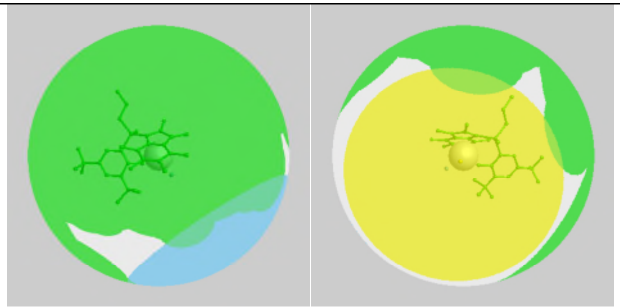	62.25	208.36
**7**	B	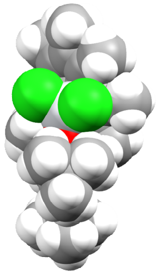	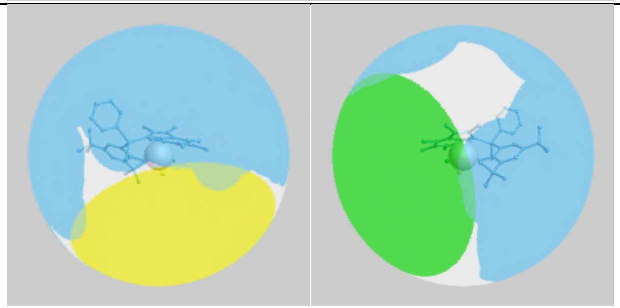	54.71	190.81
**8**	A	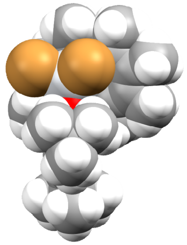	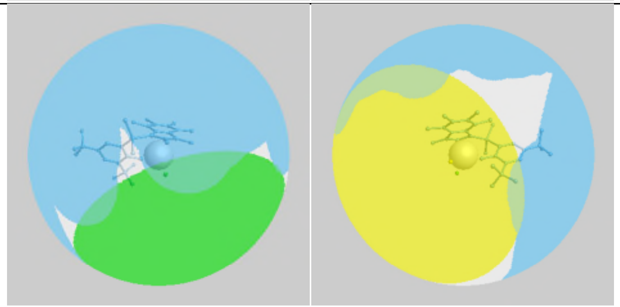	54.21	189.65

**9**-A	A	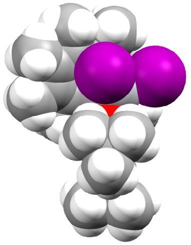	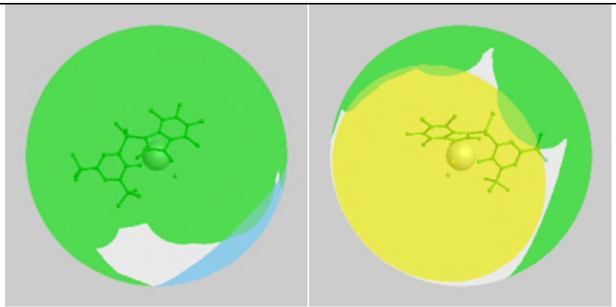	52.92	186.69
**9**-B	B	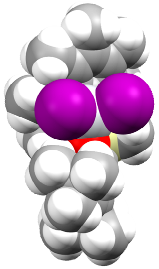	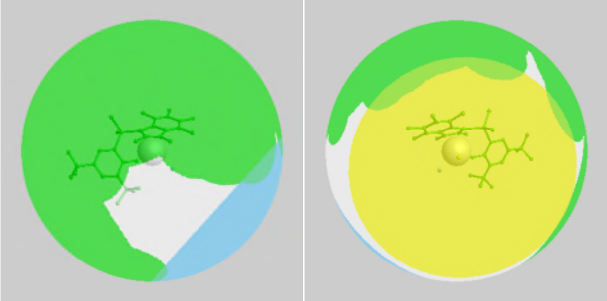	61.05	205.54
**10**	B	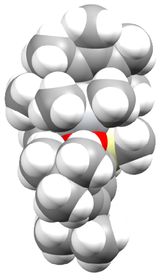	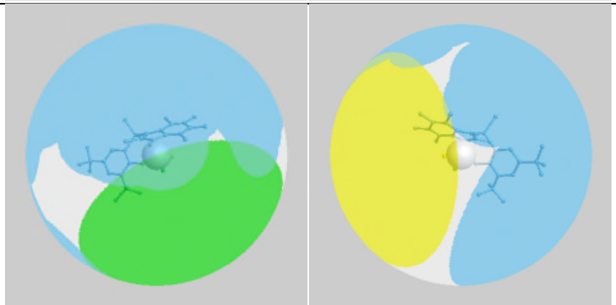	61.60	206.84

**11**	intermediate	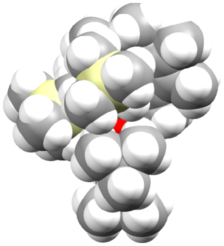	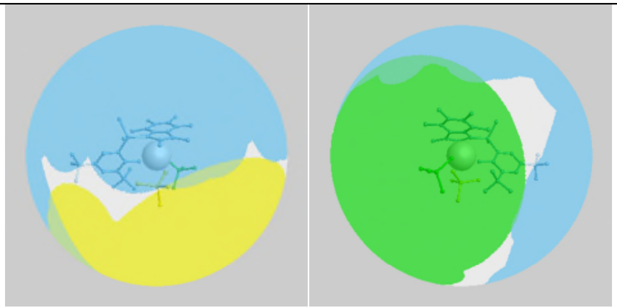	53.15	187.22
**13**	A	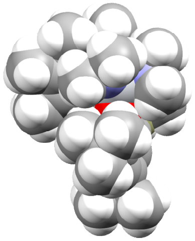	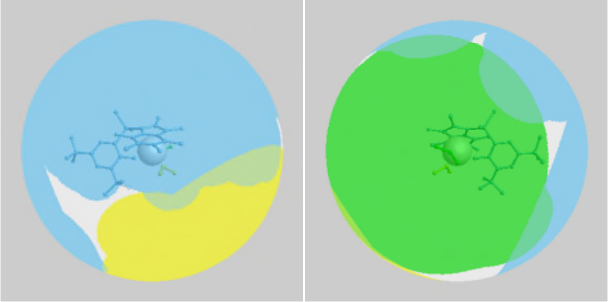	51.82	184.17
**15**	intermediate	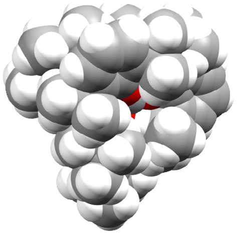	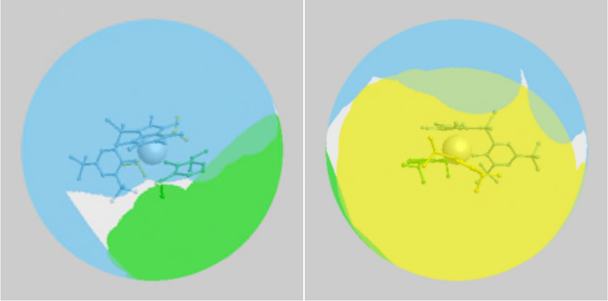	48.70	177.03

**A**	A	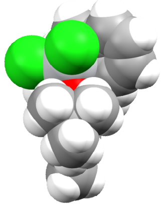	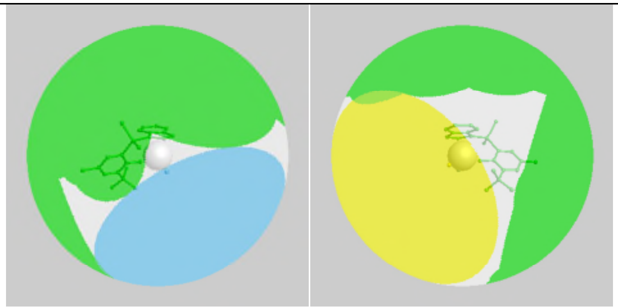	57.02	196.13

3. NMR spectra of cationic complexes

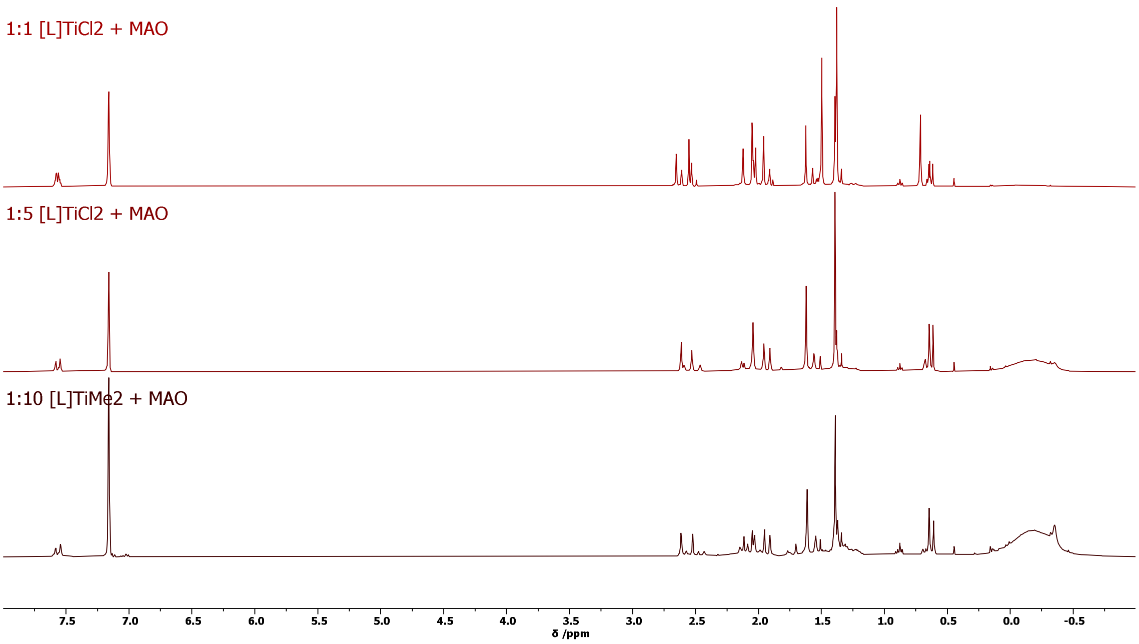

Figure S81 ^{1}H NMR spectra of the reaction between ^{Me_2}SB($^{{}^tBu_2}$ArO,I*)TiCl$_2$ (**2**) and MAO as a function of [MAO]/[Ti] (benzene-d_6, 400 MHz, 298 K).

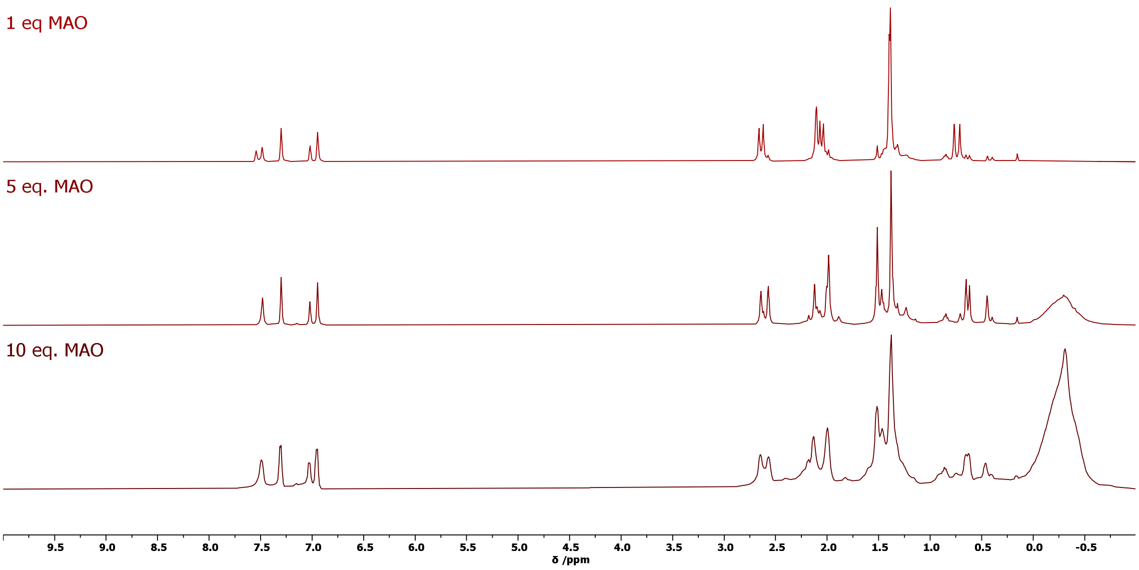

Figure S82 ^{1}H NMR spectra of the reaction between ^{Me_2}SB($^{{}^tBu_2}$ArO,I*)TiCl$_2$ (**2**) and MAO as a function of [MAO]/[Ti] (bromobenzene-d_5, 400 MHz, 297 K).

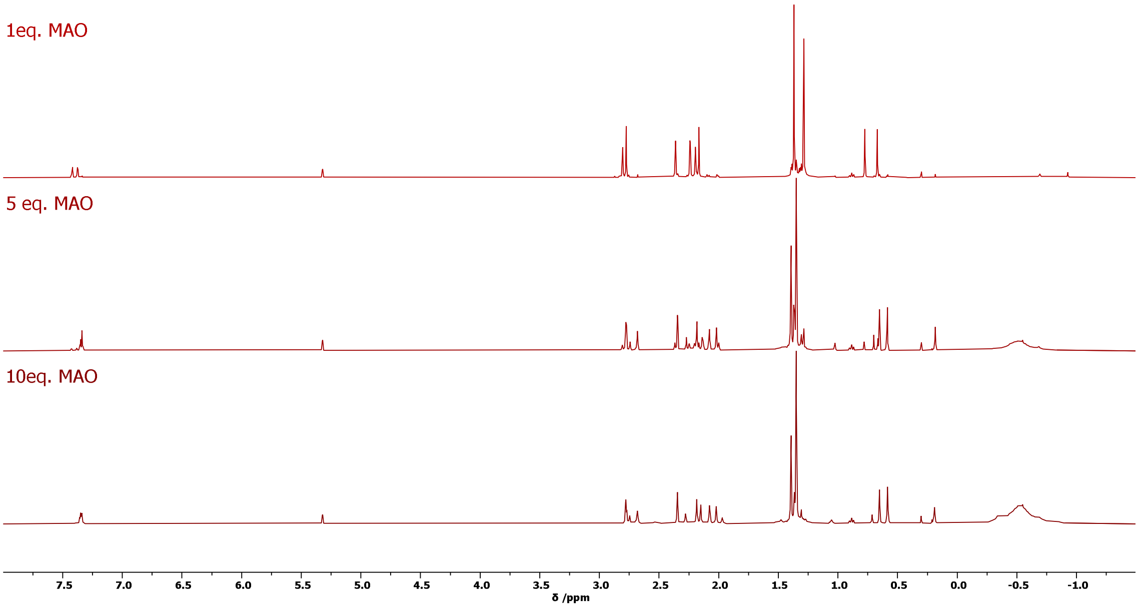

Figure S83 ^{1}H NMR spectra of the reaction between $^{Me_2}SB(^{^tBu_2}ArO,I^*)TiCl_2$ (**2**) and MAO as a function of [MAO]/[Ti] (dichloromethane-d_2, 400 MHz, 298 K).

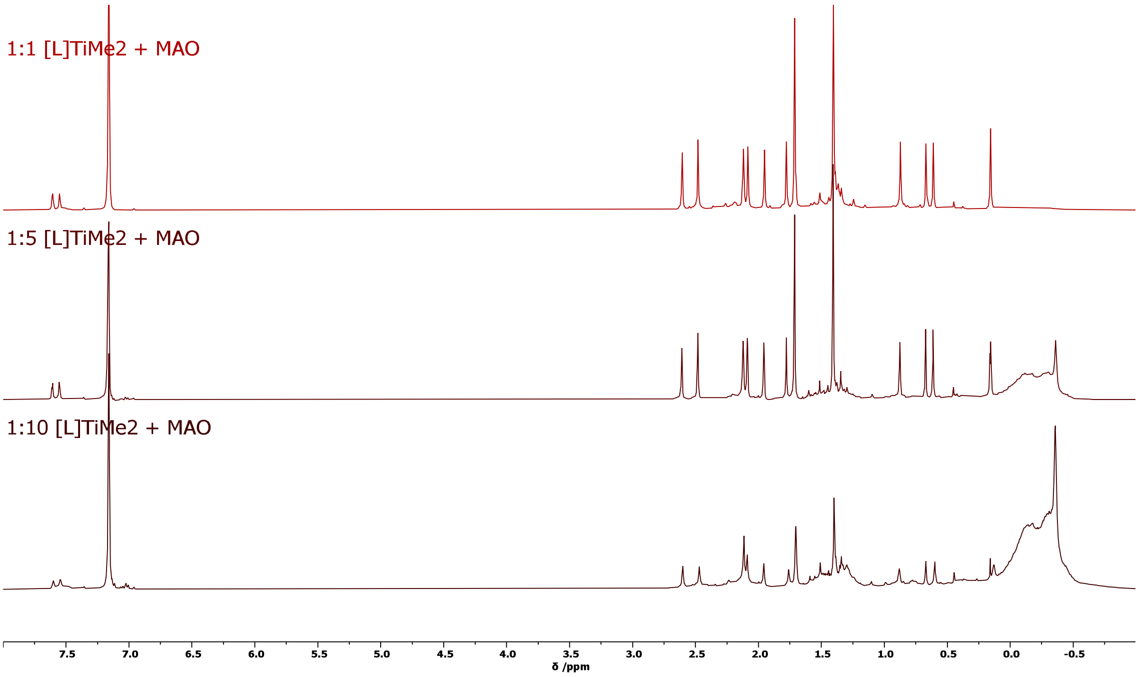

Figure S84 ^{1}H NMR spectra of the reaction between $^{Me_2}SB(^{^tBu_2}ArO,I^*)TiMe_2$ (**10**) and MAO as a function of [MAO]/[Ti] (benzene-d_6, 400 MHz, 298 K).

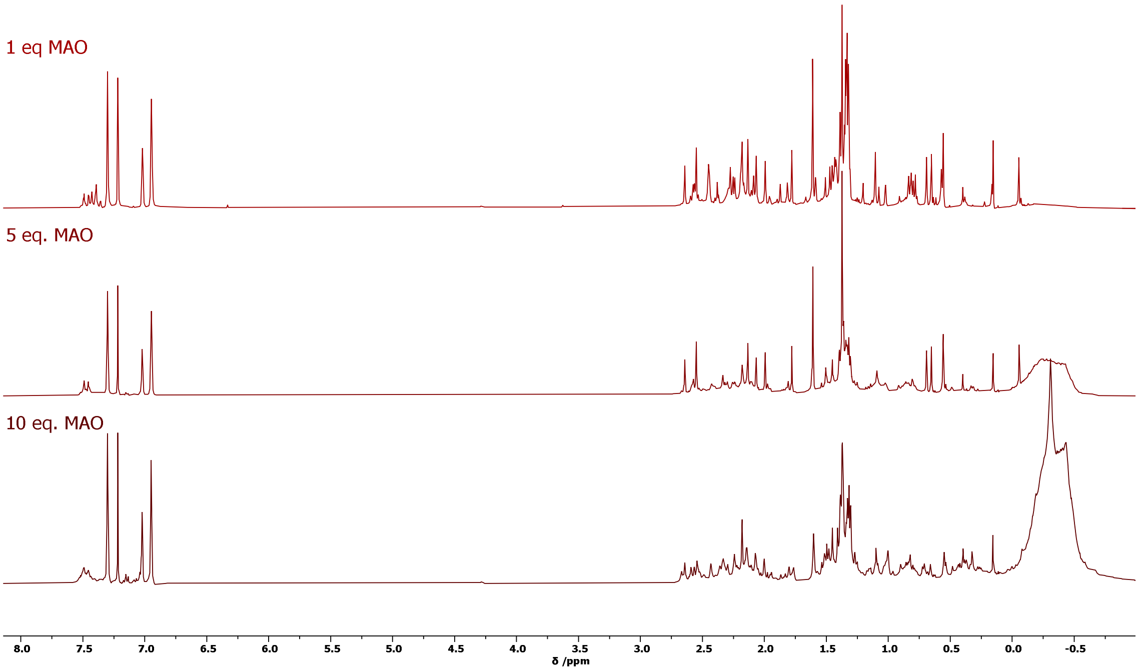

Figure S85 ^{1}H NMR spectra of the reaction between ^{Me_2}SB(^{tBu_2}ArO,I*)TiMe$_2$ (**10**) and MAO as a function of [MAO]/[Ti] (bromobenzene-d_5, 400 MHz, 298 K).

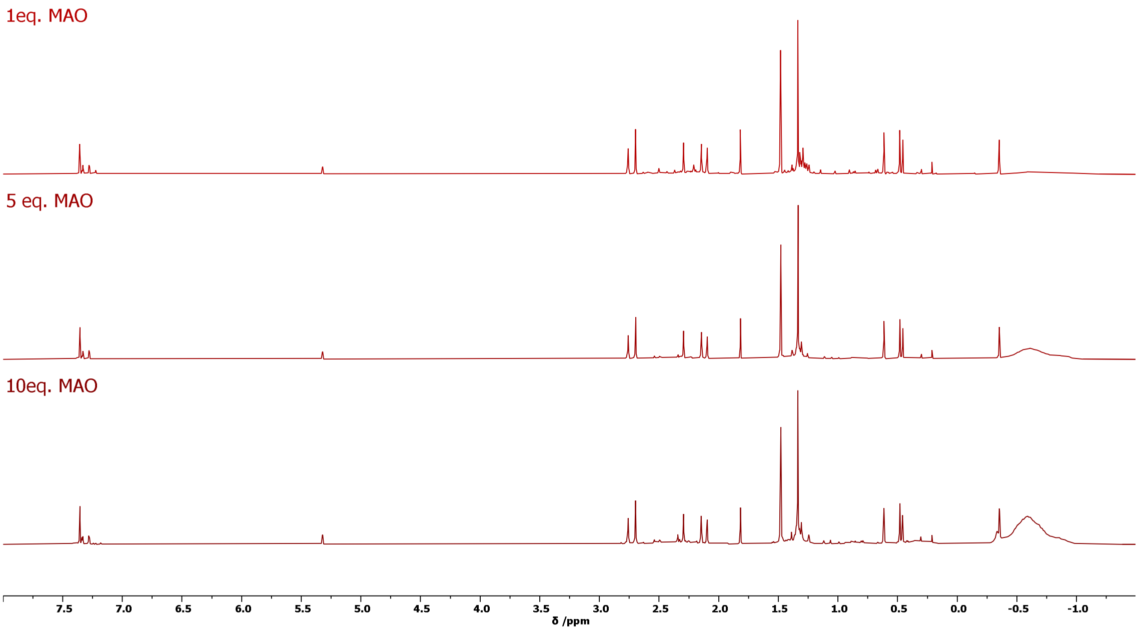

Figure S86 ^{1}H NMR spectra of the reaction between ^{Me_2}SB(^{tBu_2}ArO,I*)TiMe$_2$ (**10**) and MAO as a function of [MAO]/[Ti] (dichloromethane-d_2, 400 MHz, 297 K).

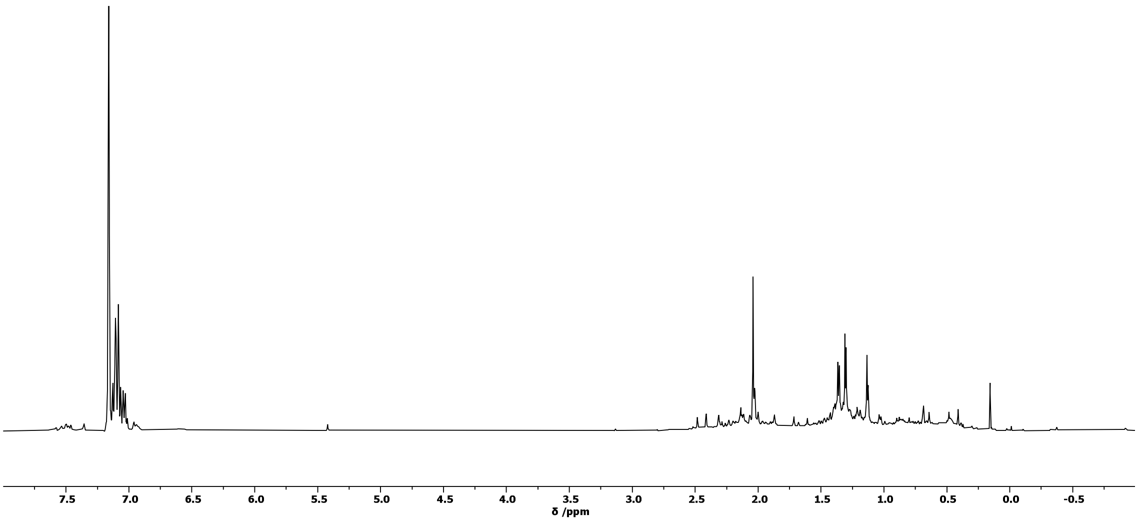

Figure S87 ^{1}H NMR spectra of the reaction between ^{Me_2}SB($^{^{t}Bu_2}$ArO,I*)TiMe$_2$ (**10**) and [Ph$_3$C][BAr$^F_4$] (benzene-d_6, 400 MHz, 297 K). A single ^{11}B resonance at δ –16 ppm and ^{19}F resonances at δ –132, –162, and –166 ppm were observed.

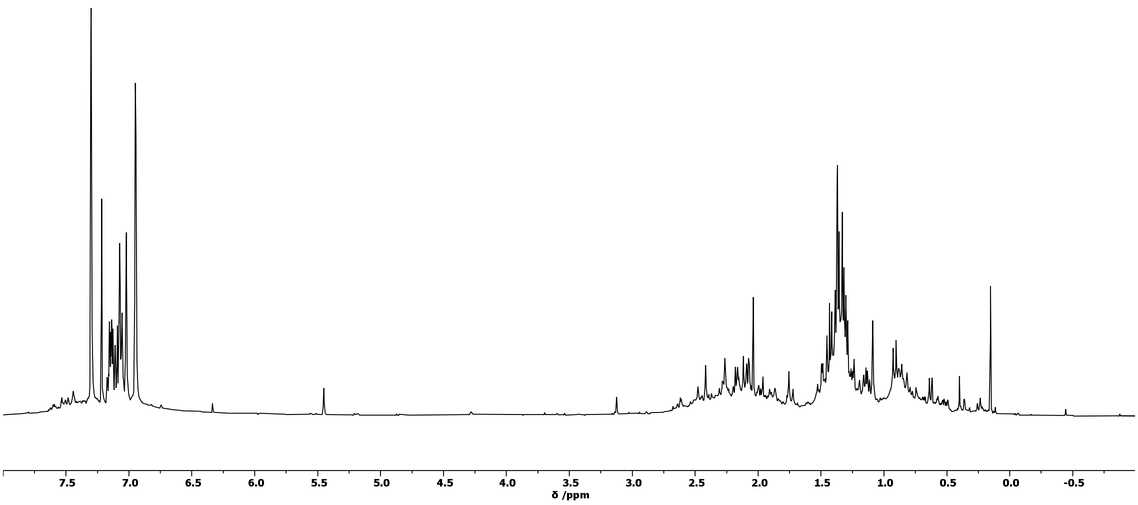

Figure S88 ^{1}H NMR spectra of the reaction between ^{Me_2}SB($^{^{t}Bu_2}$ArO,I*)TiMe$_2$ (**10**) and [Ph$_3$C][BAr$^F_4$] (bromobenzene-d_5, 400 MHz, 297 K).

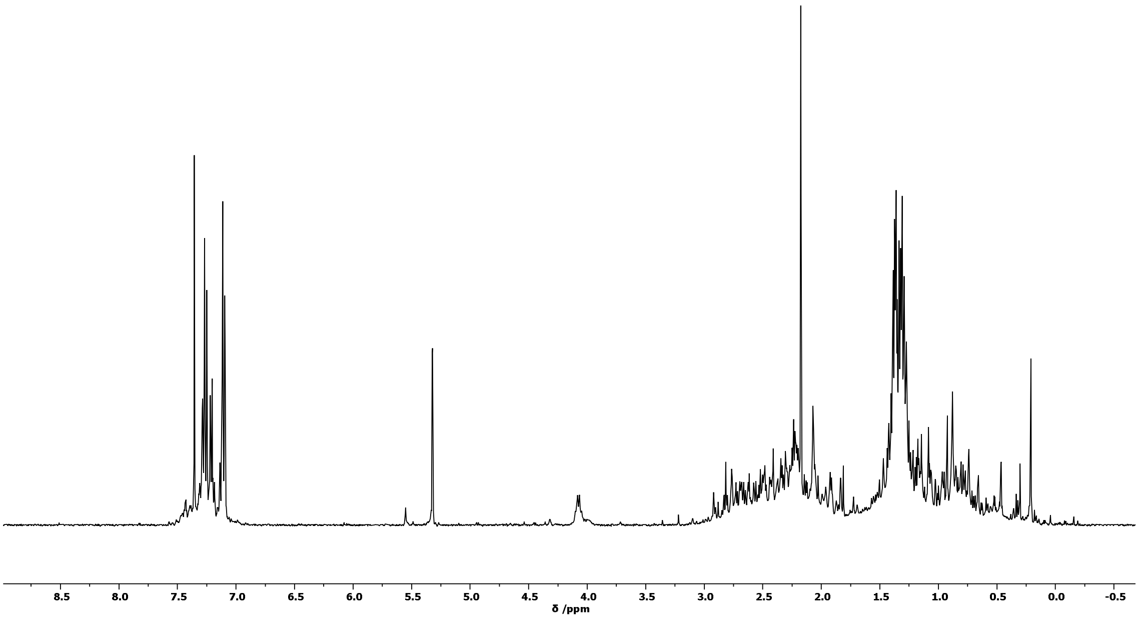

Figure S89 ^{1}H NMR spectra of the reaction between $^{Me_2}SB(^{tBu_2}ArO,I^*)TiMe_2$ (**10**) and $[Ph_3C][BAr^F_4]$ (dichloromethane-d_2, 400 MHz, 297 K). A single ^{11}B resonance at δ –17 ppm and ^{19}F resonances at δ –133, –164, and –167 ppm were observed.

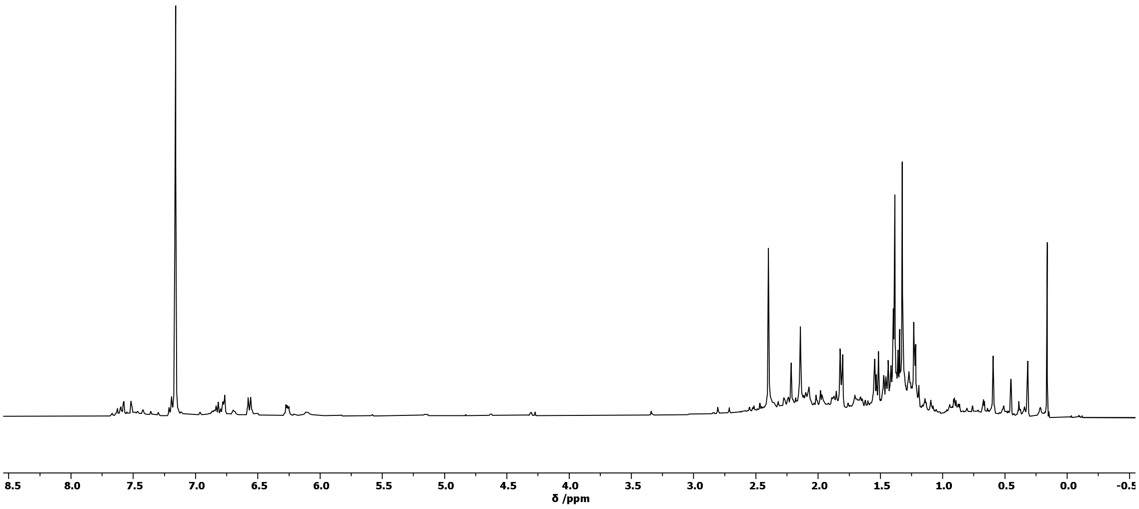

Figure S90 ^{1}H NMR spectra of the reaction between $^{Me_2}SB(^{tBu_2}ArO,I^*)TiMe_2$ (**10**) and $[PhNHMe_2][BAr^F_4]$ (benzene-d_6, 400 MHz, 298 K). ^{19}F resonances at δ –132, –162, and –166 ppm were observed.

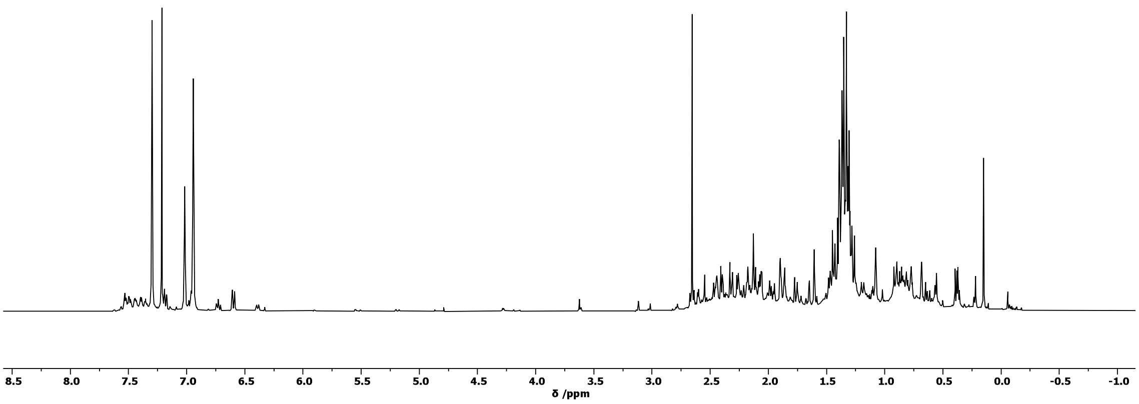

Figure S91 ^{1}H NMR spectra of the reaction between ^{Me2}SB(^{tBu2}ArO,I*)TiMe$_2$ (**10**) and [PhNHMe$_2$][BAr$^{F}_4$] (bromobenzene-d_5, 400 MHz, 298 K). ^{19}F resonances at δ –131, –162, and –166 ppm were observed.

4. ssNMR spectra of solid catalysts

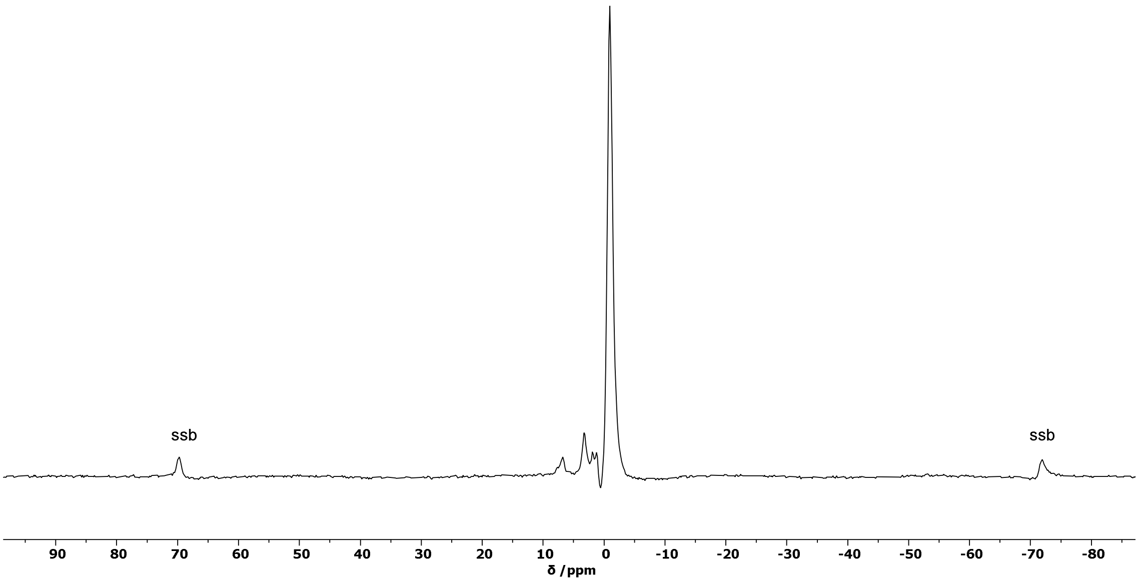

Figure S92 ^{1}H ssNMR spectrum of sMAO-^{Me2}SB(^{tBu2}ArO,I*)TiCl$_2$ (**2$_{sMAO}$**) (60 kHz, 850 MHz, 298 K).

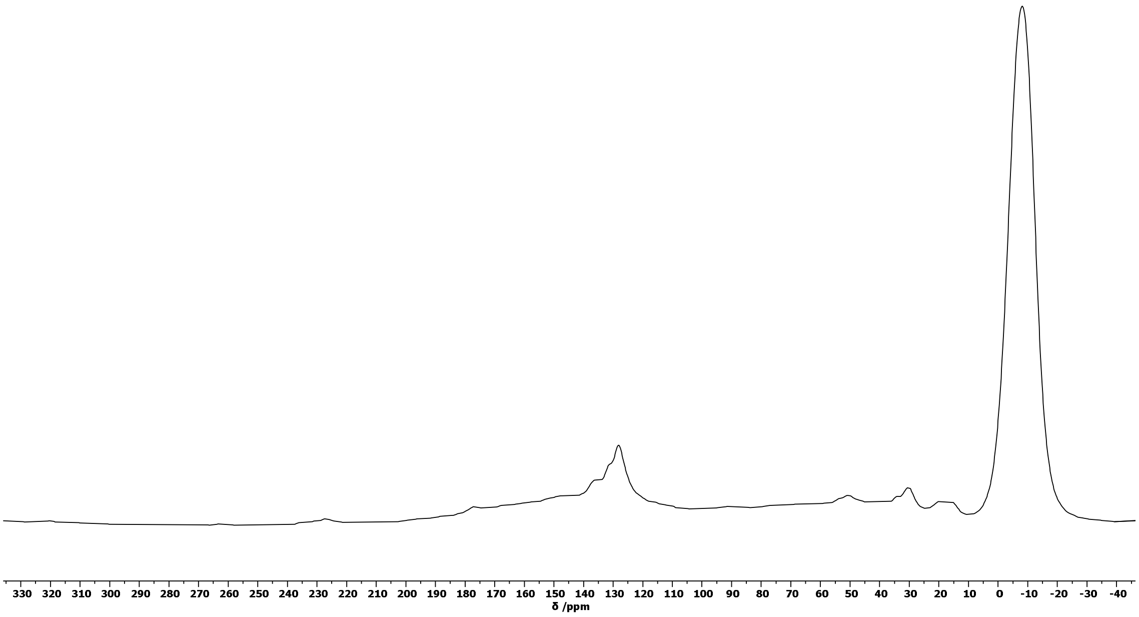

Figure S93 ^{13}C CPMAS ssNMR spectrum of sMAO-^{Me2}SB(^{tBu2}ArO,I*)$TiCl_2$ (**2$_{sMAO}$**) (10 kHz, 101 MHz, 298 K).

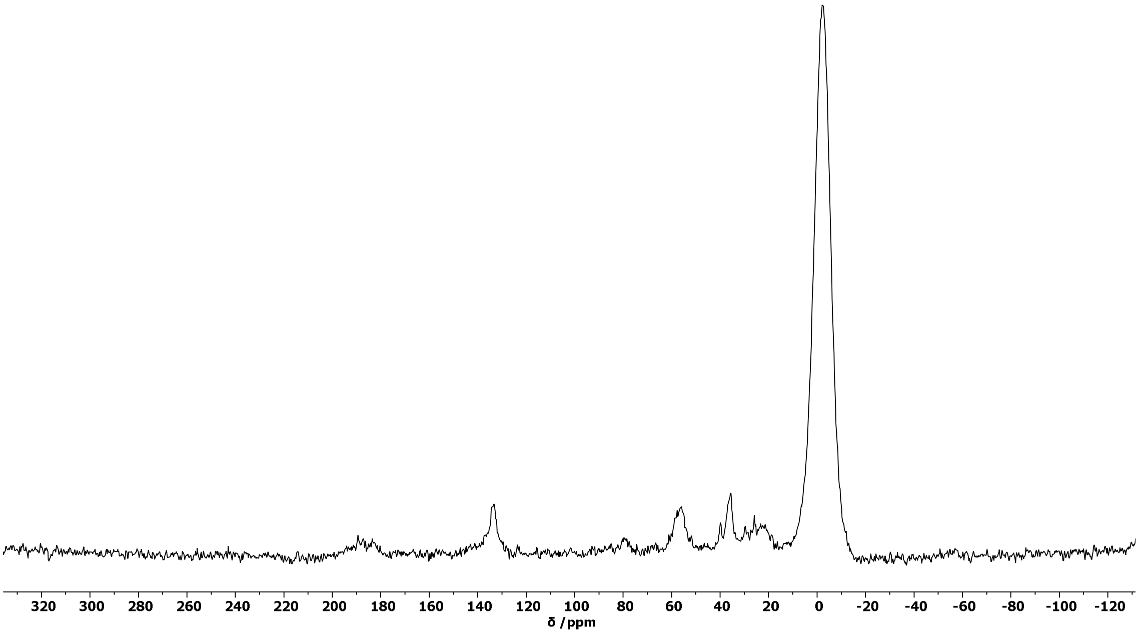

Figure S94 ^{13}C CPMAS ssNMR spectrum of sMAO-^{Me2}SB(^{tBu2}ArO,I*)$TiCl_2$ (**2$_{sMAO}$**) (60 kHz, 214 MHz, 298 K).

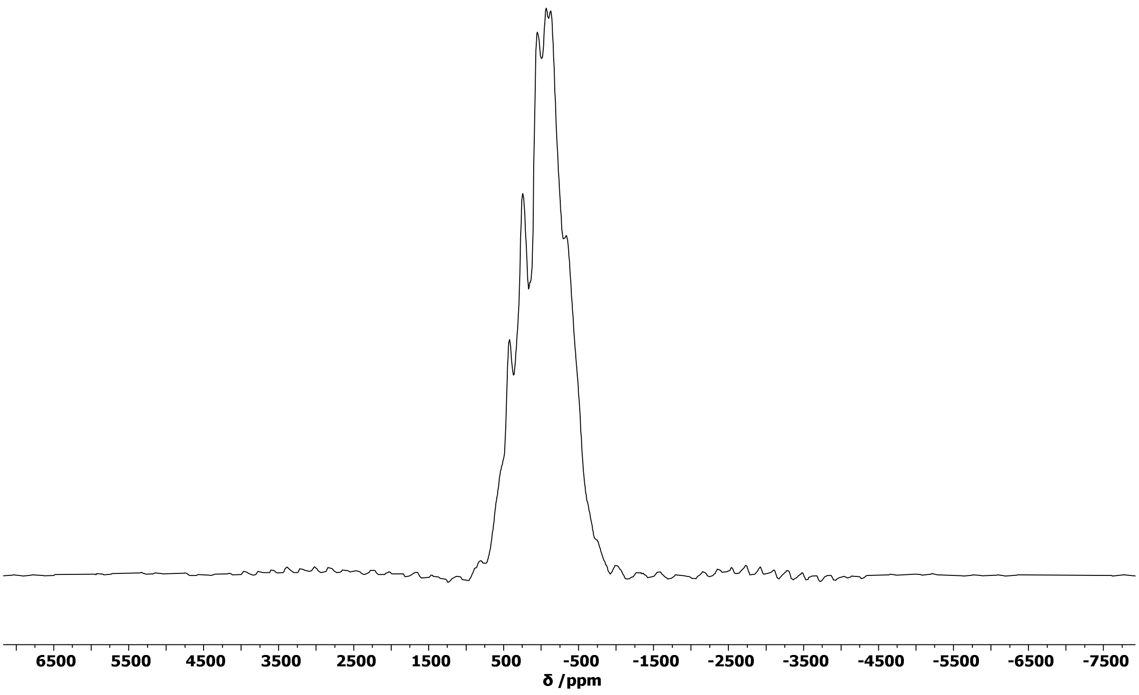

Figure S95 ^{27}Al Hahn echo ssNMR spectrum of sMAO-^{Me2}SB(^{tBu2}ArO,I*)$TiCl_2$ (**2**$_{\mathbf{sMAO}}$) (20 kHz, 104 MHz, 298 K).

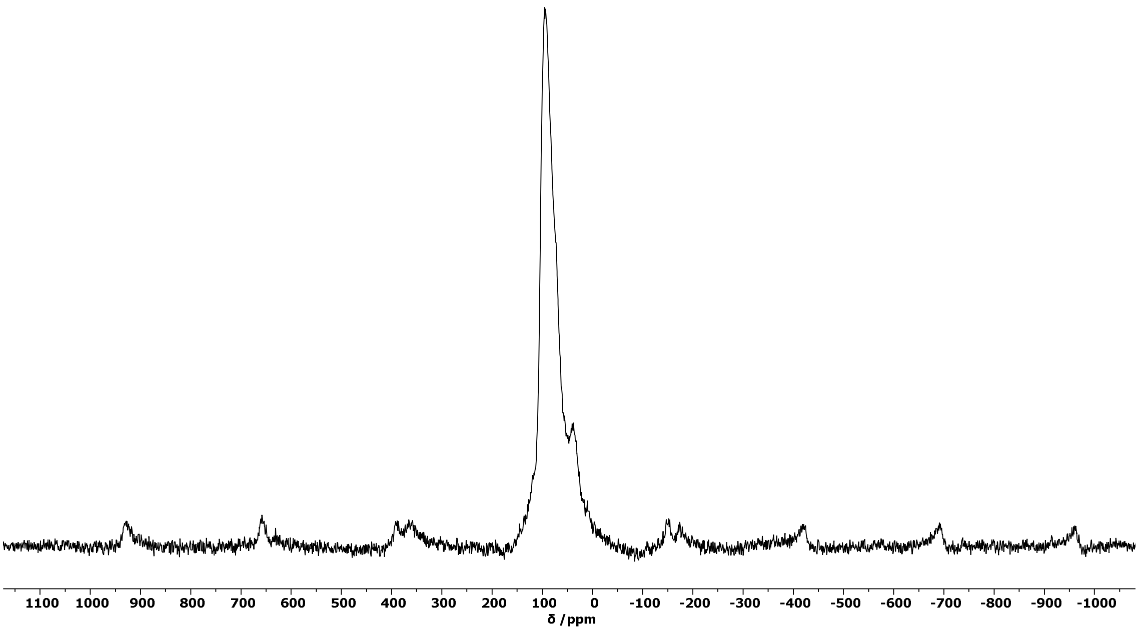

Figure S96 ^{27}Al DPMAS ssNMR spectrum of sMAO-^{Me2}SB(^{tBu2}ArO,I*)$TiCl_2$ (**2**$_{\mathbf{sMAO}}$) (60 kHz, 222 MHz, 298 K).

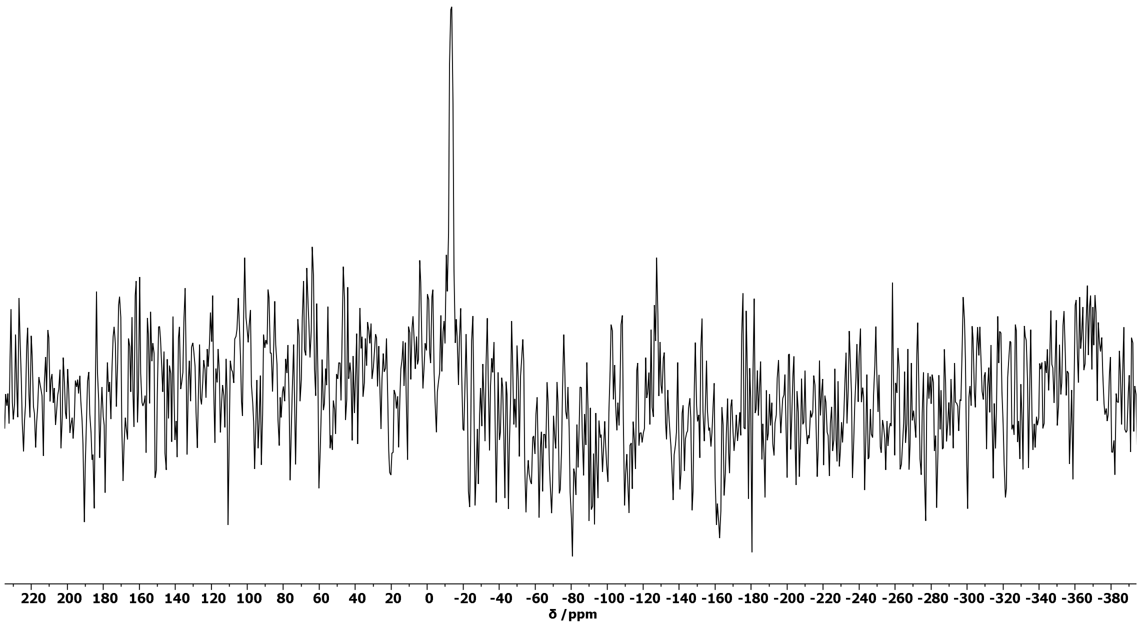

Figure S97 ^{29}Si CPMAS ssNMR spectrum of sMAO-^{Me2}SB(^{tBu2}ArO,I*)$TiCl_2$ (**2**$_{\mathbf{sMAO}}$) (10 kHz, 79 MHz, 298 K).

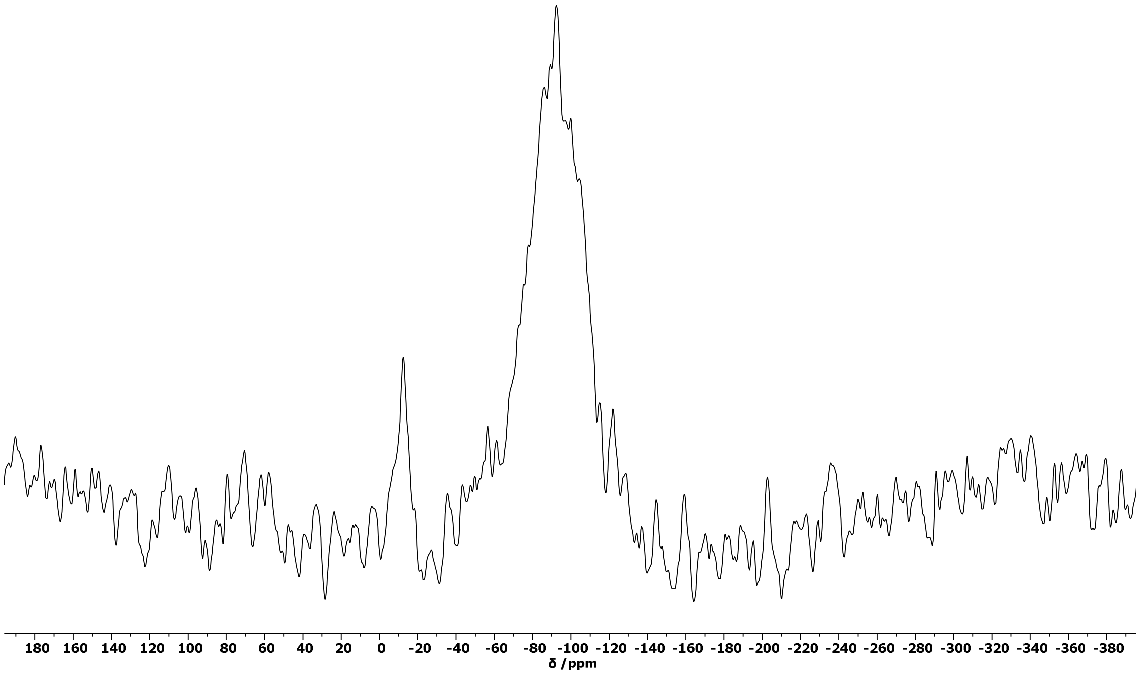

Figure S98 ^{29}Si CPMAS ssNMR spectrum of sMAO-^{Me2}SB(^{tBu2}ArO,I*)$TiCl_2$ (**2**$_{\mathbf{sMAO}}$) (60 kHz, 169 MHz, 298 K). The broad resonance at δ –90 ppm arises from residual silica impurities in the rotor.

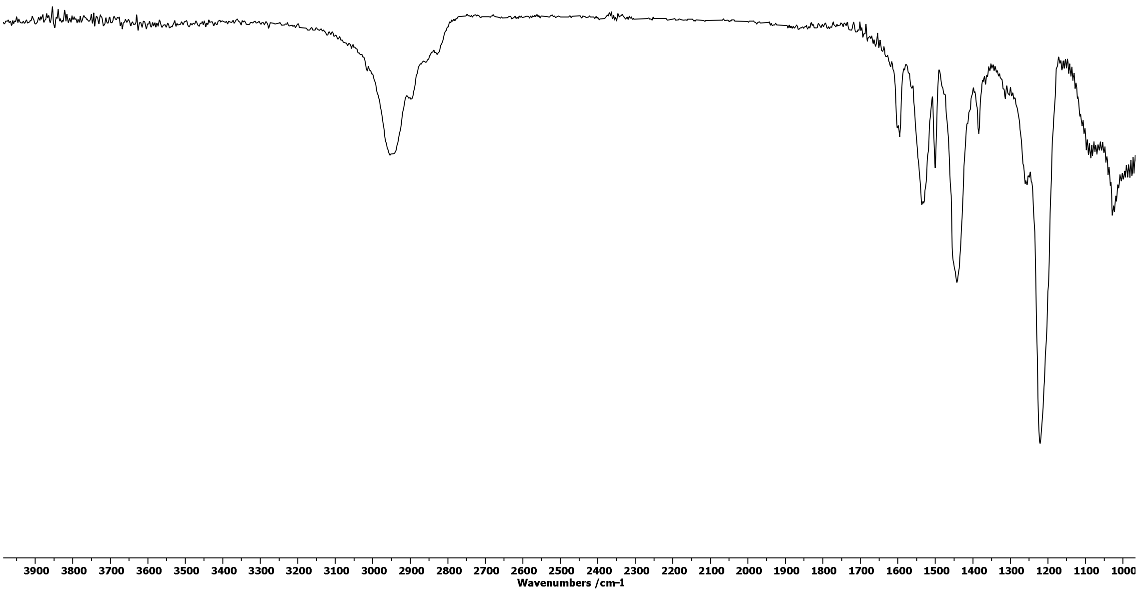

Figure S99 FTIR spectrum of sMAO-^{Me2}SB(^{tBu2}ArO,I*)$TiCl_2$ (**2**$_{\mathbf{sMAO}}$) (KBr).

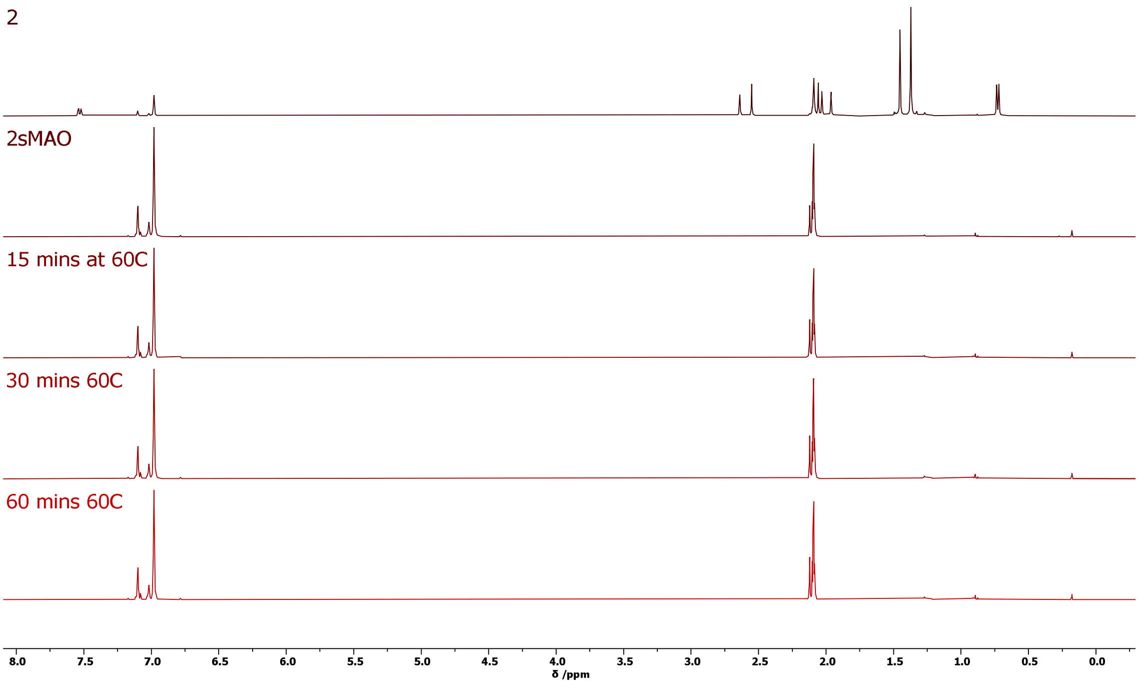

Figure S100 ^{1}H NMR spectrum of sMAO-^{Me2}SB(^{tBu2}ArO,I*)$TiCl_2$ (**2**$_{\mathbf{sMAO}}$) (toluene-d_8, 400 MHz, 298 K) demonstrating that no leaching occurs.

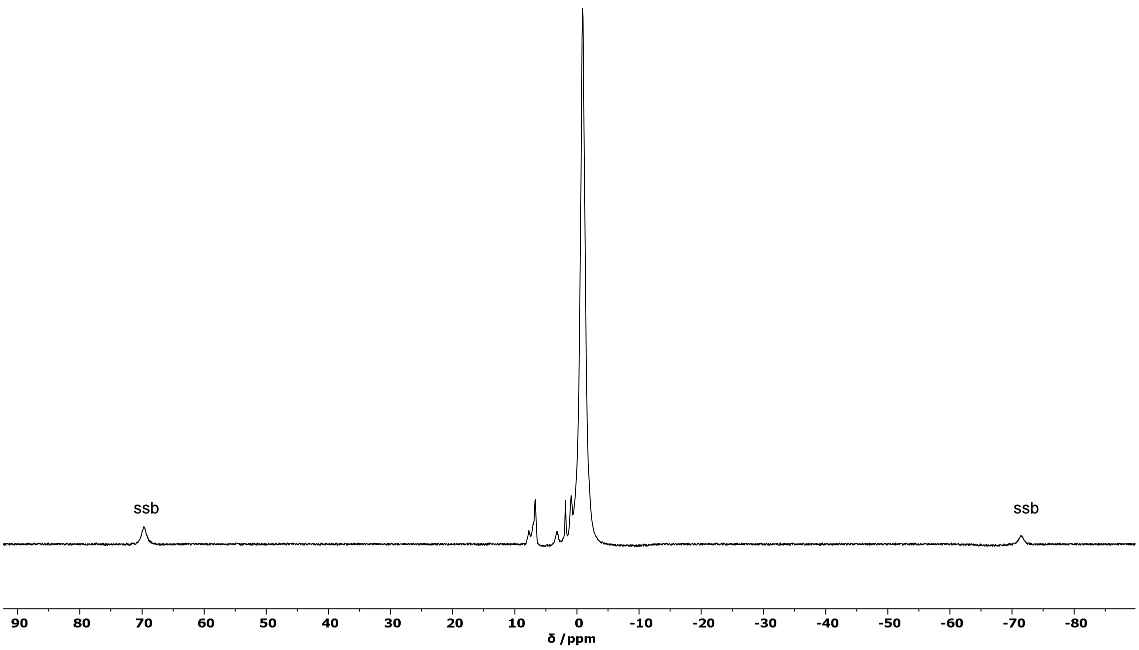

Figure S101 ^{1}H ssNMR spectrum of sMAO-^{Me2}SB(^{tBu2}ArO,I*)TiMe$_2$ (**10$_{sMAO}$**) (60 kHz, 850 MHz, 298 K).

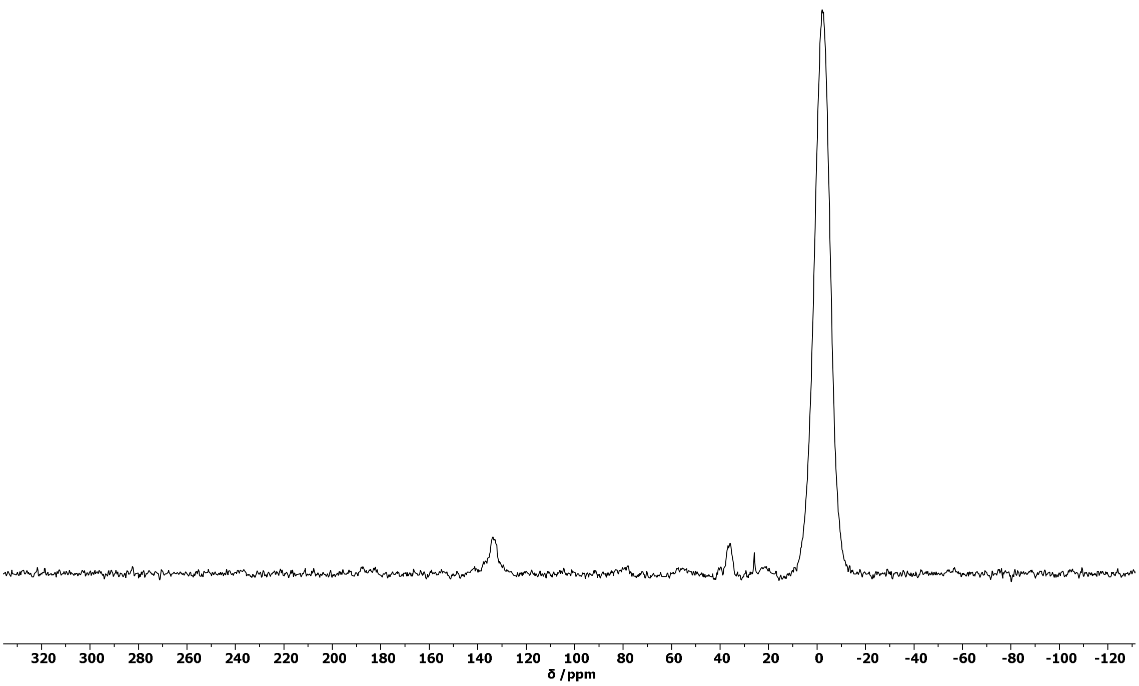

Figure S102 ^{13}C CPMAS ssNMR spectrum of sMAO-^{Me2}SB(^{tBu2}ArO,I*)TiMe$_2$ (**10$_{sMAO}$**) (60 kHz, 214 MHz, 298 K).

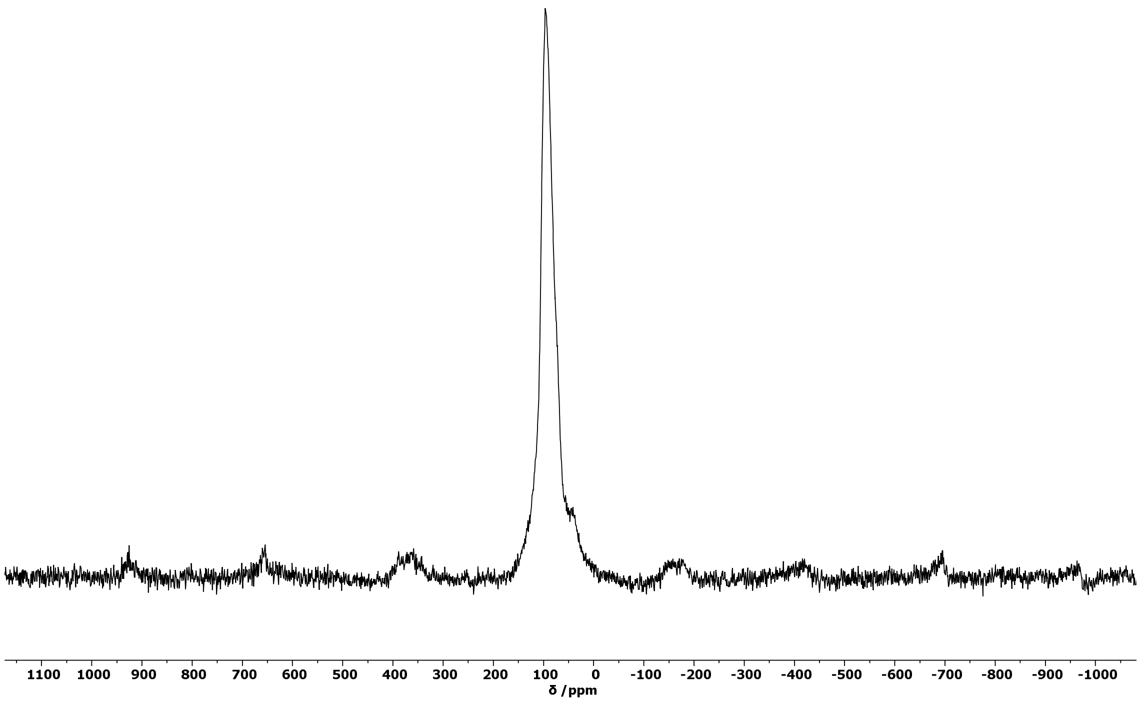

Figure S103 ^{27}Al DPMAS ssNMR spectrum of sMAO-^{Me2}SB(^{tBu2}ArO,I*)TiMe$_2$ (**10$_{sMAO}$**) (60 kHz, 222 MHz, 298 K).

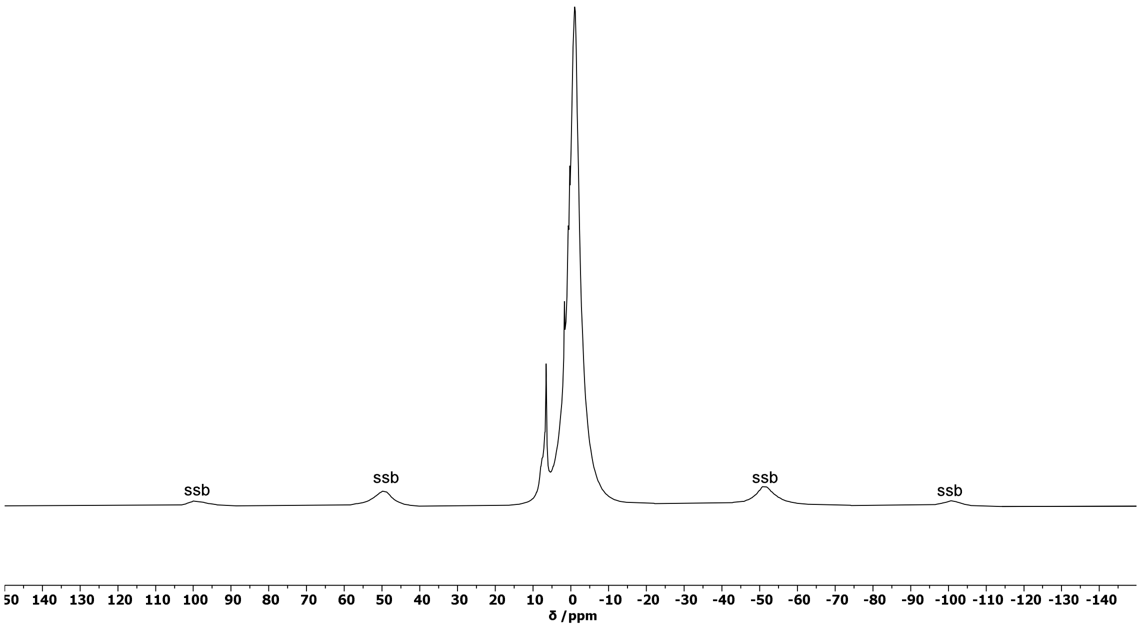

Figure S104 ^{1}H ssNMR spectrum of sMAO-^{Me2}SB(^{Cumyl2}ArO,I*)TiCl$_2$ (**5$_{sMAO}$**) (20 kHz, 400 MHz, 298 K).

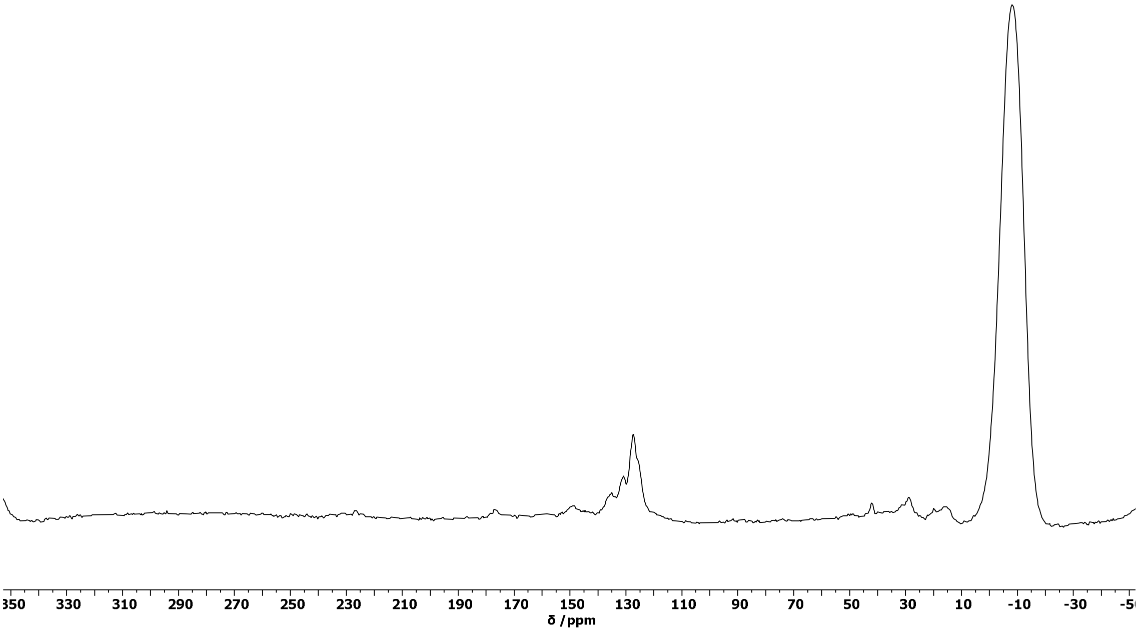

Figure S105 ^{13}C CPMAS ssNMR spectrum of sMAO-^{Me2}SB(^{Cumyl2}ArO,I*)$TiCl_2$ (**5_{sMAO}**) (10 kHz, 101 MHz, 298 K).

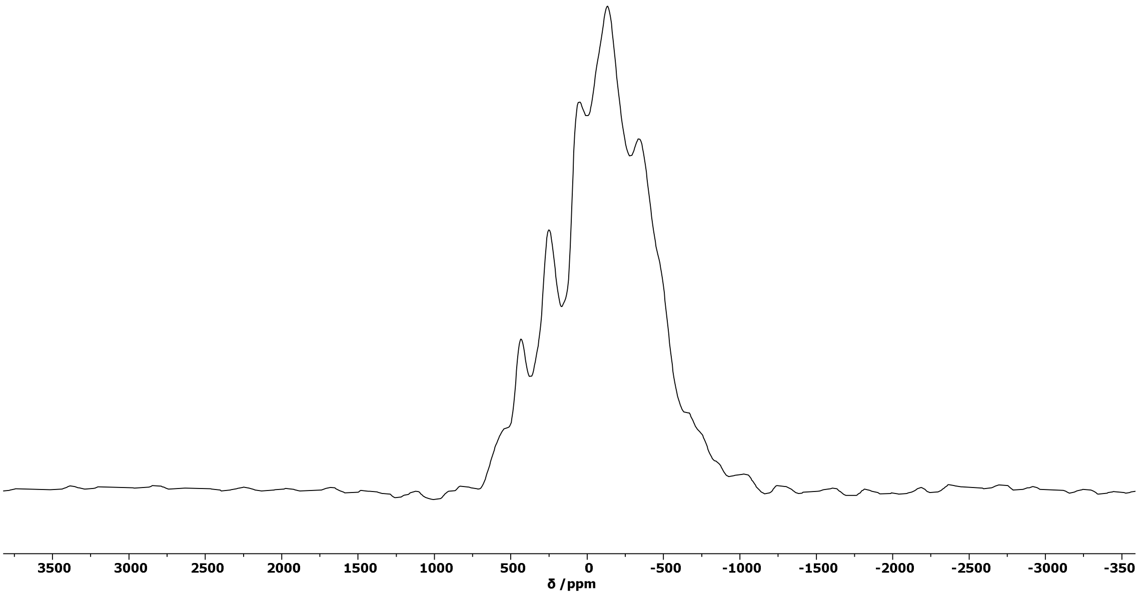

Figure S106 ^{27}Al Hahn echo ssNMR spectrum of sMAO-^{Me2}SB(^{Cumyl2}ArO,I*)$TiCl_2$ (**5_{sMAO}**) (20 kHz, 104 MHz, 298 K).

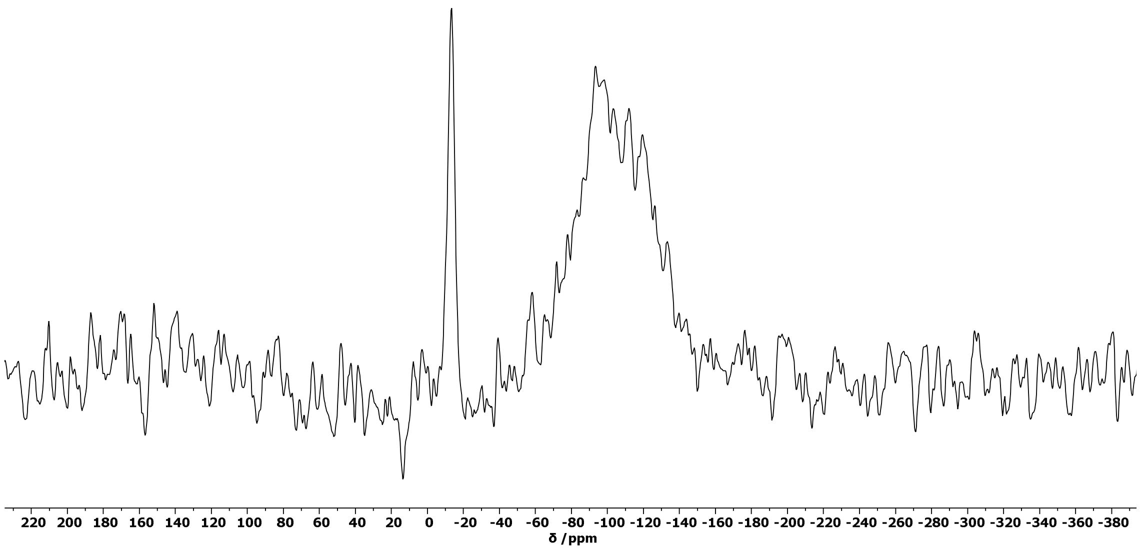

Figure S107 ^{29}Si CPMAS ssNMR spectrum of sMAO-^{Me2}SB(^{Cumyl2}ArO,I*)$TiCl_2$ ($\mathbf{5_{sMAO}}$) (10 kHz, 79 MHz, 298 K). The broad resonance at δ –100 ppm arises from residual silica impurities in the rotor.

5. Density Functional Theory calculations

Geometry optimizations were performed using ORCA 5.0.3[9-11] at the B3LYP level of theory[12-14] using def2–TZVP basis sets for heteroatoms.[15,16] The absence of imaginary frequencies confirms the convergence to true minima. Single point energy calculations were performed using the DFT–D3 dispersion correction,[17] and the Resolution of Identity approximation (RIJCOSX) was used to accelerate computations.[18,19] Cartesian coordinates were taken from solid-state X-ray crystal structures, or modifications thereof. A representative input file is:

```
#geometry optimization and frequency calculation of Atype_PHENIstar_Cl2

!RKS B3LYP D3ZERO RIJCOSX SlowConv TightSCF def2-SVP Normalprint Opt Freq PAL8

%basis  NewGTO Ti "def2-TZVP" end
        NewGTO Si "def2-TZVP" end
        NewGTO O "def2-TZVP" end
        NewGTO Cl "def2-TZVP" end
end

*xyz 0 1
[Cartesian coordinates]
```

A text file (.xyz format) of all computed molecule Cartesian coordinates in a format for convenient visualization is available. Calculated single point Gibbs free energies (298.15 K) are tabulated below:

Table S2 Optimized geometries and calculated single point Gibbs free energies (298.15 K) of PHENICS complex **A** and PHENI* complexes **1**–**15**.

**Complex**	**Experimental TA' (scXRD) (°)**	**A geometry** Optimized TA' (°)	G (kcal mol^{-1})	**B geometry** Optimized TA' (°)	G (kcal mol^{-1})	Δ(B–A) (kcal mol^{-1})
**A**	71.6	89.70	−1948335	41.77	−1948333	2.065
**1**	31.8	84.21	−2022200	36.92	−2022199	1.201
**2**	33.0	84.74	−2096054	37.13	−2096052	1.281
**3**	80.2	84.39	−1592593	35.81	−1592592	1.401
**4**	79.2	86.35	−1593199	36.4	−1593198	1.281
**5**	42.9	86.16	−2336312	40.58	−2336310	1.927
E–**6**	*n.d.*	84.96	−2145289	35.77	−2145286	2.242
Z–**6**	86.4	84.35	−2145287	36.77	−2145286	0.746
E–**7**	35.8	84.37	−2216181	39.89	−2216180	0.658
Z–**7**	*n.d.*	84.95	−2216181	36.71	−2216181	0.714
**8**	78.8	82.57	−4748975	38.7	−4748974	0.709
**9**	48.5	78.15	−1892166	40.44	−1892165	0.473
**10**	33.8	86.96	−1568425	35.59	−1568424	0.830
**11**	65.4	63.58	−2080960	39.3	−2080960	−0.191
**12**	*n.d.*	80.93	−1857922	45.59	−1857927	−4.180
**13**	78.5	77.06	−1687124	45.91	−1687122	2.213
**14**	*n.d.*	94.46	−1712133	33.18	−1712132	0.567
**15**	62.5	75.13	−2198584	39.3	−2198582	1.434

Table S3 Optimized geometries and calculated single point Gibbs free energies (298.15 K) of the methyl cations ([[L]TiMe]$^+$) and ethylene π-coordination complexes ([[L]TiMe]$^+$·(C_2H_4)) derived from PHENI* and Indenyl-PHENICS precatalysts.

**Complex**	**A geometry** Optimized TA' (°)	G (kcal mol^{-1})	**B geometry** Optimized TA' (°)	G (kcal mol^{-1})	Δ(B–A) (kcal mol^{-1})
[^{Me2}SB(^{tBu2}ArO,I*)TiMe]$^+$	87.94	−1543293	33.47	−1543297	−4.408
[^{Me2}SB(^{tBu2}ArO,I*)TiMe]$^+$·(C_2H_4)	81.55	−1592532	38.12	−1592534	−2.101
[^{Me2}SB(^{tBu,Me}ArO,Ind)TiMe]$^+$	71.80	−1321717	39.05	−1321719	−1.654
[^{Me2}SB(^{tBu,Me}ArO,Ind)TiMe]$^+$·(C_2H_4)	84.20	−1370957	42.85	−1370958	−0.128

6. Homogeneous polymerization data

Table S4 Solution-phase polymerization data using selected PHENI* catalysts. Polymerization conditions: 2 bar ethylene, 50 mL hexanes, $[Al_{MAO}]_0/[M]_0 = 1000$ (M = Ti, Ta) or $[Ti]_0:[TB]_0:[TIBA]_0 = 1:1:200$.

Complex	Temperature /°C	Time /mins	Activity /kg mol_M^{-1} h^{-1} bar^{-1}	M_w /kDa	*Đ*
**2**/MAO	30	5.00	1079 ± 53	694.6	3.0
	40	5.00	1474 ± 53	750.1	2.8
	50	5.00	2586 ± 914	708.9	2.7
	60	5.00	3842 ± 825	527.4	3.6
	70	5.00	4774 ± 707	607.6	3.6
	80	5.00	4501 ± 1247	410.0	3.3
	90	5.00	3602 ± 618	362.1	3.4
**10**/TB	30	0.93	14195 ± 3932	2557.2	3.1
	40	0.83	16188 ± 1328	2242.8	3.3
	50	0.52	26976 ± 1699	1326.5	3.4
	60	0.46	22475 ± 6342	2554.3	2.6
	70	0.66	17943 ± 5495	1422.9	3.5
	80	0.57	12218 ± 1718	2577.6	3.5
	90	0.75	14759 ± 3787	2360.0	3.6
**5**/MAO	30	1.23	17575 ± 1599	1699.8	3.5
	40	0.91	23336 ± 1223	1335.7	3.0
	50	1.03	20011 ± 4694	1005.0	3.9
	60	0.60	22658 ± 3273	617.9	3.4
	70	0.97	23158 ± 784	494.3	3.6
	80	1.37	12711 ± 5261	467.6	3.1

	90	0.85	16017 ± 5239	466.2	3.2
**18**/MAO	30	30.00	68.6 ± 1.0	*n.d.*	*n.d.*
	40	30.00	66.9 ± 9.0	*n.d.*	*n.d.*
	50	30.00	82.4 ± 14.9	*n.d.*	*n.d.*
	60	30.00	63.4 ± 2.0	*n.d.*	*n.d.*
	70	30.00	68.3 ± 3.0	*n.d.*	*n.d.*
	80	30.00	74.6 ± 4.0	*n.d.*	*n.d.*
	90	30.00	73.2 ± 2.0	*n.d.*	*n.d.*

7. Heterogeneous polymerization data

Table S5 Slurry-phase polymerization data using selected PHENI* catalysts. Polymerization conditions: 2 bar ethylene, 10 mg catalyst, either 150 mg TIBA or [Ti]/[TB] = 1, 50 mL hexanes.

Catalyst	Temperature /°C	Time /mins	Activity /kg mol_M^{-1} h^{-1} bar^{-1}	M_w /kDa	*Đ*
$\mathbf{1}_{sMAO}$/TIBA	50	30	3078 ± 34	2513.8	2.8
	60	30	3231 ± 150	2087.9	3.0
	70	30	2776 ± 190	1876.4	3.8
	80	30	2674 ± 70	1494.5	5.1
	90	30	1723 ± 192	1313.6	5.7
$\mathbf{2}_{sMAO}$/TIBA	30	30	2834 ± 338	3376.0	3.4
	40	30	3518 ± 129	3117.2	4.4
	50	30	3614 ± 123	2352.6	4.4
	60	30	3719 ± 106	2087.5	5.2
	70	30	3367 ± 151	2033.7	5.5
	80	30	2450 ± 121	1550.9	6.9
	90	30	2180 ± 17	1516.9	6.4
$\mathbf{2}_{sMAO}$/TB	30	30	192 ± 27	1797.7	3.0

	40	30	256 ± 71	1963.0	2.5
	50	30	392 ± 21	2266.7	2.8
	60	30	102 ± 41	972.8	3.2
	70	30	118 ± 14	1041.8	3.4
	80	30	154 ± 23	1050.7	3.3
	90	30	111 ± 51	1002.4	3.4
**3**$_{sMAO}$/TIBA	50	30	353 ± 40	162.0 / 2110.8 (87:13)	6.0 / 1.9
	60	30	338 ± 14	120.0 / 1303.2 (80:20)	3.8 / 3.4
	70	30	265 ± 3	163.0 / 1367.3 (81:19)	4.6 / 2.7
	80	30	216 ± 44	141.0 / 1193.4 (79:21)	3.4 / 3.4
	90	30	212 ± 1	254.0 / 1664.3 (86:14)	7.9 / 2.0
**5**$_{sMAO}$/TIBA	50	30	2114 ± 2	1862.5	3.5
	60	30	1983 ± 39	1777.0	3.5
	70	30	1597 ± 126	1392.8	3.7
	80	30	1317 ± 297	1108.2	3.9
	90	30	1022 ± 154	873.5	3.7
**6**$_{sMAO}$/TIBA	50	30	3662 ± 430	2810.1	2.5
	60	30	3897 ± 23	2337.0	3.1
	70	30	3767 ± 358	2052.3	4.0
	80	30	3453 ± 16	1728.7	4.4
	90	30	2361 ± 619	1346.1	5.6
**7**$_{sMAO}$/TIBA	50	30	3840 ± 320	1596.2	3.2

	60	30	3705 ± 540	1365.9	3.1
	70	30	3332 ± 38	1146.2	3.9
	80	30	2442 ± 269	708.2	4.4
	90	30	1351 ± 350	1087.0	4.1
**8**$_{sMAO}$/TIBA	50	30	3126 ± 86	2631.2	2.3
	60	30	3133 ± 33	2437.7	3.0
	70	30	2984 ± 16	2184.5	3.1
	80	30	2881 ± 365	1491.5	4.2
	90	30	2186 ± 50	1385.0	5.0
**9**$_{sMAO}$/TIBA	50	30	2345 ± 42	2768.4	2.1
	60	30	2493 ± 263	2696.2	2.6
	70	30	2548 ± 130	2303.2	3.4
	80	30	2103 ± 186	1970.1	4.0
	90	30	1583 ± 68	1443.8	5.1
**10**$_{sMAO}$/TIBA	50	30	2630 ± 227	3454.7	2.6
	60	30	2817 ± 291	2173.7	3.0
	70	30	2166 ± 50	3100.3	3.0
	80	30	1945 ± 275	2383.0	3.7
	90	30	1187 ± 6	2094.3	5.4
**11**$_{sMAO}$/TIBA	50	30	2536 ± 113	2244.4	2.2
	60	30	2593 ± 52	2113.9	1.7
	70	30	2484 ± 282	2622.7	3.5
	80	30	2071 ± 352	2005.0	3.9
	90	30	1470 ± 149	1658.7	5.2
**12**$_{sMAO}$/TIBA	50	30	1638 ± 20	2368.0	1.9
	60	30	1868 ± 110	2274.0	1.9

	70	30	1751 ± 200	2213.8	2.3
	80	30	1616 ± 132	1828.9	3.4
	90	30	1517 ± 17	1443.0	4.3
**13**$_{sMAO}$/TIBA	50	30	1486 ± 243	2929.6	2.0
	60	30	1869 ± 161	2522.9	2.3
	70	30	1933 ± 252	2173.3	2.9
	80	30	1630 ± 45	1926.4	3.5
	90	30	1424 ± 59	1475.9	4.9
**14**$_{sMAO}$/TIBA	50	30	1379 ± 232	2793.8	2.4
	60	30	1371 ± 49	2608.6	2.4
	70	30	1697 ± 101	2463.0	3.0
	80	30	1596 ± 8	1954.4	4.6
	90	30	1256 ± 38	1559.5	4.5
**15**$_{sMAO}$/TIBA	50	30	682 ± 93	2416.5	1.7
	60	30	759 ± 43	2524.9	2.2
	70	30	785 ± 5	2263.7	2.4
	80	30	725 ± 35	1904.2	3.0
	90	30	623 ± 20	1452.7	4.8

7.1. Structure-activity relationships

Attempts to correlate polymerization activity with structural parameters were made using the slurry-phase ethylene polymerization activity at T_p = 60 °C, the characteristic torsion angle TA', and the ligand shielding parameter G(L). Crucially, the structural parameters are crystallographic measurements of the pre-catalyst complexes in the solid-state and thus have a non-trivial relationship to the dynamic *in operando* structure of the heterogenized, cationized active species. It is shown that conformation interconversion occurs rapidly in solution, and thus the observed crystallographic conformation is merely an indication of the position of the conformational equilibrium.

While there is no clear monotonic correlation with activity, it is apparent that the A-B dichotomy can be classified in terms of both structural parameters: A-type having low TA' and high G(L), B-type having high TA' and low G(L). With the exception of **6**, catalytic activity broadly increases with decreasing TA' and increasing G(L), consistent with the observed high activities for B-type complexes. The chiral complex **6** ({Me,^{n}Pr} *ansa* bridge) is an interesting case compared to **2** ({Me,Me} bridge) and **7** ({Me,Ph} bridge) since the activities of these three compounds are comparable and **2** and **7** are virtually isostructural.

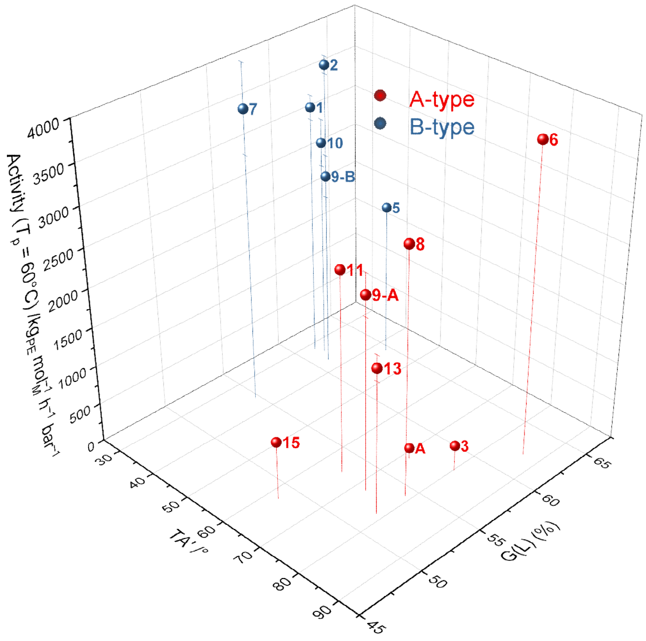

Figure S108 Slurry-phase ethylene polymerization activity of sMAO-supported PHENI* complexes as a function of the torsion angle, TA', and the ligand shielding parameter, G(L).

8. Ethylene uptake profiles

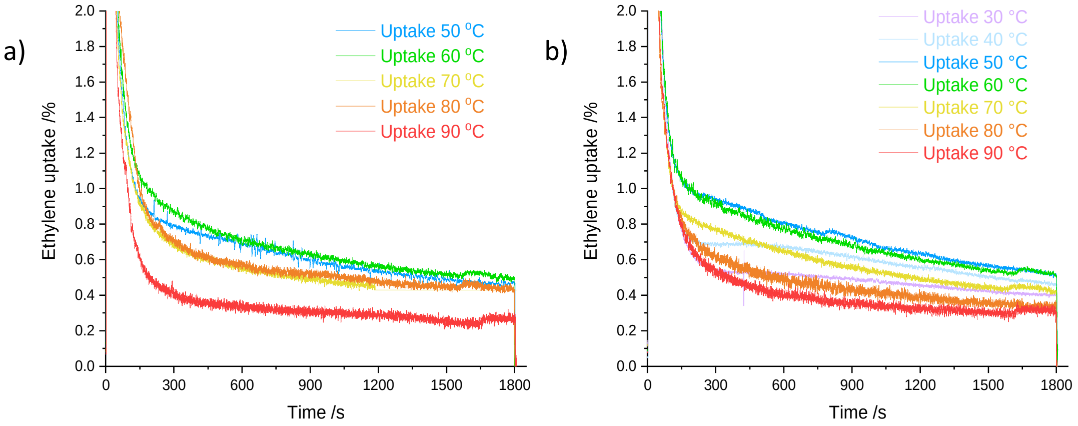

Figure S109 Ethylene uptake as a function of reaction time for a) $\mathbf{1}_{\mathbf{sMAO}}$/TIBA, and b) $\mathbf{2}_{\mathbf{sMAO}}$/TIBA. Polymerization conditions: 10 mg catalyst, 2 bar ethylene, 50 mL hexanes, 30 minutes, and 150 mg TIBA.

9. Gel-permeation chromatography

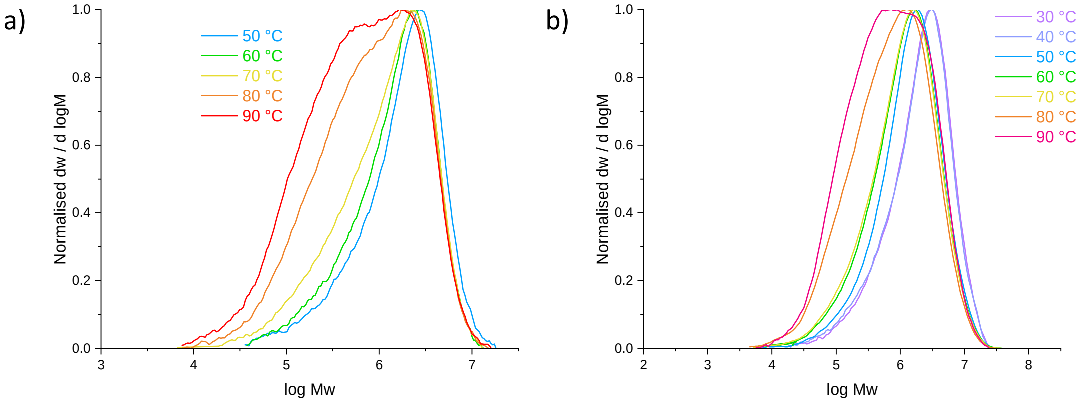

Figure S110 Gel-permeation chromatograms of polyethylene produced by a) $\mathbf{1}_{\mathbf{sMAO}}$/TIBA, or b) $\mathbf{2}_{\mathbf{sMAO}}$/TIBA as a function of temperature. Polymerization conditions: 10 mg catalyst, 2 bar ethylene, 50 mL hexanes, 30 minutes, and 150 mg TIBA.

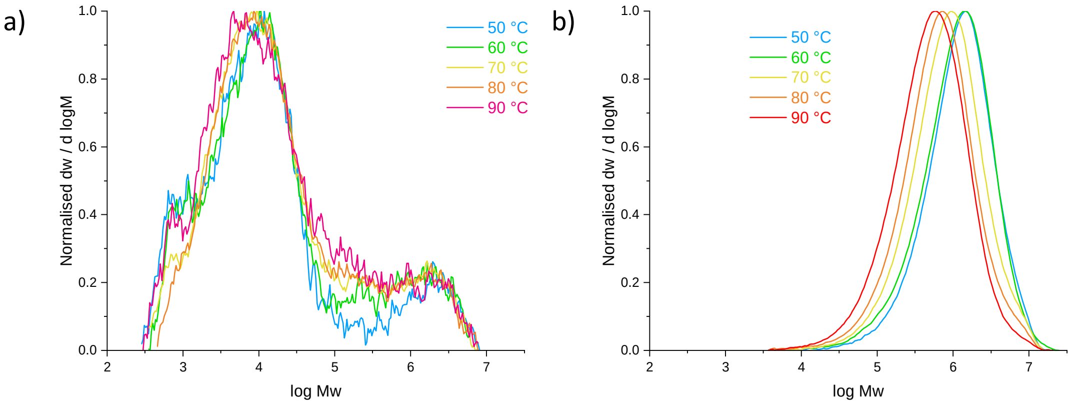

Figure S111 Gel-permeation chromatograms of polyethylene produced by a) **3**$_{sMAO}$/TIBA, or b) **5**$_{sMAO}$/TIBA as a function of temperature. Polymerization conditions: 10 mg catalyst, 2 bar ethylene, 50 mL hexanes, 30 minutes, and 150 mg TIBA.

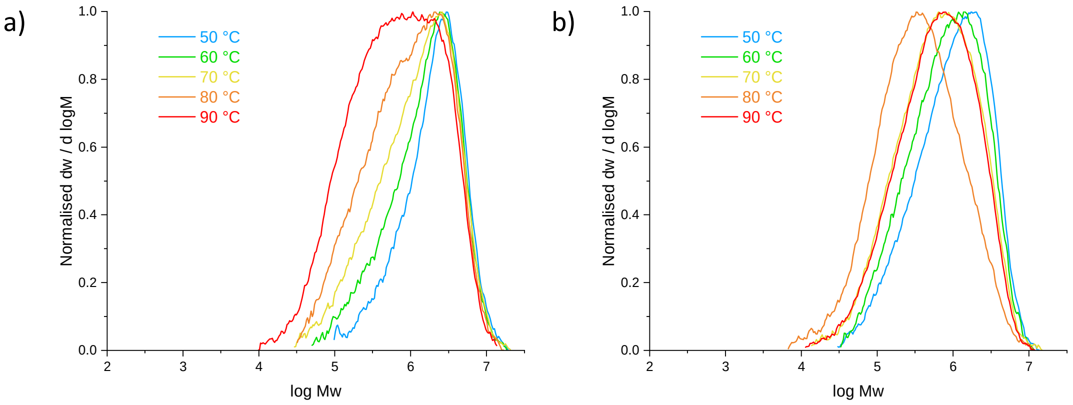

Figure S112 Gel-permeation chromatograms of polyethylene produced by a) **6**$_{sMAO}$/TIBA, or b) **7**$_{sMAO}$/TIBA as a function of temperature. Polymerization conditions: 10 mg catalyst, 2 bar ethylene, 50 mL hexanes, 30 minutes, and 150 mg TIBA.

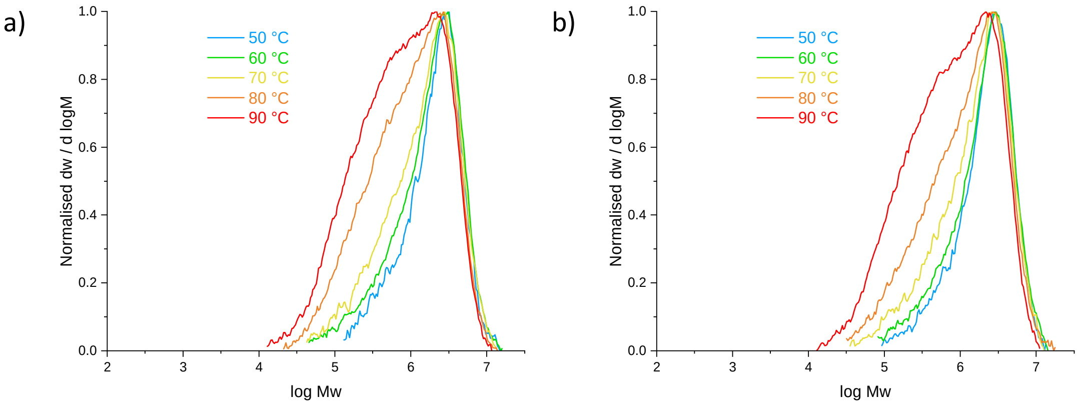

Figure S113 Gel-permeation chromatograms of polyethylene produced by a) **8**$_{sMAO}$/TIBA, or b) **9**$_{sMAO}$/TIBA as a function of temperature. Polymerization conditions: 10 mg catalyst, 2 bar ethylene, 50 mL hexanes, 30 minutes, and 150 mg TIBA.

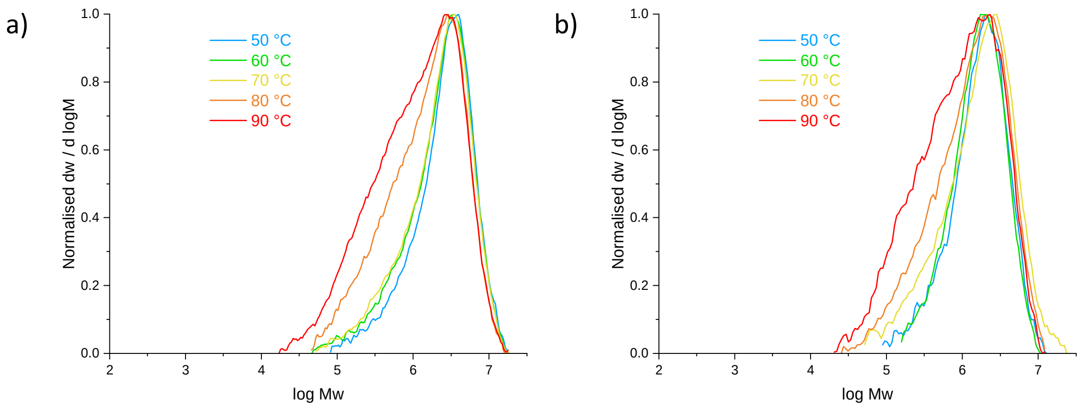

Figure S114 Gel-permeation chromatograms of polyethylene produced by a) **10_{sMAO}**/TIBA, or b) **11_{sMAO}**/TIBA as a function of temperature. Polymerization conditions: 10 mg catalyst, 2 bar ethylene, 50 mL hexanes, 30 minutes, and 150 mg TIBA.

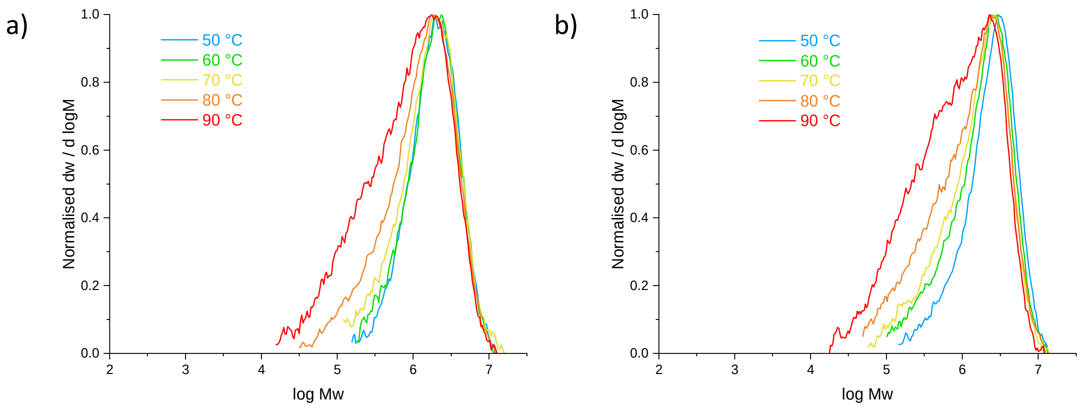

Figure S115 Gel-permeation chromatograms of polyethylene produced by a) **12_{sMAO}**/TIBA, or b) **13_{sMAO}**/TIBA as a function of temperature. Polymerization conditions: 10 mg catalyst, 2 bar ethylene, 50 mL hexanes, 30 minutes, and 150 mg TIBA.

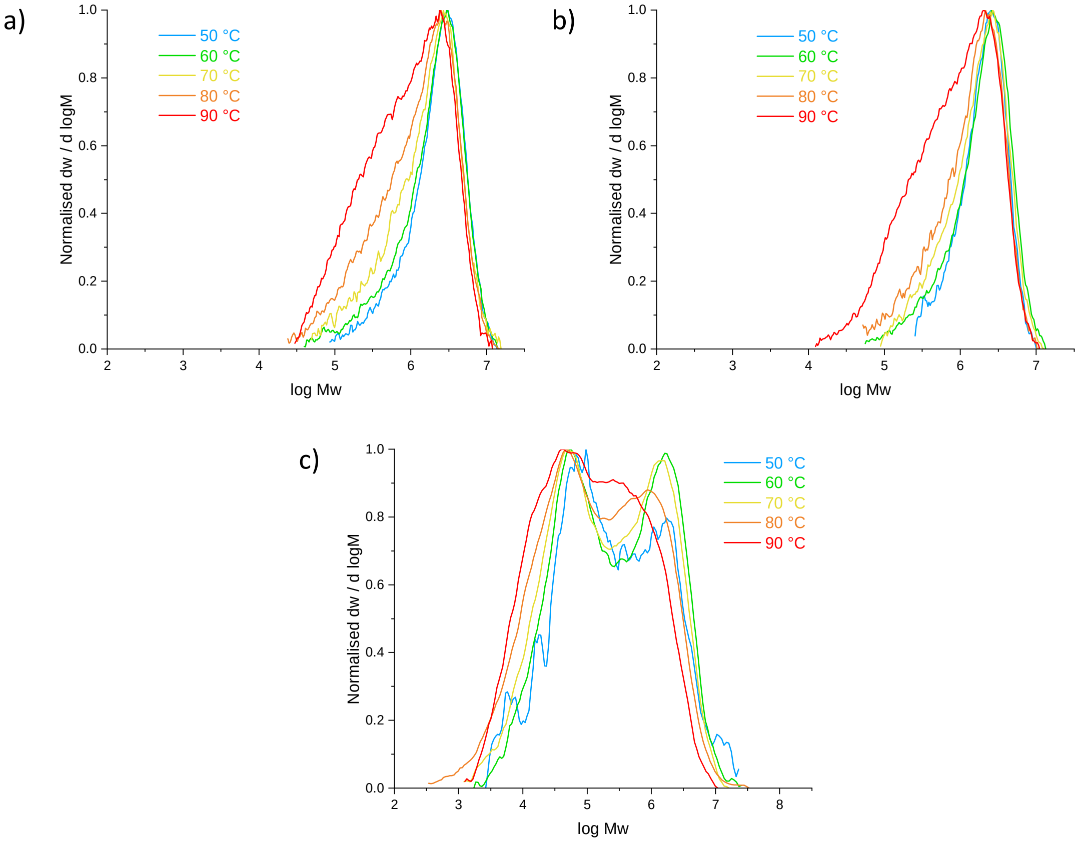

Figure S116 Gel-permeation chromatograms of polyethylene produced by a) $\mathbf{14}_{sMAO}$/TIBA, b) $\mathbf{15}_{sMAO}$/TIBA, or c) $\mathbf{A}_{sMAO}$/TIBA as a function of temperature. Polymerization conditions: 10 mg catalyst, 2 bar ethylene, 50 mL hexanes, 30 minutes, and 150 mg TIBA.

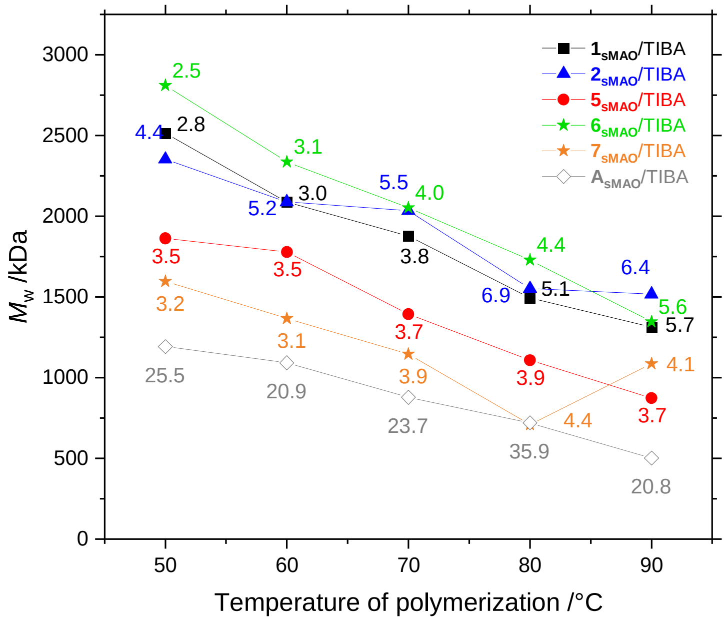

Figure S117 Polyethylene molecular weight (M_w, $Đ$ annotated) as a function of Polymerization temperature for sMAO-supported ^{Me_2}SB(^{tBu,Me}ArO,I*)$TiCl_2$ (**1**), ^{Me_2}SB(^{tBu_2}ArO,I*)$TiCl_2$ (**2**), ^{Me_2}SB(^{Cumyl_2}ArO,I*)$TiCl_2$ (**5**), *rac*-^{Me,nPr}SB(^{tBu_2}ArO,I*)$TiCl_2$ (**6**), *rac*-^{Me,Ph}SB(^{tBu_2}ArO,I*)$TiCl_2$ (**7**), and ^{Me_2}SB(^{tBu,Me}ArO,Ind)$TiCl_2$ (**A**). Polymerization conditions: 10 mg solid catalyst, 150 mg TIBA, 2 bar ethylene, 50 mL hexanes, and 30 minutes.

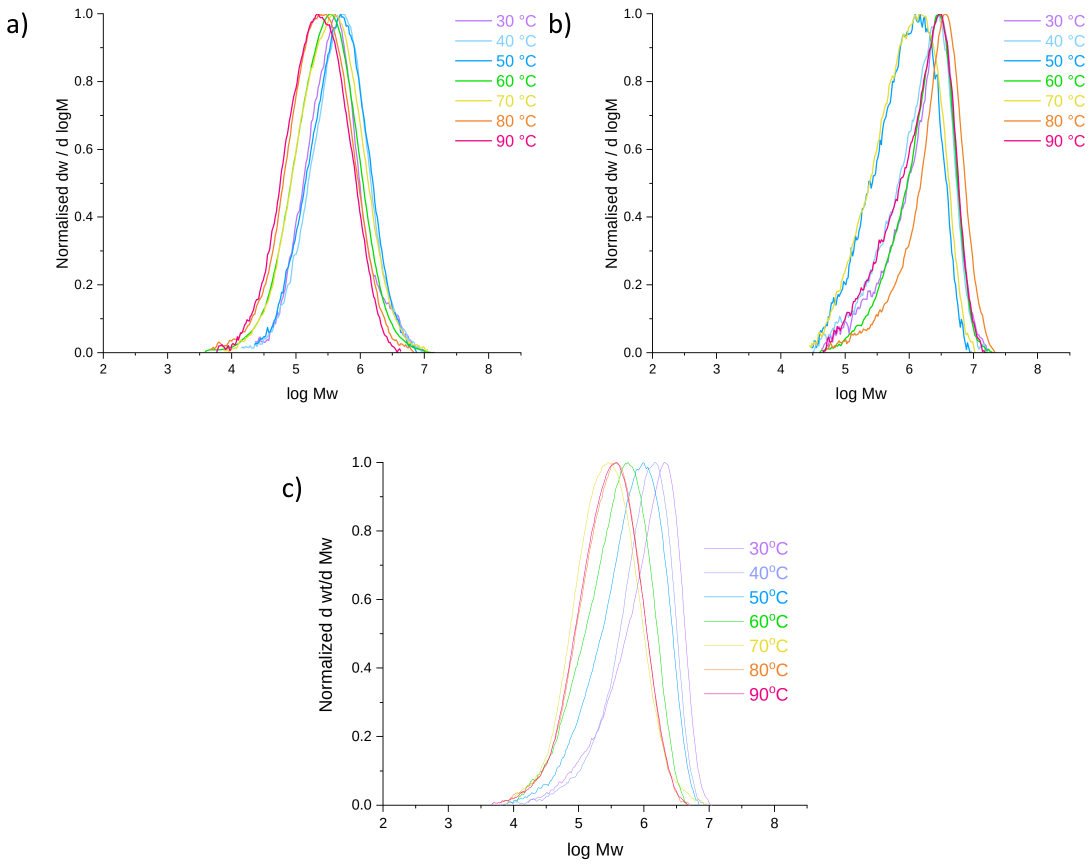

Figure S118 Gel-permeation chromatograms of polyethylene produced by a) **2**/MAO, b) **10**/TB/TIBA, or c) **5**/MAO as a function of temperature. Polymerization conditions: 715 nmol complex, MAO ($[Al_{MAO}]/[Ti] = 1000$) or TB/TIBA ([TB]:[TIBA]:[Ti] = 1:200:1), 2 bar ethylene, 50 mL hexanes, and 5 minutes or until stirring ceased.

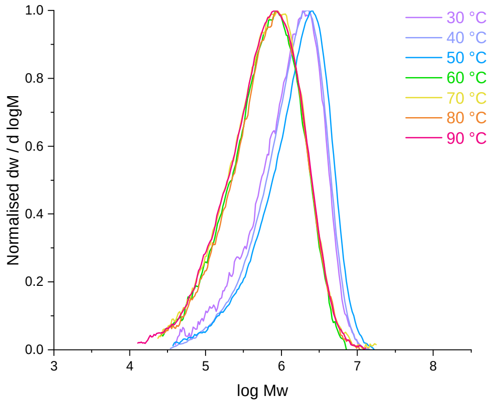

Figure S119 Gel-permeation chromatogram of polyethylene produced by $\mathbf{2_{sMAO}}$/TB as a function of temperature. Polymerization conditions: 10 mg catalyst, 2 bar ethylene, 50 mL hexanes, 30 minutes, and [TB]/[Ti] = 1.

10. Differential scanning calorimetry

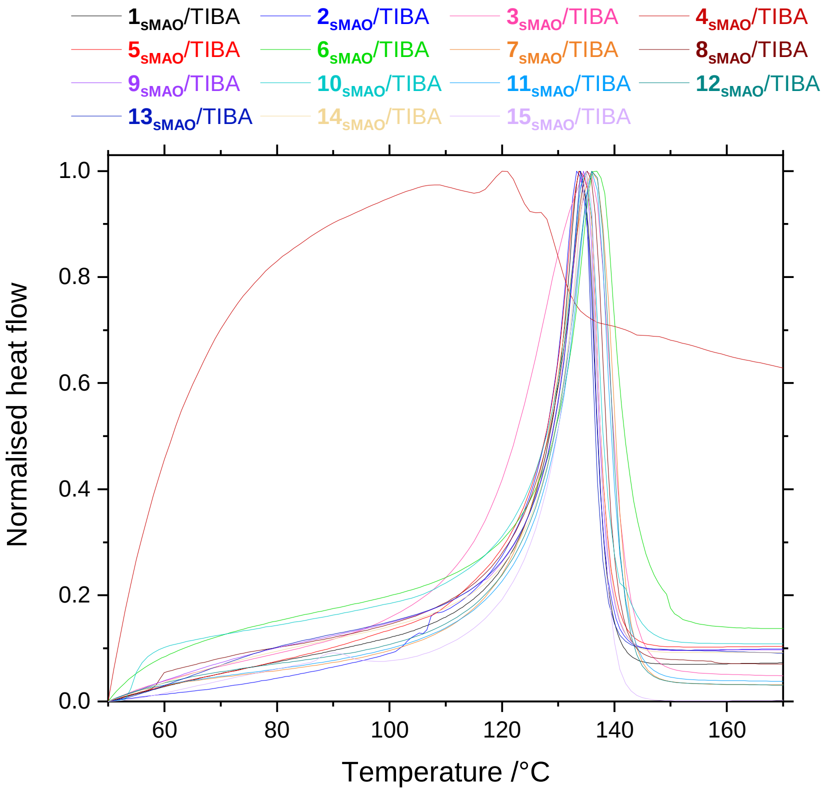

Figure S120 Differential scanning calorimetry plot of polyethylene produced by $\mathbf{1}_{sMAO}$–$\mathbf{15}_{sMAO}$. Polymerization conditions: 10 mg catalyst, 150 mg TIBA, 2 bar ethylene, 50 mL hexanes, 30 minutes, and 60 °C.

Table S6 Melting points and crystallinities of PE produced by PHENI* catalysts $\mathbf{1}_{sMAO}$–$\mathbf{15}_{sMAO}$ at T_p = 60 °C. * The low crystallinity of PE produced by $\mathbf{4}_{sMAO}$/TIBA is attributed to the large amount of catalyst residue present.

Catalyst	T_m /°C	Crystallinity (%)
$\mathbf{1}_{sMAO}$/TIBA	133.7	67.9
$\mathbf{2}_{sMAO}$/TIBA	133.4	77.7
$\mathbf{3}_{sMAO}$/TIBA	135.5	95.0
$\mathbf{4}_{sMAO}$/TIBA	121.0	1.7*
$\mathbf{5}_{sMAO}$/TIBA	133.9	71.3
$\mathbf{6}_{sMAO}$/TIBA	136.4	64.4
$\mathbf{7}_{sMAO}$/TIBA	136.1	89.0
$\mathbf{8}_{sMAO}$/TIBA	135.3	83.0
$\mathbf{9}_{sMAO}$/TIBA	134.5	75.7
$\mathbf{10}_{sMAO}$/TIBA	134.6	70.1
$\mathbf{11}_{sMAO}$/TIBA	135.9	64.3
$\mathbf{12}_{sMAO}$/TIBA	136.3	72.8
$\mathbf{13}_{sMAO}$/TIBA	134.3	67.1
$\mathbf{14}_{sMAO}$/TIBA	134.9	67.2
$\mathbf{15}_{sMAO}$/TIBA	135.3	57.5
$\mathbf{A}_{sMAO}$/TIBA	132.4	76.0

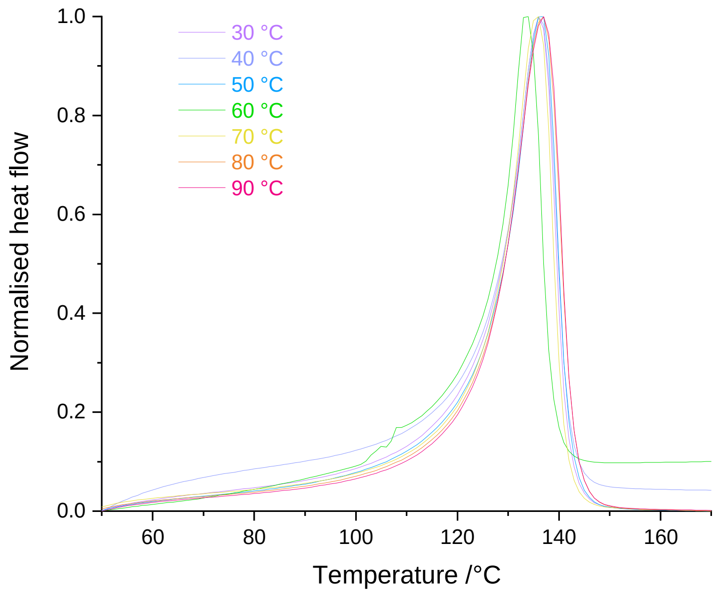

Figure S121 Differential scanning calorimetry plot of polyethylene produced by **2**$_{sMAO}$/TIBA as a function of Polymerization temperature. Polymerization conditions: 10 mg catalyst, 150 mg TIBA, 2 bar ethylene, 50 mL hexanes, 30 minutes, and 30–90 °C.

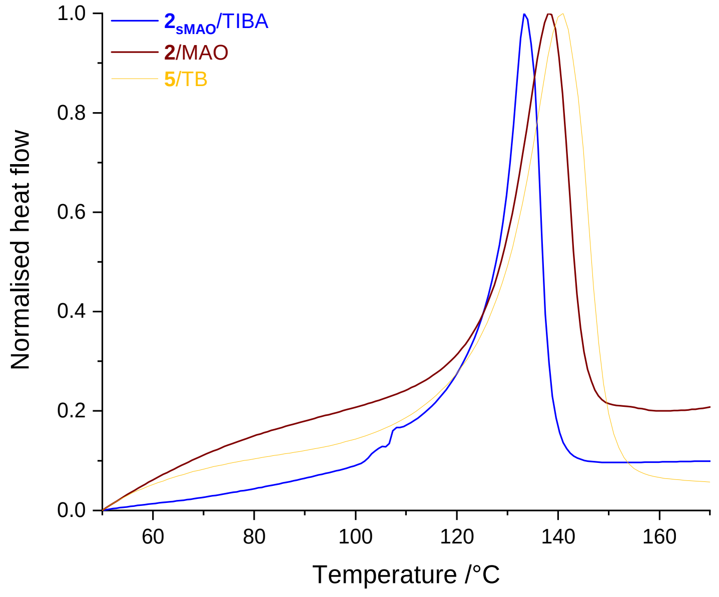

Figure S122 Differential scanning calorimetry plot of polyethylene produced by **2**$_{sMAO}$/TIBA, **2**/MAO, and **5**/TB. Slurry-phase Polymerization conditions: 10 mg catalyst, 150 mg TIBA, 2 bar ethylene, 50 mL hexanes, 30 minutes, and 60 °C. Solution-phase Polymerization conditions: 715 nmol complex, MAO ([Al$_{MAO}$]/[Ti] = 1000) or TB/TIBA ([TB]:[TIBA]:[Ti] = 1:200:1), 2 bar ethylene, 50 mL hexanes, and 5 minutes or until stirring ceased.

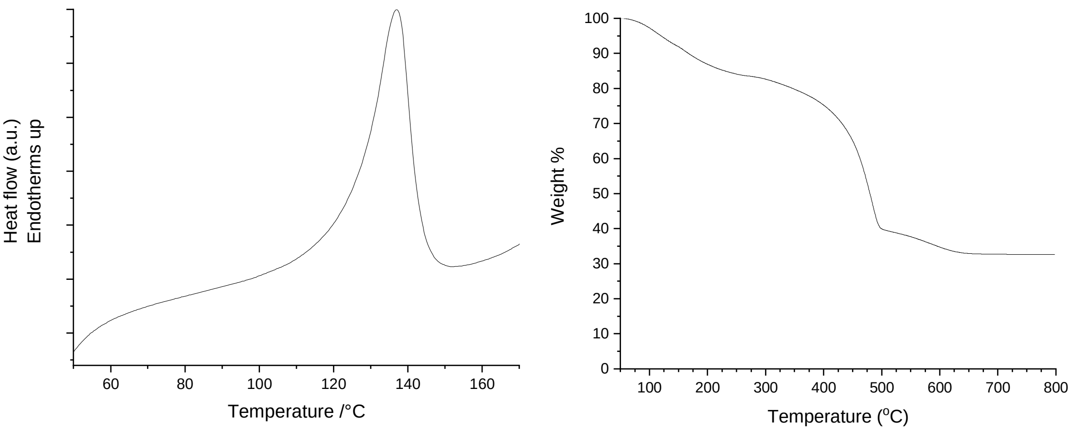

Figure S123 Differential scanning calorimetry (left) and thermogravimetric (right) plots of polyethylene produced by **18**/MAO at 50 °C. Polymerization conditions: 715 nmol complex, MAO ($[Al_{MAO}]/[Ta]$ = 1000), 2 bar ethylene, 50 mL hexanes, and 30 minutes. T_m = 137 °C, α = 14%, residual mass at 800 °C = 32%. Thermogravimetric analyses were performed using a PerkinElmer TGA 8000. Samples were loaded into pre-weighed ceramic crucibles and the weight change was recorded from 30–800 °C at 20 K min^{-1} under a continuous flow of nitrogen.

11. High pressure high-throughput screening

Table S7 Slurry-phase Polymerization data using selected PHENI* catalysts. Polymerization conditions: 0.05–0.80 mg solid catalyst, 10 µmol TIBA, 8.3 bar ethylene, 5 mL heptane, and either 60 minutes or until 120 psi ethylene uptake.

Catalyst	Temperature /°C	Activity /kg_{PE} mol^{-1} h^{-1} bar^{-1}	M_w /kDa	*Đ*
$\mathbf{1}_{sMAO}$/TIBA	40	790 ± 120	4555	1.8
	60	2140 ± 360	4704	2.0
	80	3750 ± 1860	4423	2.1
$\mathbf{2}_{sMAO}$/TIBA	40	1700 ± 120	4256	2.0
	60	4410 ± 660	4708	2.0
	80	6000 ± 590	4610	2.1
$\mathbf{3}_{sMAO}$/TIBA	80	190 ± 20	*n.d.*	*n.d.*
$\mathbf{5}_{sMAO}$/TIBA	40	1210 ± 170	4709	1.8
	60	3225 ± 640	4817	1.8
	80	4200 ± 1400	4472	2.0
$\mathbf{11}_{sMAO}$/TIBA	40	1540 ± 70	4881	2.2
	60	3310 ± 110	4545	2.0
	80	3960 ± 100	4098	2.1
$\mathbf{A}_{sMAO}$/TIBA	40	150 ± 1	2446	6.0
	60	190 ± 30	2263	9.2
	80	110 ± 30	1794	10.3

11.1. Ethylene uptake profiles

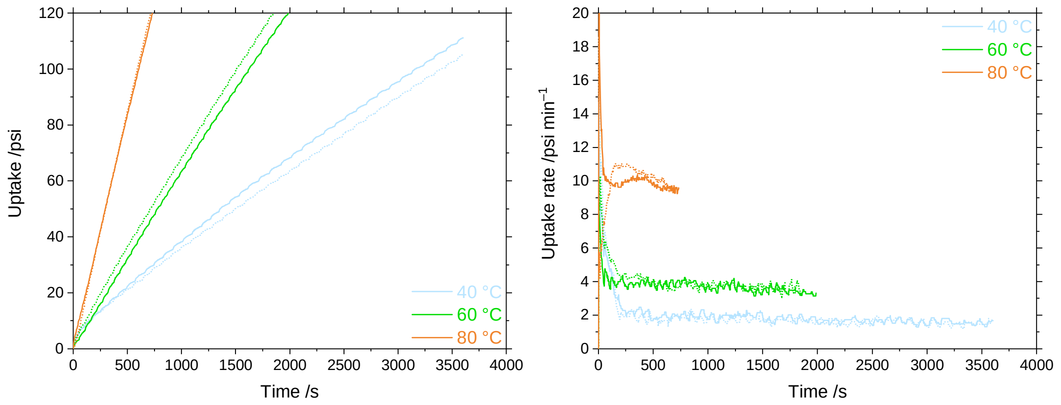

Figure S124 Ethylene uptake (left) and uptake rate (right) of $\mathbf{1}_{sMAO}$/TIBA. Polymerization conditions: 0.05–0.80 mg solid catalyst, 10 µmol TIBA, 8.3 bar ethylene, 5 mL heptane, and either 60 minutes or until 120 psi ethylene uptake.

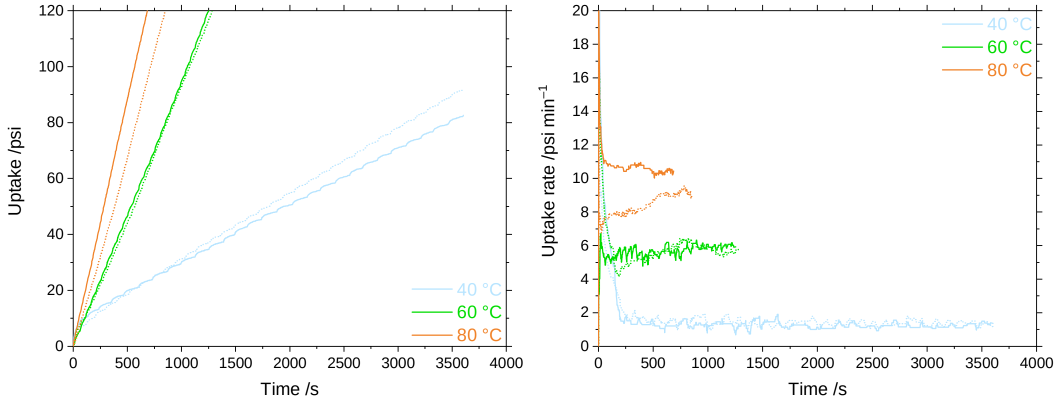

Figure S125 Ethylene uptake (left) and uptake rate (right) of **2**$_{\mathbf{sMAO}}$/TIBA. Polymerization conditions: 0.05–0.80 mg solid catalyst, 10 µmol TIBA, 8.3 bar ethylene, 5 mL heptane, and either 60 minutes or until 120 psi ethylene uptake.

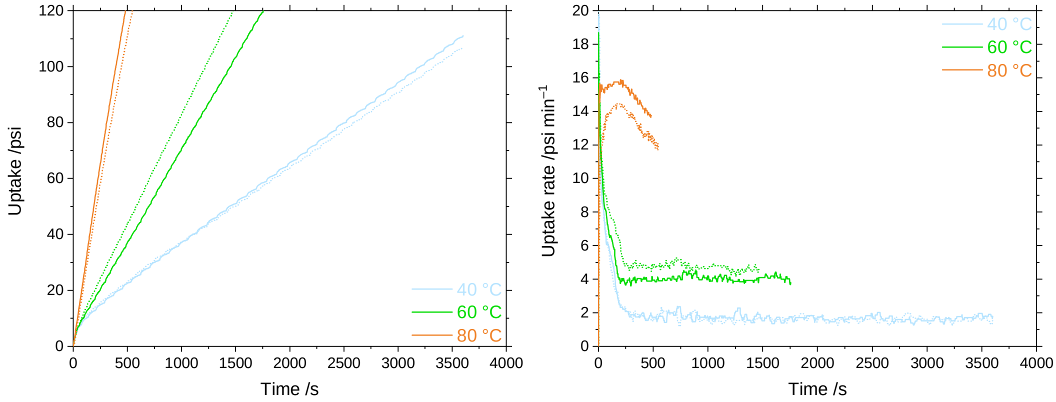

Figure S126 Ethylene uptake (left) and uptake rate (right) of **11**$_{\mathbf{sMAO}}$/TIBA. Polymerization conditions: 0.05–0.80 mg solid catalyst, 10 µmol TIBA, 8.3 bar ethylene, 5 mL heptane, and either 60 minutes or until 120 psi ethylene uptake.

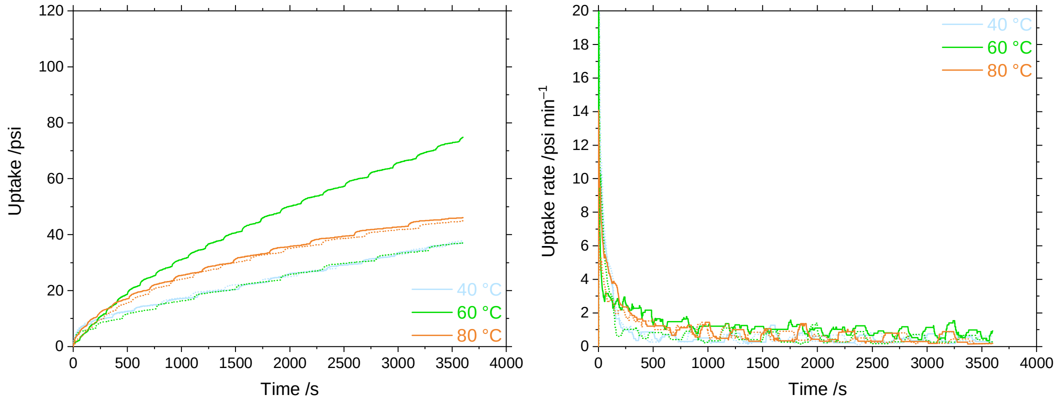

Figure S127 Ethylene uptake (left) and uptake rate (right) of **A**$_{\mathbf{sMAO}}$/TIBA. Polymerization conditions: 0.05–0.80 mg solid catalyst, 10 µmol TIBA, 8.3 bar ethylene, 5 mL heptane, and either 60 minutes or until 120 psi ethylene uptake.

11.2. Gel-permeation chromatography

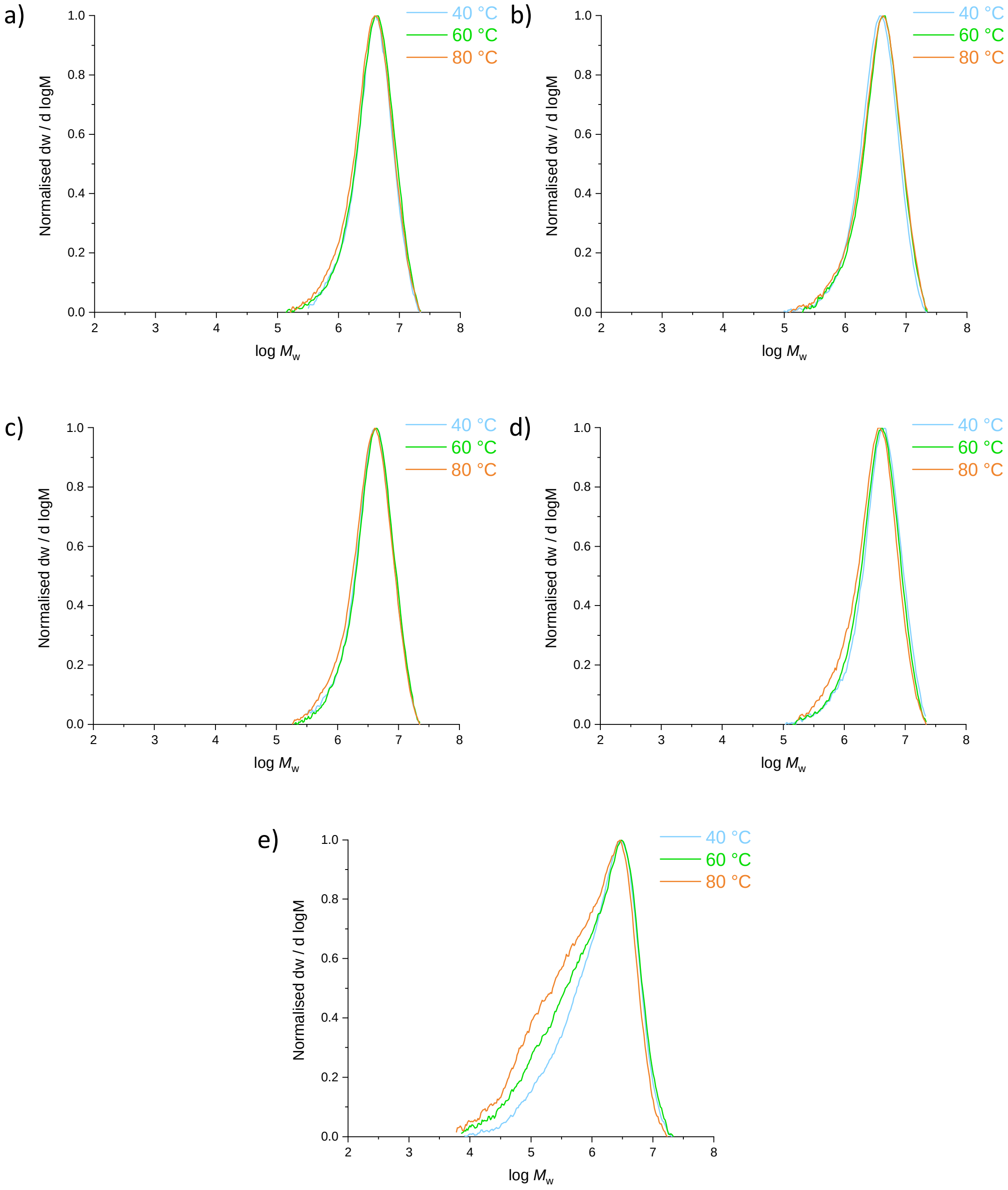

Figure S128 Gel-permeation chromatograms of polyethylene produced by a) $\mathbf{1}_{\mathbf{sMAO}}$/TIBA, b) $\mathbf{2}_{\mathbf{sMAO}}$/TIBA, c) $\mathbf{11}_{\mathbf{sMAO}}$/TIBA, d) $\mathbf{5}_{\mathbf{sMAO}}$/TIBA, or e) **A**/TIBA as a function of temperature. Polymerization conditions: 0.05–0.80 mg solid catalyst, 10 µmol TIBA, 8.3 bar ethylene, 5 mL heptane, and either 60 minutes or until 120 psi ethylene uptake.

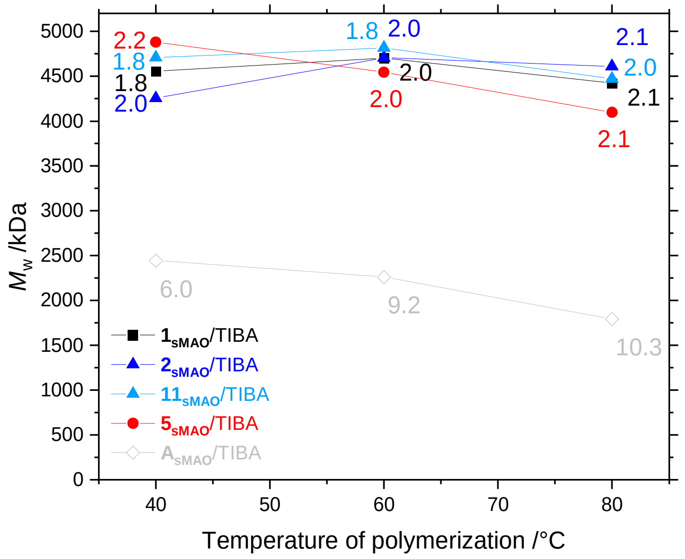

Figure S129 Polymer molecular weight (M_w, *Đ* annotated) as a function of Polymerization temperature for sMAO-supported ^{Me2}SB(^{tBu,Me}ArO,I*)TiCl$_2$ (**1**), ^{Me2}SB(^{tBu2}ArO,I*)TiCl$_2$ (**2**), ^{Me2}SB(^{tBu2}ArO,I*)Ti(CH$_2$SiMe$_3$)$_2$ (**11**), ^{Me2}SB(^{Cumyl2}ArO,I*)TiCl$_2$ (**5**), and ^{Me2}SB(^{tBu,Me}ArO,Ind)TiCl$_2$ (**A**). Polymerization conditions: 0.05–0.80 mg solid catalyst, 10 µmol TIBA, 8.3 bar ethylene, 5 mL heptane, and either 60 minutes or until 120 psi ethylene uptake.

12. References

1 R. D. Shannon, *Acta Crystallogr. A*, 1976, **32**, 751-767.
2 T. Senda, H. Hanaoka, Y. Oda, H. Tsurugi, K. Mashima, *Organometallics*, 2010, **29**, 2080-2084.
3 M. G. Thorn, J. R. Parker, P. E. Fanwick, I. P. Rothwell, *Organometallics*, 2003, **22**, 4658-4664.
4 J. S. Vilardo, M. M. Salberg, J. R. Parker, P. E. Fanwick, I. P. Rothwell, *Inorg. Chim. Acta*, 2000, **299**, 135-141.
5 V. C. Gibson, T. P. Kee, W. Clegg, *J. Chem. Soc., Dalton Trans.*, 1990, 3199-3210.
6 H. W. Roesky, F. Schrumpf, M. Noltemeyer, *Z. Naturforsch. B*, 1989, **44**, 35-40.
7 A. W. Addison, T. N. Rao, J. Reedijk, J. van Rijn, G. C. Verschoor, *J. Chem. Soc., Dalton Trans.*, 1984, 1349-1356.
8 I. A. Guzei and M. Wendt, *Dalton Trans.*, 2006, 3991-3999.
9 F. Neese, *WIREs Comput. Mol. Sci.*, 2012, **2**, 73-78.
10 F. Neese, F. Wennmohs, U. Becker, C. Riplinger, *J. Chem. Phys.*, 2020, **152**.
11 F. Neese, *WIREs Comput. Mol. Sci.*, 2022, **12**, e1606.
12 A. D. Becke, *J. Chem. Phys.*, 1986, **84**, 4524-4529.
13 C. Lee, W. Yang, R. G. Parr, *Phys. Rev. B Condens. Matter*, 1988, **37**, 785.
14 A. D. Becke, *J. Chem. Phys.*, 1993, **98**, 5648-5652.
15 D. A. Pantazis, X. Y. Chen, C. R. Landis, F. Neese, *J. Chem. Theory Comput.*, 2008, **4**, 908-919.
16 D. A. Pantazis and F. Neese, *J. Chem. Theory Comput.*, 2009, **5**, 2229-2238.
17 S. Grimme, J. Antony, S. Ehrlich, H. Krieg, *J. Chem. Phys.*, 2010, **132**, 154104.
18 F. Neese, F. Wennmohs, A. Hansen, U. Becker, *Chem. Phys.*, 2009, **356**, 98-109.
19 R. Izsák and F. Neese, *J. Chem. Phys.*, 2011, **135**.
